# A gene signature based method for identifying subtypes and subtype-specific drivers in cancer with an application to medulloblastoma

**DOI:** 10.1186/1471-2105-14-S18-S1

**Published:** 2013-11-05

**Authors:** Peikai Chen, Yubo Fan, Tsz-kwong Man, YS Hung, Ching C Lau, Stephen TC Wong

**Affiliations:** 1 Department of Electrical and Electronic Engineering, The University of Hong Kong, Pokfulam Road, Hong Kong; 2 Department of Systems Medicine and Bioengineering, The Methodist Hospital Research Institute, Weill Cornell Medical College, Houston, TX 77030, USA; 3 Texas Children's Cancer and Hematology Centers and Dan L. Duncan Cancer Center, Baylor College of Medicine, Houston, TX 77030, USA; 4 Department of Pediatrics, Baylor College of Medicine, Houston, TX 77030, USA; 5 Structural and Computational Biology and Molecular Biophysics Program, Baylor College of Medicine, Houston, TX 77030, USA

**Keywords:** subtypes of cancer, medulloblastoma, gene signature, copy number aberrations, microarrays, driver genes

## Abstract

**Background:**

Subtypes are widely found in cancer. They are characterized with different behaviors in clinical and molecular profiles, such as survival rates, gene signature and copy number aberrations (CNAs). While cancer is generally believed to have been caused by genetic aberrations, the number of such events is tremendous in the cancer tissue and only a small subset of them may be tumorigenic. On the other hand, gene expression signature of a subtype represents residuals of the subtype-specific cancer mechanisms. Using high-throughput data to link these factors to define subtype boundaries and identify subtype-specific drivers, is a promising yet largely unexplored topic.

**Results:**

We report a systematic method to automate the identification of cancer subtypes and candidate drivers. Specifically, we propose an iterative algorithm that alternates between gene expression clustering and gene signature selection. We applied the method to datasets of the pediatric cerebellar tumor medulloblastoma (MB). The subtyping algorithm consistently converges on multiple datasets of medulloblastoma, and the converged signatures and copy number landscapes are also found to be highly reproducible across the datasets. Based on the identified subtypes, we developed a PCA-based approach for subtype-specific identification of cancer drivers. The top-ranked driver candidates are found to be enriched with known pathways in certain subtypes of MB. This might reveal new understandings for these subtypes.

This article is an extended abstract of our ICCABS '12 paper (Chen *et al.* 2012), with revised methods in iterative subtyping, the use of canonical correlation analysis for driver-identification, and an extra dataset (Northcott90 dataset) for cross-validations. Discussions of the algorithm performance and of the slightly different gene lists identified are also added.

**Conclusions:**

Our study indicates that subtype-signature defines the subtype boundaries, characterizes the subtype-specific processes and can be used to prioritize signature-related drivers.

## Background

Cancer is initiated and driven by aberrant genetic events, such as copy number aberrations (CNAs), called the drivers of cancer. Further, quite a few cancer, such as breast cancer [1], glioblastoma [2] and medulloblastoma [3], are confirmed to contain subtypes, with distinct inter-subtype molecular profiles and clinical outcomes. Different subtypes may have arisen because of different mechanisms, such as hits on different pathways and/or different cells-of-origin [4] within the same tissue/organ. Stratifying the patients into appropriate subtypes is the key to uncover the drivers of these mechanisms. This step, referred to as the subtyping of cancer, usually relies on the class discovery of cancer expression datasets.

There is a large body of techniques available for class discovery within a cancer dataset, such as spectral clustering [5], non-negative matrix factorization [6], etc. A straightforward and one-step strategy is to employ one such technique to train the class labels for a group of samples, and detect the set of most differentially expressed genes (DEGs) using the trained labels and the same expression data. The set of DEGs, called the gene signature, can be used for functional analysis of the corresponding subtype.

**Figure 1:**
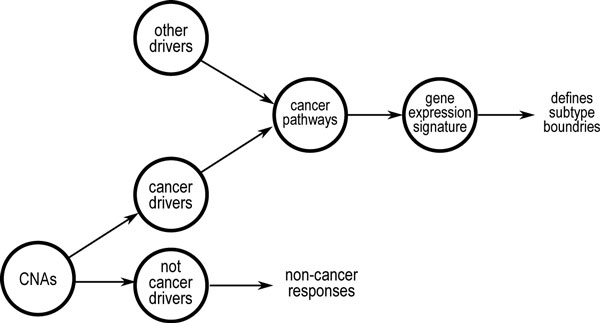
**A hypothetical diagram depicting the role of CNAs in cancer, subtypes and their relations with signatures**. While most CNAs are assumed to affect the expressions of the affected genes only and have limited harms, a few CNAs together with other drivers, e.g. mutations, may perturb subtype-specific pathways and trigger subtypes of cancer. This consequently leads to the observation of a set of signature genes.

On the other hand, genetic aberrations enriched in a cancer subtype may also be related to, or even have causal roles in the corresponding subtype. Particularly, one type of genetic events, CNA, is widely found in the cancer genomes [7]. CNAs are also found to be positively correlated with the raw expressions of affected genes [3]. In some cancer, such as medulloblastoma, the CNA patterns are also found to be subtype-dependent [8]. However, given the large number of CNA-affected genes that often occur within a cancer genome, it is not practical to assume that all of them are tumorigenic. Instead, most of the CNAs may just cause mechanic responses in the affected genes' expressions, but otherwise are not related to the cancer process. Apart from these, a small proportion of CNAs may be involved in the initiating, driving or sustaining of the cancer process, which also gives rise to the subtype-specific signature. This is summarized in the hypothetical diagram in Figure 1. The diagram indicates that the gene signature not only reflects the underlying processes characterizing individual subtypes and hence can be used for subtyping; but may also be used to trace the subtype-specific drivers.

In recent years, tools such as GISTIC [9] have gained increasing popularity for their capabilities to discover recurrent CNA patterns of a clinical condition. Genes within these recurrent patterns are assumed to be pathologically and clinically important. Unfortunately, in the highly twisted cancer genomes, recurrent regions tend to occupy broad regions and harbor hundreds of genes or more, making it less specific to equate these genes to cancer drivers. A recent study [10] attempts to relate recurrent CNAs with co-expression modules, in an effort to find melanoma drivers, in a subtype-non-specific manner.

**Figure 2:**
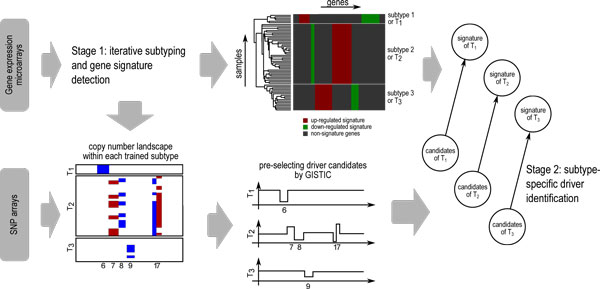
**Approach overview**. Gene expression microarray measurements are used for training the class labels and detecting signature genes. The copy number profiles (by SNP arrays) of the corresponding samples undergo candidate pre-selection by GISTIC. A subtype-specific method identifies top signature-related candidates.

Here, we focus on the subtype-signature and within-subtype recurrent CNAs; and propose an integrative approach to perform subtyping and driver-identification. The approach consists of two stages. First, given a cancer expression dataset, perform subtyping to train class labels for individual cases and detect signature genes for each subtype (i.e., class). Second, CNA measurements (e.g., SNP arrays) corresponding to samples of each subtype are subjected to pre-selection procedures, e.g. GISTIC, for a reduced set of driver candidates. The driver candidates are then ranked in order of correlation with the corresponding subtype's signature genes. The top ranked candidates will be manually reviewed for their potential roles in the subtypes. The approach is summarized in Figure 2. The following describes key issues regarding the algorithmic design for these two stages at the concept level, with further details in Methods.

### Iterative subtyping

The one-step approach described above is both easy to implement and computationally efficient. However, it may suffer from overfitting, as the determination of the number of clusters given a clustering dendrogram can be quite arbitrary. This is because the relation between the class labels and the signature genes is not considered. Suppose there are *K *inherent subtypes, each with a set of signature genes, the large number of non-signature genes, together with measurement noises, may blur the subtype boundaries. This results in multiple ways of cutting a dendrogram, as in the case of medulloblastoma where various numbers of clusters have been reported [3,11]. As a result, different DEGs will be produced by different methods on the same disease. Yet as depicted in Figure 1, the real pathway-related DEGs (i.e., signature) should be inherent in each subtype but not dependent on how the dendrogram is cut.

To address this issue, we first introduce a regularization by defining a subtype as the smallest group of samples that share a substantial set of DEGs (against all other samples, including normal cases). This is to ensure that on the one hand, as many subtypes are uncovered as possible, while on the other hand, each of them is characterized with a sufficient number of signature genes. In practice, this can be implemented by starting with a large number and cutting the resulting dendrogram into corresponding number of clusters. The subtype-signature detection (described in Methods) is applied to the resulting clusters. The resulting clusters can be categorized into *good clusters *and *bad clusters*. The former has a sufficiently large number of signature genes, while the latter has fewer than a certain percentage of total signature genes. Cases of the bad clusters are re-assigned to their closest good clusters. Further, since the inherent signature genes determine the class boundary more than non-signature genes, the union of the signature genes after one iteration may be used as input to inform the subset of genes on which clustering is to be performed, in the next iteration.

The next iteration will attempt to cut the dendrogram into the same number of clusters as the good clusters in last iteration. This is based on the assumption that each step of clustering and signature detection produces a set of signature genes that is getting closer to the genuine signatures. The above procedure ensures that the number of clusters to be tested is monotonously decreasing. To avoid *K *being trapped in small numbers, at each iteration, the number of clusters to be tested is increased by a small margin Δ*K *above the number good clusters in the last step. In this way, this algorithm is made to favor a larger number of patterns over smaller ones, if both have sufficient numbers of signatures genes.

The above procedure is iterated until the algorithm converges, indicating that the optimum where maximum number of subtypes each with a sizable signature has been reached. Our iterative approach can be compared to the iterative algorithm for a bounded (and regularized) optimization problem. In cutting the dendrograms, resulting clusters that contain only one case are considered unlikely to be a subtype and are re-assigned to other *core clusters *(i.e., containing more than one case). The procedural implementation of the algorithm is given below.

Given an expression dataset $E\in {\mathbb{R}}^{N\times M}$ with *N *samples and *M *genes, select an initial set of genes, say the top 10% of genes with largest variances, to yield a reduced dataset  Ê. Start with a (sufficiently) large number *K*, the algorithm consists of the following steps:

1. Perform clustering (e.g., spectral clustering) on  Ê, and form a dendrogram.

2. Cut the dendrogram into *K *clusters. If this results in some clusters with one sample, assign these samples to their closest clusters. Consequently, we obtain *K*′ *core clusters *(*K′ *≤ *K*).

3. Detect the signature (described in Methods) *S_k _*for each subtype *k *∈ {1, .., *K′*}, using the original dataset *E*.

4. Obtain the union of signatures: Φ = *S*_1 _∪ … ∪ *S_K′_*.

5. For clusters (i.e., bad clusters) whose signature genes are fewer than a percentage of |Φ|, the cases in these clusters are re-assigned to their closest good clusters. This results in a total number of clusters *K″ *≤ *K′*.

6. Replace the above  Ê with one that is based on the set Φ.

7. Update *K *= *K*″ + Δ*K*, where Δ*K *is a small positive integer.

8. Repeat Steps 1 to 7, till a certain convergence criterion is reached, e.g. the number of subtypes remains unchanged.

### The subtype-specific driver identification

According to Figure 1, even within a subtype, there could be different driving events. Here, we focus on the dominant subtype-specific events, i.e., CNAs. A CNA-affected gene can activate the cancer process through its CNA-induced aberrant expressions. Therefore, its expression may be correlated with the signature genes, which are believed to be the consequences of subtype-specific processes. This problem is equivalent to finding variables in one set that maximally correlate with another set of variables, a problem known as canonical correlation analysis (CCA) [12]. Particularly, given a subtype *k *and its signature $S_{k}\left(|S_{k}|\overset{\text{Δ}}{=}J\right)$ and *L *pre-selected candidate CNA genes, two vectors of variables ${\text{x}}_{a}\in R^{J\times \text{1}}$ and ${\text{x}}_{b}\in R^{L\times \text{1}}$are used to denote the expressions of them, respectively. The objective is find two vectors ${\text{w}}_{a}\in R^{J\times \text{1}}$ and ${\text{w}}_{b}\in R^{L\times \text{1}}$, such that:

(1) $$\rho =\frac{{\text{w}}_{a}^{T}C_{ab}{\text{w}}_{b}}{\sqrt{{\text{w}}_{a}^{T}C_{aa}{\text{w}}_{a}{\text{w}}_{b}^{T}C_{bb}{\text{w}}_{b}}}$$

is maximized, where *C_ab _*is the between-set covariance matrix and *C_aa _*and *C_bb _*are the within-set covariance matrices. The pre-selected candidates whose absolute weightings $|w_{b}^{l}|\left(l=\text{1},\;...,\;L\right)$ are largest are assumed to be related with the signature genes, whereas those whose weightings are close to zero as assumed to be not related. Similarly, signature genes whose absolute weightings are large can be assumed to be related with the candidates. If thresholds are chosen, solutions to the above problem result in a CNA-regulated network consisting of subsets of signature genes and pre-selected candidates.

In solving Eq. (1), techniques such as sparse canonical correlation analysis (SCCA) [13] kernelize the covariance matrices, and reduce the problem to:

(2) $$\underset{{\text{w}}_{a},{\text{w}}_{b}}{\text{maximize}}\;{\text{w}}_{a}^{T}C_{ab}{\text{w}}_{b}+\mu |{\text{w}}_{a}|+\mu |{\text{w}}_{b}|$$

subject to ${\text{w}}_{a}^{T}C_{aa}{\text{w}}_{a}={\text{w}}_{a}^{T}C_{bb}{\text{w}}_{b}=1.$. This avoids the hard thresholds above, but the solution is highly sensitive to the hyperparameter *µ*. Note that the magnitudes of w*_a _*and w*_b _*do not matter, e.g., if ${\text{w}}_{a}^{T}C_{aa}{\text{w}}_{a}\neq 1$, one can replace w*_a _*with ${\tilde{\text{w}}}_{a}={\text{w}}_{a}/\sqrt{{\text{w}}_{a}^{T}C_{aa}{\text{w}}_{a}}$. The key becomes finding the directions of **w***_a _*and **w***_b _*such ${\text{w}}_{a}^{T}C_{ab}{\text{w}}_{b}$ is maximized. This is similar to the idea of principal component analysis (PCA). Specifically, we may assume that a row-wise zero-mean operation has been applied to *C_ab_*. A PCA approach can next be applied to determine the correlation of each CNA with the set *S_k_*, as follows:

a. Perform an $\text{SVD}:C_{ab}=U\Sigma V^{T}$, and then project *C_ab _*onto the first principal component **u**_1 _of *U*, to give ${ŵ}_{b}=C_{ab}^{T}{\text{u}}_{1}$. The individual entry of ${ŵ}_{b}$ represents the overall correlation of each CNA gene with the set of signature genes.

b. For a candidate CNA gene *l *∈ {1, .., *L*}, the more positive ${ŵ}_{b}^{l}$ the more gene *l *is positively correlated with *S_k_*, and vice versa. Therefore, the values of ${ŵ}_{b}$ serve as indicators of driver potential for the candidate CNA genes. For convenience, ${ŵ}_{b}^{l}$ referred to as the driver potential of a candidate.

c. The *p*-values of ${ŵ}_{b}$ can be obtained by generating a random set of signature genes and repeating the above procedure to produce a null distribution for ${ŵ}_{b}$. The candidate drivers can then be ranked by their *p*-values.

In the above, if we let ${ŵ}_{a}=u_{1}$, then ${ŵ}_{a}^{T}C_{ab}{ŵ}_{b}={\text{u}}_{1}^{T}C_{ab}C_{ab}^{T}{\text{u}}_{1}=\sigma_{1}^{2}$, where *σ*_1 _is largest singular value of *C_ab_*. Note that the above procedure may not lead to the maximum value for *ρ *in Eq. (1), but as shall be shown in the Results, it effectively detects signature-related candidates.

## Results and discussion

To implement the proposed approach, we applied it to medulloblastoma (MB) datasets. MB is a pediatric brain tumor that usually affects children below the age of 15. The overall five-year survival rate for MB-affected children is poor (around 50% [14]) and varies a lot from patient to patient, subject to different predisposition conditions. Integrative genomic studies [3,8] in recent years have attempted to classify MB patients into various numbers of subtypes, two of which are well-accepted, namely, the *Wnt*- and *Shh-*pathway associating subtypes, respectively. For the remaining non-*Wnt*/non-*Shh *patients, there are still debates on the exact number of subtypes inside this group.

### Dataset-independent convergence of the subtyping algorithm

**Figure 3:**
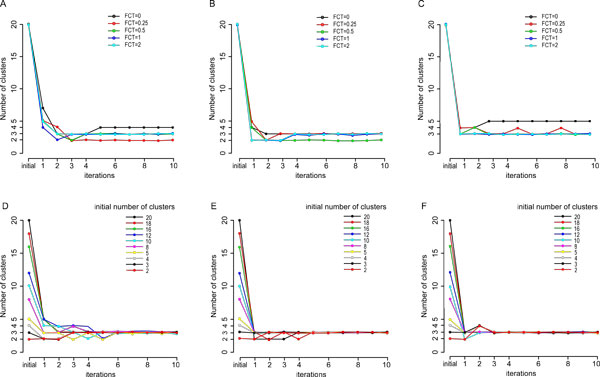
**Convergence of the iterative subtyping algorithm**. A-C, The convergence properties of the subtyping algorithm on the three datasets, Cho73 (A), Northcott90 (B) and Kool62 (C), each with various subtype-specific FCTs. D-F, The convergence properties to the three medulloblastoma datasets as above, each with various initial numbers of clusters ranging from 2 to 20. The FCT of 1.0 is used in all experiments in D-F.

We applied the subtyping algorithm to three expression datasets of medulloblastoma. The set of genes with top 10% variances was chosen to be the initial gene set for the algorithm. Clusters with fewer than 1*/*(4*K′*) of all detected signature genes were determined to be bad clusters, where *K′ *is the *core cluster *number as defined in the algorithm above, and Δ*K *was set to 1. The key parameter that defines the degree of specificity of a DEG, the subtype-specific fold change threshold (FCT) (described in Methods), was varied from 0 to 2.0 on all three datasets (with initial cluster number fixed at 20), to assess the convergence properties of the algorithm. As shown in Figure 3A-C, in all three datasets, as the FCT increases, the convergence is enhanced. For example, when FCT is 1.0 or 2.0, the algorithm efficiently converges to three in all datasets. Whereas, when the FCT is small, e.g., 0 (black curves), 0.25 (red curves) or 0.5 (green curves), a slight change in the FCT causes the algorithm to converge to different numbers of clusters (the red and black curves in A), or not converged at all (the red curve in C). This suggests that the subtype-specificity imposed on signature selection does increase the convergence of the iterative algorithm.

**Figure 4:**
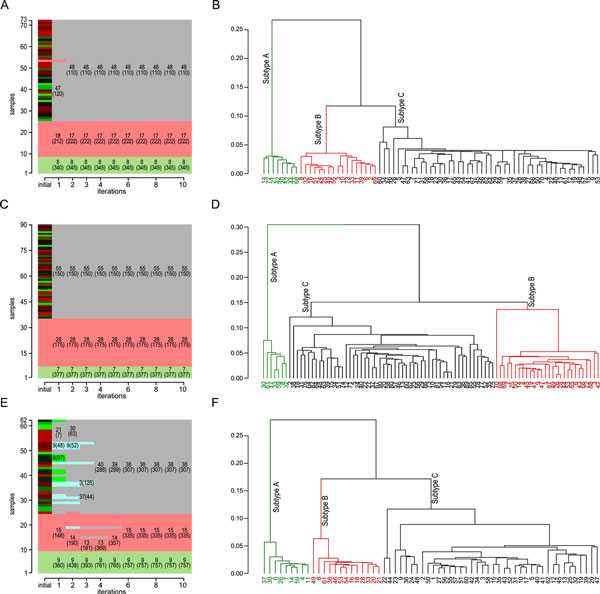
**The subtypes and signatures as the algorithm converges**. The numbers of cases and signatures (in brackets) in each good (and core) clusters as the algorithm converges (in 10 iterations) on three datasets, Cho73 (A), Northcott90 (C) and Kool62 (E). The cases are ordered according to subtyping of the last (converged) iteration. All experiments were started with 20 initial clusters, FCT set to 1.0 and Δ*K *= 1. The converged dendrograms of the corresponding datasets are shown in B, D and F, respectively.

We then fixed the FCT to be 1.0, and varied the initial number of clusters, from 2 to 20, as shown in Figure 3D-F. Again, the algorithm converges within several iterations on all datasets to the same number of clusters, namely three clusters. Figure 4 shows the numbers of cases and signature genes in each subtype as the algorithm converges, and the converged dendrograms, after 10 iterations. As a comparison, the aforementioned one-step approach using the same initial set of genes and same clustering method (i.e., spectral clustering) was applied to the three MB datasets and the resulting dendrograms are shown in Figure S1 (Additional file 1). It appears that the converged dendrograms in Figure 4 demonstrate much clearer subtype boundaries than the corresponding dendrograms in Figure S1.

### Cross-dataset validations of identified subtypes and characterization of them by signatures

Since the datasets all converge to three subtypes, they are labeled as subtypes A, B and C. To test if the converged subtypes are dataset-independent, we performed two analyses. First, the converged signatures of the datasets were compared in a pairwise manner, as shown in Table 1. From the table, the detected signatures are highly specific to their corresponding subtypes and highly reproducible on different datasets. Second, we performed cross-validations by using one dataset and its trained labels and signatures to predict the labels of another dataset, and vice versa. The datasets were normalized (described in Methods) before being used for cross-validations and the *k*-NN method (*k *= 3) was used to predict the labels of the testing sets. The predicted labels were compared with the testing set's self-trained labels to form a confusion matrix, from which the accuracy can be computed. Table 2 shows the cross-validation results. All the cross-validations have accuracies higher than 95%. These two analyses indicate that the identified subtypes are highly stable and independent of datasets.

**Table 1:** Cross-dataset comparisons of converged subtype signatures.

Datasets and subtypes		Northcott90				Kool62			**# s.g**.
			
	A	B	C	(# o.v.g.)	A	B	C	(# o.v.g.)	(# n.s.g., %)
Cho73									
A	136	0	1		207	2	6		345 (128, 37.1)
B	4	64	3		6	105	6		222 (56, 25.2)
C	0	1	37		3	5	66		110 (60, 54.5)
(# o.v.g.)				(237)				(378)	
Northcott90									
A					260	1	2		377 (97, 25.8)
B					1	110	2		175 (45, 25.7)
C					1	0	81		150 (77, 51.3)
(# o.v.g.)								(451)	

# s.g.	377	175		150	757	335	307		
(# n.s.g.)	(97)	(45)		(77)	(219)	(126)	(172)		
(%)					(28.9)	(37.6)	(56.0)		

The datasets have different numbers of probe-sets and were normalized separately, leading to different numbers of signature genes for different datasets of a subtype. o.v.g.=overlapping genes; s.g.=signature genes; n.s.g.=negative signature genes.

**Table 2:** Cross-dataset validations of the subtypes.

	Testing sets											
	
Training sets and self-trained subtypes	Cho73				Northcott90				Kool62			
			
	A	B	C	(accu.)	A	B	C	(accu.)	A	B	C	(accu.)
Cho73								(98.9%)				(96.7%)
A					7	0	0		9	0	0	
B					0	27	0		0	13	0	
C					0	1	55		0	2	38	
Northcott90				(97.3%)								(100%)
A	8	0	0						9	0	0	
B	0	17	2						0	15	0	
C	0	0	46						0	0	38	
Kool62				(100%)				(100%)				
A	8	0	0		7	0	0					
B	0	17	0		0	28	0					
C	0	0	48		0	0	55					

This table shows the numbers of cases in each testing set predicted to be of a subtype trained by the training set (first column). For example, the "2" in the testing set Kool62 indicates that 2 cases were self-trained to be of subtype B, but predicted to be of subtype C by the Cho73 dataset. accu.=accuracy.

**Figure 5:**
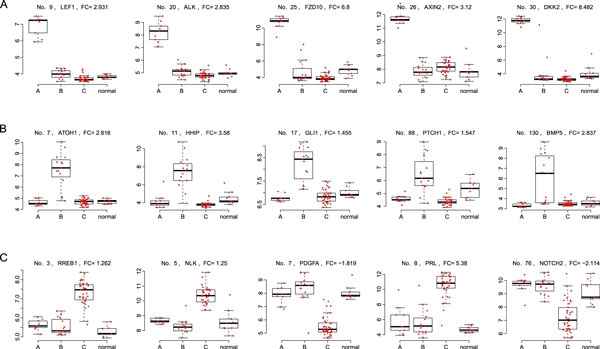
**Examples of key signature genes**. Examples of gene signatures for individual subtypes in Kool62. A, B, C: example key signature genes in Subtypes A, B and C, respectively. Within each plot, the three boxplots refer to expressions of a gene in the three subtypes, respectively. Normal refers to normal cases. Also shown are the original data with small amount of jittering on the horizontal axis. FC: fold change. Ranked by LIMMA adjusted *p*-values.

To characterize the converged subtypes, the gene signatures in the converged iterations were examined. Additional file 1 - Table S2 shows the pathway enrichment analysis of subtype signatures by GSEA [15]. From the table, Subtype A is significantly enriched with *Wnt*-pathway genes, while Subtype B is enriched with *Shh*-pathway genes. A comparison of the cases in Subtypes A and B with studies that published the data (Additional file 1 - Table S1) confirms that these two subtypes are the *Wnt*- and *Shh*-pathway associating pathways, respectively. For convenience of discussion, these two subtypes are referred to as the WNT and SHH subtypes, respectively. Examples of key signature genes of the subtypes (of the Kool62 dataset) are shown in Figure 5 (complete lists and plots in Additional file 2).

From Figure 5 and Additional file 2, it can be seen that the detected signature genes are specifically up-regulated or down-regulated in a particular subtype, and the inter-subtype variations in the non-specific subtypes are highly suppressed. Most importantly, these subtype-specific DEGs are not only numerically correlated but also functionally related. For example, the five genes in Figure 5A are all involved in the *Wnt*-pathway. *FZD10 *codes for the protein *Frizzled*, which is a membrane receptor for *Wnt*. Its abundant expressions may lead to the activation of *Wnt *pathway. *LEF1 *codes for a key transcription factor of the *Wnt *pathway and is responsible for the transcriptions of many *Wnt *target genes. Of interest, a number of genes that have antagonistic roles, such as the *DKK*s (Dickkopfs) and *AXIN2 *[16], are also up-regulated. These genes might be the consequences of negative feedback controls of the activated pathways. A similar phenomenon can be found in SHH (Figure 5B), where *GLI1/2 *are the key transcription factors and are specifically up-regulated, resulting in targets such as *HHIP *[17] and *PTCH1 *being highly expressed, too. *HHIP *in return regulates the *Shh*-pathway [18], while the protein *Patched *encoded by *PTCH1 *negatively regulates the *Shh*-pathway [19]. Indeed, inhibition of *PTCH1 *is among the recent attempts to find drug targets for cancers including medulloblastoma [20]. Overall, these signature genes confirm our hypothesis that they are the consequences of the subtype-specific cancer processes.

The third subtype (Subtype C) has the largest number of patients in all three datasets. Note that, this group was further divided into various numbers of subtypes, as shown in Additional file 1 - Table S1. Indeed, in the iterative process, our algorithm also detects that there can be four or five *core clusters *in the Kool62 datasets (iterations 1 to 3, Figure 4E). But gradually, core clusters containing too few signature genes were determined to be bad clusters and cases in them were re-assigned to their closest good clusters, before the algorithm converges. To further investigate the problem, we used our signature detection algorithm and the labels reported by the original studies to detect signature genes for the subtypes. As shown in Additional file 1 - Table S1, many subtypes claimed by previous studies contain extremely low numbers of signature genes, i.e., subtype-specific DEGs. For example, Group D in the Northcott90 dataset contains only 6 signature genes, and Subtype C in Kool62 contains only 5 signature genes. We then used cases of each of the non-*Wnt*/non-*Shh *(NWS) subtypes, with cases of the *Wnt *and *Shh *subtypes, and the normal cases to detect signature genes (using same technique as above, with FCT = 1.0) for each of the NWS subtypes claimed by the previous studies. It turns out that these NWS subtypes have high overlaps in signature genes (Table 3). For example, Group C and Group D in the Northcott90 dataset have 76 overlapping signature genes, which is more than 40% of the signature genes in both groups. Similarly, the NWS subtypes C, D and E in Kool62 have 46 overlapping signature genes, which is about 20~40% of the signatures of the corresponding subtypes. Although in the Cho73, the five NWS subtypes have only one overlapping signature gene, when only selective NWS subtypes are considered, substantial overlaps can be seen. For example, Subtypes 4 and 5 share 34 signature genes, which is more than 35% in both subtypes. This further confirms that signatures of these subtypes are highly overlapping, which in turn means that the NWS subtypes are functionally overlapped with each other. Given these analyses, it is reasonable to favor the convergence results above by referring to all NWS cases as one subtype, namely, the NWS subtype.

**Table 3:** The subtype signatures of the non-Wnt/non-Shh subtypes by previous studies.

Datasets and original labels	Subtype WNT (# s.g.)	Subtype SHH (# s.g.)	NWS subtypes (# s.g.)
Cho73			
1	313	243	34 *
2 5 7	342	237	45 *, ‡, ¶
4	366	222	75 †, ‡, ¶
5	339	244	93 †, ¶
7	330	245	6
(Total overlaps)	(203)	(127)	(1)
(Selective overlaps) Northcott90			(* 7, † 34, ‡ 25, ¶12)
Group C	330	179	170
Group D	396	181	186
(Total overlaps)	(253)	(112)	(76)
Kool62			
Subtype C	773	287	284 *,‡
Subtype D	738	299	237 *, †, ‡
Subtype E	657	268	105 †
(Total overlaps)	(498)	(167)	(46)
(Selective overlaps)			(* 158, † 66, ‡ 53)

Each row represents the numbers of signature genes for the WNT, SHH and one of the NWS subtypes (the label of which is shown on the first column of each row), respectively. The total overlaps refer to the intersection of all signatures of a group in each dataset, e.g., |*S*_Subtype C _∩ *S*_Subtype D _∩ *S*_Subtype E_| = 46. Selective overlaps refer to the intersection of the signatures with the same symbol. The difference between this table and Table S1 is that in the latter, all subtypes are trained together, while in the former, only one of the reported NWS subtypes is trained at a time with the WNT, SHH and normal cases for detection of signatures. s.g.=signature genes.

Note that, the NWS seems to be more negatively regulated than other subtypes, as it contains more negative signature genes than positive ones (Table 1, 51.3~56.0%), while the WNT and SHH subtypes have far fewer percentages of negative signature genes (25.2~37.6%). Indeed, not only was the numbers of NWS subtypes not clear, the functional characterization of them has not been as successful as the WNT and SHH subtypes either, where dominant pathways exist evidently. The signature genes by this study may give a hint on the underlying process of NWS. As shown in Figure 5C, a *Wnt*-pathway inhibitor *NLK *[21] is specifically up-regulated. Also negatively regulated are the *Notch *pathway receptor *NOTCH2 *and the growth factor *PDGFA*. The suppression of these genes indicates that as compared with WNT and SHH, where the key pathways are activated, NWS may have an opposite mechanism, namely, some key pathways are inactivated. In all, our study confirms the hypothesis that gene signatures define the subtype boundaries and unveil subtype mechanisms. Finding the correct boundaries depends on sufficient signature genes contained in the subset of genes used for clustering, and detecting the signatures depends on the correct subtype boundaries. This justifies the above iterative subtyping algorithm.

### Subtype-specific copy number landscapes and candidate driver pre-selection

**Figure 6:**
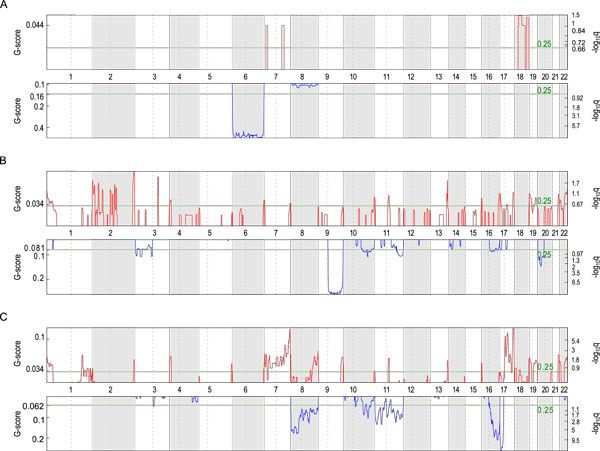
**Subtype-specific GISTIC landscapes of the Cho73 dataset**. The three sub-plots correspond to GISTIC copy number landscapes of the three subtypes of the Cho73 dataset, respectively. A, Subtype A (WNT); B, Subtype B (SHH); C, Subtype C (NWS). In each subplot, the upper panel (red) corresponds to the recurrent copy number gains, while the lower panel (blue) corresponds to the recurrent copy losses. The numbers to left of each panel refer to the G-scores. The numbers to the right of each panel refer to the − log_10 _*q*-values. The green lines refer to the *q*-value threshold of 0.25 (or − log_10 _*q *= 0.602). The numbers (1 to 22) in-between the panels refer to the autosomes.

Copy number profiles of the two datasets with publicly available SNP arrays, Cho73 and Northcott90, were inferred as described in Methods. The copy number landscapes are show in Additional file 1 - Figure S3. As shown in Figure 2, profiles of each subtype are further processed with GISTIC (http://via genepattern.broadinstitute.org) for detection of recurrent CNAs within each subtype. Copy number profiles with log_2_(ratio)-1 greater than 0.35 are considered to be copy number gains, while those below -0.35 are considered to be copy number losses. The subtype-specific GISTIC landscapes of the Cho73 dataset is shown in Figure 6, and those for the Northcott90 dataset is shown in Additional file 1 - Figure S4. The *q*-value threshold of 0.01 is used to determine recurrent CNA regions. Genes (UCSC human reference assembly hg18) within these regions are considered to be CNA-affected genes. The numbers of CNA-affected genes are tabulated in Additional file 1 - Table S3.

As Figure S4 and Table S3 show, the copy number landscapes and GISTIC landscapes demonstrate extremely strong subtype-specificity. For example, Subtype A is dominant with Chr6 deletions, and Subtype B is characterized with Chr9 deletions. Subtype C is more complex, CNAs are observed in Chr7-8, Chr11, Chr16-16, etc. These patterns are also highly dataset-independent. The overlapping candidates in Table S3 were taken to be the pre-selected CNA candidates.

### Testing the driver identification algorithm with synthetic data

To test the driver identification algorithm, we generated a synthetic gene expression dataset with 10,000 genes and 100 cases. Entries of the dataset were initialized with identically and independently distributed standard Gaussian noises. The first 100 genes are assumed to be signature genes and the next 200 are assumed to be candidate genes, while the remaining 9,700 genes are assumed to be non-signature and non-candidate (NSNC) genes. Each of the candidate genes *i *is given a weighting of *w_i_*. We added synthetic inter-dependencies to the signature and candidate genes by updating each signature gene with ${\tilde{x}}_{j}=\underset{i}{\sum }w_{i}c_{ij}x_{i}+x_{j}$, where *x_i _*and *x_j _*are the initialized gene expressions of the *i*-th candidate and *j*-th signature genes, respectively; and *c_ij _*is a random number (~ *U*(0, 1)) indicating the regulating potential of candidate *i *on signature *j*. We tested the algorithm with four types of *w_i_*, namely, *w_i _*~ *U*[−1, 0], *w_i _*~ *U*[0, 1], *w_i _*= 0 and *w_i _*~ *U*[−1, 1]. As additional file 1 - Figure S5 shows, when *w_i _*are initialized with non-positive random numbers, the estimated driver potentials ${ŵ}^{\prime }s$ are significantly lower than those of the NSNC genes. Similarly, when *w_i _*are initialized with non-negative random numbers, ${ŵ}^{\prime }s$ are significantly higher than those of the NSNC genes. When $w_{i}=0,{ŵ}^{\prime }s$ have no significant differences in the signature or NSNC genes. And when both positive and negative weightings are used, although no significant difference is observed in the means, the tails of the two distributions are very different. Particularly, the distribution of ${ŵ}^{\prime }s$ for the signature genes has longer tails on both sides.

This study indicates that the technique is able to identify the potential drivers by using the NSNC genes as the null model, if the inter-dependencies between the drivers and the signature still exist at the time of measurement.

### Subtype related driver candidates revealed by driver identification

The above technique was applied to the two datasets Cho73 and Northcott90, where both expression and CNA profiles are available. Additional file 2 - Figure S6 shows the candidate and null distributions for the subtypes in both datasets. The curves demonstrate similar patterns in different datasets for the same subtype. For example, in Subtype A (WNT), CNA candidates of both datasets tend to have longer negative tails, while in Subtype B (SHH), CNA candidates of both datasets tend to have longer positive tails. In Subtype C (NWS), CNA candidates of both datasets have slightly longer tails than the null distributions. Further, the significant candidates are found to be highly reproducible (30.0%~44%, Additional file 2 - Table S4).

Table 4 shows the reproducible top-ranked (*p*-value *<*0.01) candidate drivers in each subtype. Of note, all three subtypes contain some genes that seem to suggest the underlying pathways that characterize the subtypes. For example, Subtype A candidate drivers include *MAP3K7*, which has inhibiting roles in the *Wnt-*pathway [22]. Its deletion might cause the difficulty to inactivate *Wnt*-pathway and results in a persistently activating *Wnt*-pathway. Further, *TULP4 *is recently identified as a candidate suppressor gene in the *Wnt*associating subtype [23]. The top candidate driver in Subtype B, *PTCH1*, is a key gene in the *Shh*-pathway. Its deletion may result in the inability to inhibit Smoothened, and activate *Shh *permanently. Indeed, mutations in *PTCH1 *have been one of the top targets in recent literature attempting to find predisposition loci for MB [24]. Perhaps of most interest is the *Wnt*-associating genes, such as *FZD1, PPP3CB *and *NLK*, that are found in Subtype C. As compared with *Wnt*- and *Shh*-subtypes, the gene signature of this subgroup of MB does not seem to be significantly enriched with any canonical pathways. The candidate drivers that significantly correlate with the signature genes here seem to suggest that some *Wnt*-pathway activities are involved in Subtype C; although the exact roles of *Wnt *have yet to be clarified, as both *NLK *and *FZD1 *are amplified but the former has inhibiting while the latter has promoting roles.

**Table 4:** Candidate CNA drivers within each subtype.

Subtypes	Significant candidate drivers
Subtype A	
(WNT)	*NRN1, SOX4, NUP153, FAM8A1, C6orf62, MRS2, BTN2A1, ZNF193, ZNF187, BAT3, C6orf134, ZNF318, UBR2, KIAA0240, CDC5L, MUT, ICK, FBXO9, PTP4A1, SMAP1, SLC35A1, RNGTT, SNAP91, **MAP3K7**, IBTK, SFRS18, ZNF292, PHIP, DOPEY1, SYNCRIP, CASP8AP2, MARCKS, HDAC2, ASF1A, FOXO3, HSF2, CDC2L6, TSPYL4, MED23, TRMT11, FAM184A, CCDC28A, HECA, AHI1, RBM16, **TULP4**, TCP1, TBP *
Subtype B	
(SHH)	***PTCH1**, SLC35D2, ANGPTL2, TRPM3, UGCG, ALAD, HDHD3, STOM, ASTN2, RABGAP1, GOLGA1, AK1, SPTAN1, DNM1, BAT2L, NPLOC4*
Subtype C	
(NWS)	*PDGFA, SRI, PCOLCE, EPHB4, TRIP6, SYPL1, MDFIC, MAP2K6, GPRC5C, NAV2, AHNAK, GNG3, GPR56, MAF, PMP22, IQCE, CHN2, POM121, GTF2IRD1, PCLO, **FZD1**, AKAP9, PSMD11, **NLK**, RHOT1, ACLY, MPP3, CBX1, MMD, HEATR6, MED13, KCNJ2, TEX2, **PPP3CB**, DLG5, CHD3 *

## Conclusions

In this work, a two-stage algorithmic framework was developed to perform gene-signature based cancer subtyping and to identify subtype-specific CNA drivers. The algorithm was applied to datasets of medulloblastoma, producing dataset-independent subtyping results. The driver identification results were found to be enriched with cancer-driving pathways. This study is novel in the following three aspects.

First, the signature-based subtyping technique ensures that as many subtypes are uncovered as possible while each of them is required to have a sufficient number of signature genes. This emphasis on subtype-specificity of signature genes allows for functional interpretation of the cancer process or pathways underlying each subtype. This procedure is not only a technique, but also a concept of viewing cancer subtypes.

Second, a comparison of our results with previous results indicates that the non- *Wnt*/non-*Shh *subtypes have high overlaps in their gene signatures, resulting in their merger into one single subtype (the NWS subtype) by our algorithm. Although distinct clusters can be observed in the NWS subtype, the fact that their signature genes are highly overlapping suggests that these distinct clusters may be due to non-functional causes, such as different copy number profiles, different cells-of-origin, etc. Therefore, it is not appropriate to classify them as expression subtypes, and a future direction would be to extract copy number subtypes that explain these distinct clusters under NWS.

Third, the proposed driver identification method relates the within-subtype recurrent genetic events to the subtype signature based on the strengths of their inter-dependencies. The idea to conduct this in a subtype-specific manner echoes the ultimate purpose of subtyping: towards more refined understandings of the disease. This idea can be further explored. For example, as depicted in Figure 1, most of the CNAs may be passengers, and some CNA drivers may have hit-and-run properties, i.e., inter-dependencies do not hold by the time of measurements (as a result of dynamic processes). This makes them non-observable by the current method. Further, other types of candidate drivers, such point mutations and DNA-methylation, may be screened as well.

## Methods

### Datasets and preprocessing

Three publicly available medulloblastoma datasets were used.

The first dataset consists of 73 primary MB samples and 11 normal cerebellum controls by Cho *et al*. [8] (GEO: GSE19399). All cases in this dataset except the controls have matched SNP arrays.

The second dataset consists of 90 primary MB cases in both expression (exon) arrays and SNP arrays by Northcott *et al*. [25] (GEO: GSE21166 and GSE14437). Four cerebellar samples from (GEO: GSE13344) were used as controls for this dataset.

The third dataset consists of 62 primary MB samples from Kool *et al*. [3] (GEO: GSE10327). No publicly available CNA measurements are available for this dataset. Another nine cases of expression profiles of cerebellum were obtained from the controls of a schizophrenia study (GEO: GSE4036). These nine cases are in the same platform as the Kool dataset and were used as the controls for current study.

For convenience, the three datasets are referred to as Cho73, Northcott90 and Kool62, respectively.

Expression arrays of all cases in all three datasets were processed with the RMA algorithm [26] or its variants. Since the three datasets are in different platforms, some genes are measured in one but not the other datasets. To handle this, only the set of genes common to both datasets are used, and their probesets are normalized, when performing cross-dataset validations. Specifically, given a gene with means *m*_1 _and *m*_2_, and standard deviations *s*_1 _and *s*_2_, in two cross validating datasets, respectively, the normalized expression of this gene shall have a mean of (*m*_1 _+ *m*_2_)/2 and a standard deviation of $\sqrt{s_{1}^{2}/n_{1}+s_{2}^{2}/n_{2}}$, where *n*_1 _and *n*_2 _are the numbers of cases in the two datasets.

An in-house developed software (not published) running on a cluster of computers was used to process the large numbers of SNP arrays. HapMap [27] data were used as the unmatched references for inferring CNA profiles.

### Gene signature detection

A subtype signature is defined to be the set of genes whose expressions are dys-regulated specifically in a subtype. To ensure this specificity, on top of statistical significance, we impose a fold change requirement.

First, given expressions {*y_j_*|*j *= 1, …, *M*} of a gene and the corresponding trained subtype labels {*L_j_*|*L_j _*= 1, …, *K*}, the purpose is to test if this gene is differentially expressed in subtype *k*, as compared with all other cases, regardless of their labels. This is a two-class feature selection problem, and can be efficiently handled by LIMMA [28], with a correction of the multiple comparisons by the BH method [29].

Second, define the subtype-specific deviation:

(3) $${\text{Δ}}_{k}=\text{min}\left(\left|\mu_{k}-\underset{\ell \neq k}{\text{min}}\left(\mu_{\ell }\right)|,|\mu_{k}-\underset{\ell \neq k}{\text{max}}\left(\mu_{\ell }\right)\right|\right)$$

and the non-specific deviation:

(4) $${\text{Δ}}_{k}^{\prime }=\;|\underset{\ell \neq k}{\text{max}}\left(\mu_{\ell }\right)-\underset{\ell \neq k}{\text{min}}\left(\mu_{\ell }\right)|$$

Here, *µ_k _*and $\mu_{\ell }$ are the expected means of the corresponding subtypes. A gene is said to be specific to subtype *k *if $\mu_{k}>\mu_{\ell }$ (or $\mu_{k}<\mu_{\ell }$) for all ℓ≠k, and the subtype-specific fold change, $\text{FC}\left(k\right)={\text{Δ}}_{k}-\;{\text{Δ}}_{k}^{\prime }$ is greater than a certain threshold (illustrated in Additional file 1 - Figure S2). The set of genes that are determined to be both significantly expressed by LIMMA and specific to *k *shall be the subtype signature of *k*, denoted as *S_k_*.

## List of abbreviations used

CCA: Canonical Correlation Analysis; CNA: Copy Number Aberration; DEG: Differentially-expressed gene; FCT: Fold-change threshold; GEO: Gene Expression Omnibus; GISTIC: Genomic Identification of Significant Targets in Cancer; GSEA: Gene Set Enrichment Analysis; LIMMA: Linear Models for Microarray Data; MB: Medulloblastoma; NSNC: non-signature and non-candidate; NWS: non-*Wnt*/non-*Shh *subtype; PCA: Principal Component Analysis; RMA: Robust Mult-chip Average; SCCA: Sparse Canonical Correlation Analysis; SHH: *Shh*-pathway associating subtype; SNP Single-nucleotide Polymorphism; SVD: Singular Value Decomposition; UCSC: University of California, Santa Cruz; WNT: *Wnt*-pathway associating subtype.

## Competing interests

The authors declare that they have no competing interests.

## Authors' contributions

STCW and CCL introduced the problem. PKC proposed the algorithms, wrote the programs and analyzed the data. YBF and YSH refined the models. STCW, CCL and TKM interpreted the results. The draft was written by PKC. All authors read and approved the final manuscript. Parts of the work were conducted while PKC was on leave from HKU to TMHRI.

## Supplementary Material

Additional file 1 **Supplementary figures and tables**. Click here for file

Additional file 2 **The converged signatures for the subtypes of the three datasets**. Click here for file

## Declarations

Publication of this article was supported by the TT and WF Chao Foundation.

This article has been published as part of *BMC Bioinformatics *Volume 14 Supplement 18, 2013: Selected articles from the Second IEEE International Conference on Computational Advances in Bio and Medical Sciences (ICCABS 2012): Bioinformatics. The full contents of the supplement are available online at http://www.biomedcentral.com/bmcbioinformatics/supplements/14/S18.
