# Supplementary material for: A gene signature based method for identifying subtypes and subtype-specific drivers in cancer with an application to medulloblastoma
Source: BMC Bioinformatics. 2013 Nov 5;14(Suppl 18):S1. doi: 10.1186/1471-2105-14-S18-S1 (PMC3820164; doi:10.1186/1471-2105-14-S18-S1)
Supplement: Additional file 1 — Supplementary figures and tables. [file 1471-2105-14-S18-S1-S1.pdf]

1 Figures

Figure S1 - One step subtyping approach on the three medulloblastoma datasets.

The following three subplots correspond to application of the one-step subtyping algorithm to the three cancer datasets, Cho73 (A), Northcott90 (B) and Kool62 (C).

A

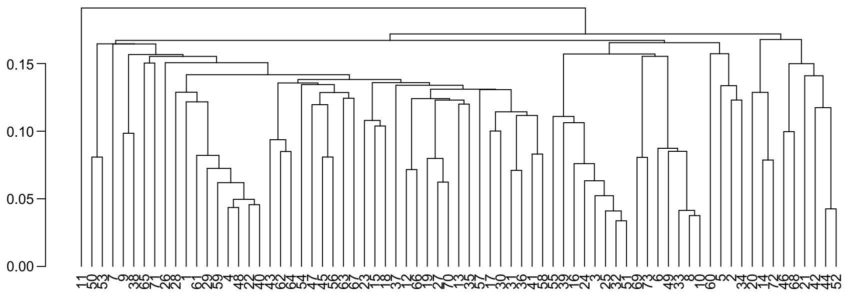

B

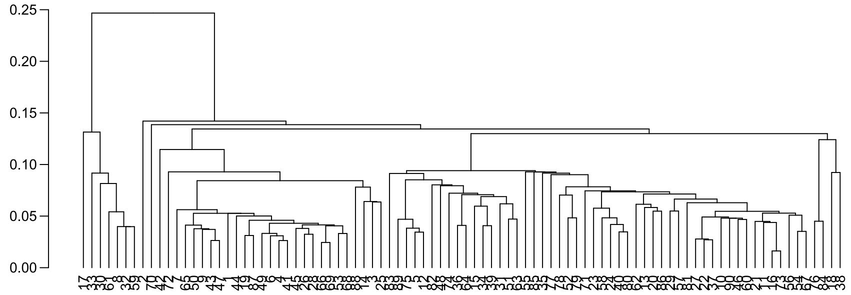

C

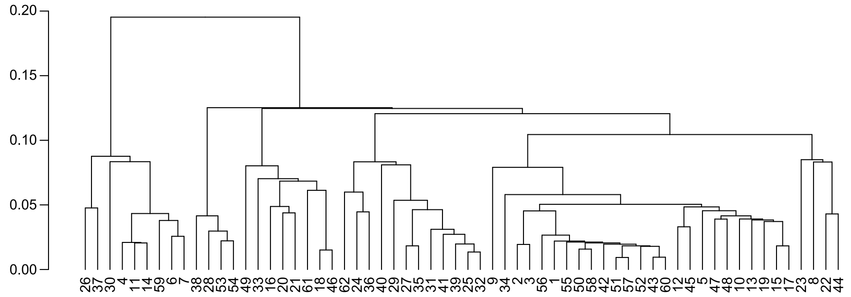

Figure S2 - illustrating the definition of subtype-specific fold change for signature genes.
This figure illustrates the specificity fold change, defined as $\Delta_k - \Delta'_k$, of a up-regulated signature gene. A down-regulated signature gene follows the same fashion.

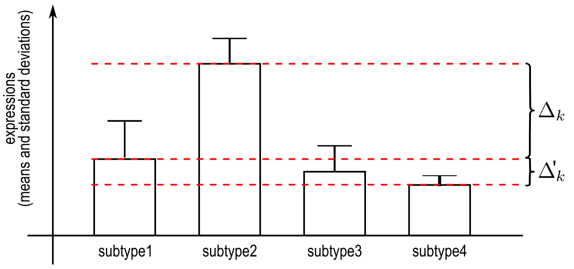

Figure S3 - Copy number landscape within each trained subtype.
This figure shows the copy number landscape of each subtype of the Cho73 dataset (A) and the Northcott90 (B) dataset. In each subplot, the horizontal axis represents the chromosomes, and the vertical axis the cases. Since medulloblastoma has not been shown to be gender-related and gender information is not available for some of the datasets, the X chromosome is not shown. The case numbers are matched to Figure 4 of the paper. Red colors indicate copy number gains (i.e., CN>2), while blue colors indicate copy number losses (i.e., CN< 2). The two red horizontal lines in both (A) and (B) refer to the subtype boundaries.

A

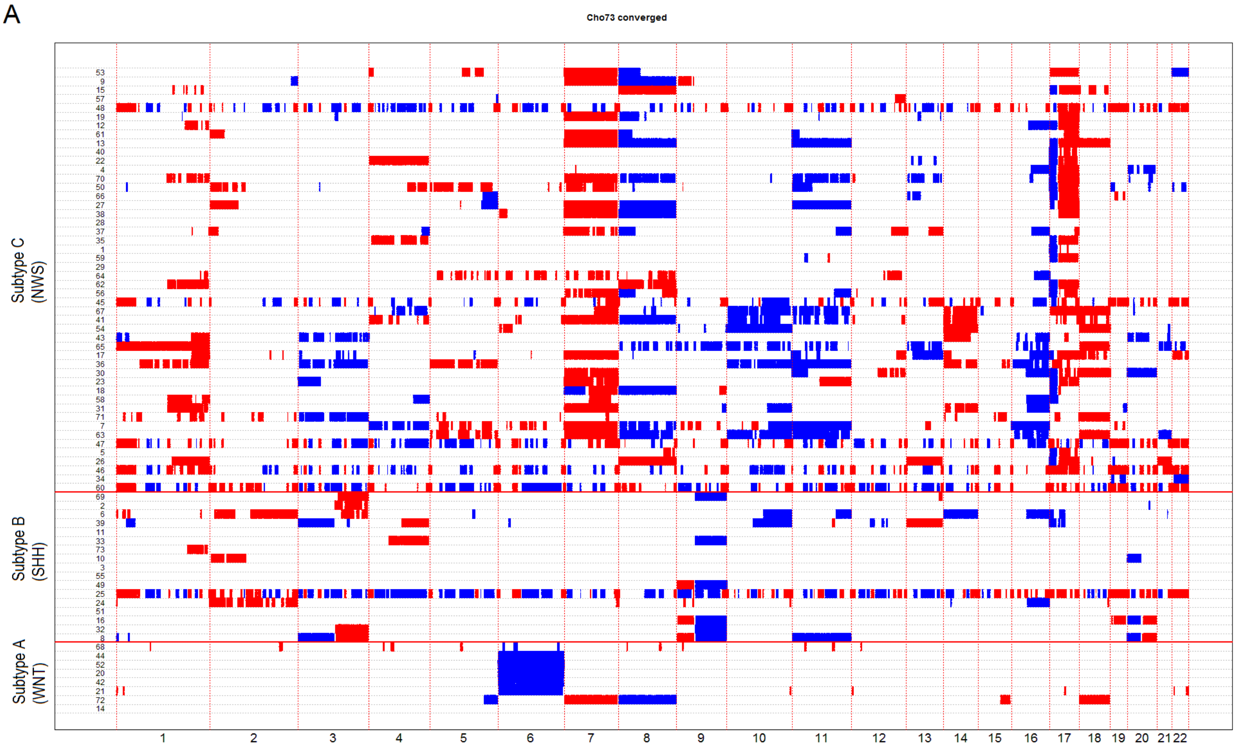

B

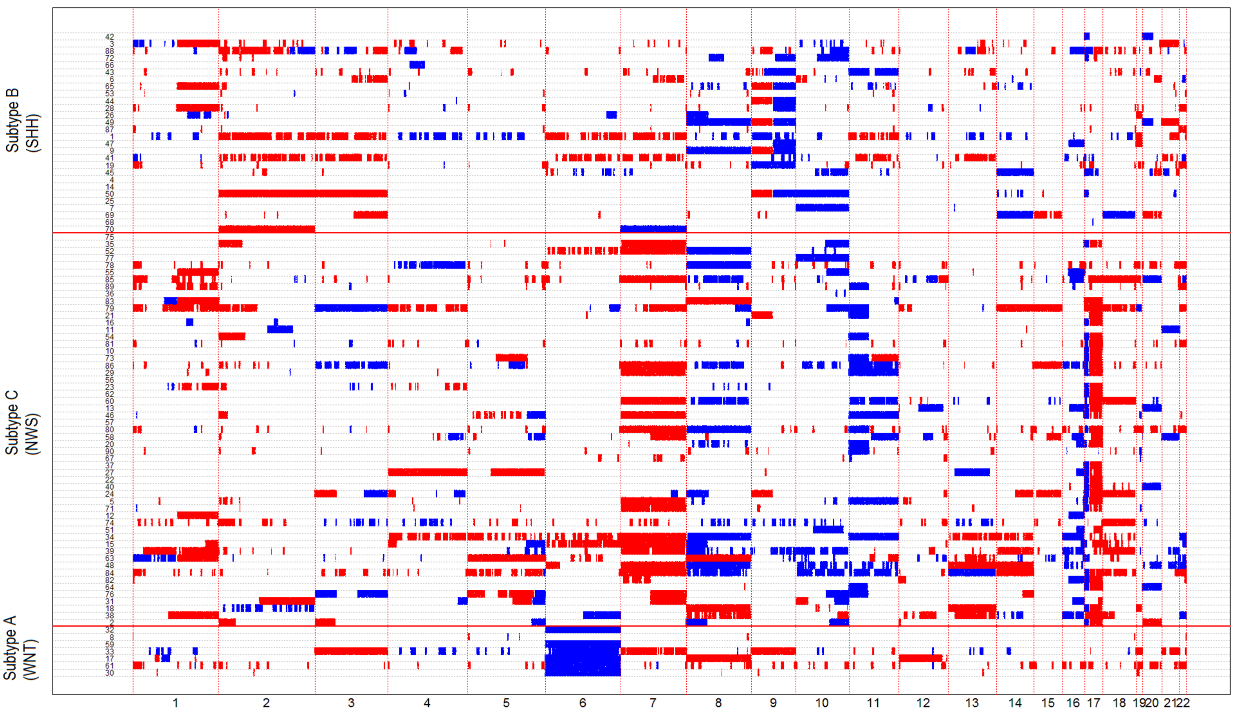
Northcott90 converged
Subtype B
(SHH)
Subtype C
(NWS)
Subtype A
(WNT)
1 2 3 4 5 6 7 8 9 10 11 12 13 14 15 16 17 18 19 20 21 22

Figure S4 - GISTIC landscapes for the subtypes of the Northcott90 dataset.

The following sub-plots correspond to GISTIC copy number landscapes of the three subtypes of the Northcott90 dataset. A, Subtype A (WNT); B, Subtype B (SHH); C, Subtype C. In each sub-plot, the upper panel (red) corresponds to recurrent copy number gains, while the lower panel (blue) corresponds to recurrent copy losses. The numbers to the left of each panel refer to the G-scores. The numbers to the right of each panel refer to the $-\log_{10} q$-values. The green lines refer to the q-value threshold of 0.25 (or $-\log_{10} q = 0.602$). The numbers (1 to 22) in between the panels refer to the somatic chromosomes. As in Figure S2, MB has not been found to be gender-linked and self-matched arrays are not available, so the X and Y chromosomes are not shown.

A

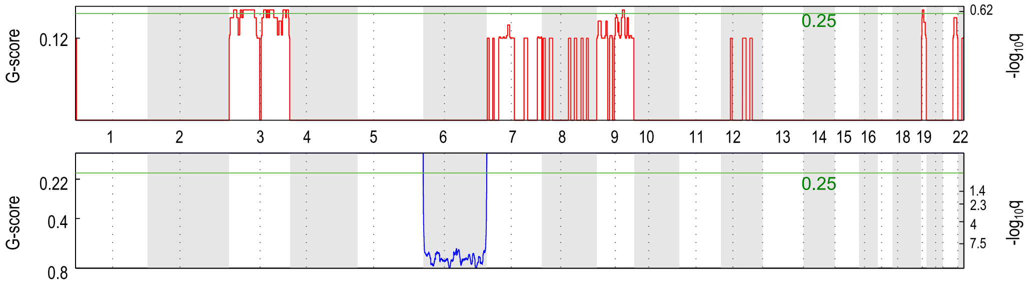

B

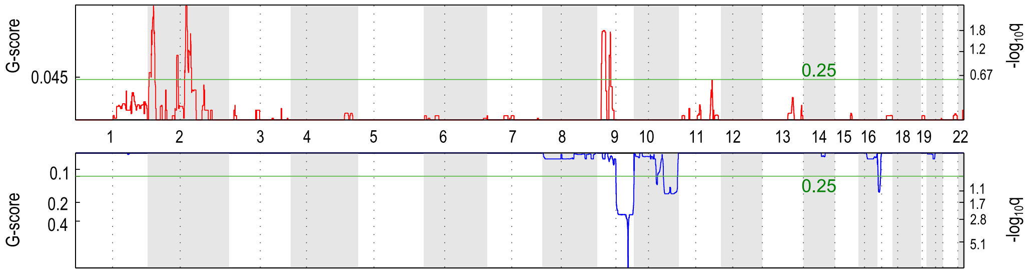

C

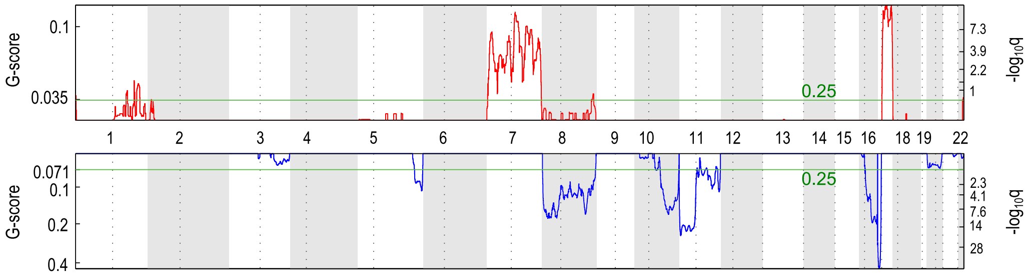

Figure S5 - Driver identification algorithm on synthetic data.

The sub-plots A-D show the empirical distributions of the estimated driver potentials for the four types of real underlying regulating potentials, (A) $w_i \sim [-1, 0]$, (B) $w_i \sim [0, 1]$, (C) $w_i = 0$ and (D) $w_i \sim [-1, 1]$, respectively. In A-D, red curves correspond to distributions of non-signature/non-candidate (NSNC) genes; and black curves correspond to distributions of signature genes. Densities are esitmated by kernel methods (parameters at the bottom of each sub-plots) The black and red vertical lines indicate the means of the corresponding distributions. The sub-plots E-H show boxplots of the estimated potentials of A-E, respectively.

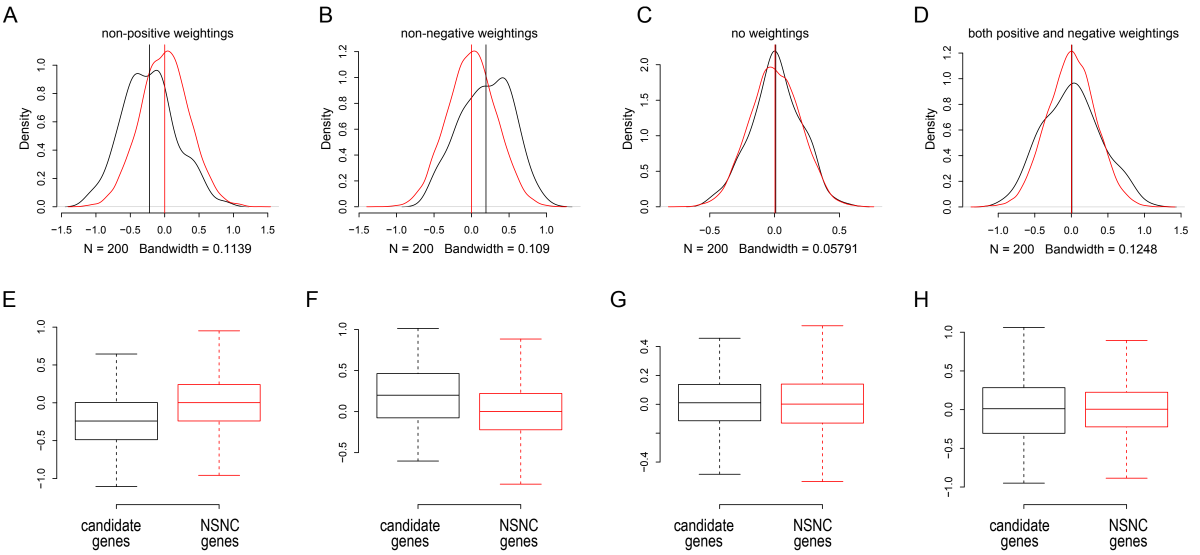

Figure S6 - Driver identification algorithm on real data.

The sub-plots A-C show the empirical distributions (esitmated using kernel methods as in Figure S5) of the candidate CNAs (red curves) and NSNC genes (black curves) for the three subtypes A-C of Cho73 dataset, respectively. The sub-plots D-F show the same distributions to the subtypes A-C of Northcott90, respectively. The two vertical dashed lines in each sub-plot refer to the lower and upper thresholds for 0.01 cutoff.

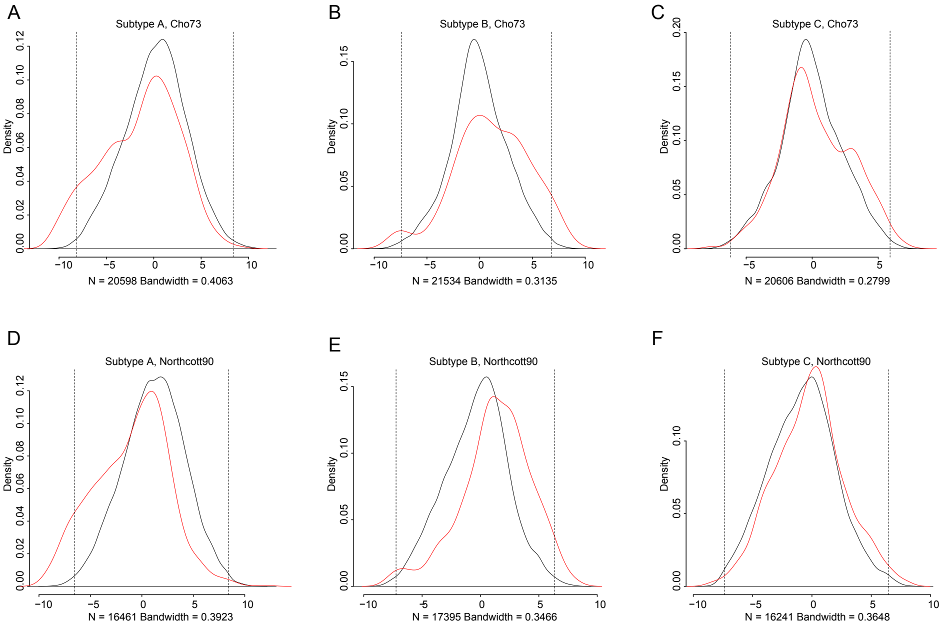

2 Tables

Table S1 - A comparison of trained subtype labels by our algorithm and by the original studies that produced the data.

Datasets and original labels	Trained class labels by our algorithm (# of cases)			
	Subtype A	Subtype B	Subtype C	# signature genes
Cho73				
1	0	0	10	3
2	0	0	10	2
3 (Shh)	0	17	0	25
4	0	0	14	0
5	0	0	12	20
6 (Wnt)	8	0	0	53
7	0	0	2	125
Northcott90				
Group A (WNT)	7	0	0	119
Group B (SHH)	0	28	0	27
Group C	0	0	16	30
Group D	0	0	39	6
Kool62				
Subtype A (Wnt)	9	0	0	248
Subtype B (Shh)	0	13	0	69
Subtype C	0	0	16	5
Subtype D	0	0	11	15
Subtype E	0	0	11	20

Note: the first column represents the subtype labels by the original studies. Numbers in the second to the fourth column represent the numbers of cases in each subtype. Numbers in the fifth column represent the numbers of signature genes detected on the original subtypes using our signature detection algorithm (with FC=1.0).

Table S2 - Pathway analysis of subtype signatures.

Subtypes and top pathways [# pathway genes]	Description	# Genes in overlap	*p*-values
Subtype A			
WNT_SIGNALING_PATHWAY [151]	Wnt signaling pathway	9	**0.0382**
THYROID_CANCER [29]	Thyroid cancer	3	0.0556
BASAL_CELL_CARCINOMA [55]	Basal cell carcinoma	4	0.0833
ENDOMETRIAL_CANCER [52]	Endometrial cancer	3	0.207
ECM_RECEPTOR_INTERACTION [84]	ECM-receptor interaction	4	0.248
ACUTE_MYELOID_LEUKEMIA [60]	Acute myeloid leukemia	3	0.271
COLORECTAL_CANCER [62]	Colorectal cancer	3	0.288
Subtype B			
HEDGEHOG_SIGNALING _PATHWAY [56]	Hedgehog signaling pathway	5	**0.00205**
BASAL_CELL_CARCINOMA [55]	Basal cell carcinoma	3	0.0601
NEUROTROPHIN_SIGNALING _PATHWAY [126]	Neurotrophin signaling pathway	4	0.15
ECM_RECEPTOR_INTERACTION [84]	ECM-receptor interaction	3	0.157
SMALL_CELL_LUNG_CANCER [84]	Small cell lung cancer	3	0.157
ABC_TRANSPORTERS [44]	ABC transporters	2	0.161
PATHWAYS_IN_CANCER [328]	Pathways in cancer	8	0.165
Subtype C			
BASAL_CELL_CARCINOMA [55]	Basal cell carcinoma	2	0.0707
WNT_SIGNALING_PATHWAY [151]	Wnt signaling pathway	3	0.118
MELANOGENESIS [102]	Melanogenesis	2	0.195
NOTCH_SIGNALING_PATHWAY [47]	Notch signaling pathway	4	0.295
CYTOKINE_CYTOKINE_RECEPTOR _ INTERAERACTION [267]	Cytokine-cytokine receptor interaction	2	0.636
MAPK_SIGNALING_PATHWAY [267]	MAPK signaling pathway	2	0.636
NEUROACTIVE_LIGAND_RECEPTOR _ INTERTERACTION [272]	Neuroactive ligand -receptor interaction	2	0.647

Notes: numbers in bold indicate statistically significant (p <0.05). The reproducible signature genes were used, i.e., the intersection of all signatures of a subtype in the three datasets.

Table S3 - Numbers of pre-selected driver candidates within each subtype.

Chromosomes		Subtype A			Subtype B			Subtype C		
		Cho73	NC90	Overlaps	Cho73	NC90	Overlaps	Cho73	NC90	Overlaps
Chr1	gains	0	0	0	0	0	0	100	0	0
	losses	0	0	0	0	0	0	0	0	0
Chr2	gains	0	0	0	30	93	0	0	6	0
	losses	0	0	0	0	0	0	0	0	0
Chr3	gains	0	0	0	11	0	0	0	0	0
	losses	0	0	0	0	0	0	0	0	0
Chr6	gains	0	0	0	0	0	0	0	0	0
	losses	1118	1033	1015	0	0	0	0	0	0
Chr7	gains	0	0	0	0	0	0	551	732	358
	losses	0	0	0	0	0	0	0	0	0
Chr8	gains	0	0	0	0	0	0	21	0	0
	losses	0	0	0	0	0	0	327	428	244
Chr9	gains	0	0	0	0	16	0	46	0	0
	losses	0	0	0	568	367	357	0	0	0
Chr10	gains	0	0	0	0	0	0	0	0	0
	losses	0	0	0	0	0	0	2	329	2
Chr11	gains	0	0	0	0	0	0	0	0	0
	losses	0	0	0	0	0	0	338	287	134
Chr16	gains	0	0	0	0	0	0	0	0	0
	losses	0	0	0	0	0	0	310	255	213
Chr17	gains	0	0	0	0	0	0	702	650	569
	losses	0	0	0	0	0	0	258	80	73
Chr18	gains	76	0	0	0	0	0	0	0	0
	losses	0	0	0	0	0	0	0	0	0
Chr19	gains	0	0	0	0	0	0	0	0	0
	losses	0	0	0	0	0	0	0	0	0
Chr21	gains	0	0	0	0	0	0	79	0	0
	losses	0	0	0	0	0	0	0	0	0
Chr22	gains	0	0	0	0	0	0	37	0	0
	losses	0	0	0	0	0	0	0	0	0
Total		1194	1033	1015	627	476	357	2795	2761	1593

Note: NC90=Northcott90. Chr4-5, Chr12-15 and Chr20 are not shown as they contain no CNA-affected genes. Overlaps refer to the numbers of overlapping (pre-selected) candidates in both datasets.

Table S4 - Numbers of significant candidates.

Datasets	Subtype A (WNT)	Subtype B (SHH)	Subtype C (NWS)
Cho73	154 (31.2%)	50 (32%)	120 (30%)
Northcott90	139 (34.5%)	37 (43.2%)	81 (44.4%)
(overlaps)	48	16	36

Note: significant candidates = empirical p-values ≤ 0.01. Numbers in brackets indicate the overlaps as percentages of the significant candidates.
