# Supplementary material for: A gene signature based method for identifying subtypes and subtype-specific drivers in cancer with an application to medulloblastoma
Source: BMC Bioinformatics. 2013 Nov 5;14(Suppl 18):S1. doi: 10.1186/1471-2105-14-S18-S1 (PMC3820164; doi:10.1186/1471-2105-14-S18-S1)
Supplement: Additional file 2 — The converged signatures for the subtypes of the three datasets. [file 1471-2105-14-S18-S1-S2.zip › convergeMap-Northcott90.pdf]

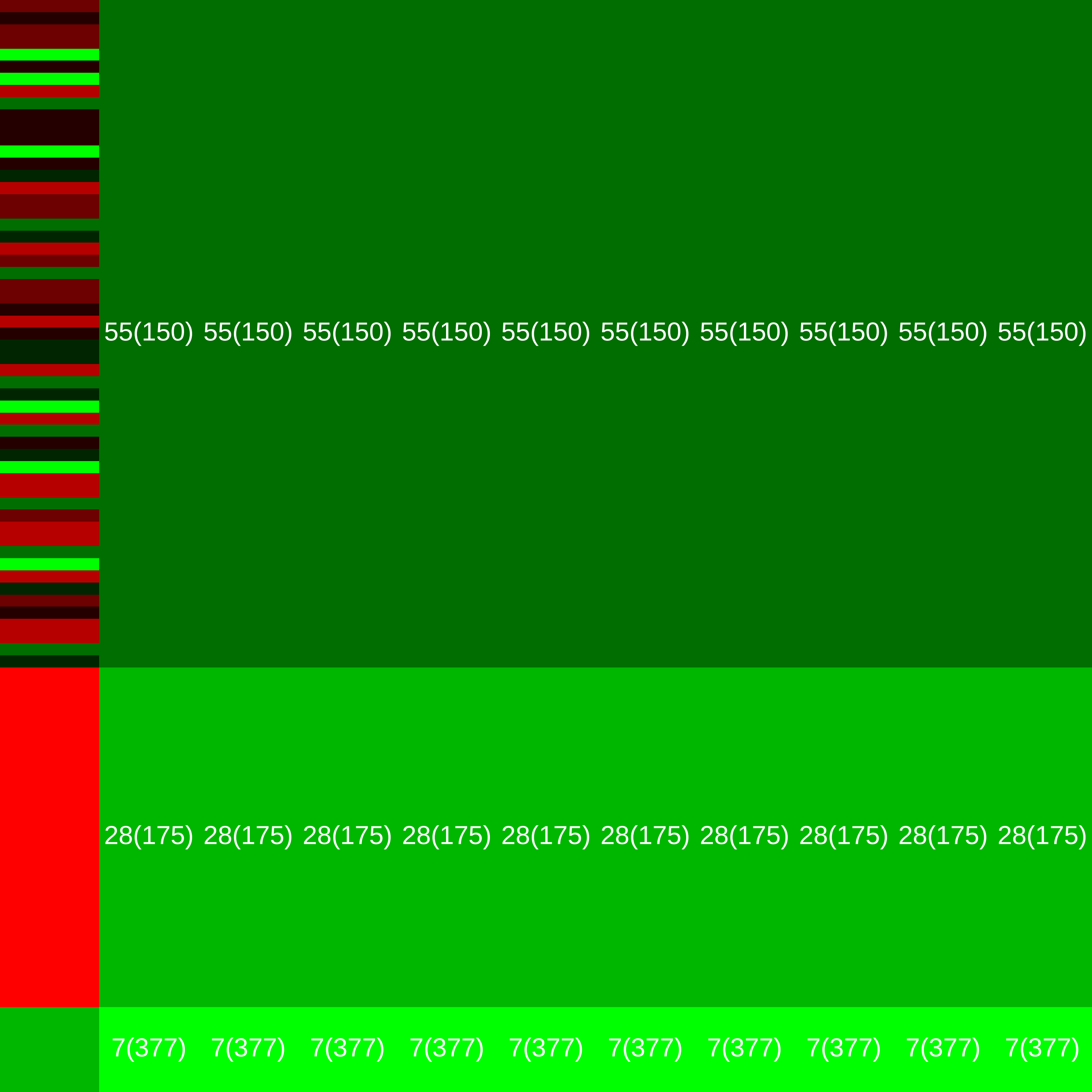
55(150) 55(150) 55(150) 55(150) 55(150) 55(150) 55(150) 55(150) 55(150) 55(150)
28(175) 28(175) 28(175) 28(175) 28(175) 28(175) 28(175) 28(175) 28(175) 28(175)
7(377) 7(377) 7(377) 7(377) 7(377) 7(377) 7(377) 7(377) 7(377) 7(377)
