# Supplementary figures and images for: A gene signature based method for identifying subtypes and subtype-specific drivers in cancer with an application to medulloblastoma
Source: BMC Bioinformatics. 2013 Nov 5;14(Suppl 18):S1. doi: 10.1186/1471-2105-14-S18-S1 (PMC3820164; doi:10.1186/1471-2105-14-S18-S1)

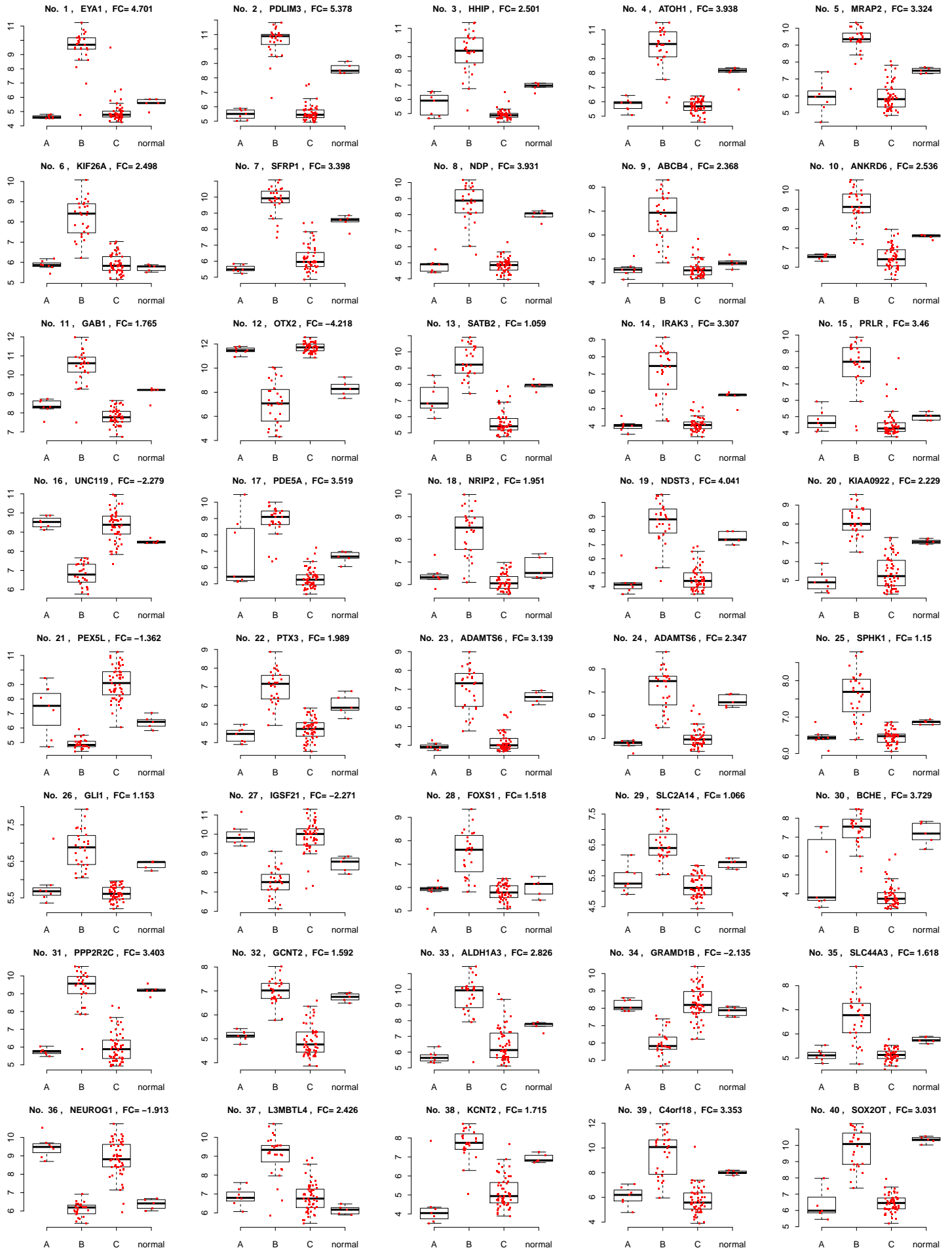

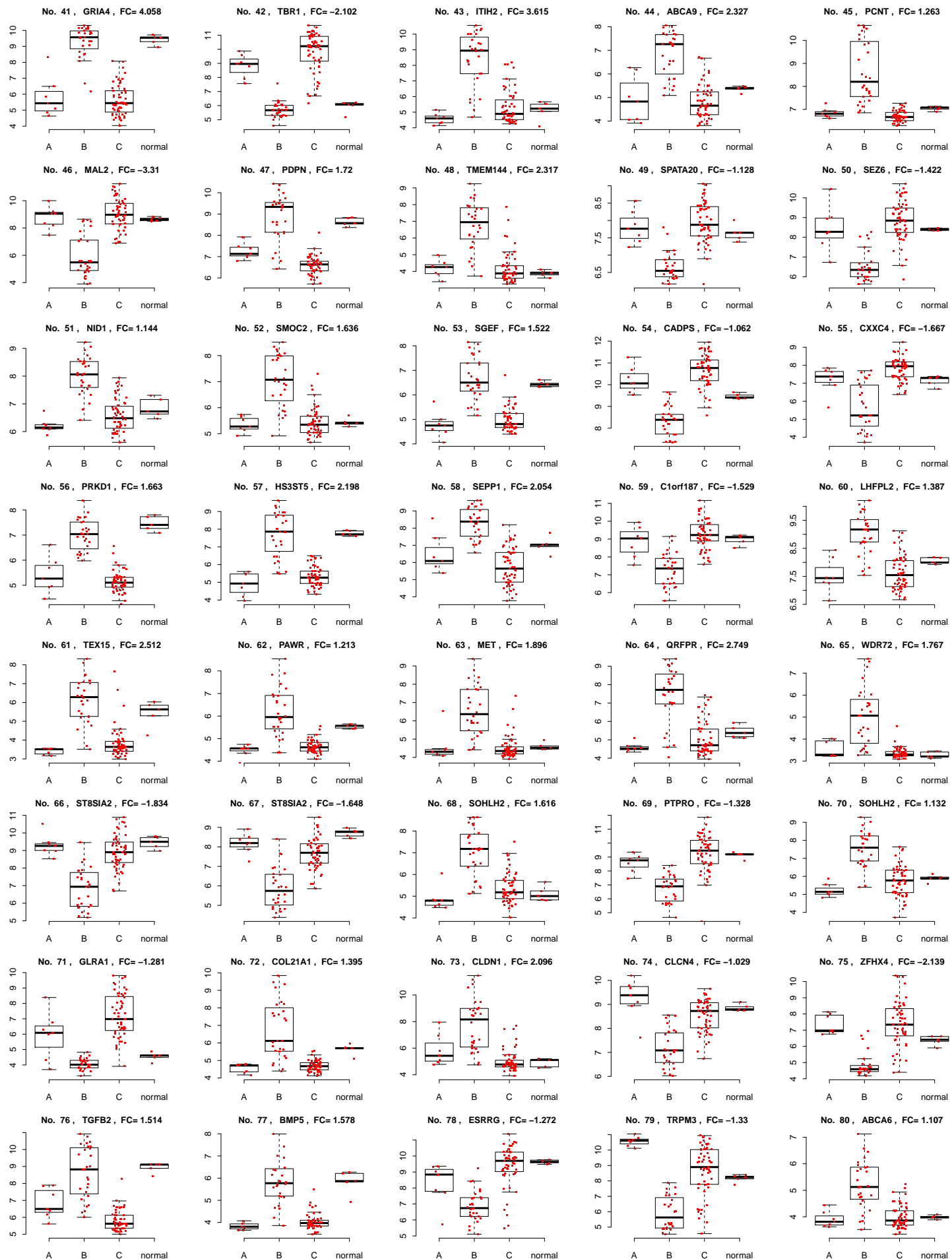

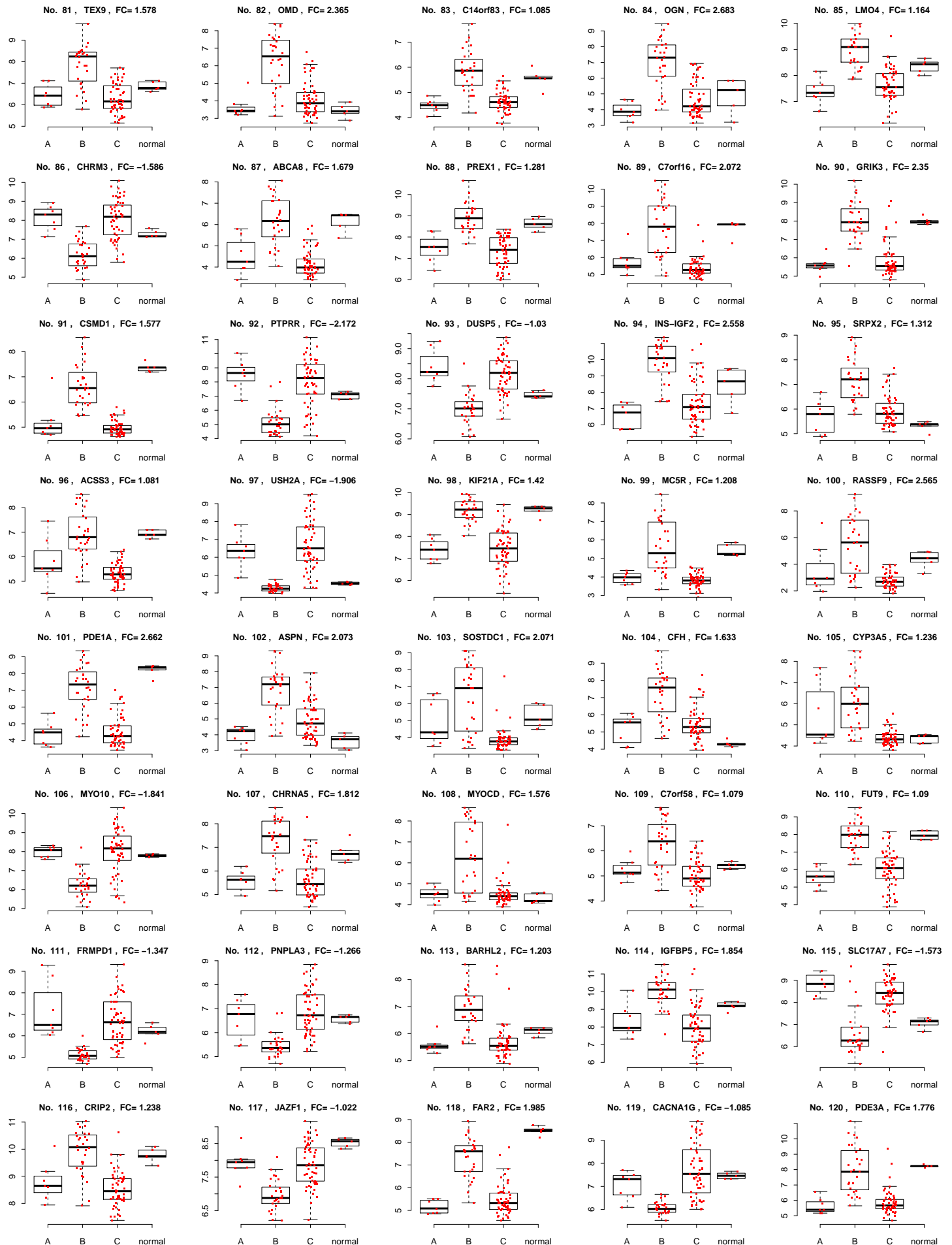

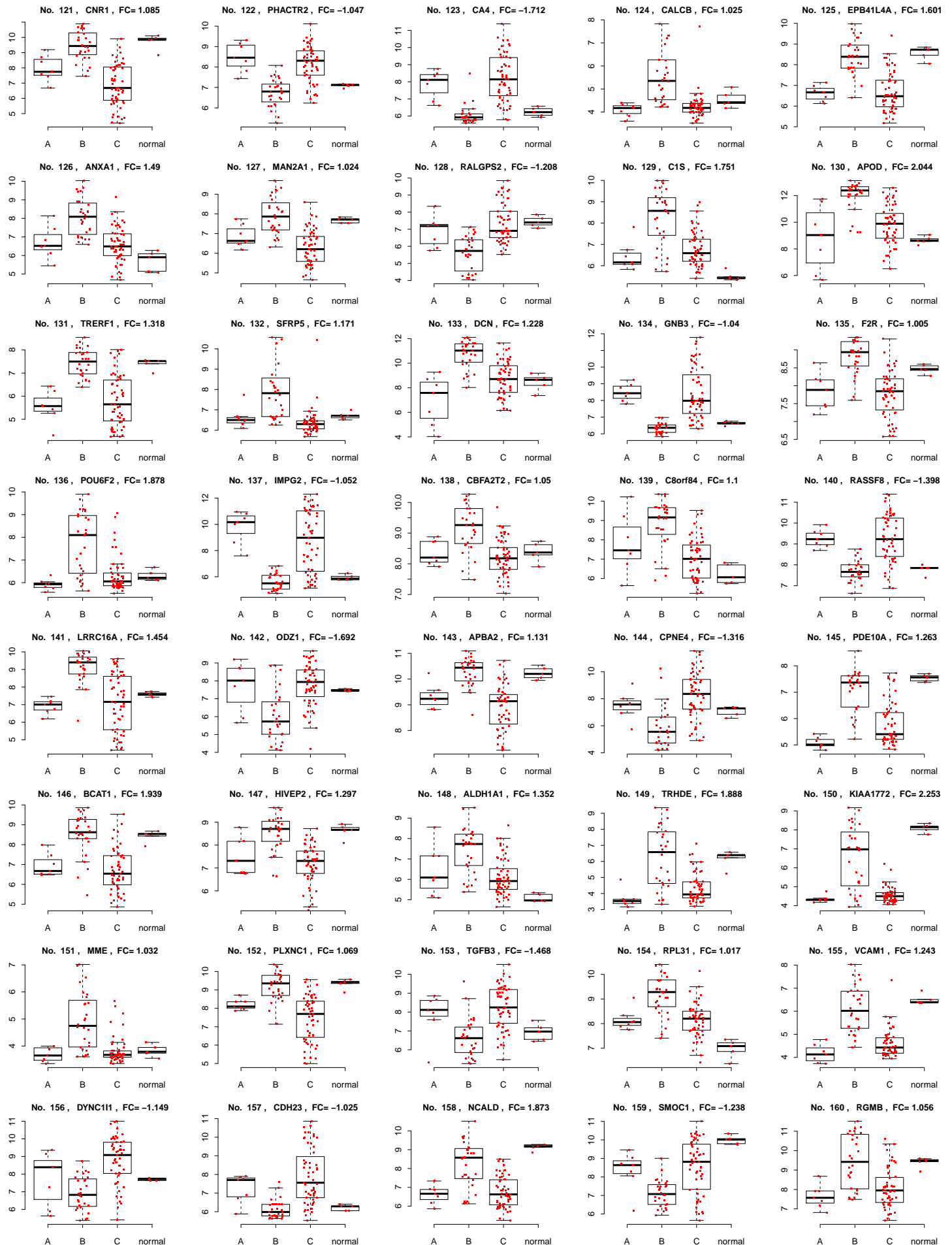

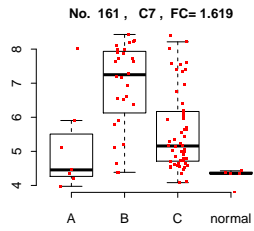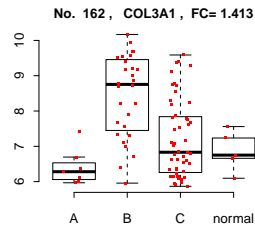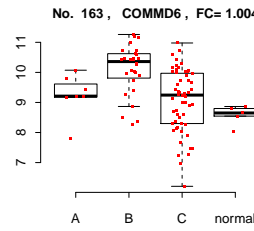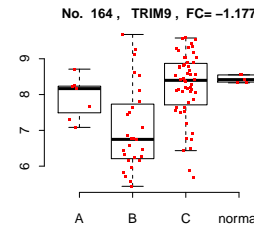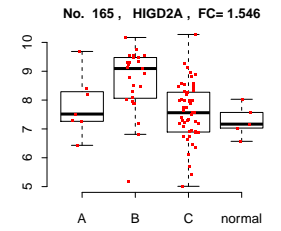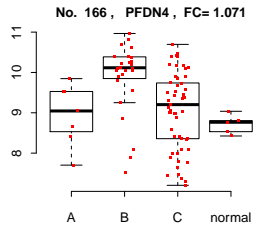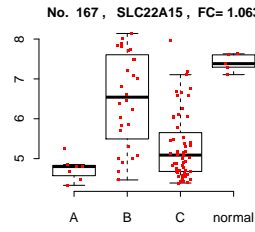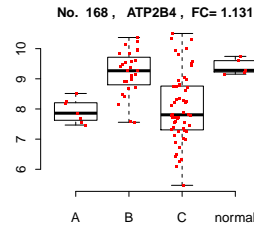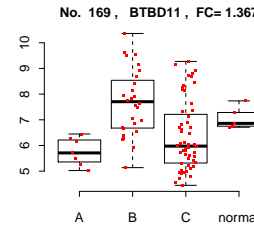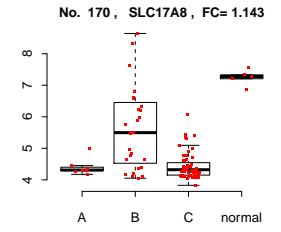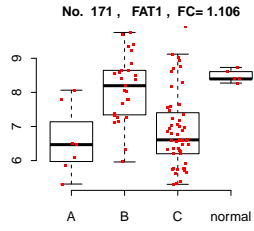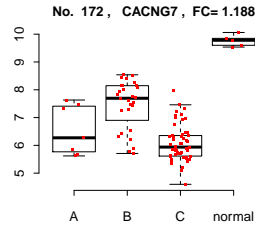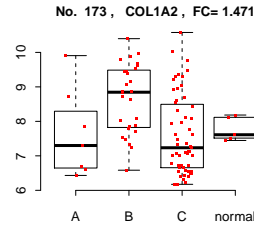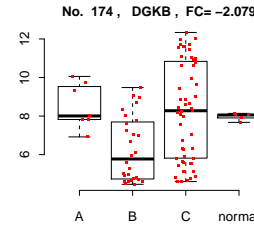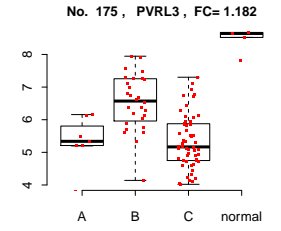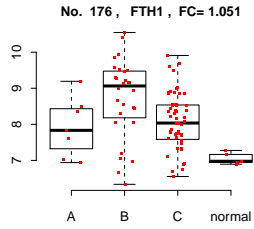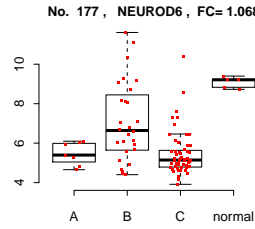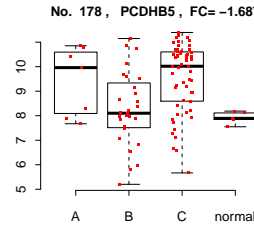

Supplement: Additional file 2 — The converged signatures for the subtypes of the three datasets. [file 1471-2105-14-S18-S1-S2.zip › plot-Northcott90-SubtypeB.pdf]

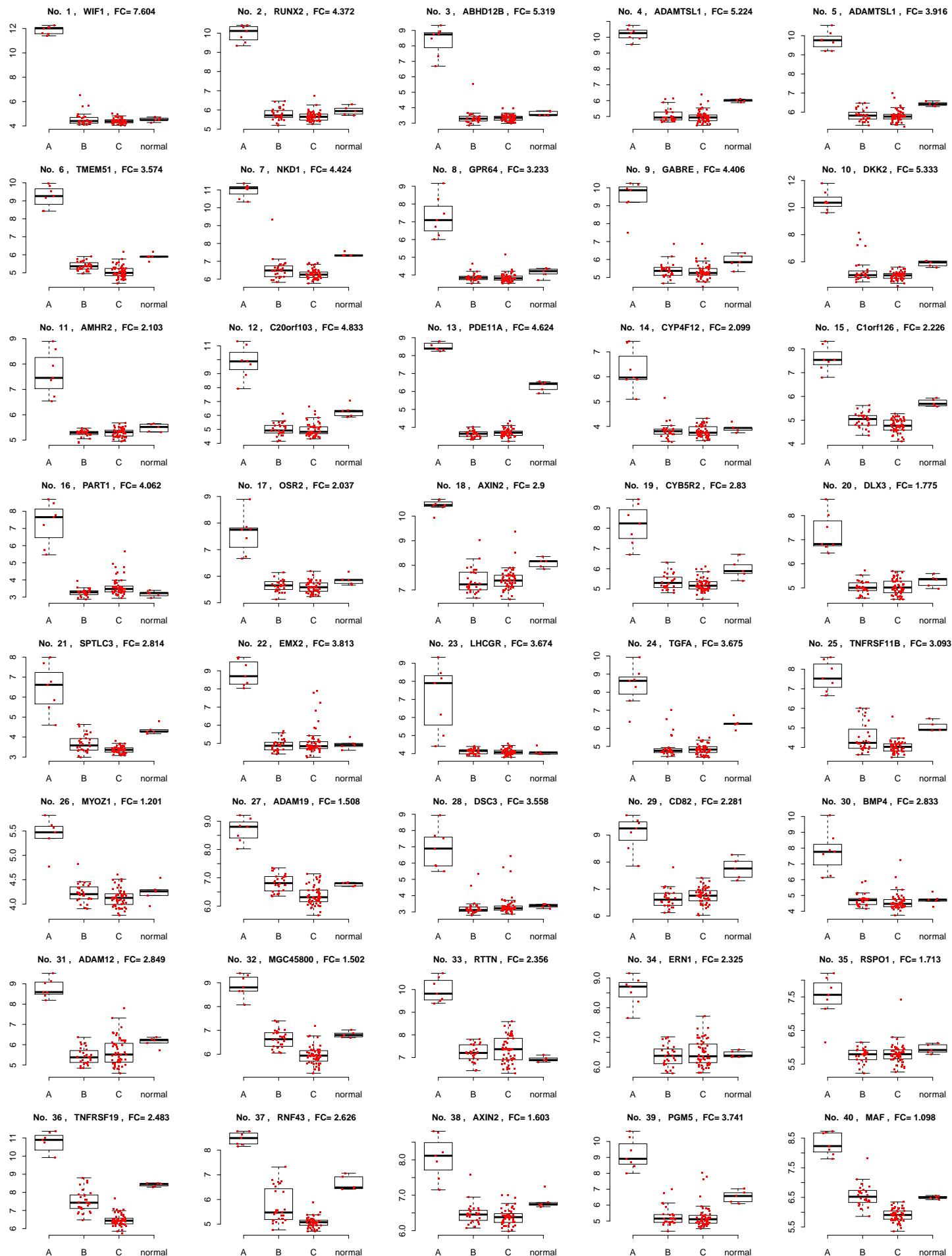

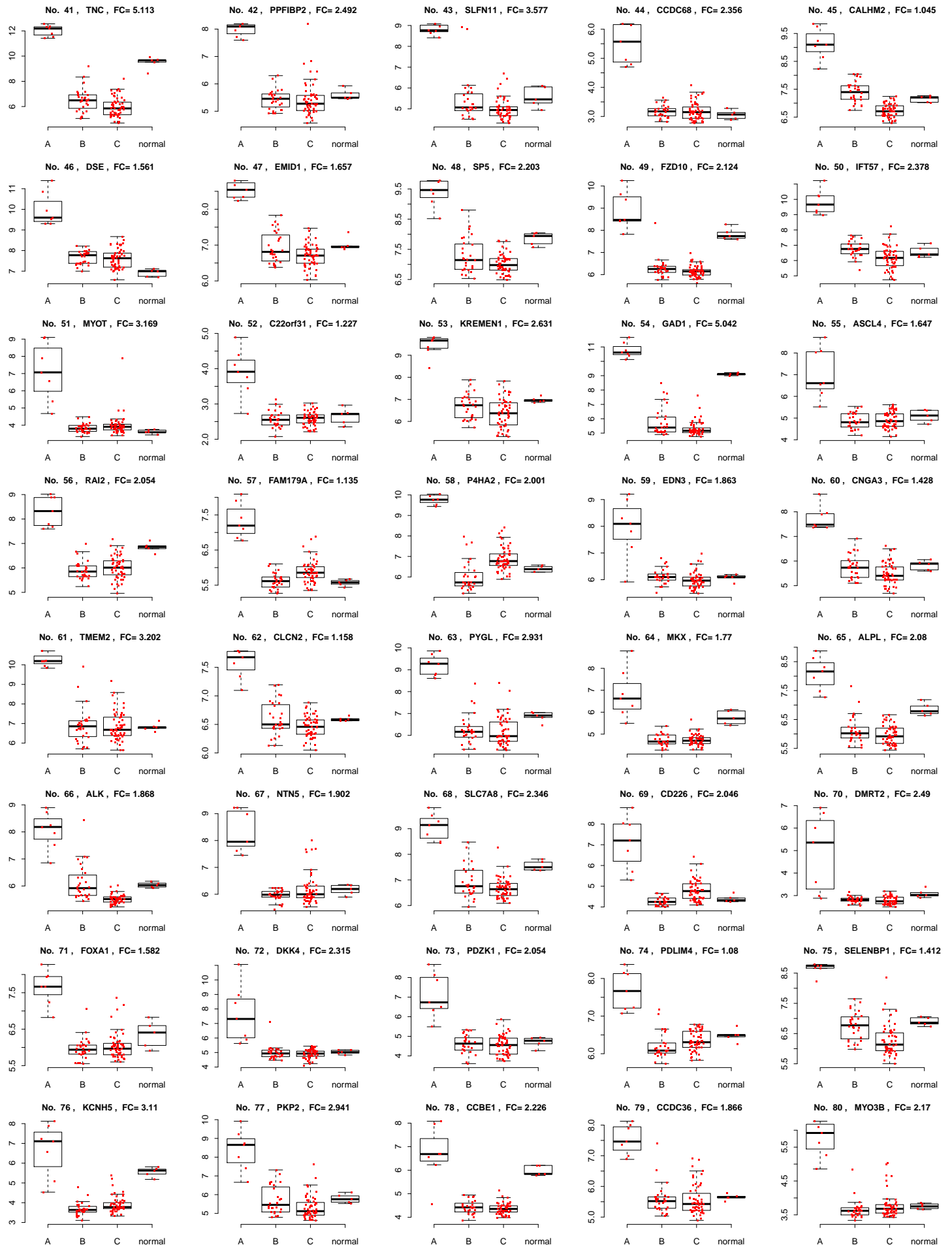

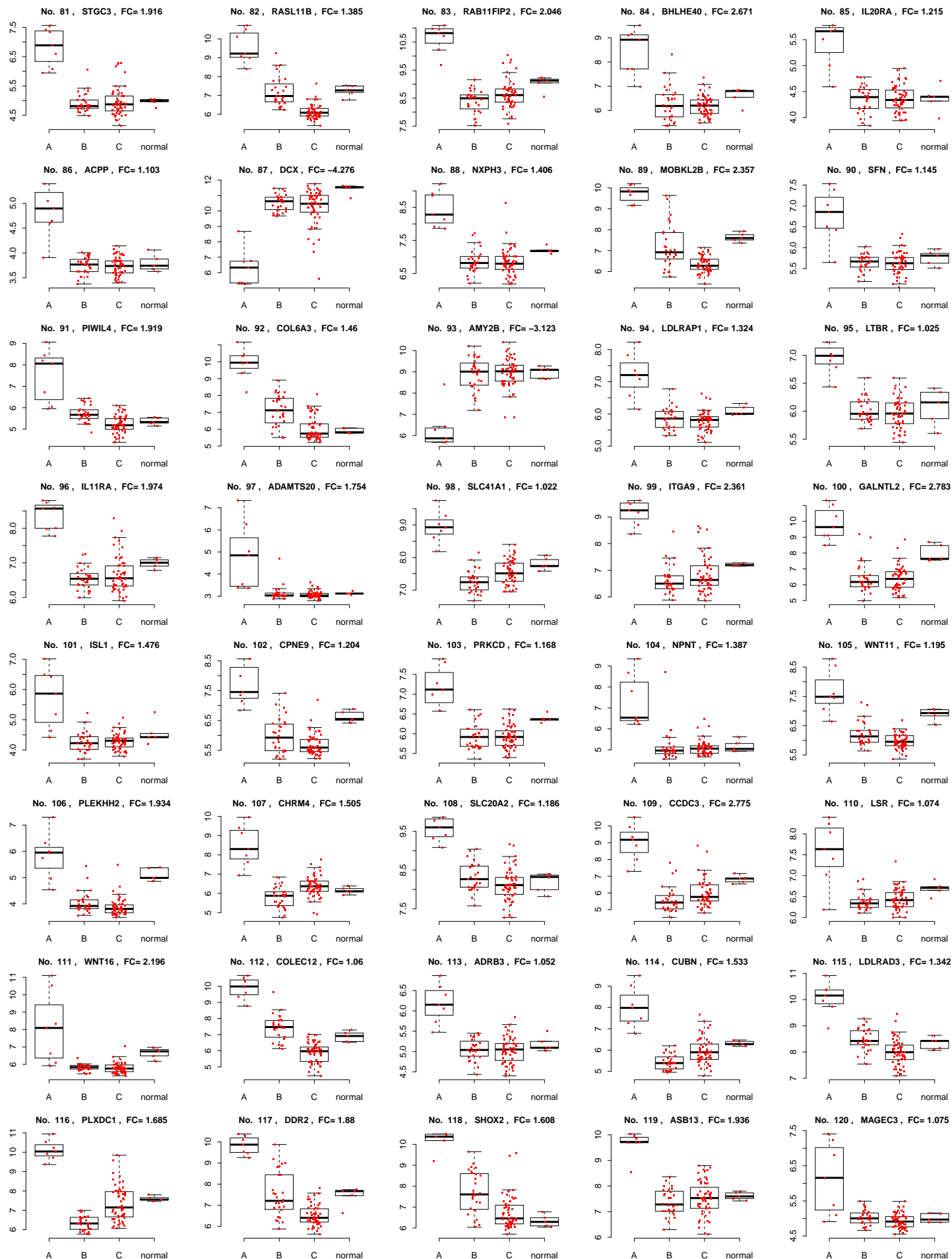

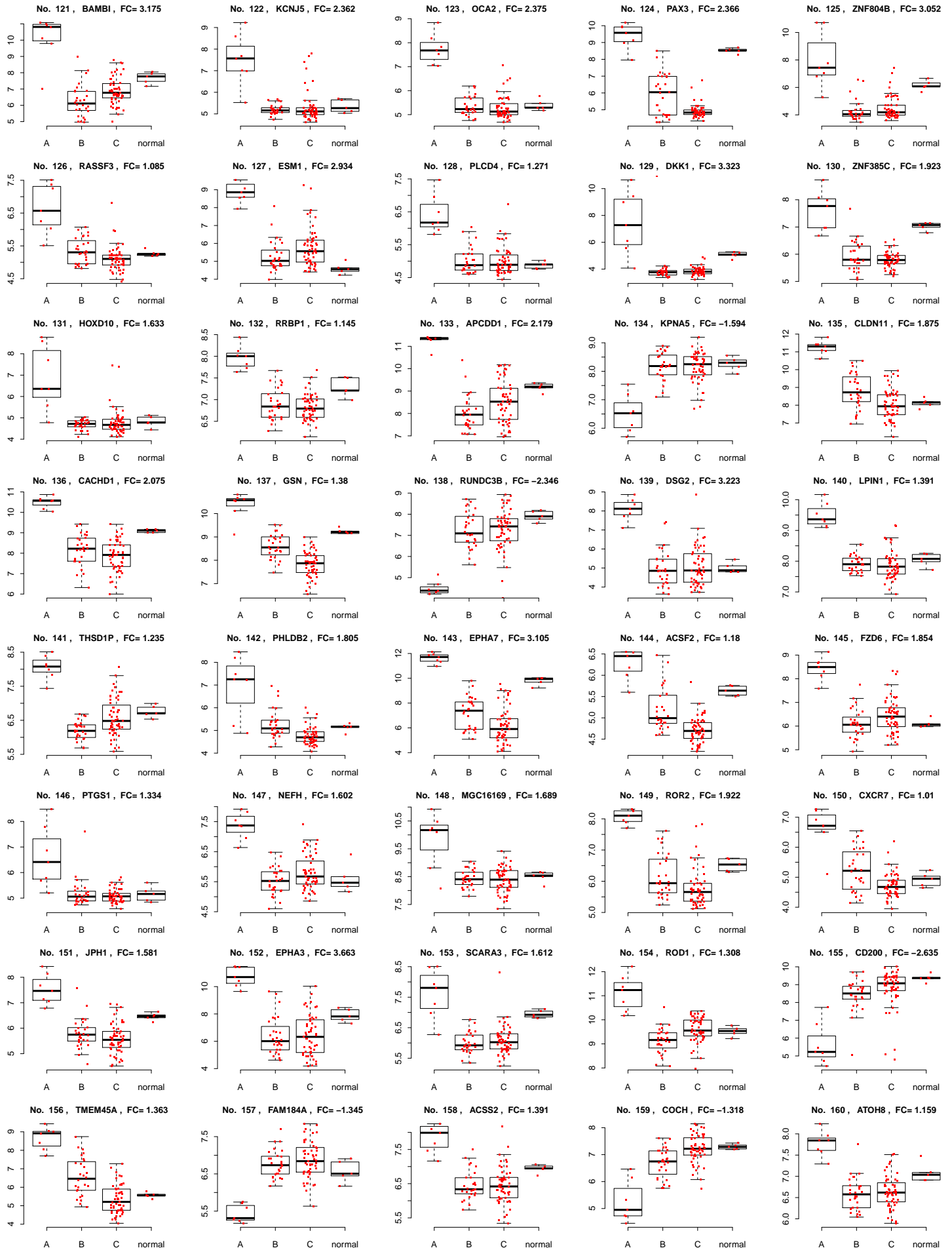

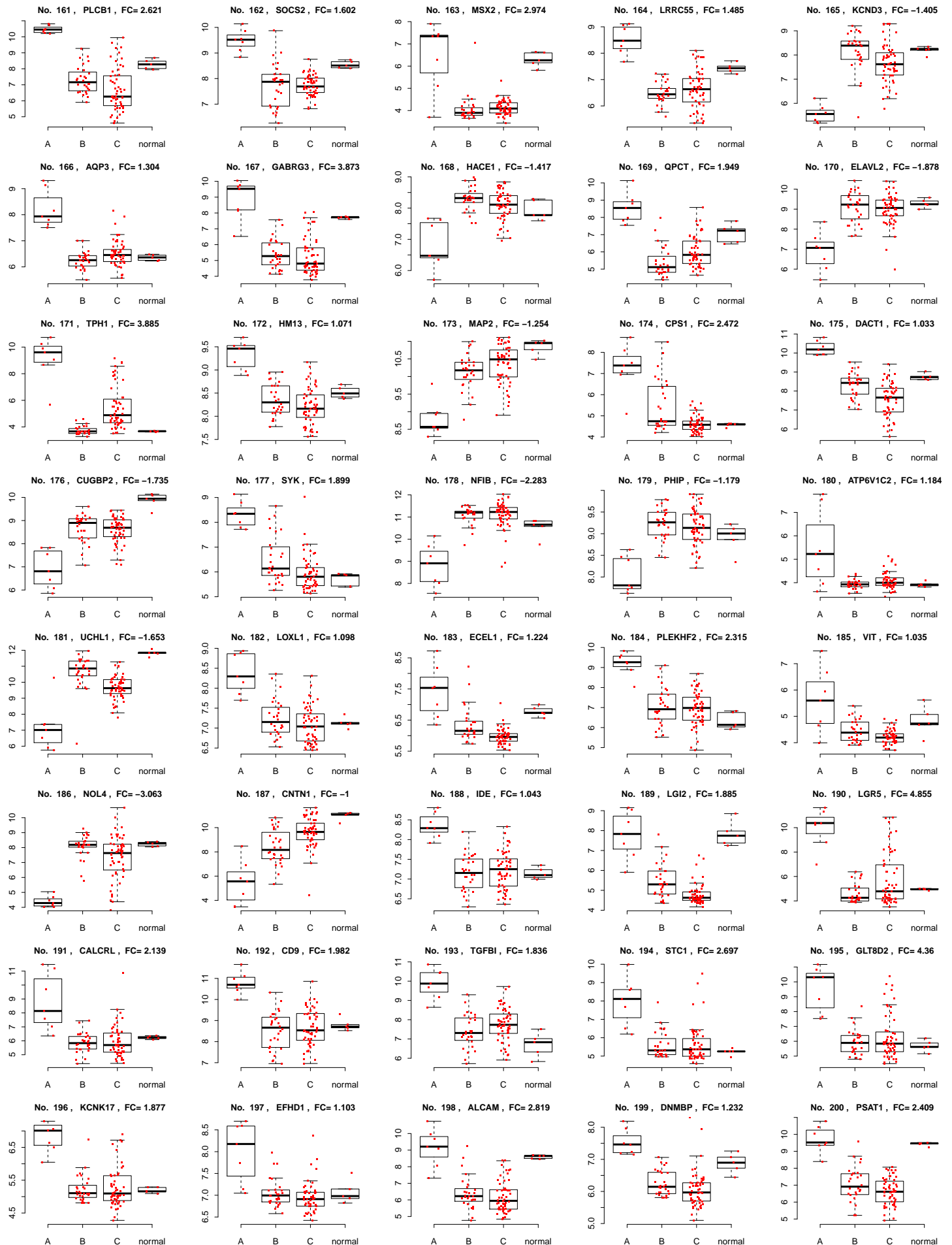

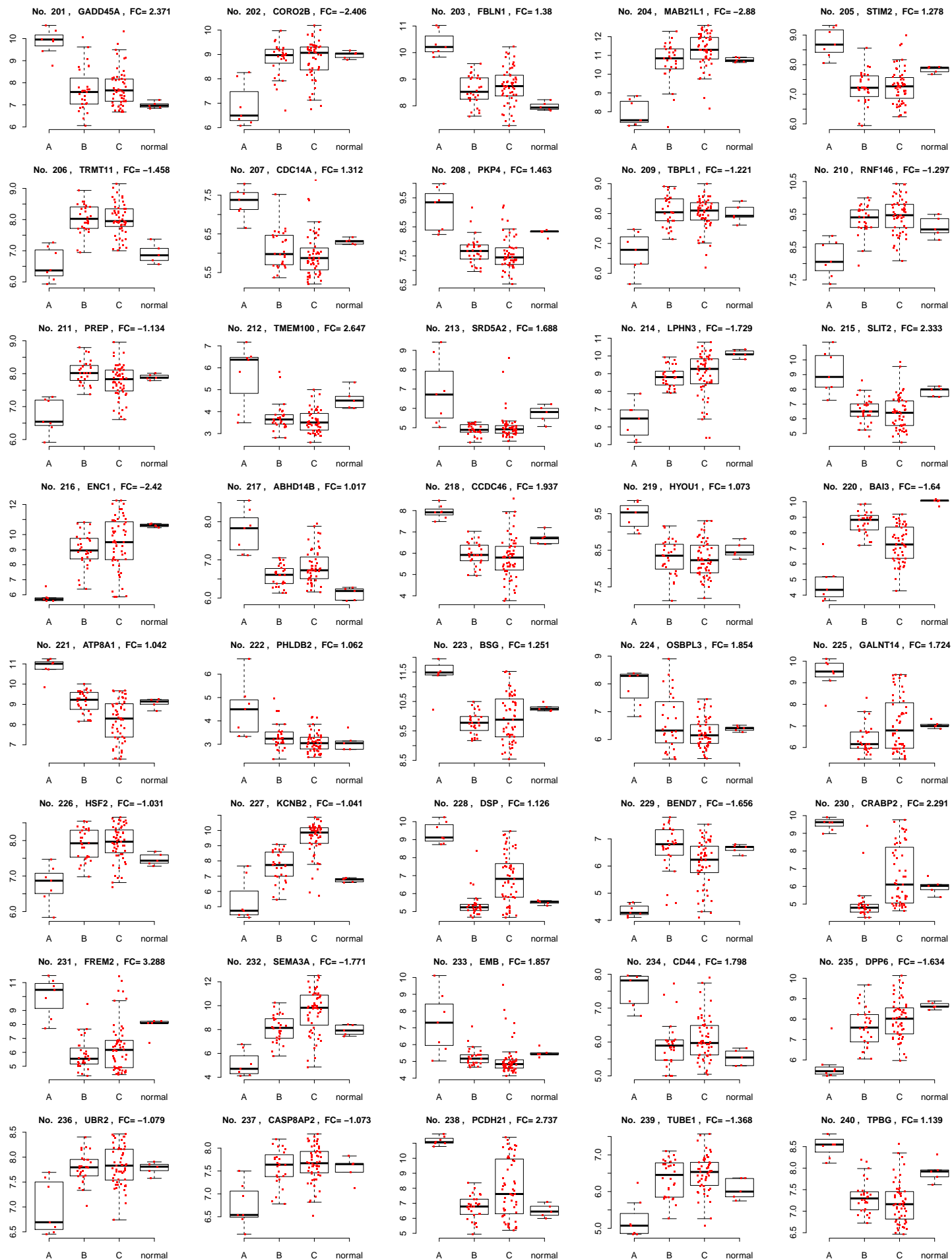

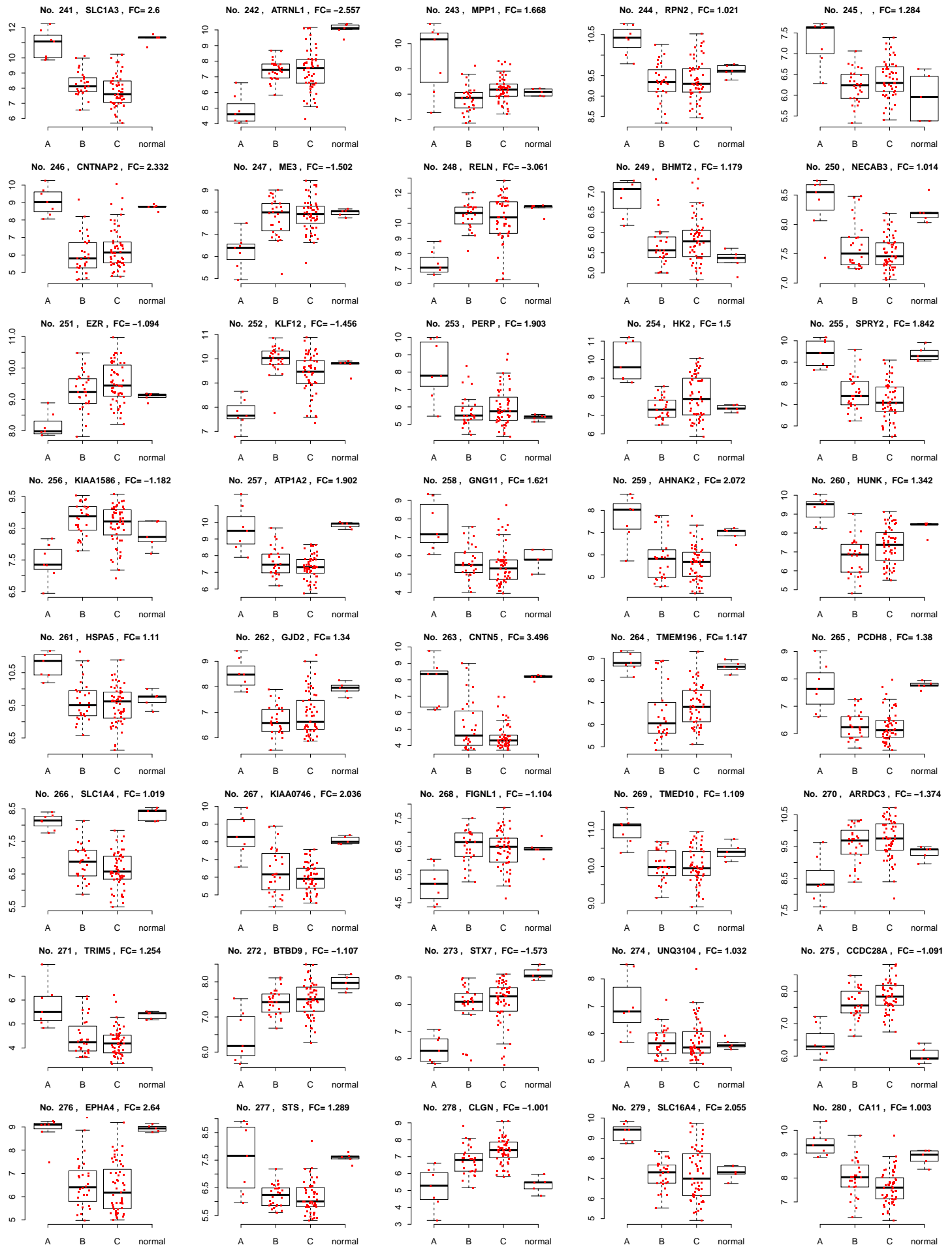

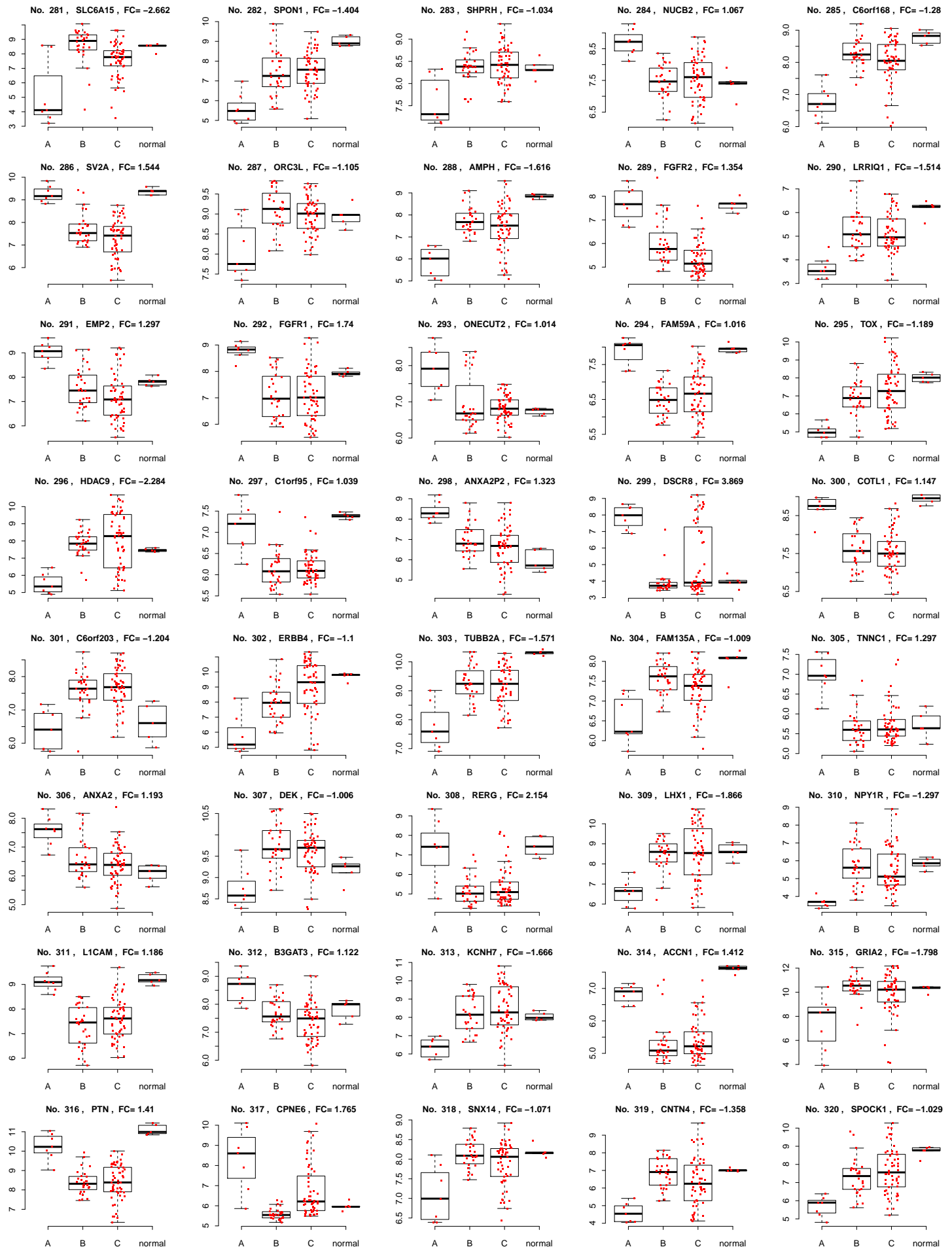

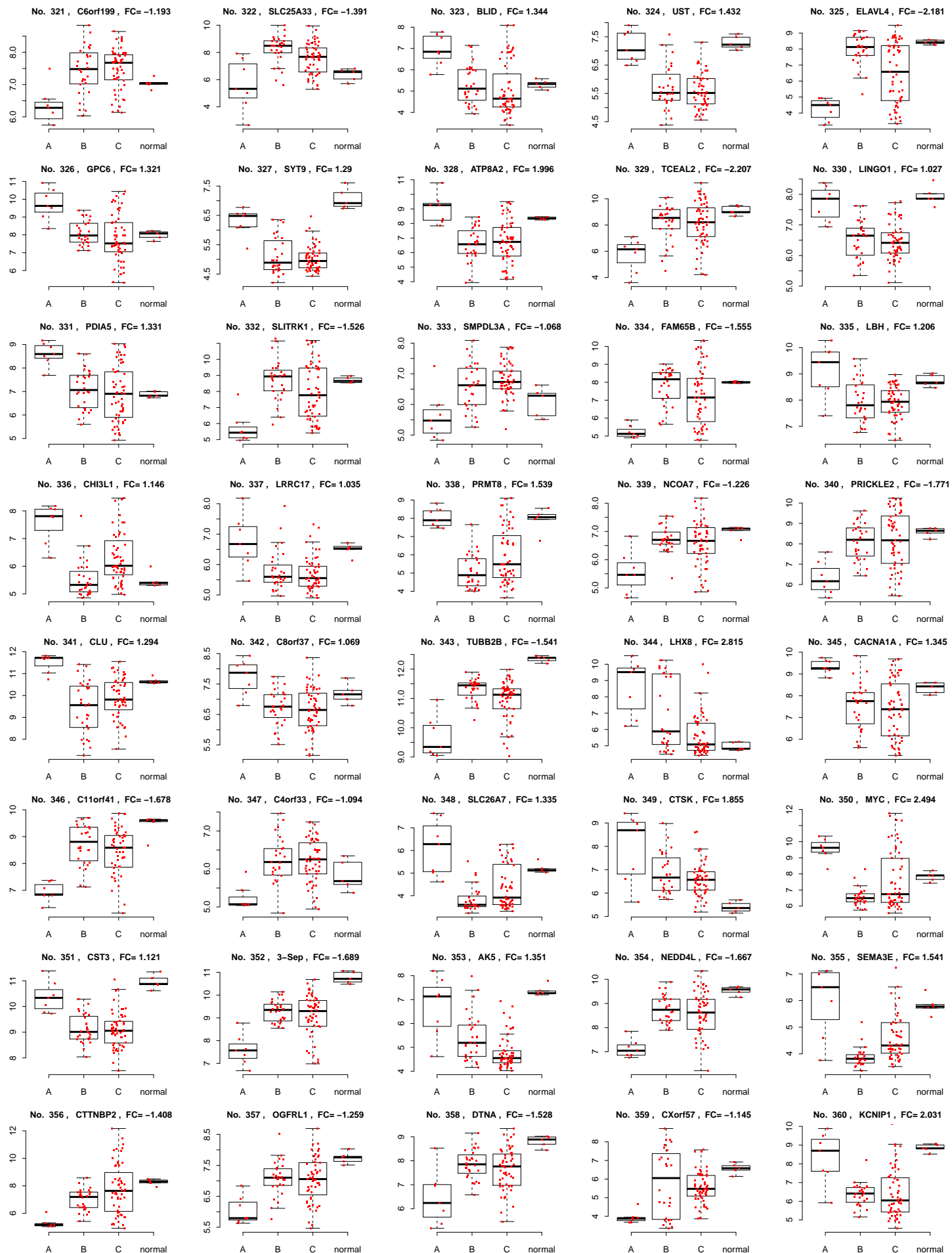

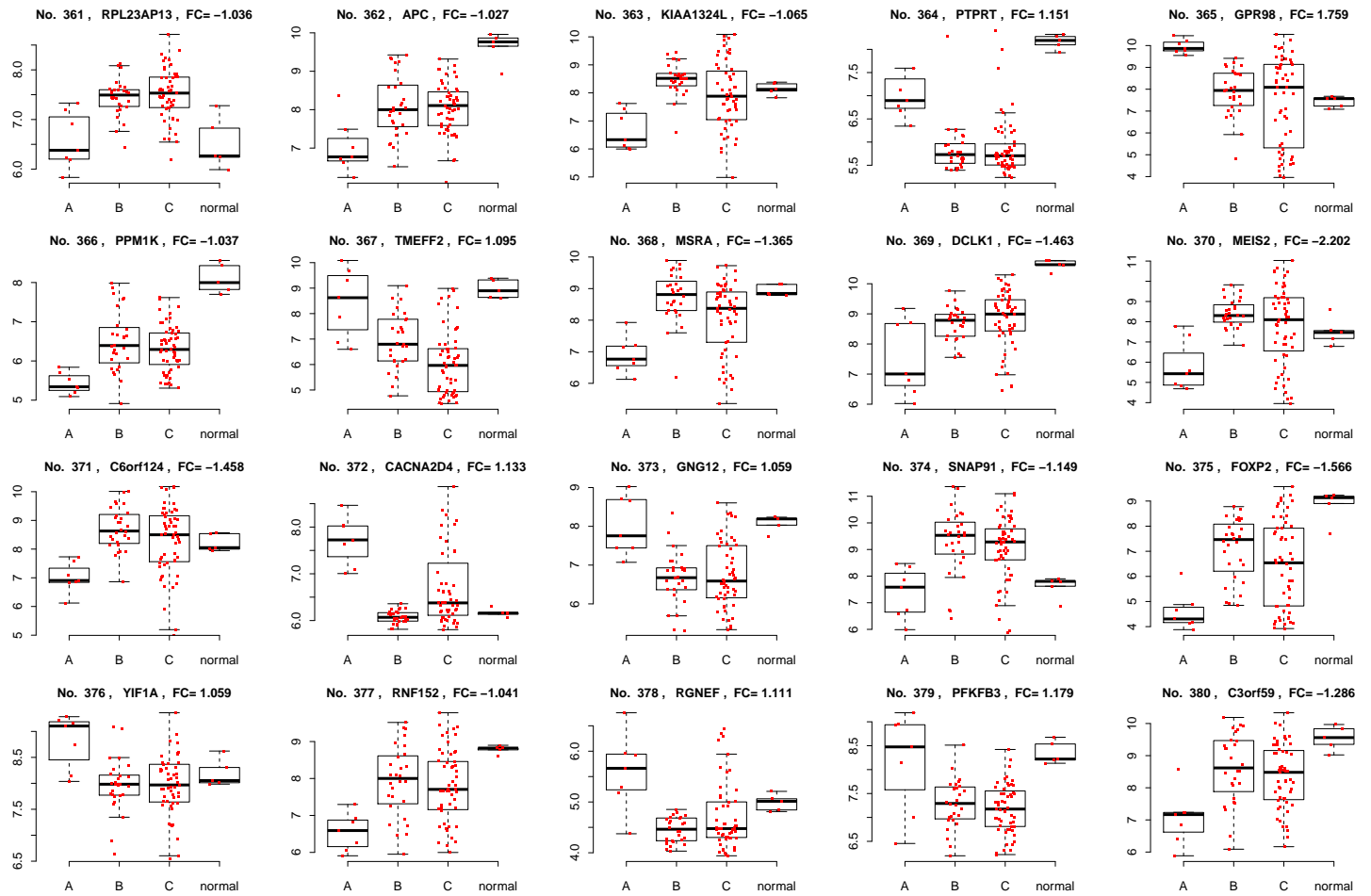

Supplement: Additional file 2 — The converged signatures for the subtypes of the three datasets. [file 1471-2105-14-S18-S1-S2.zip › plot-Northcott90-SubtypeA.pdf]

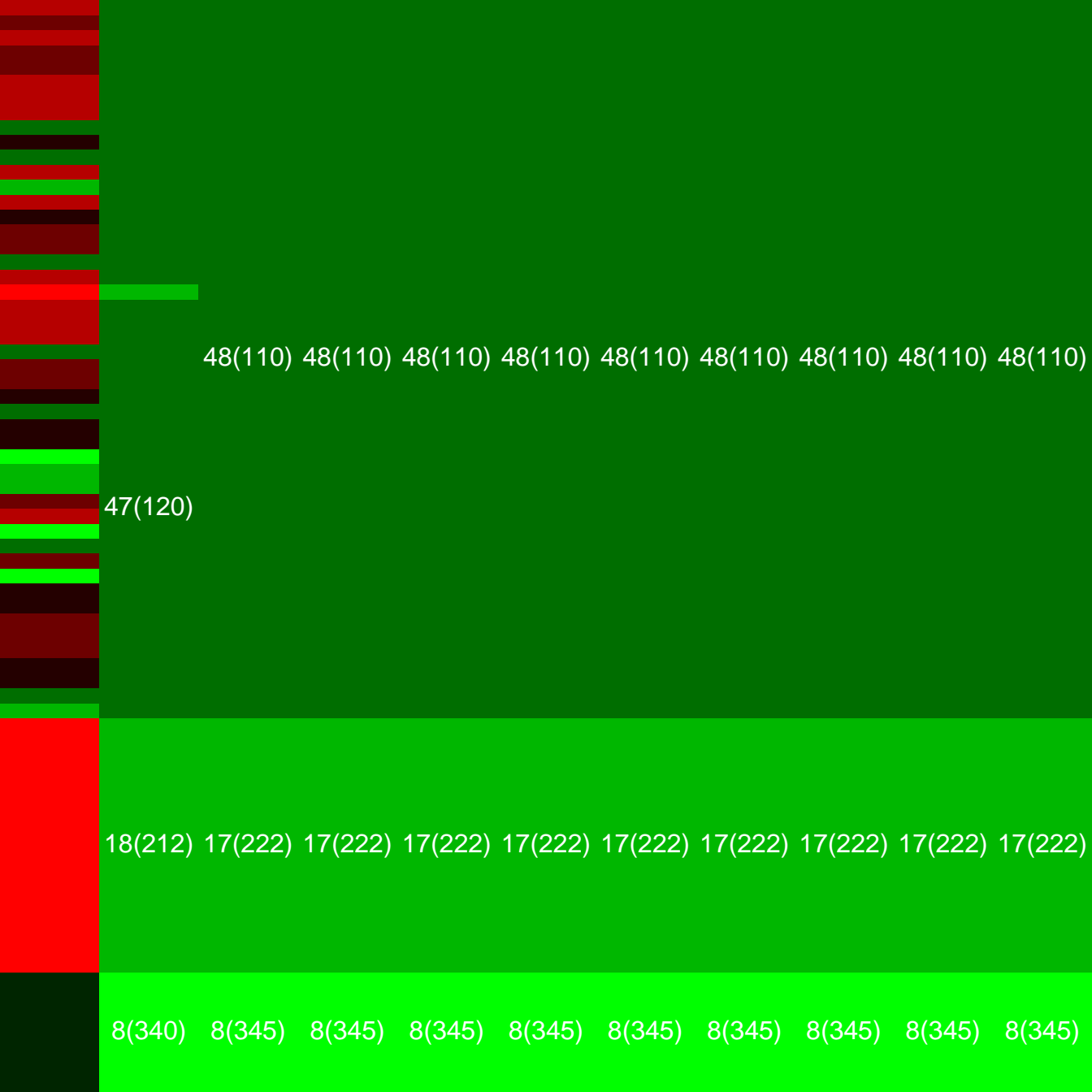

Supplement: Additional file 2 — The converged signatures for the subtypes of the three datasets. [file 1471-2105-14-S18-S1-S2.zip › convergeMap-Cho73.pdf]

# Cho73

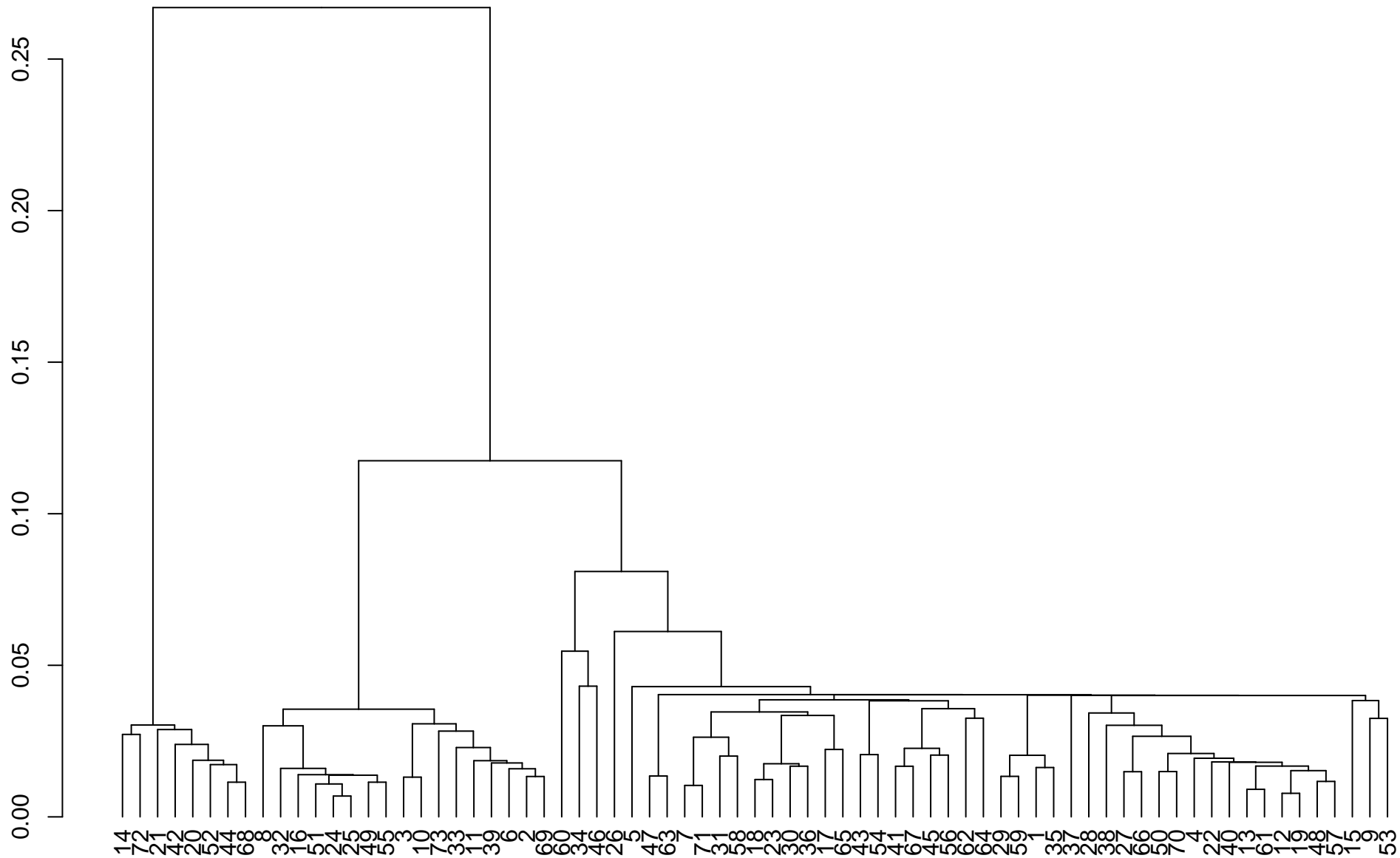

Supplement: Additional file 2 — The converged signatures for the subtypes of the three datasets. [file 1471-2105-14-S18-S1-S2.zip › convergeMap-Cho73-dendrogram.pdf]

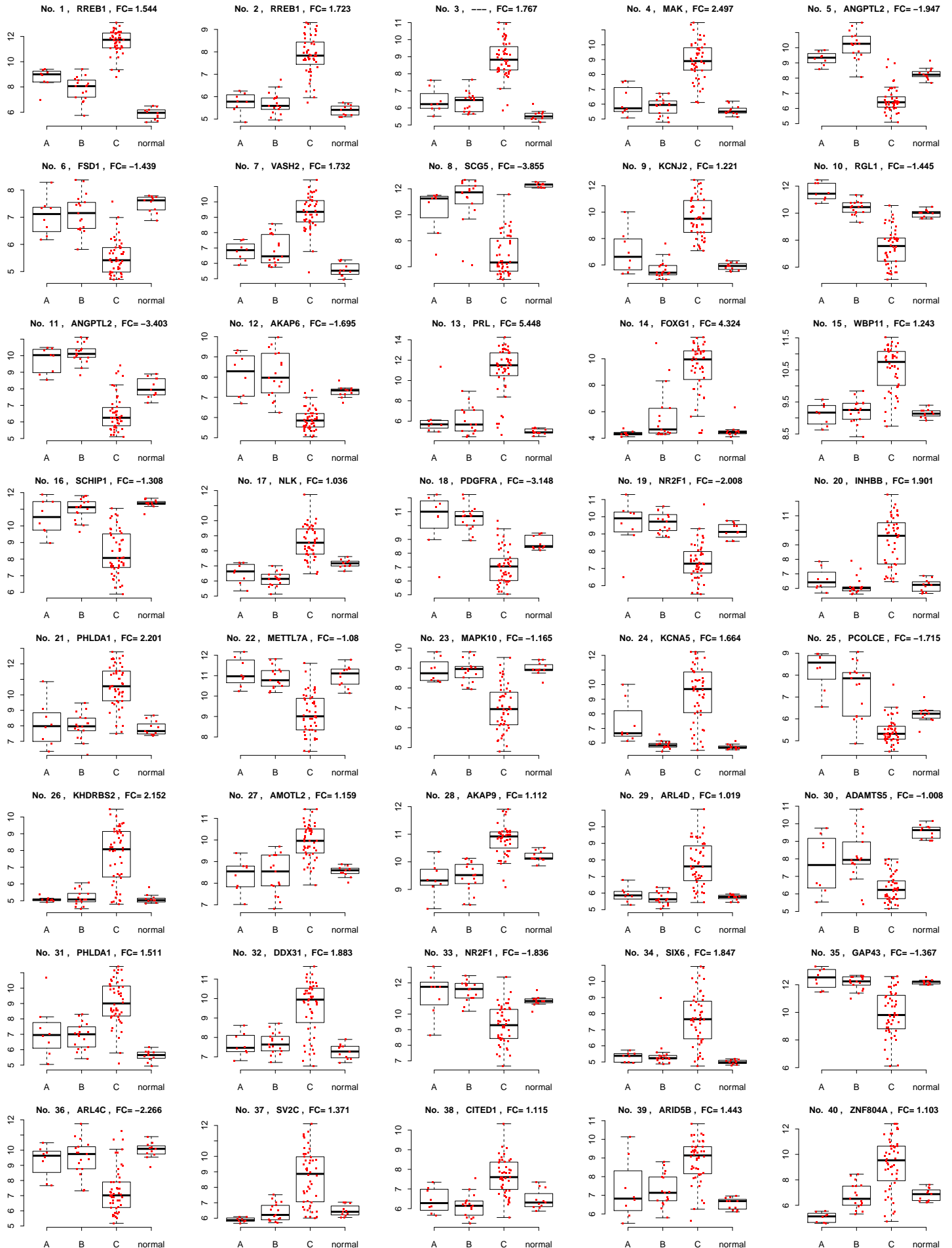

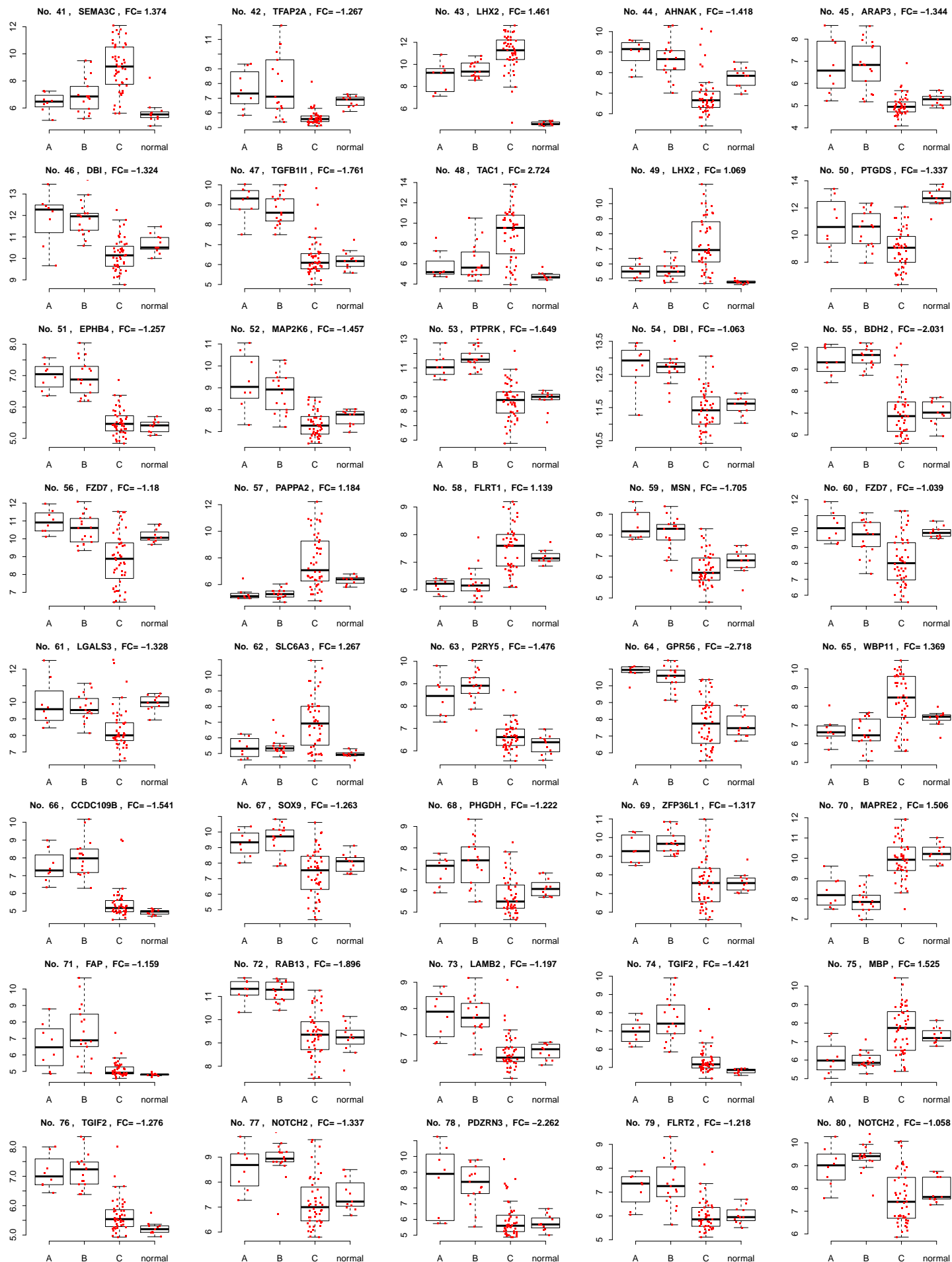

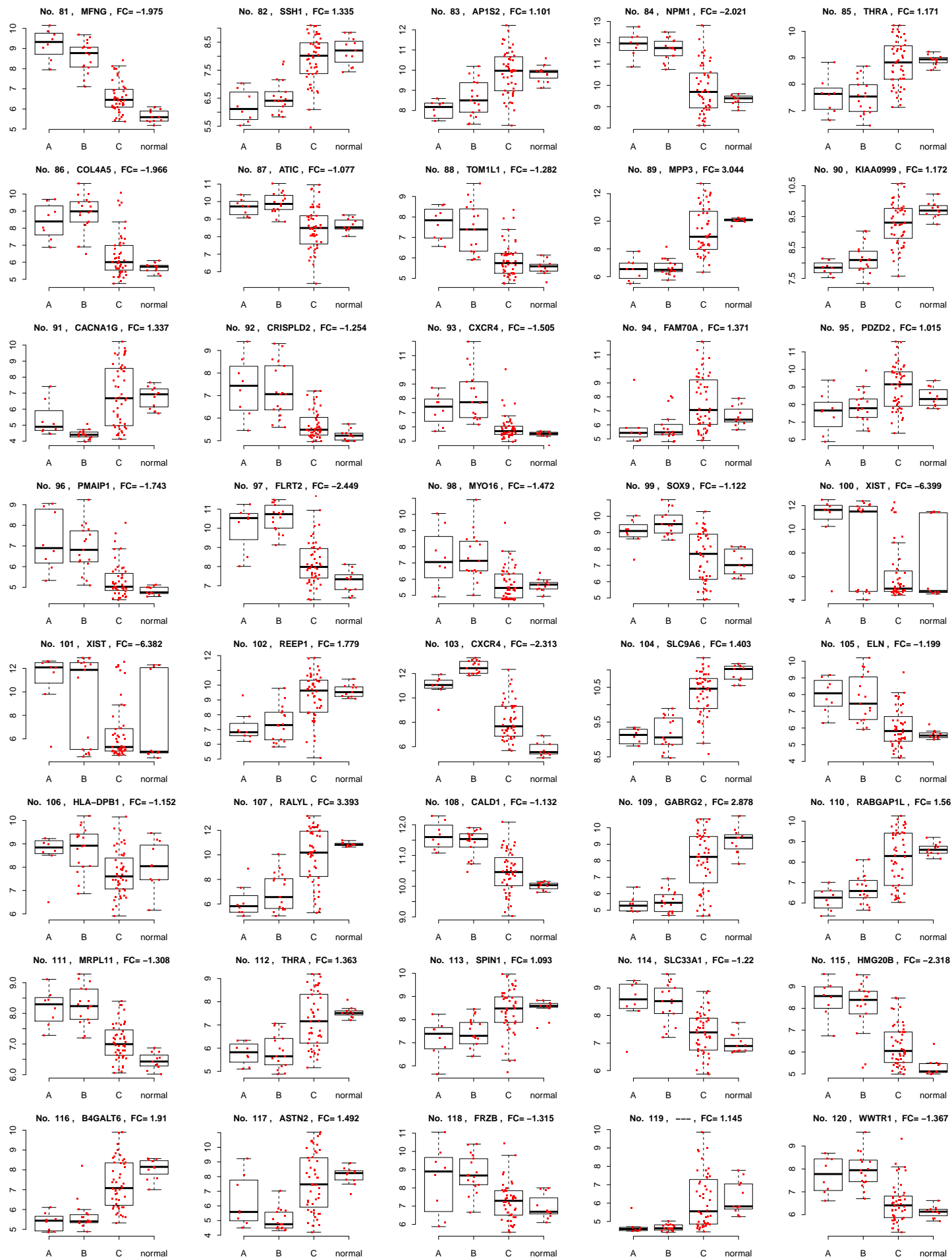

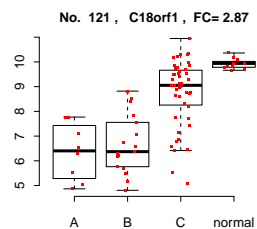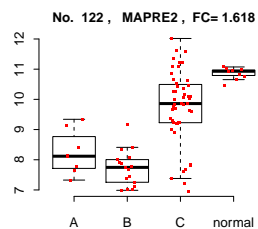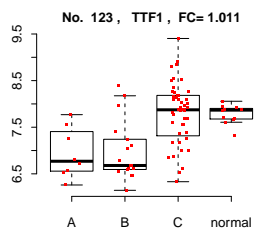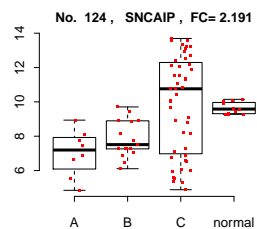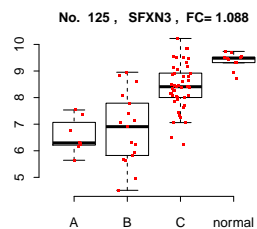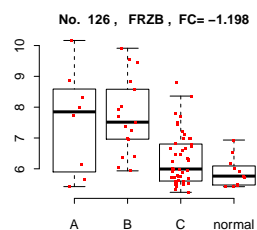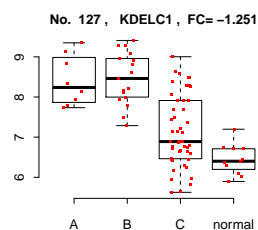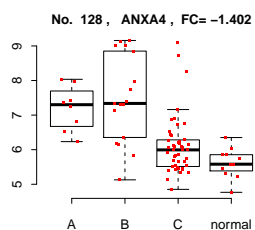

Supplement: Additional file 2 — The converged signatures for the subtypes of the three datasets. [file 1471-2105-14-S18-S1-S2.zip › plot-Cho73-SubtypeC.pdf]

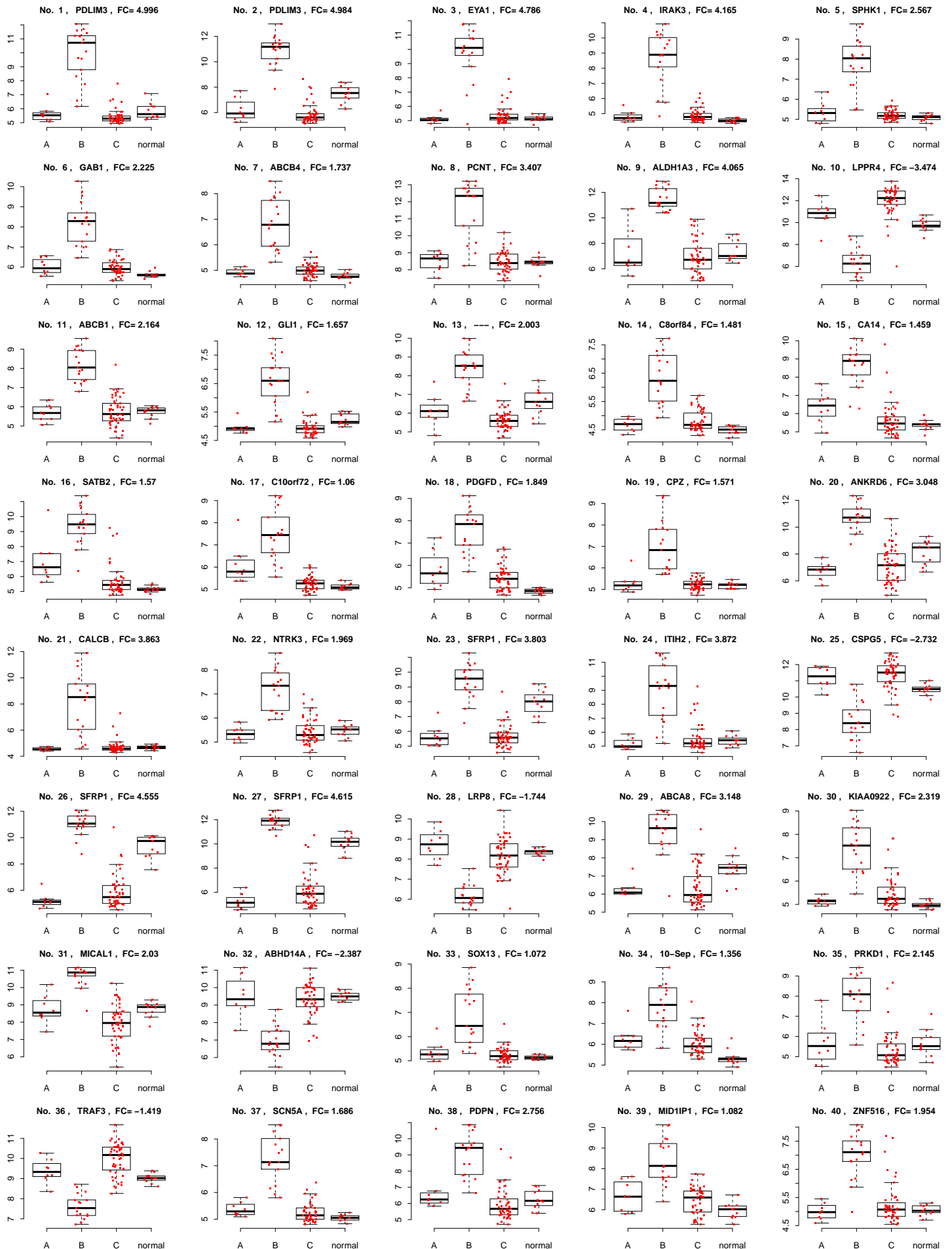

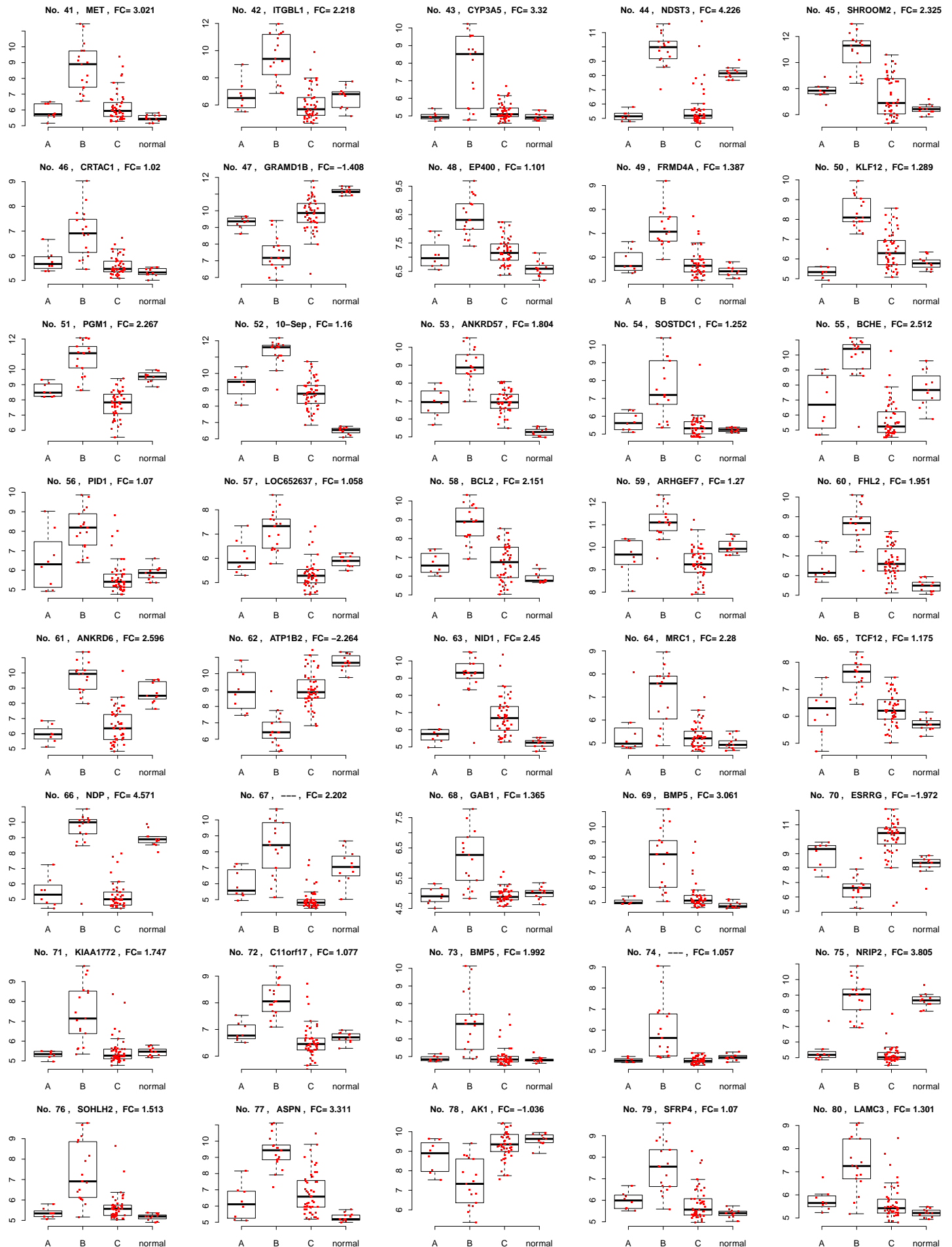

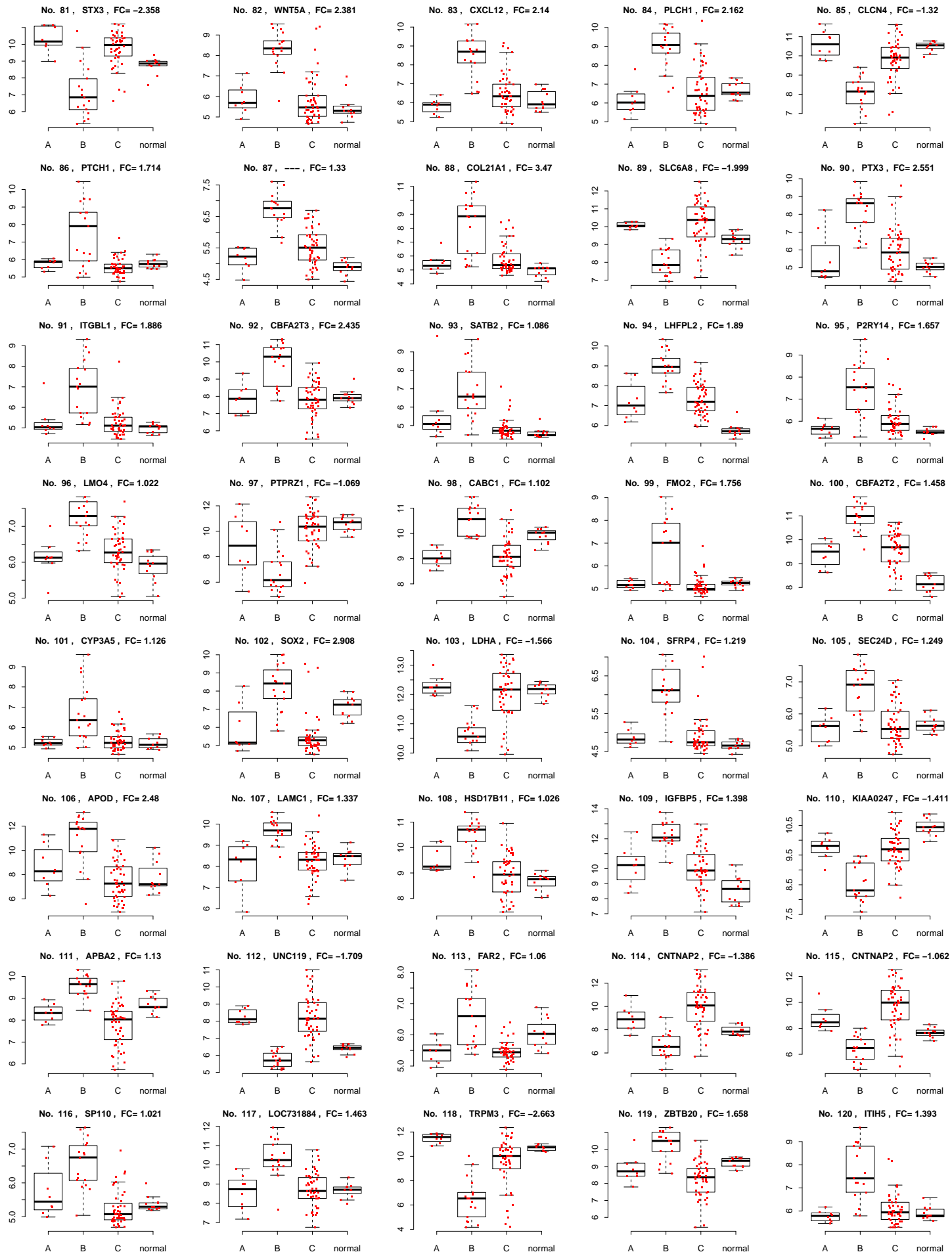

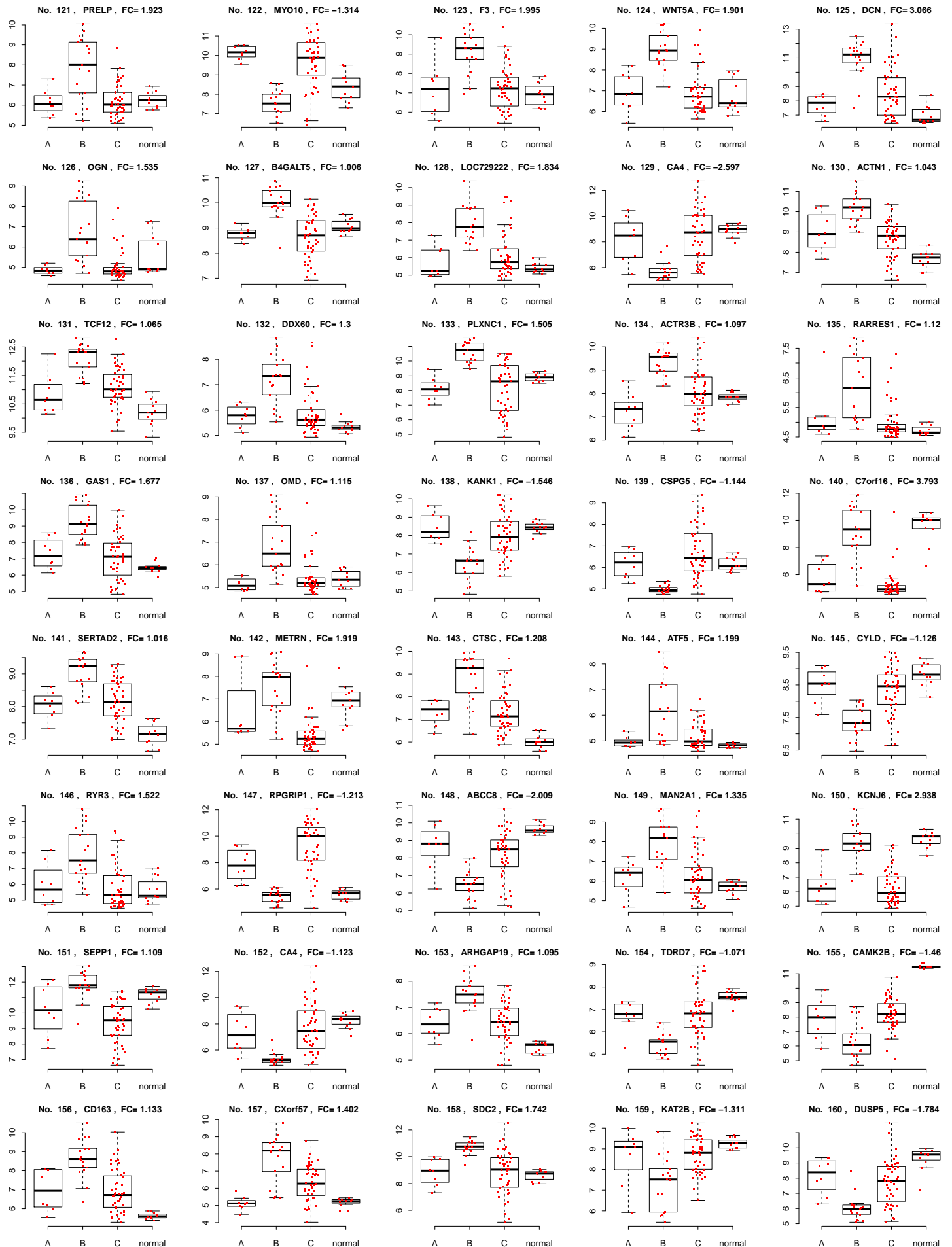

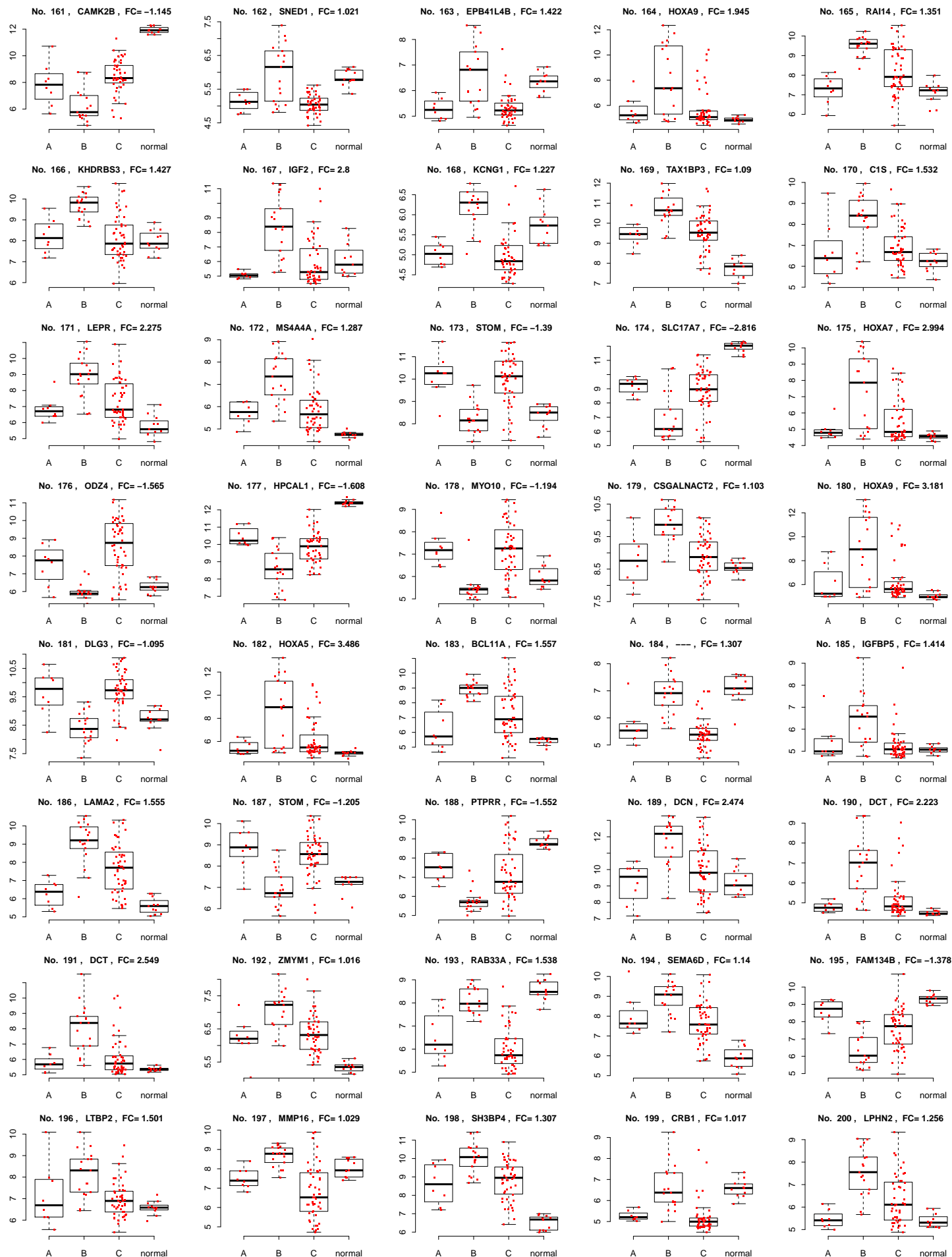

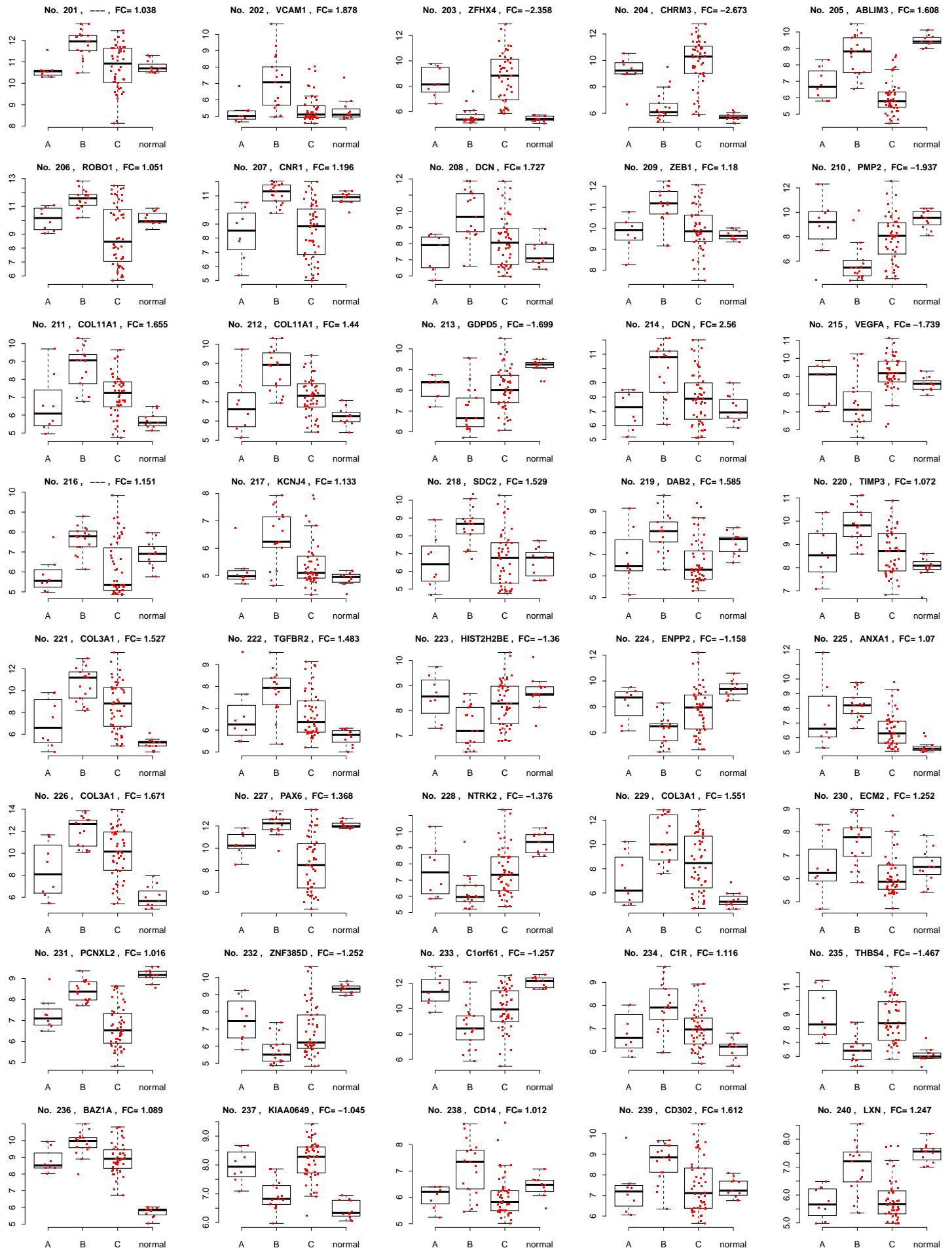

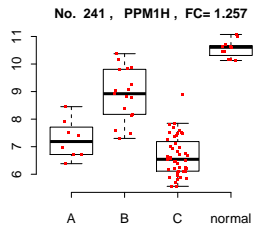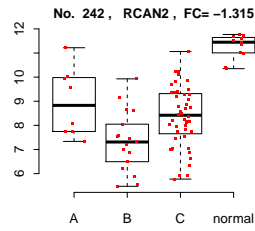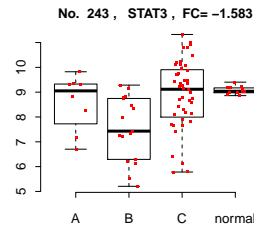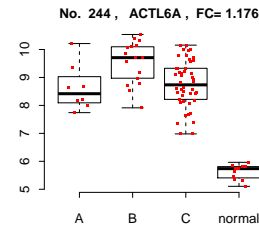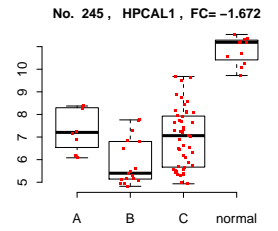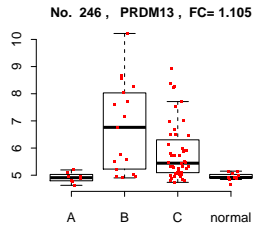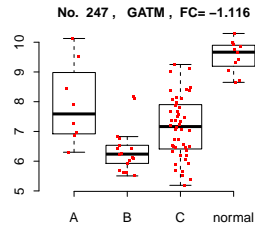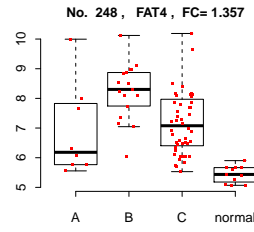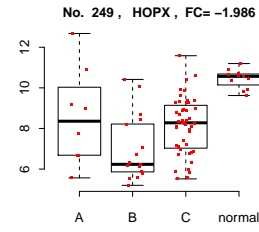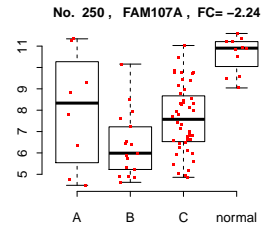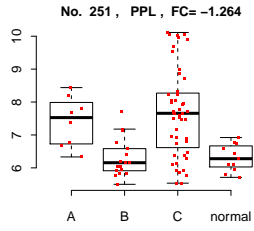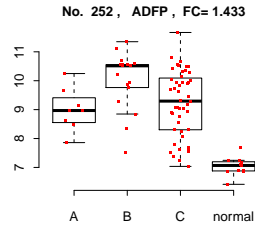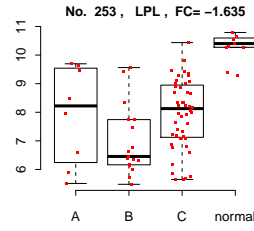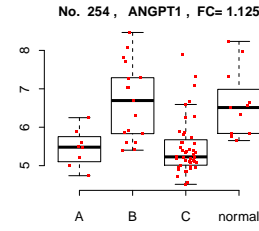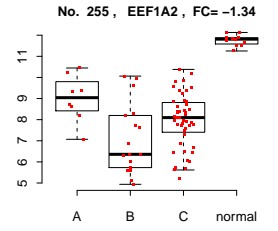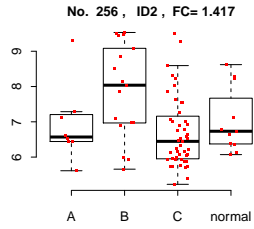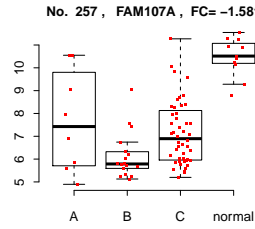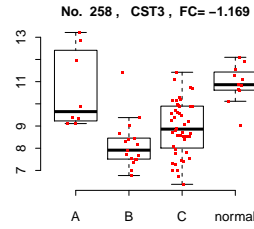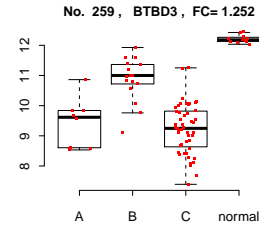

Supplement: Additional file 2 — The converged signatures for the subtypes of the three datasets. [file 1471-2105-14-S18-S1-S2.zip › plot-Cho73-SubtypeB.pdf]

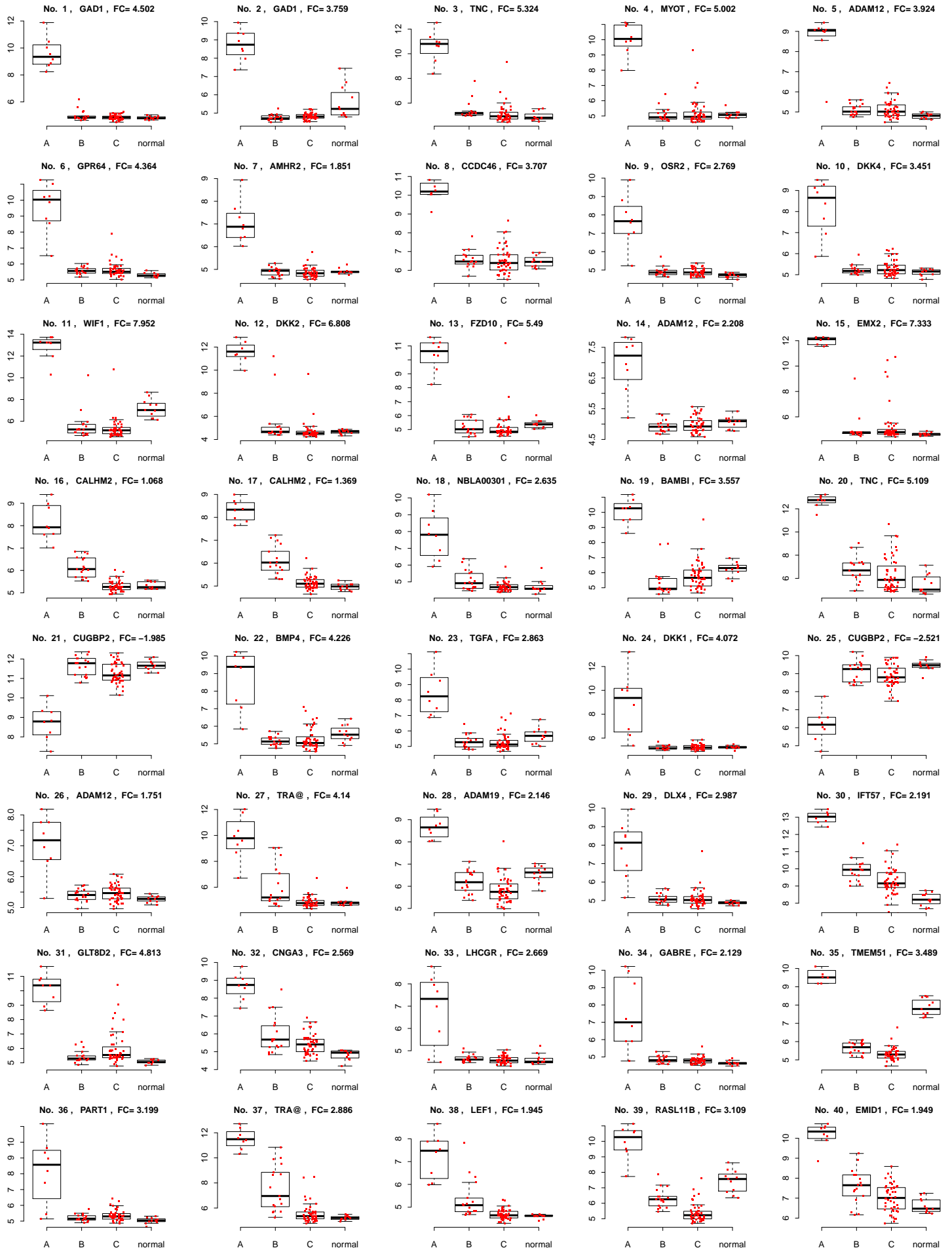

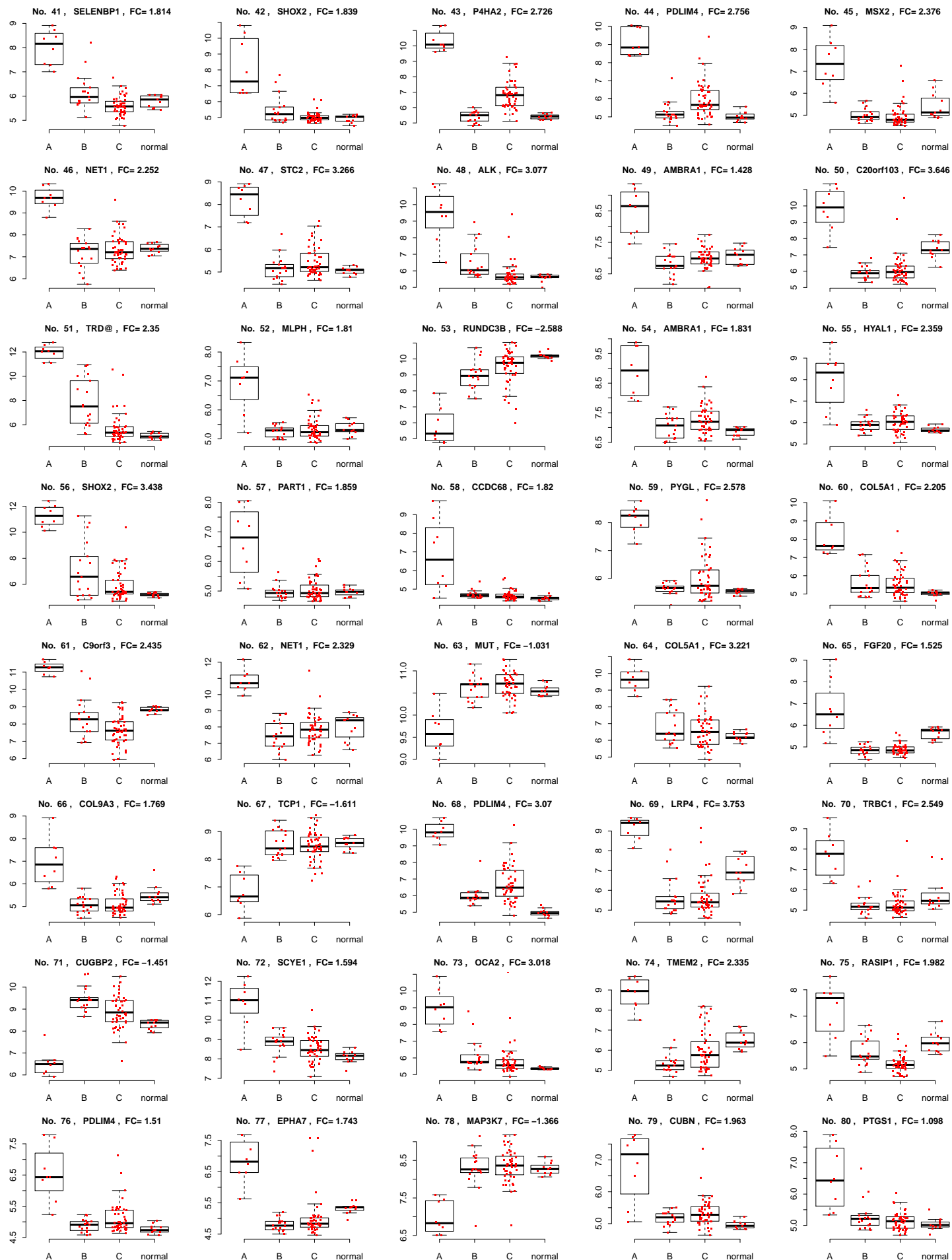

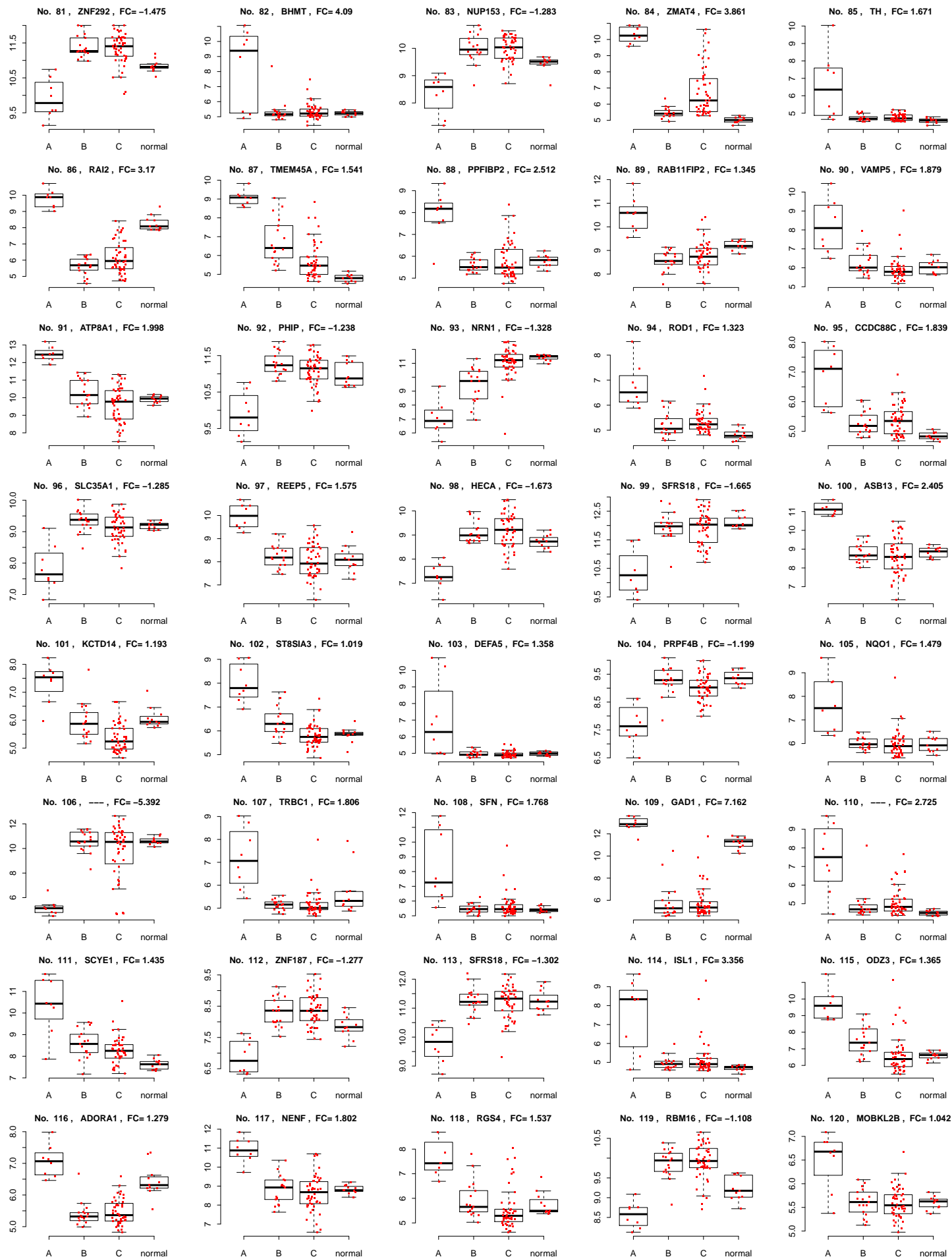

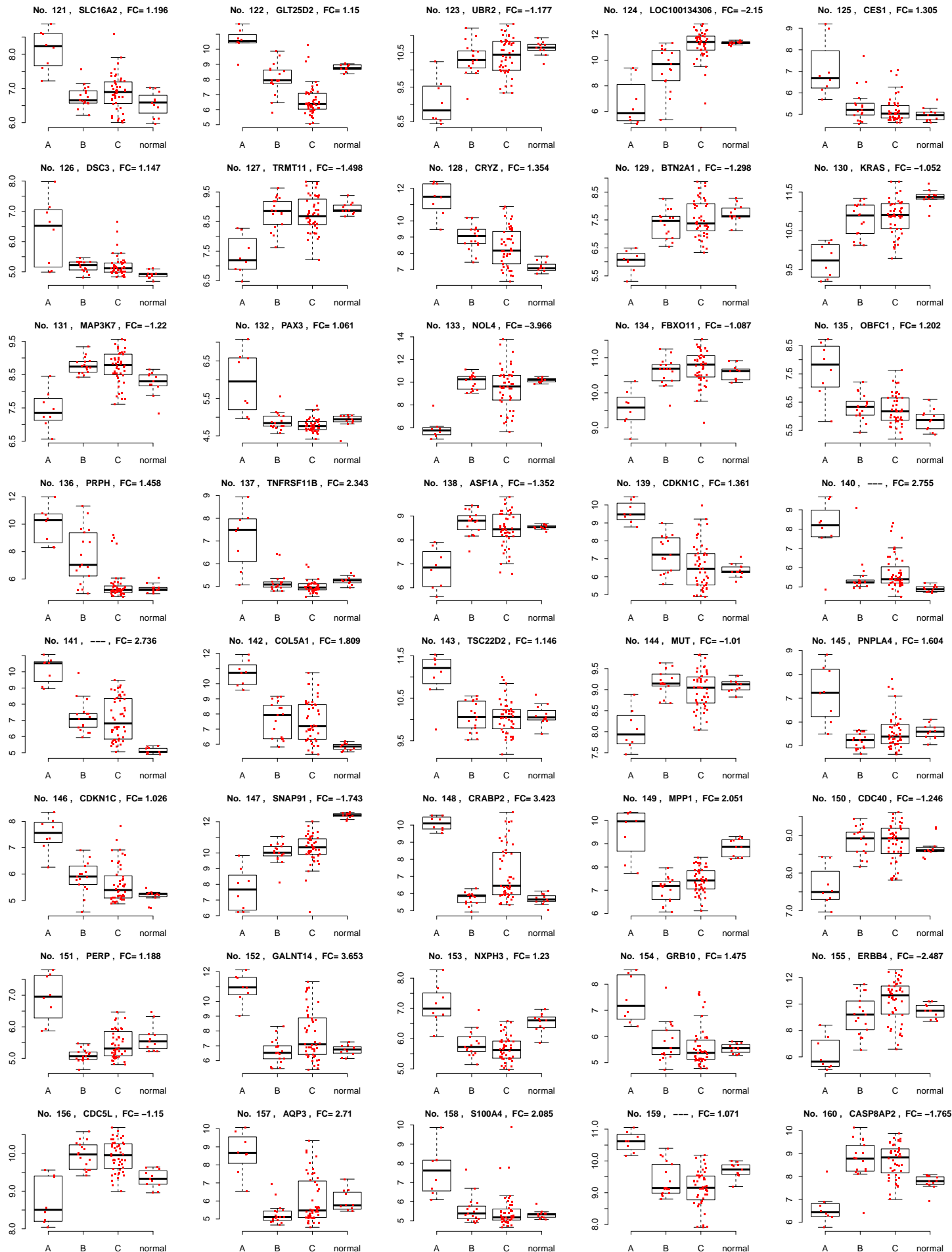

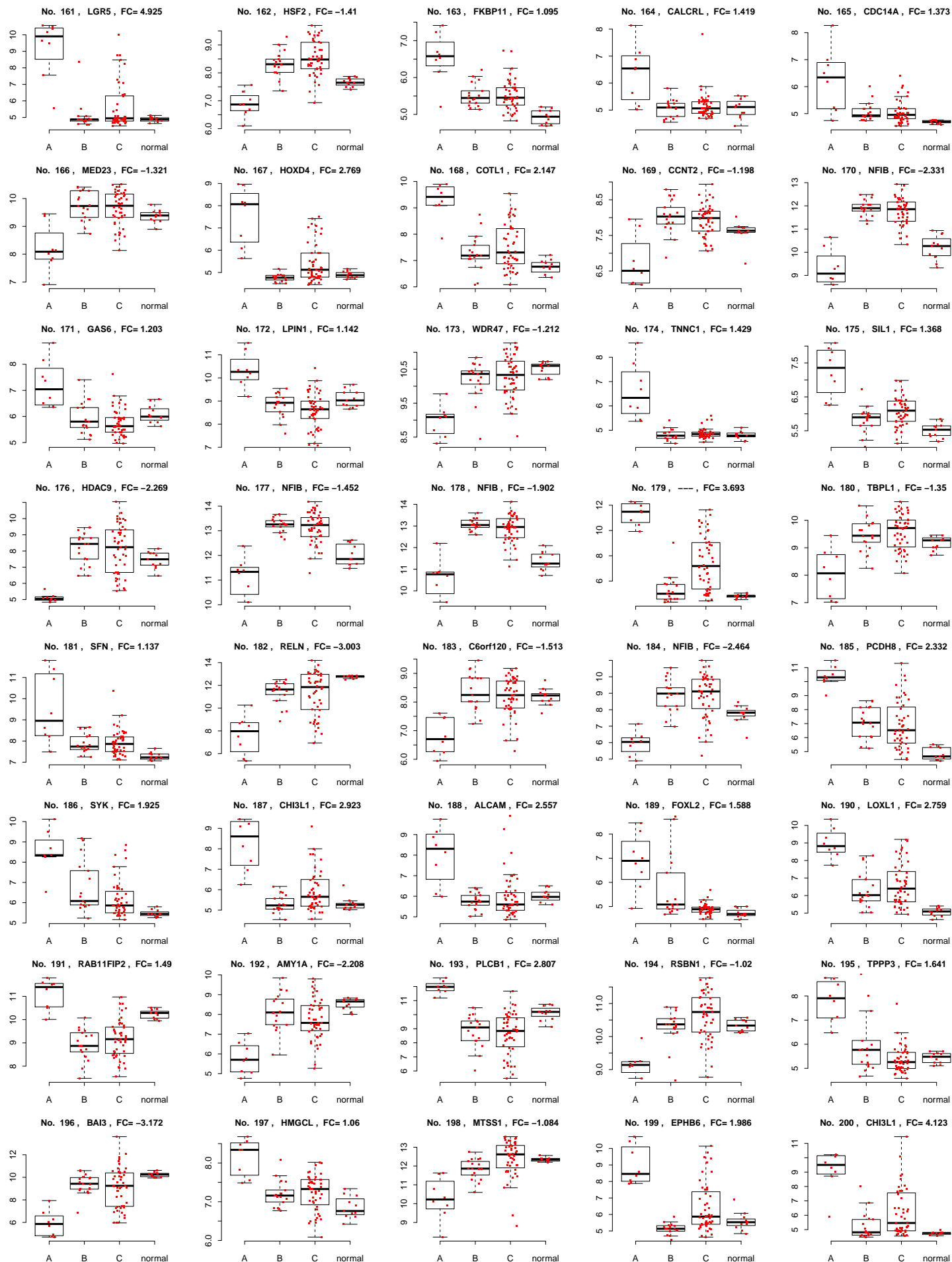

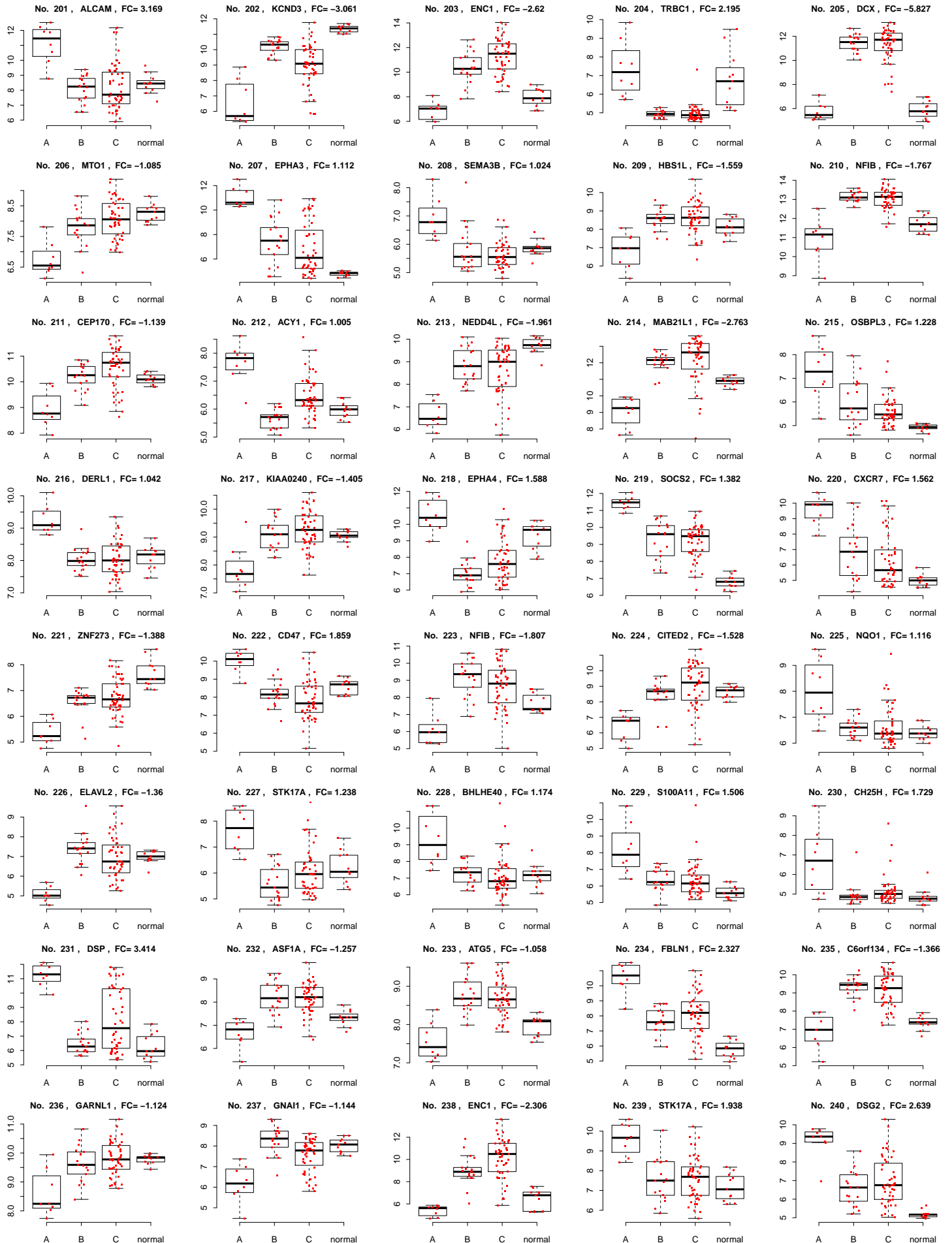

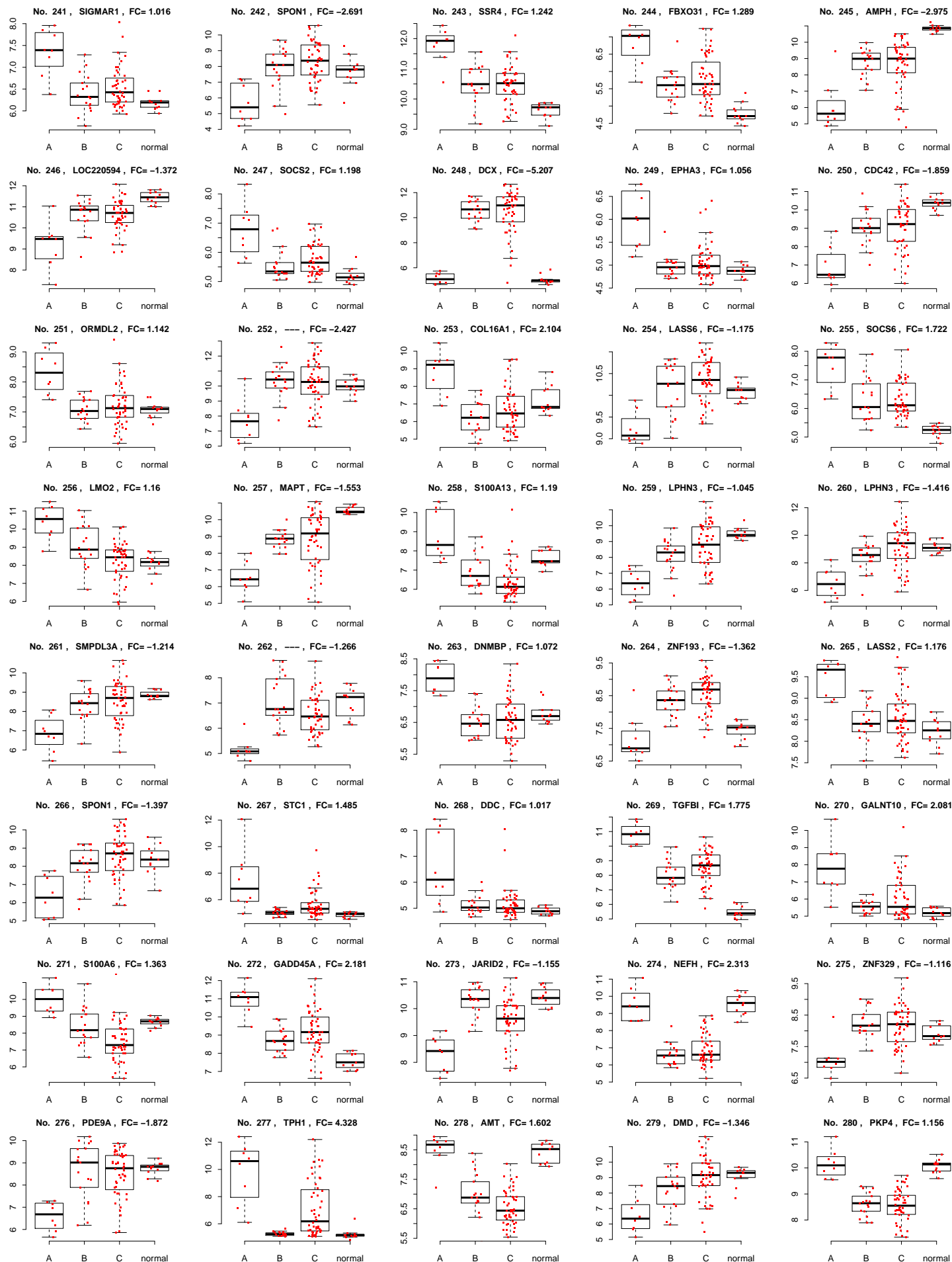

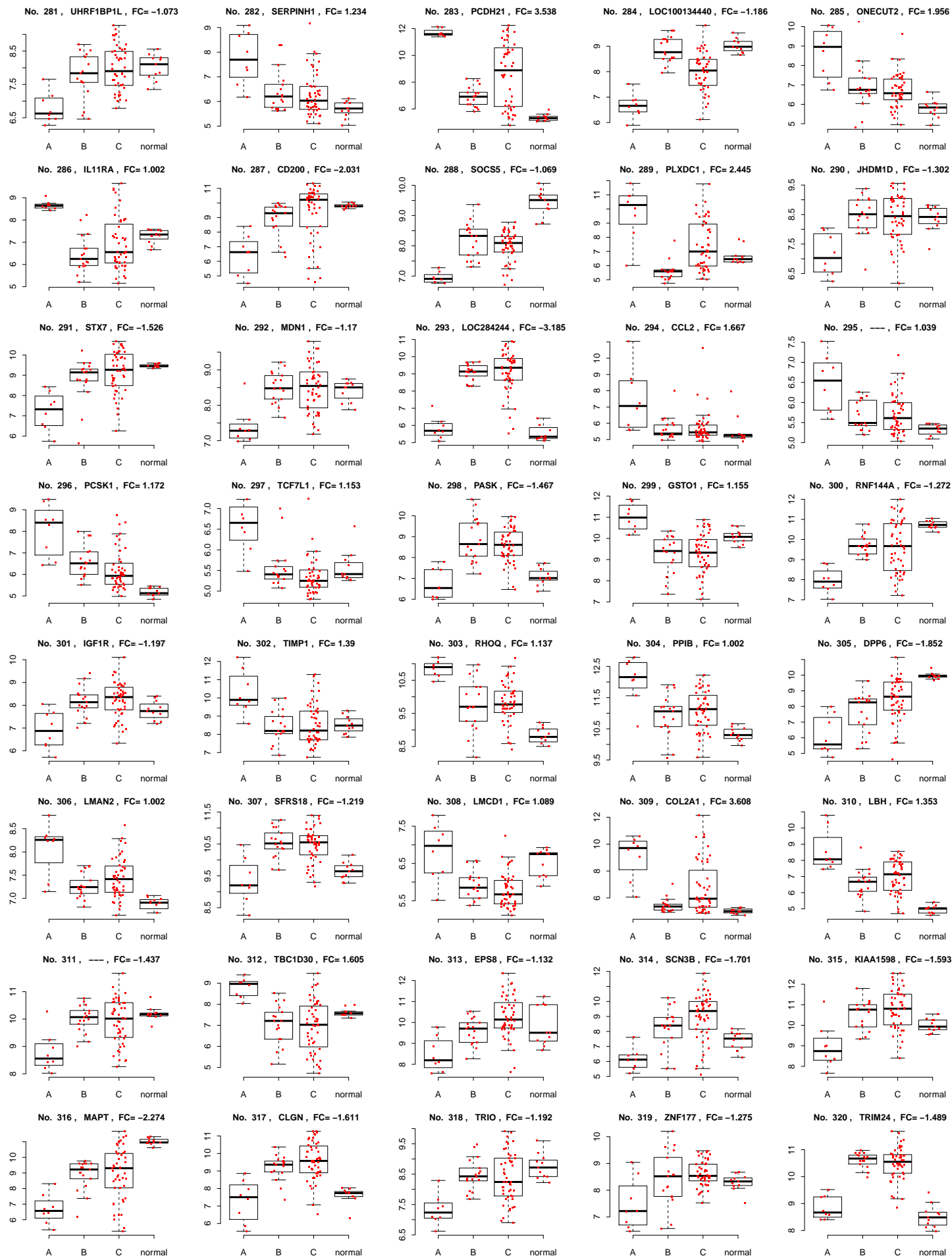

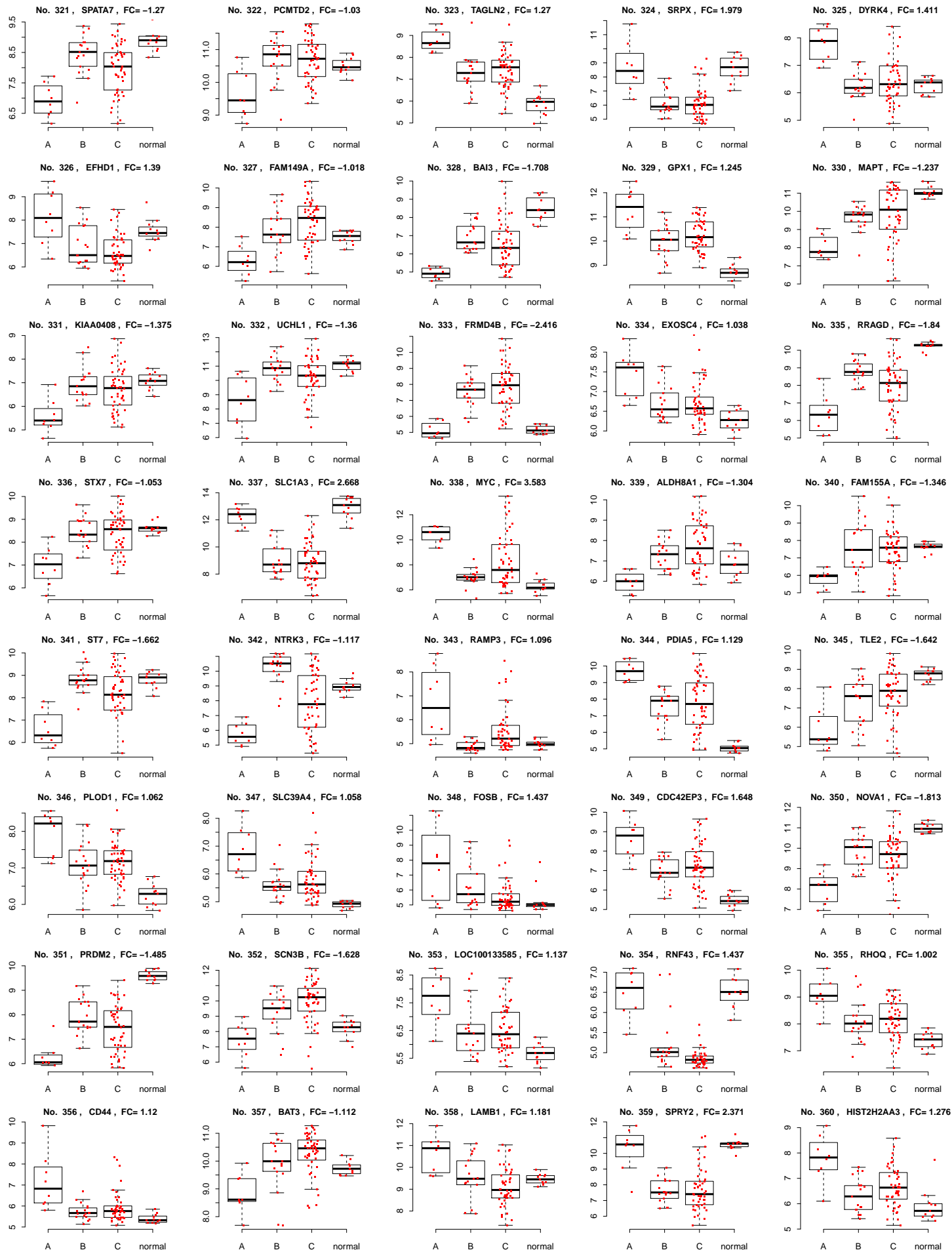

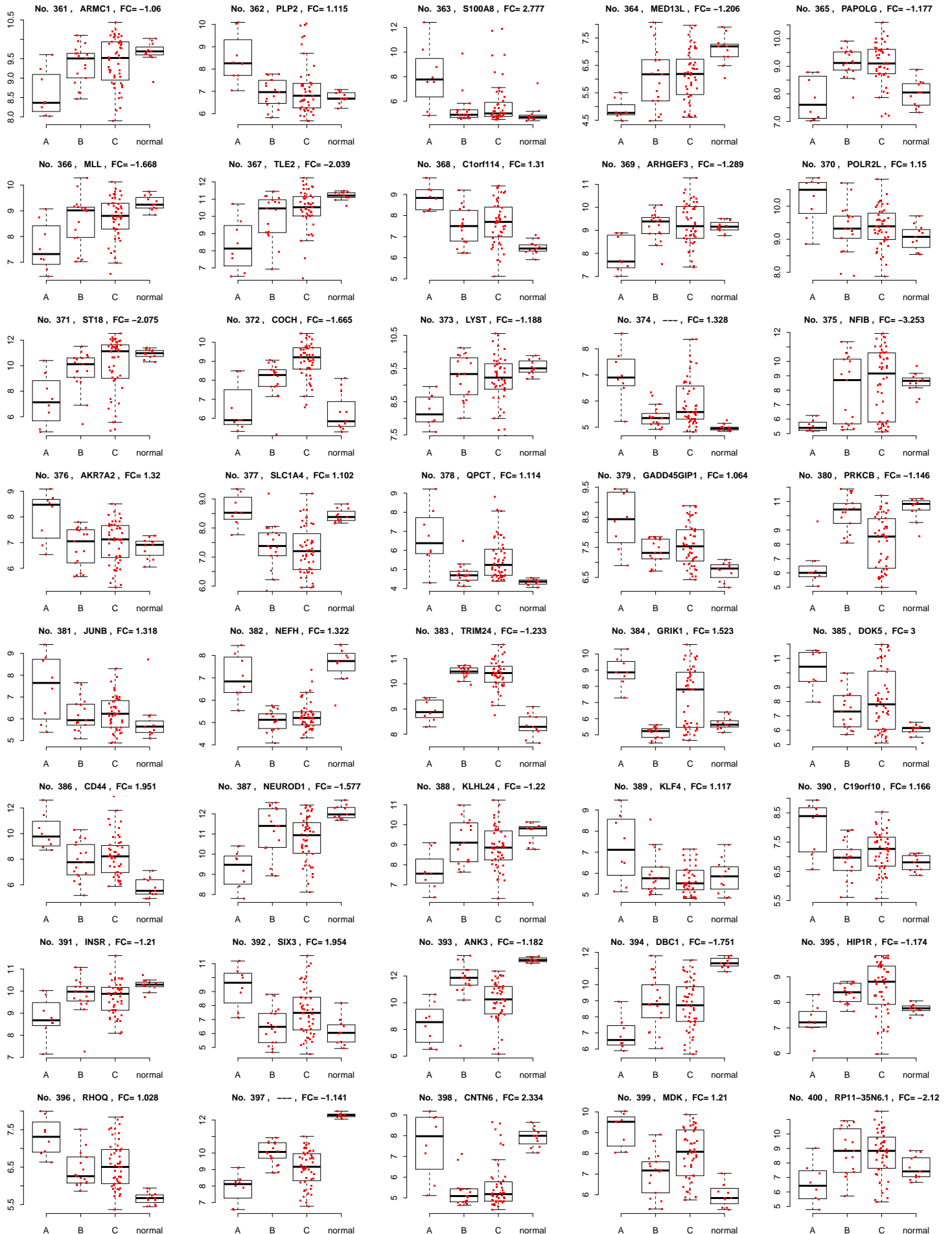

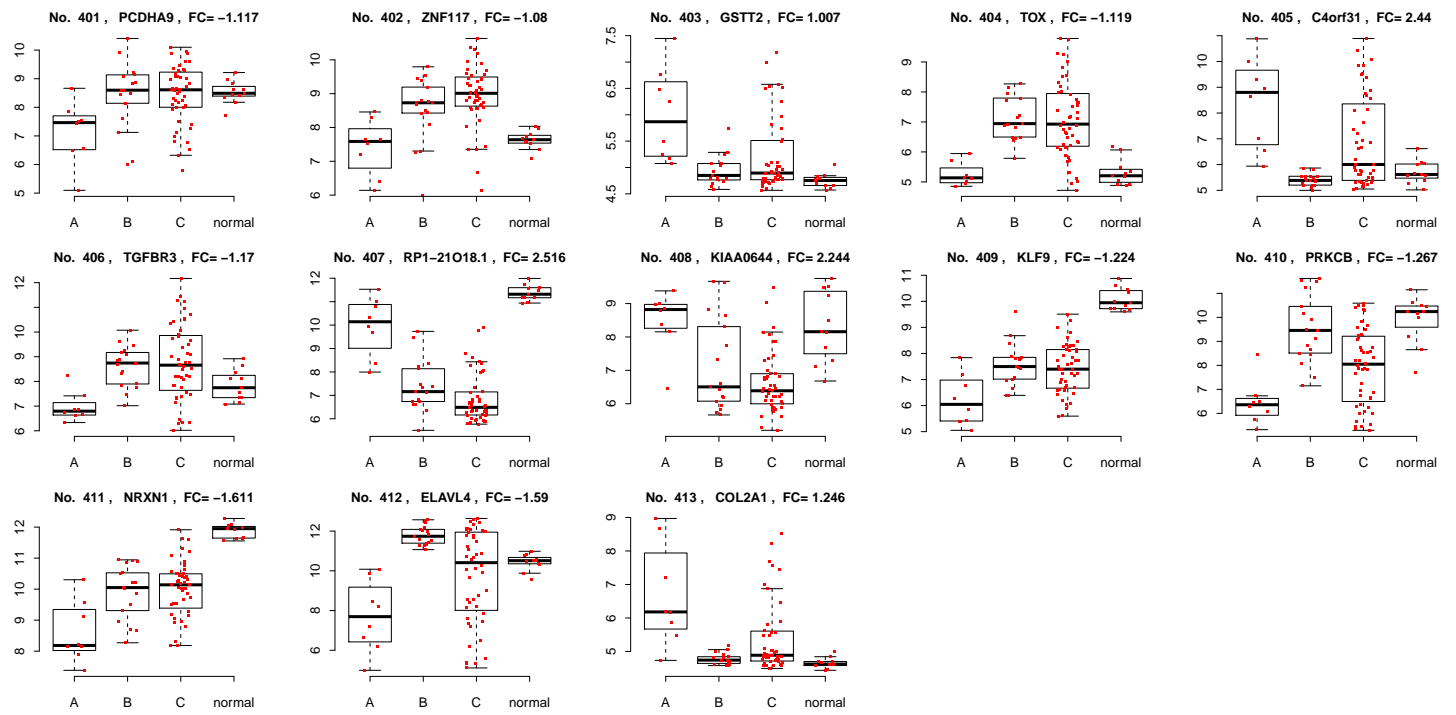

Supplement: Additional file 2 — The converged signatures for the subtypes of the three datasets. [file 1471-2105-14-S18-S1-S2.zip › plot-Cho73-SubtypeA.pdf]

# Kool62

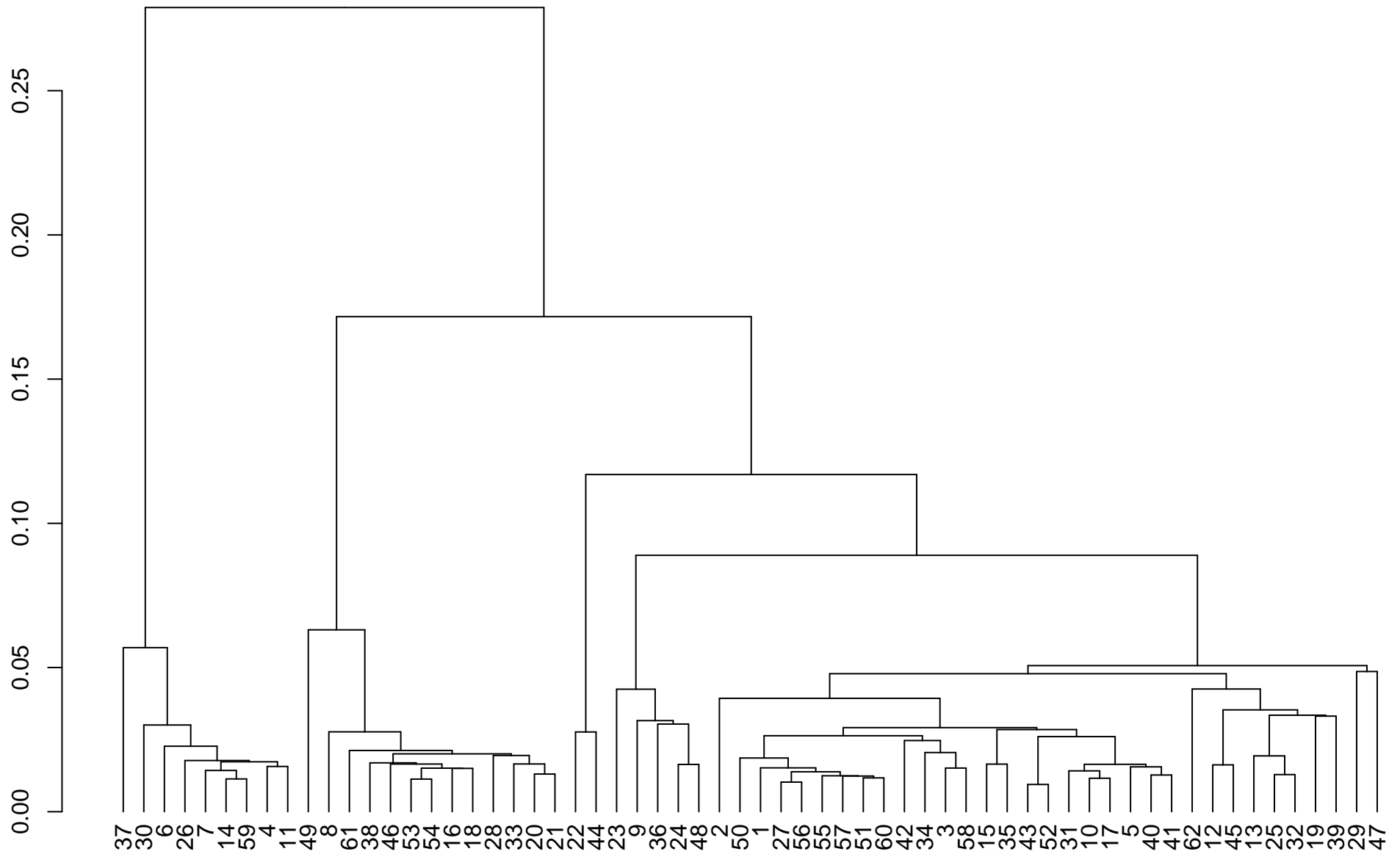

Supplement: Additional file 2 — The converged signatures for the subtypes of the three datasets. [file 1471-2105-14-S18-S1-S2.zip › convergeMap-Kool62-dendrogram.pdf]

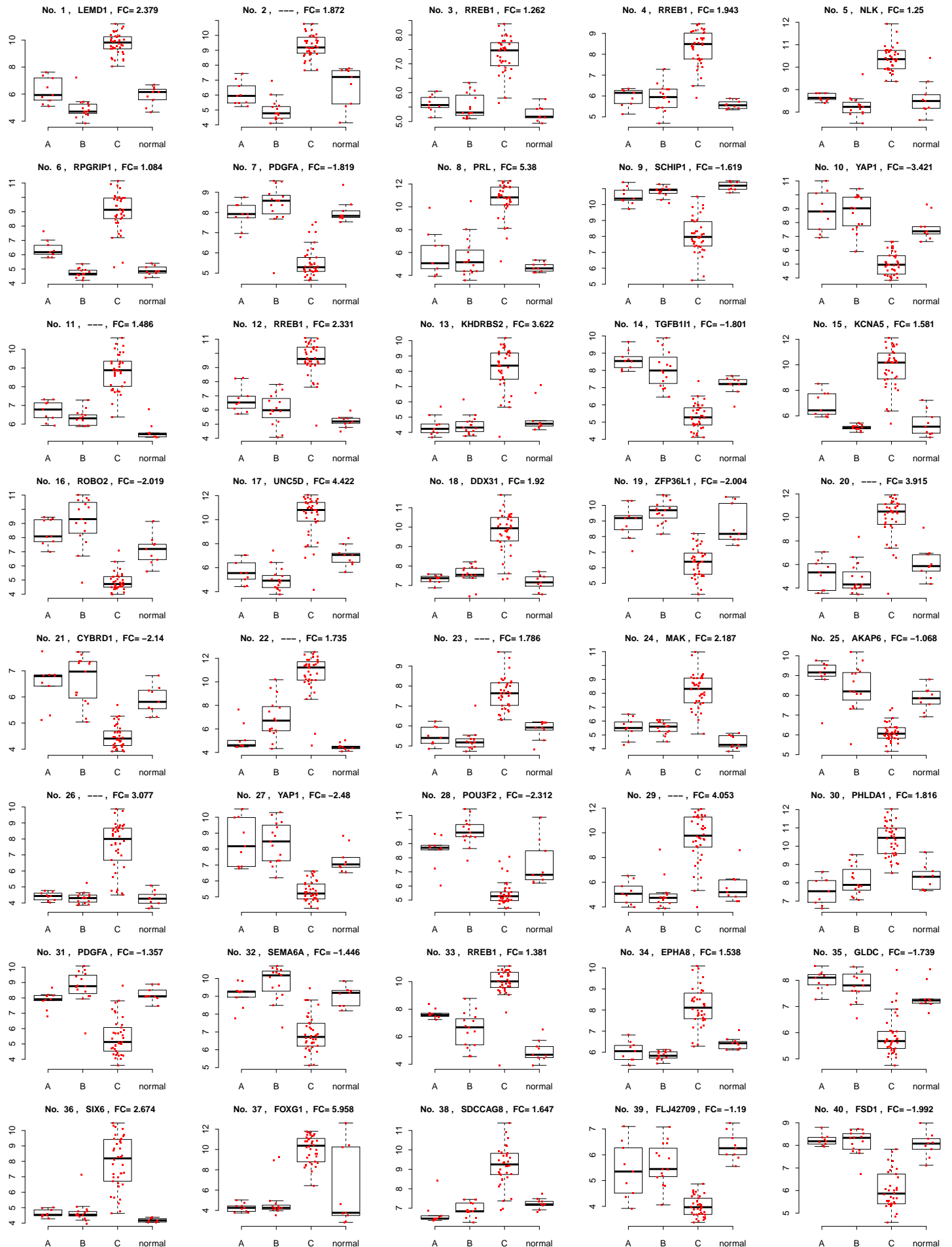

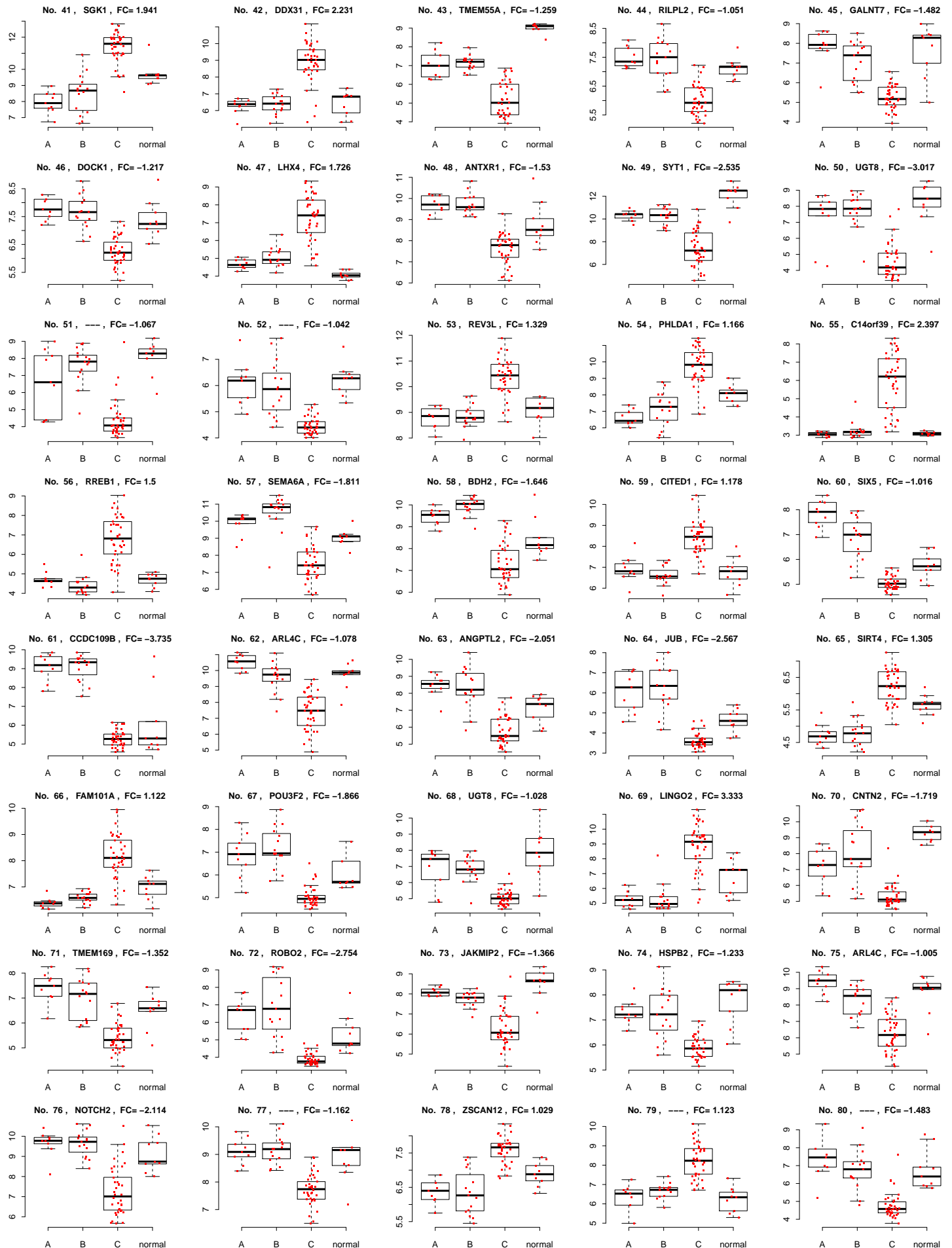

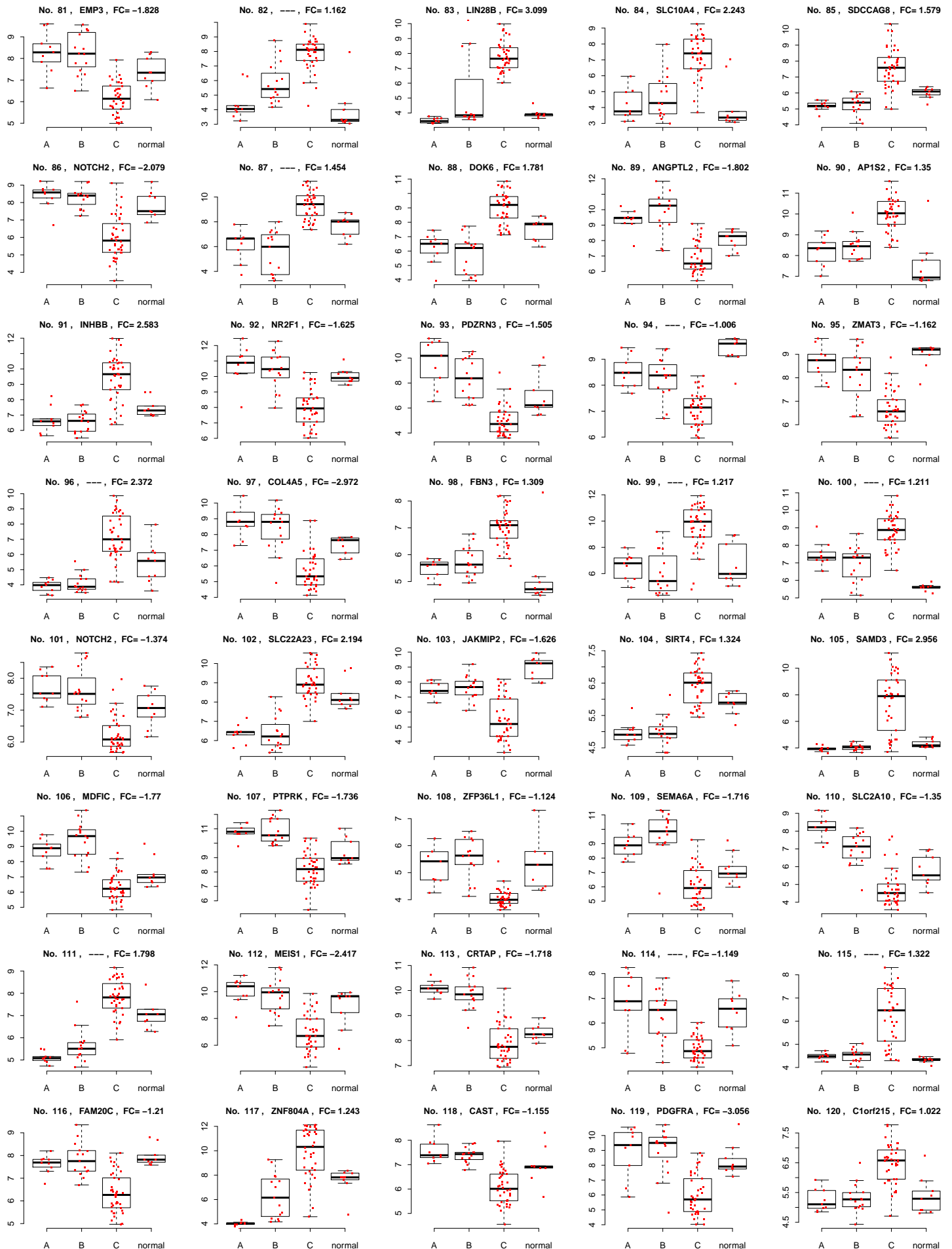

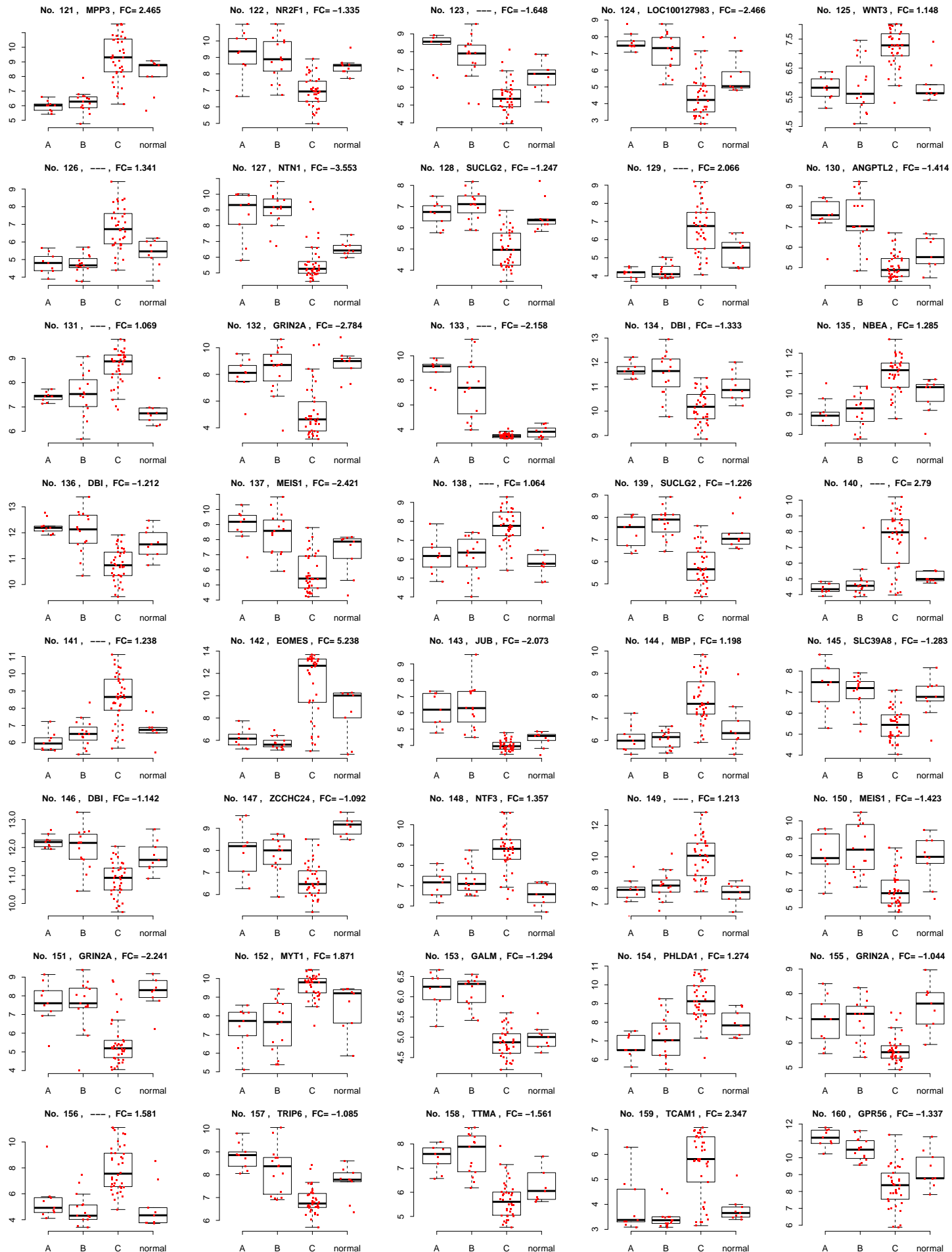

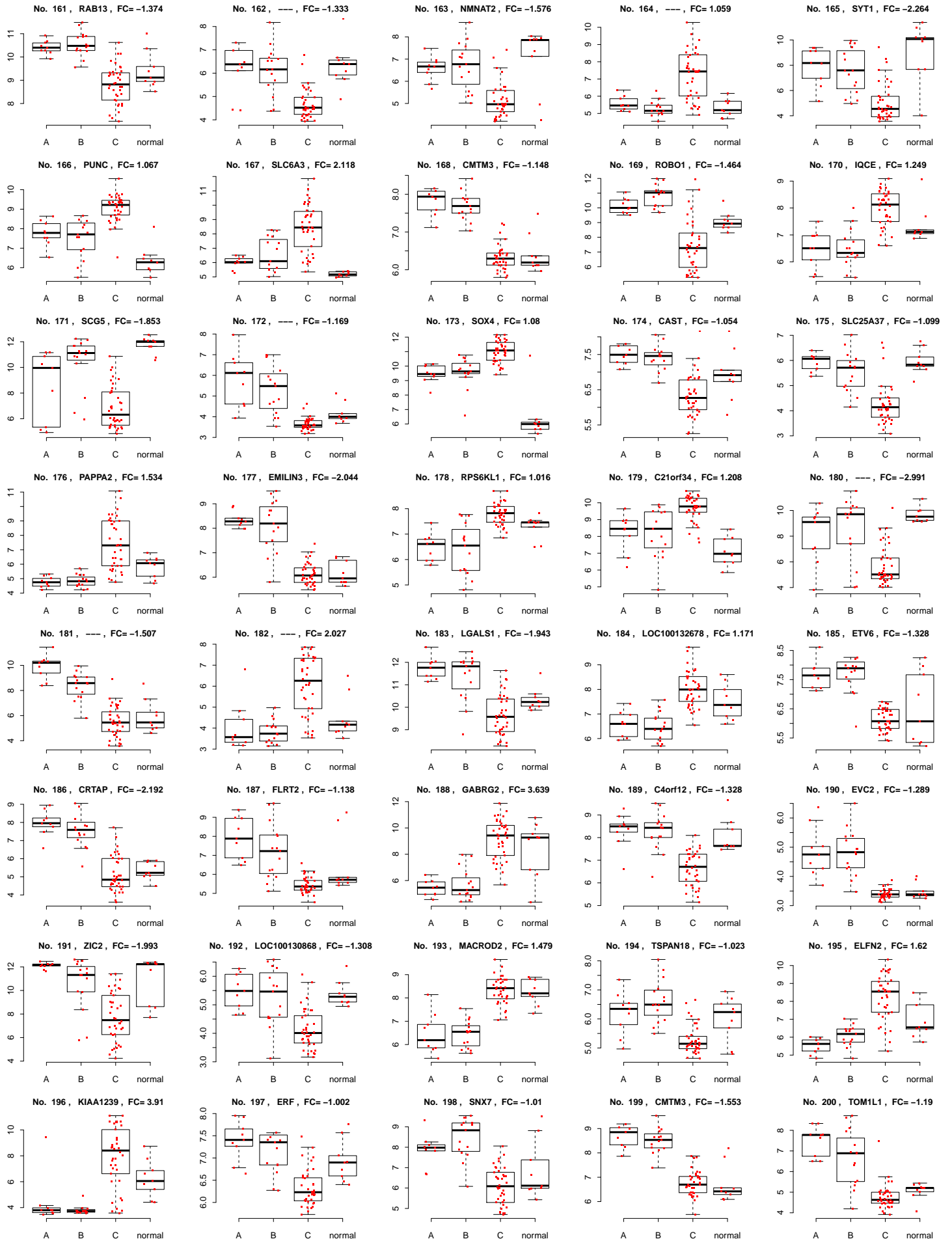

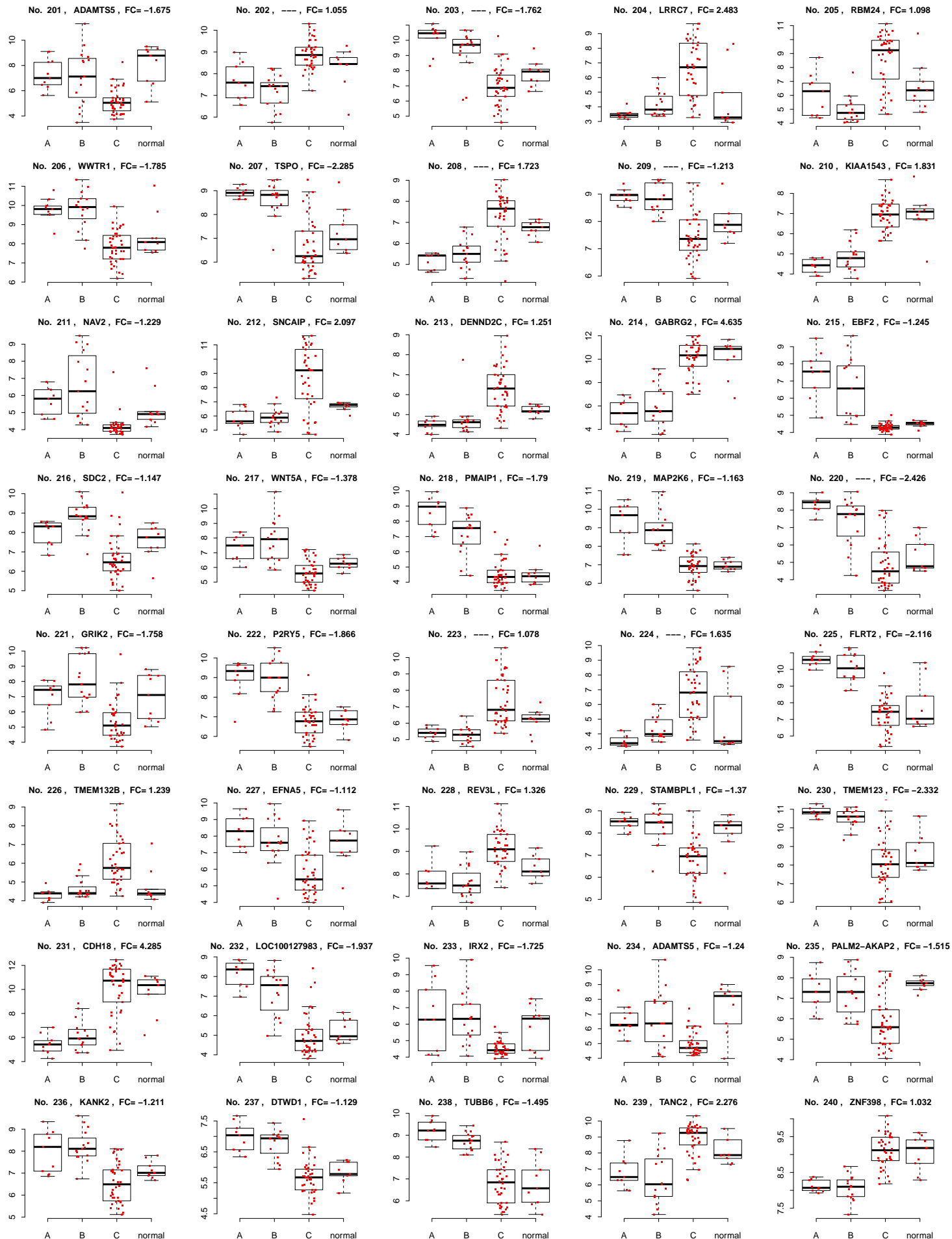

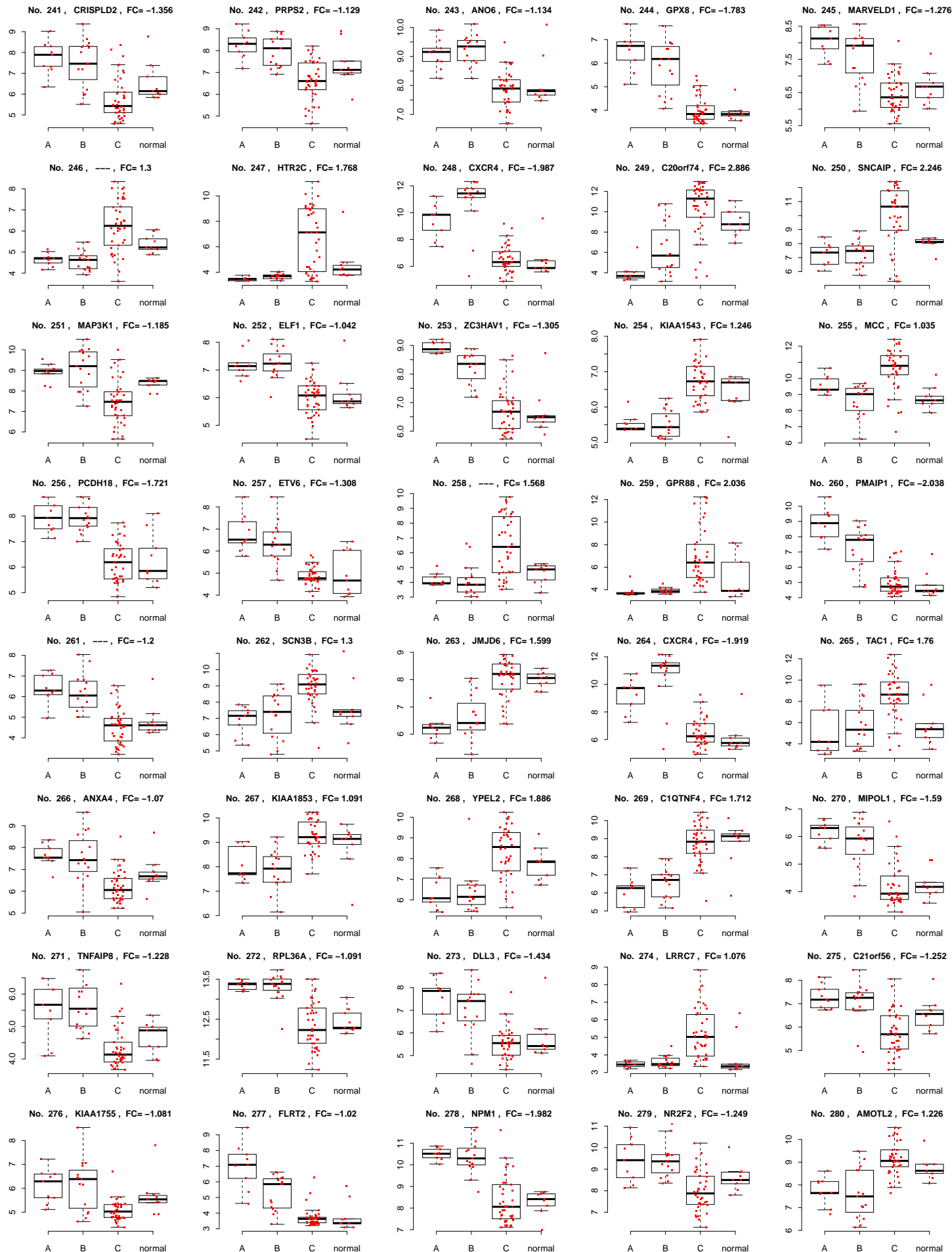

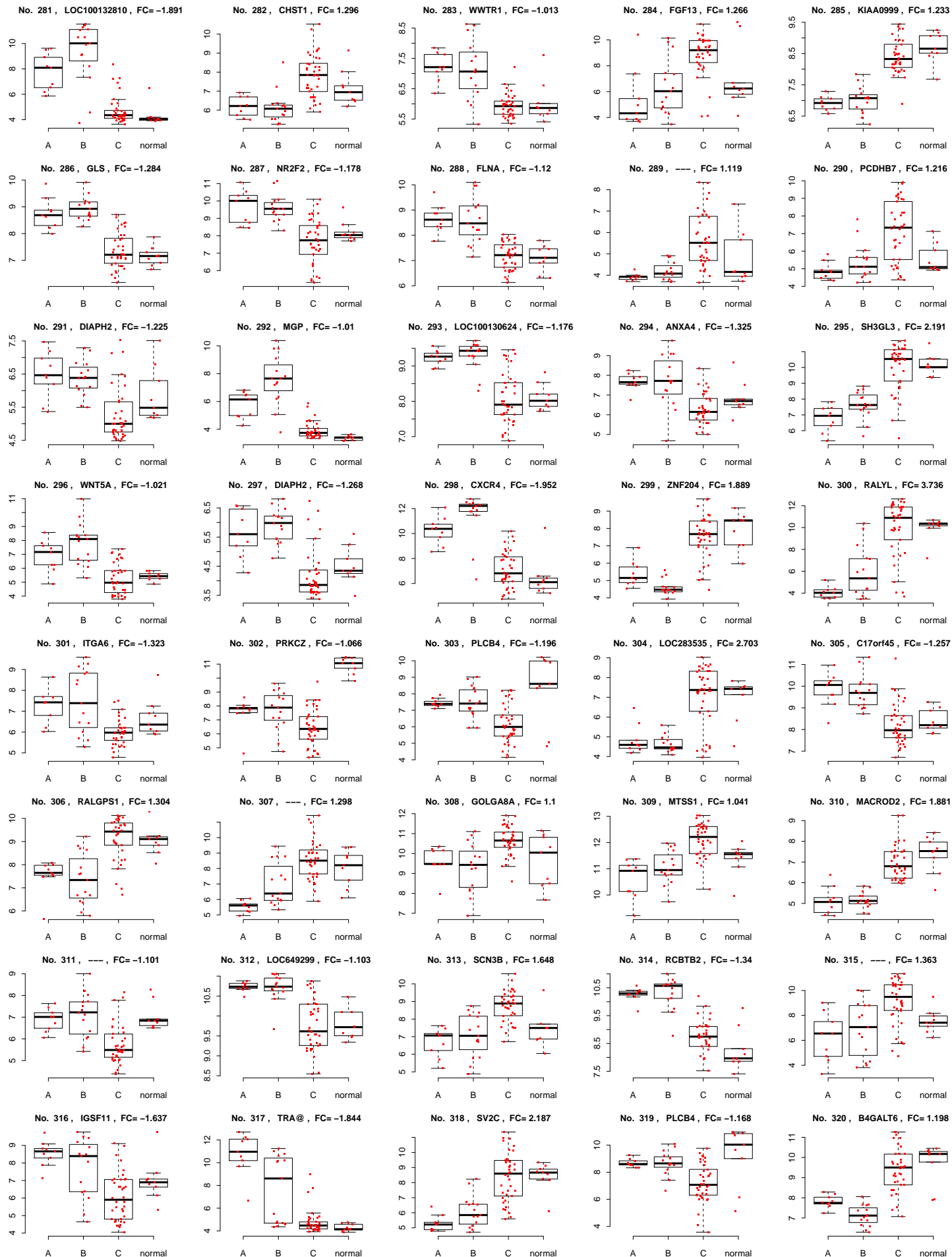

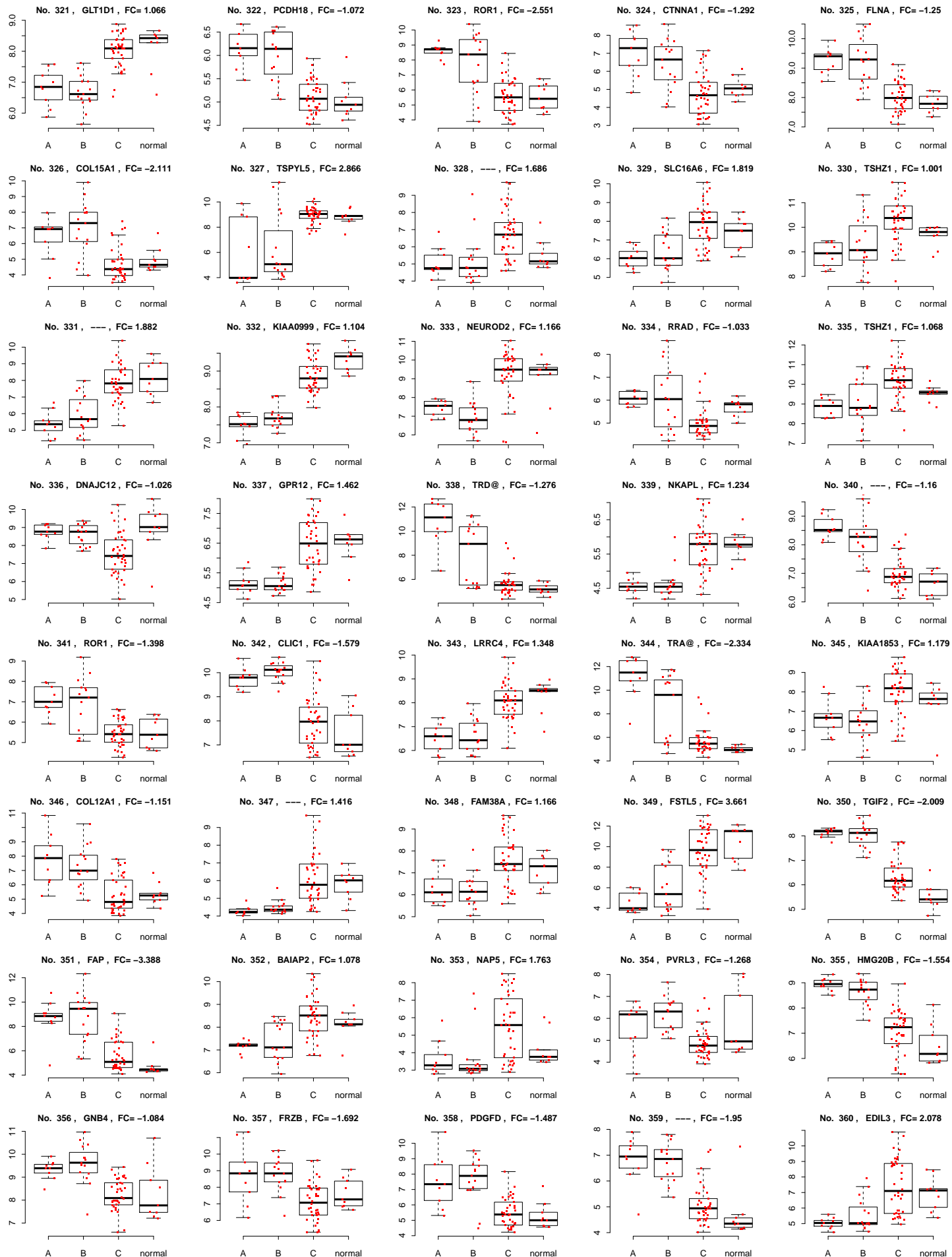

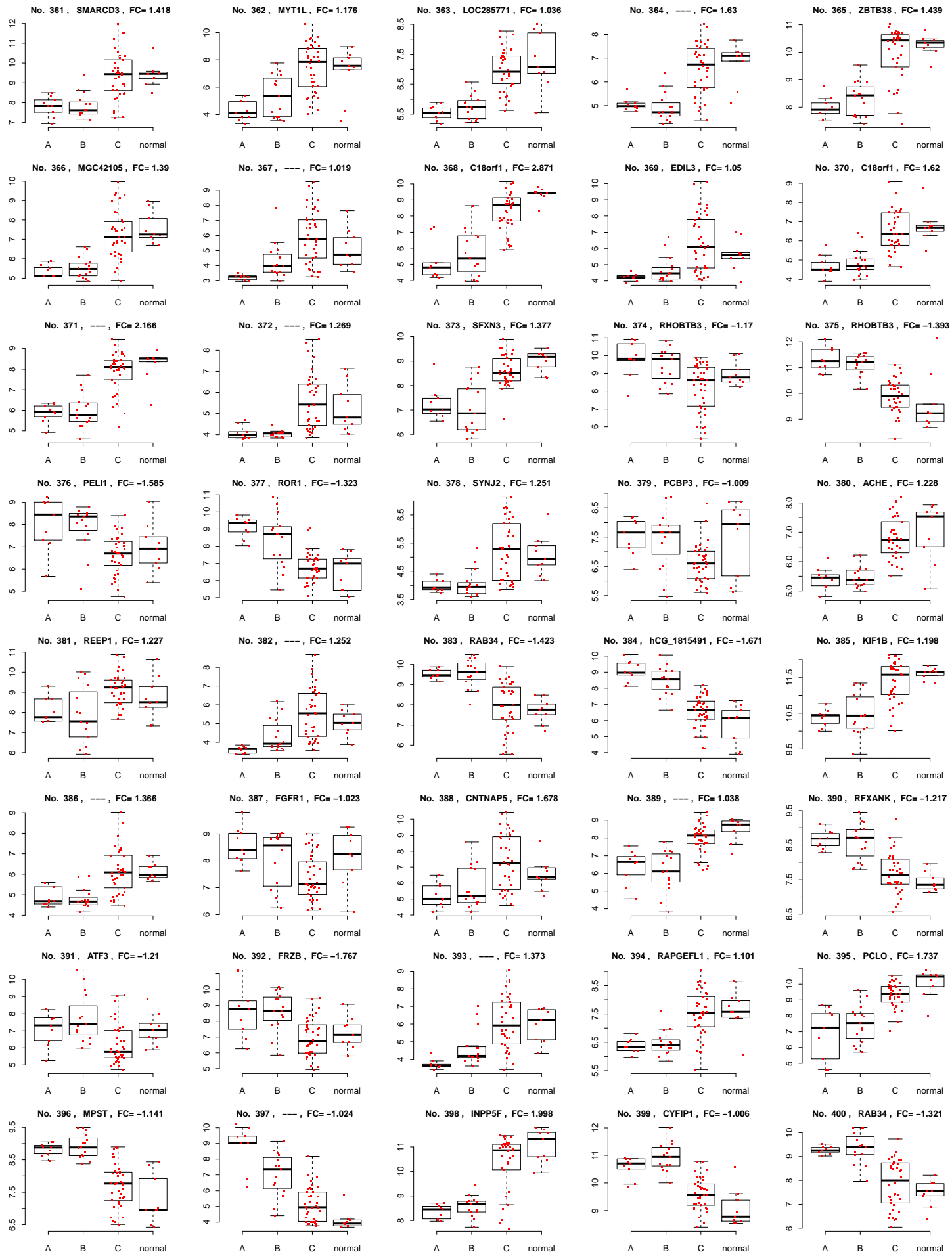

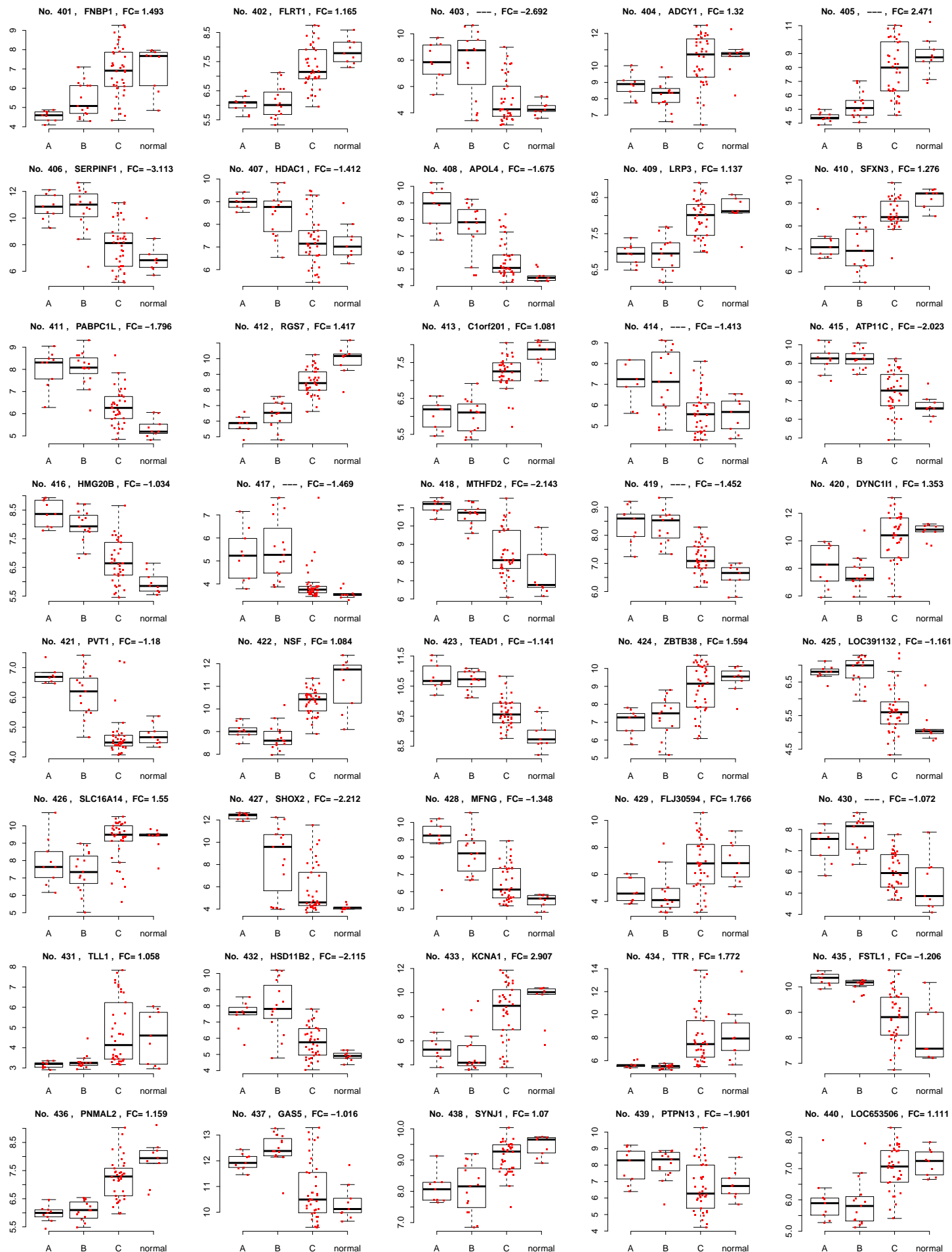

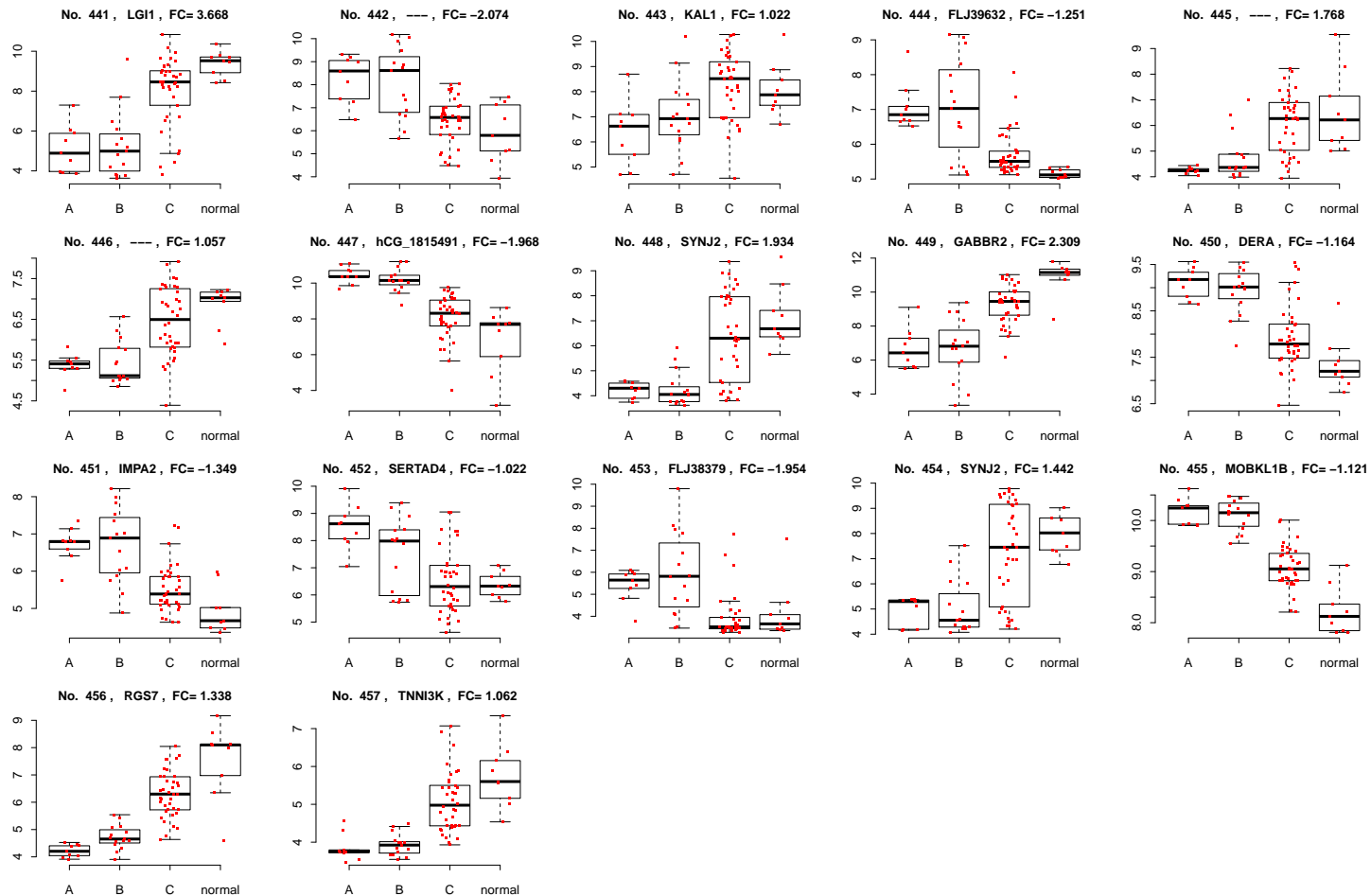

Supplement: Additional file 2 — The converged signatures for the subtypes of the three datasets. [file 1471-2105-14-S18-S1-S2.zip › plot-Kool62-SubtypeC.pdf]

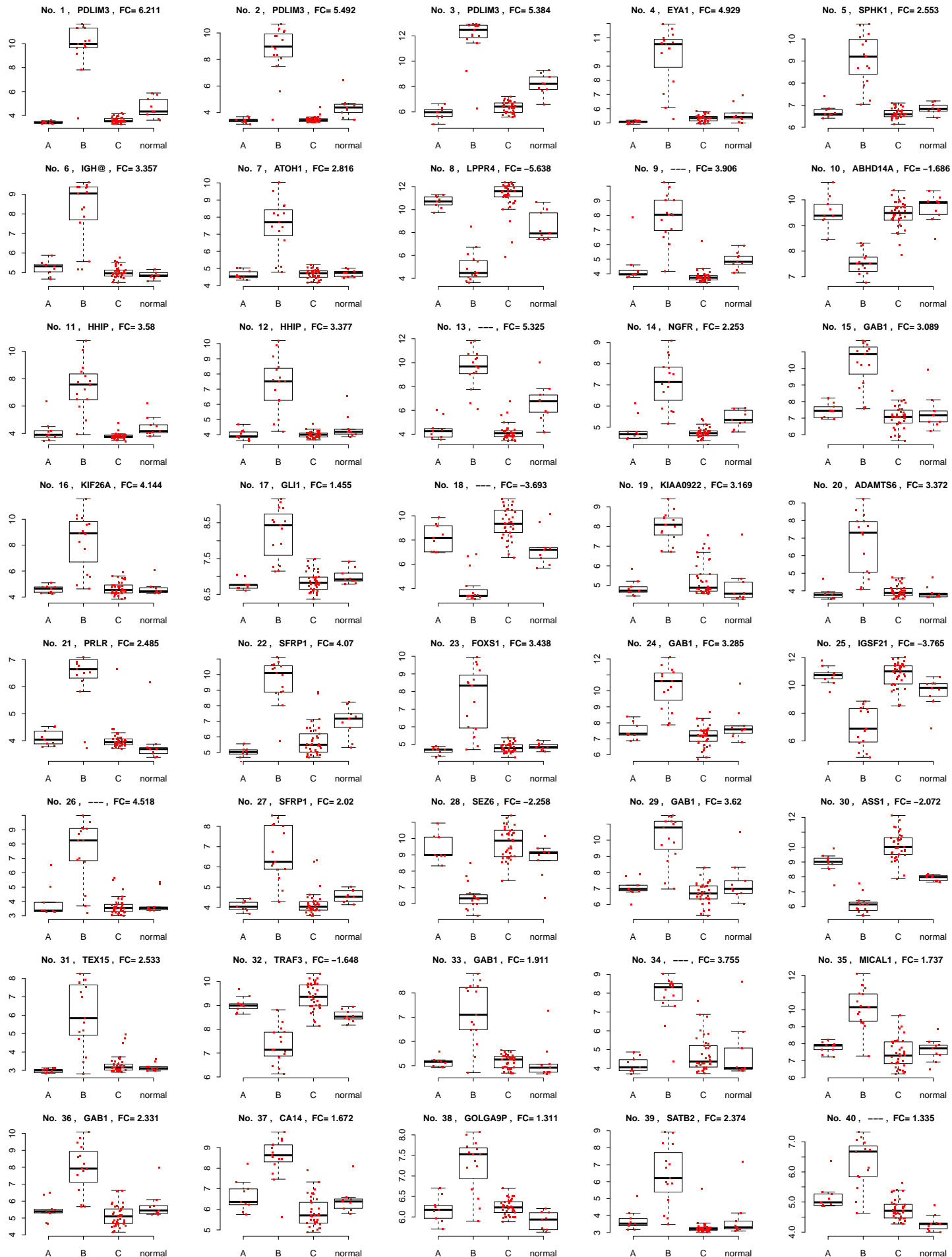

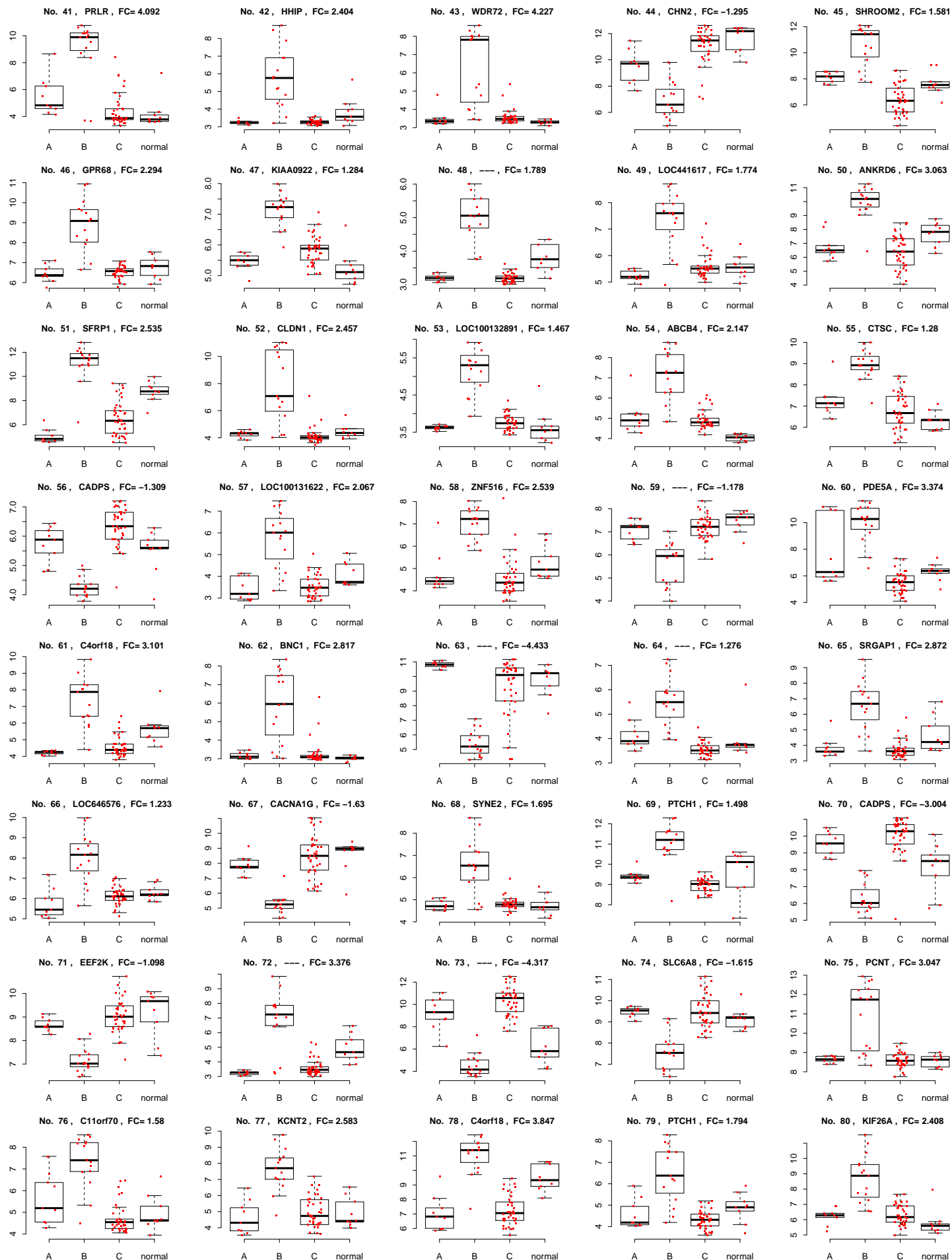

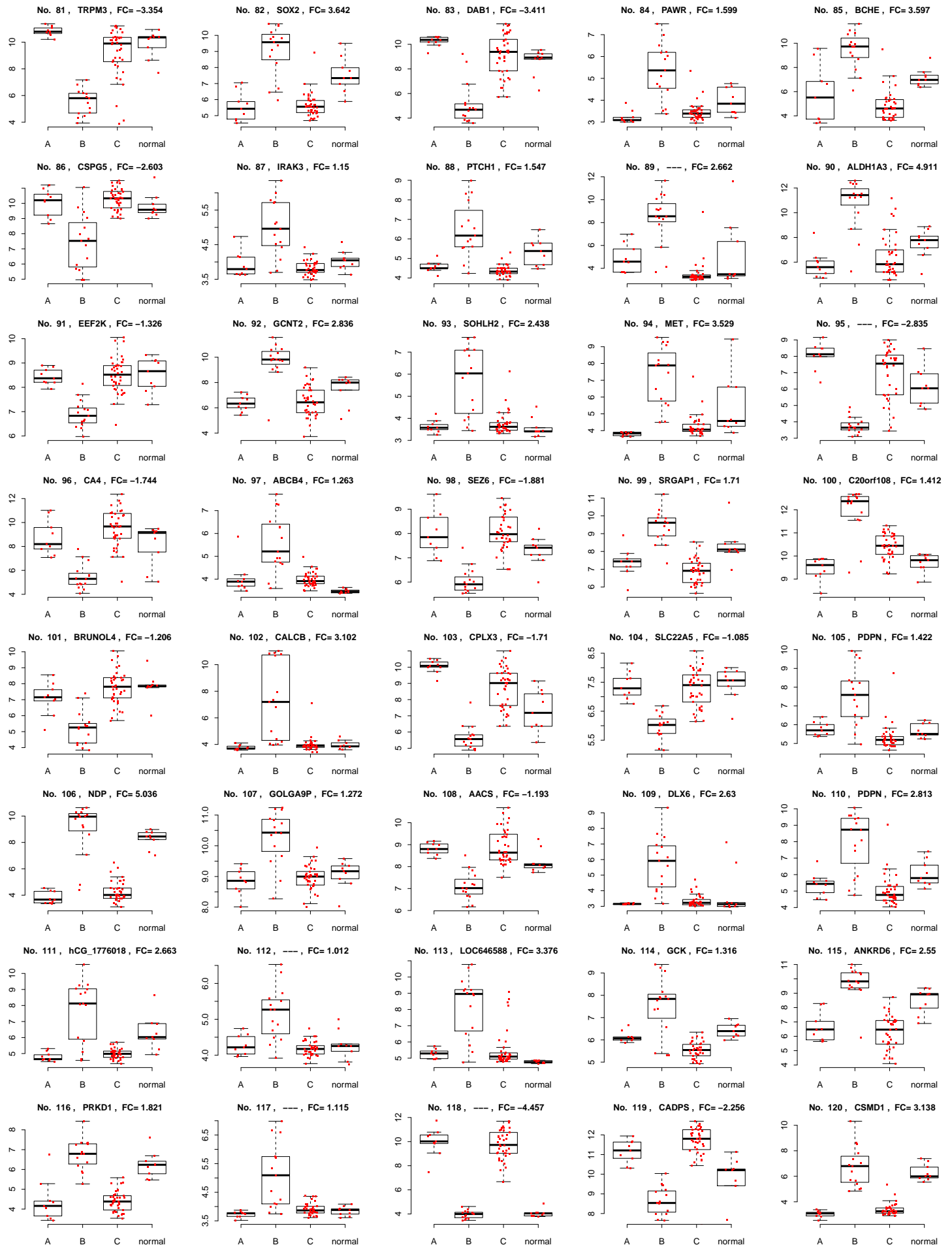

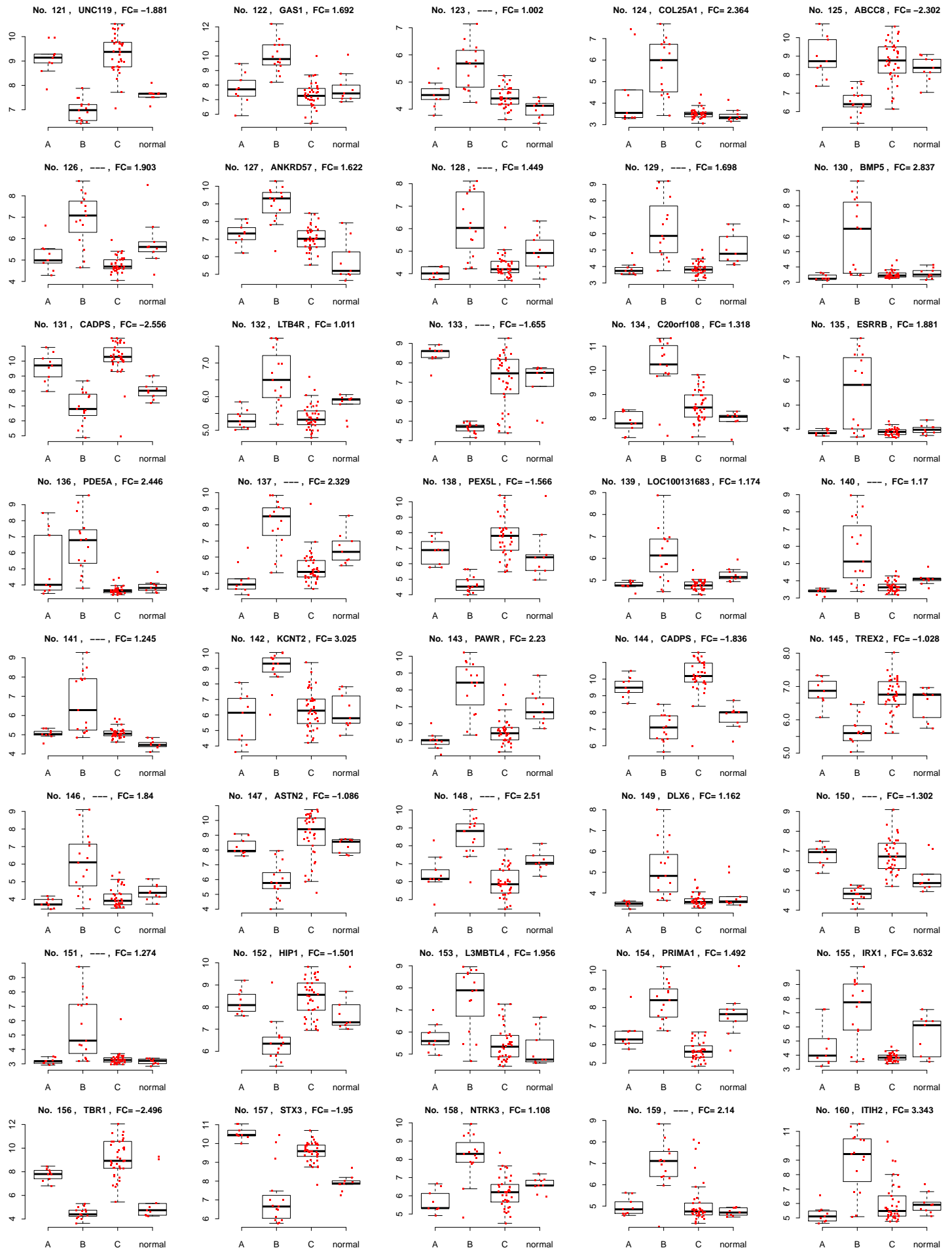

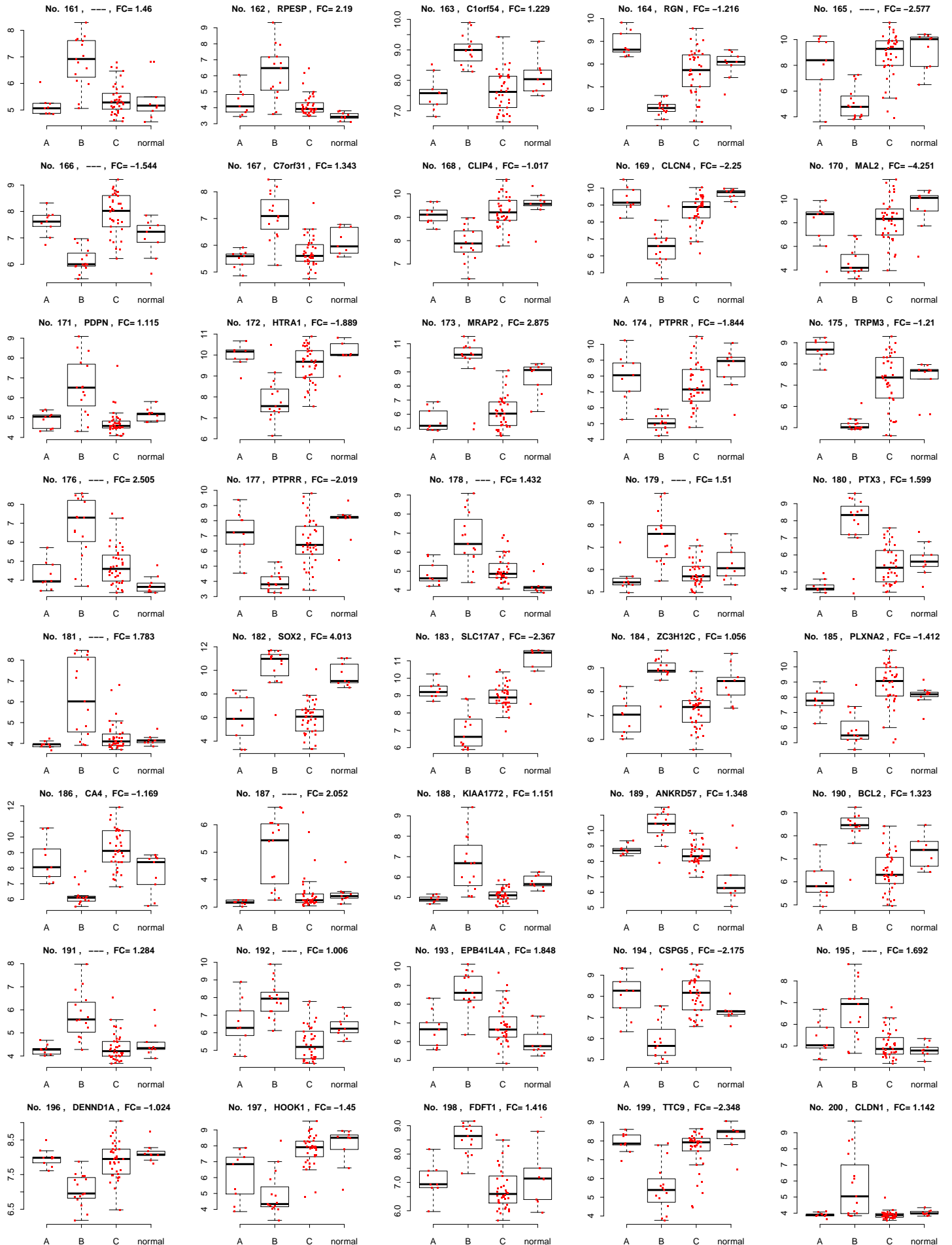

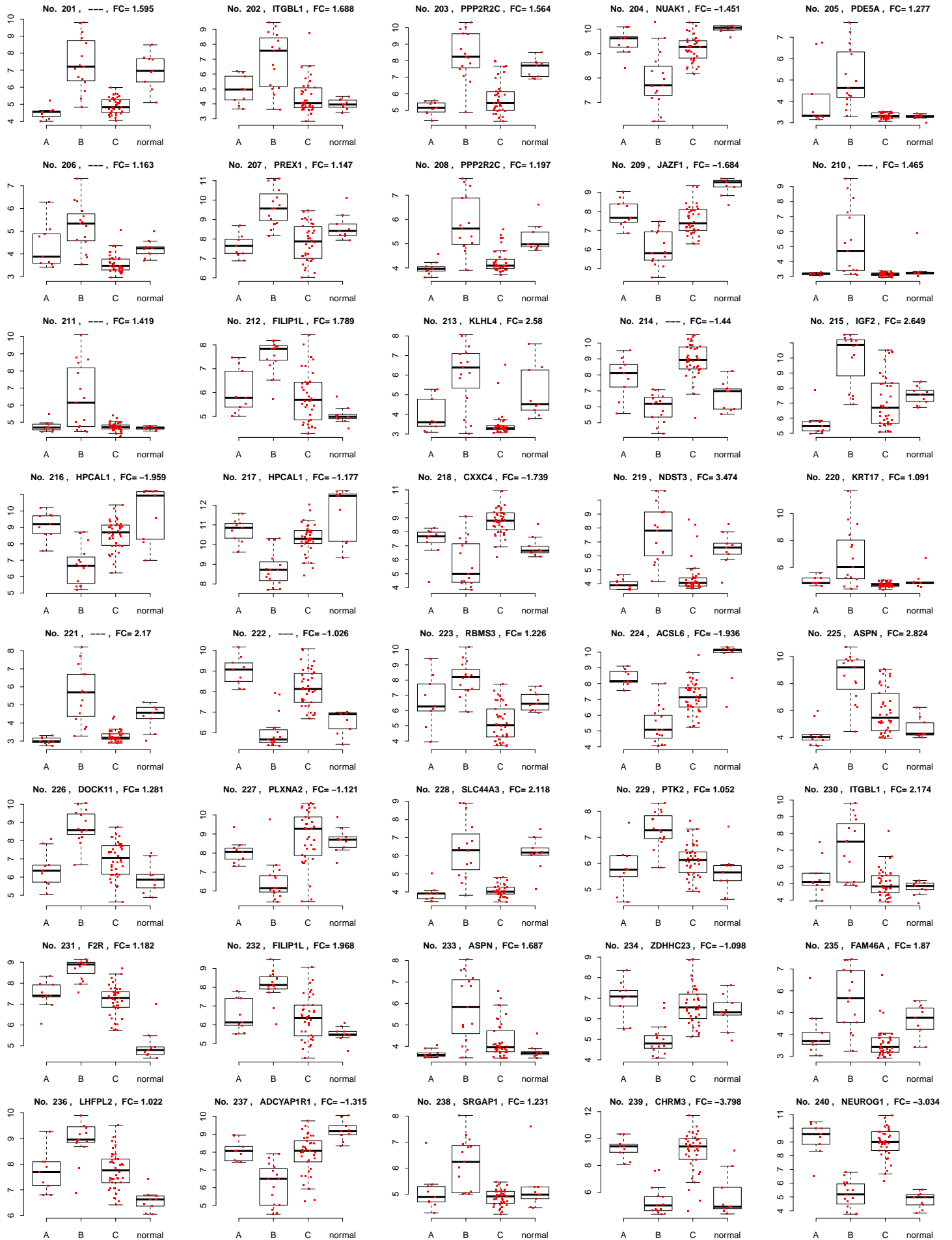

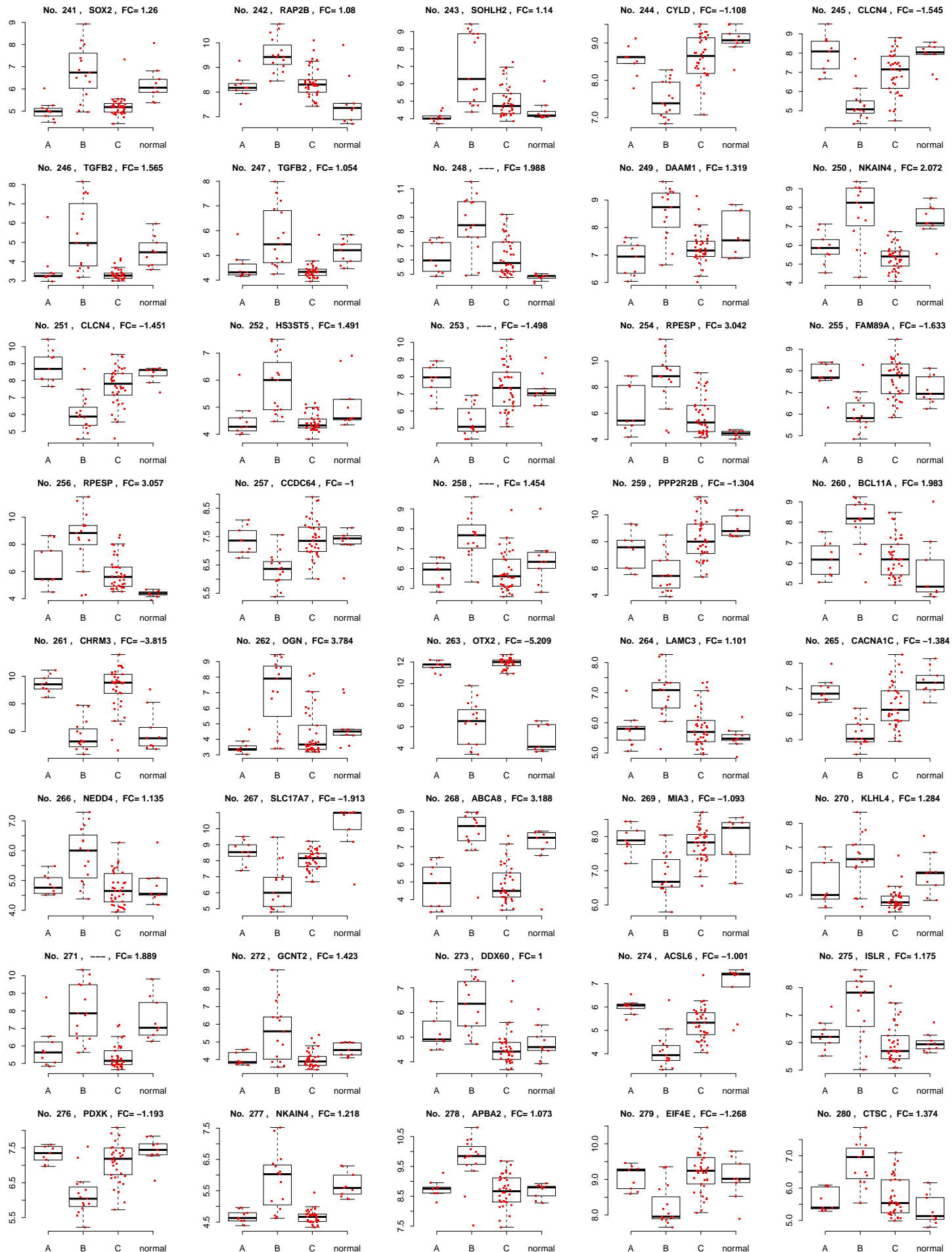

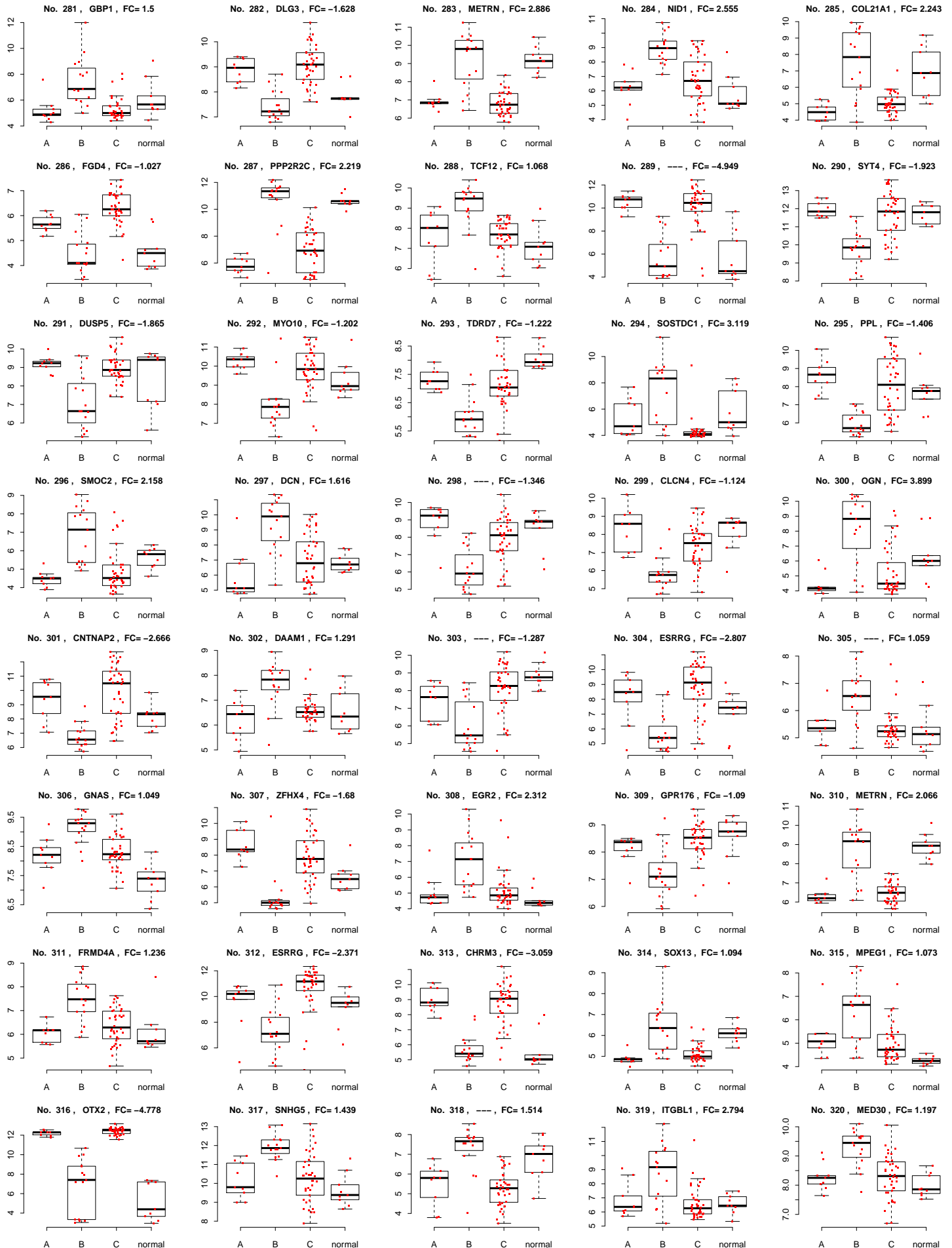

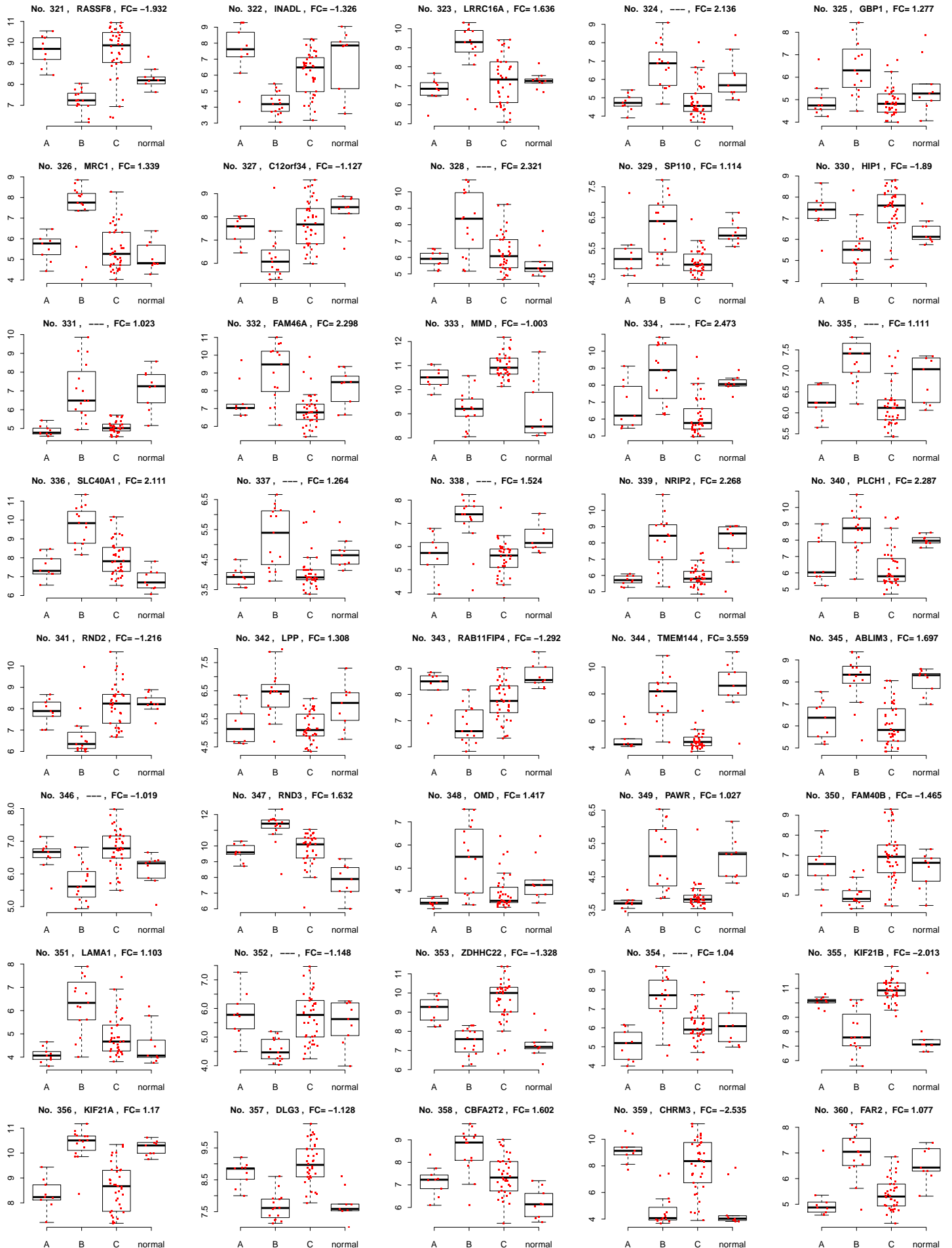

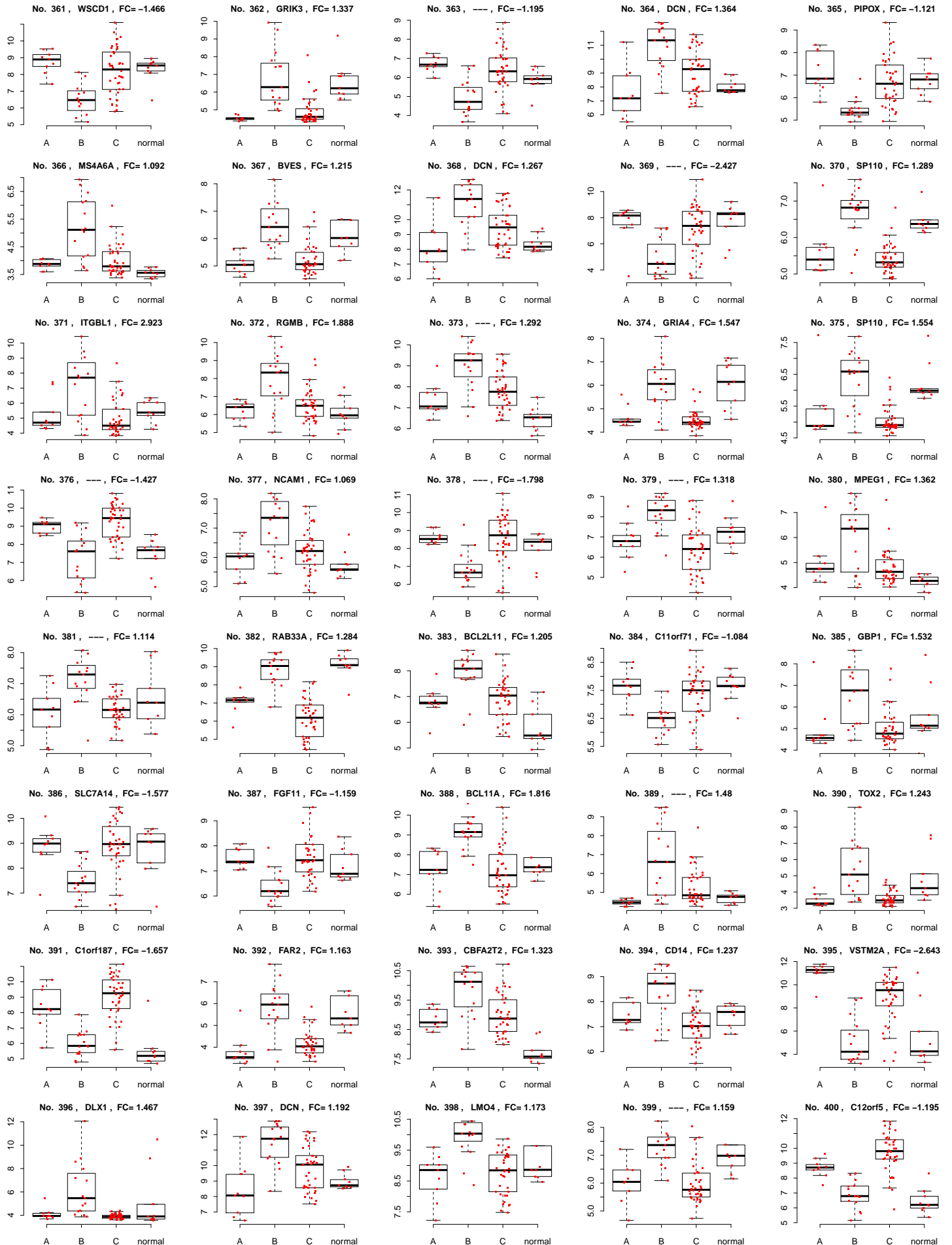

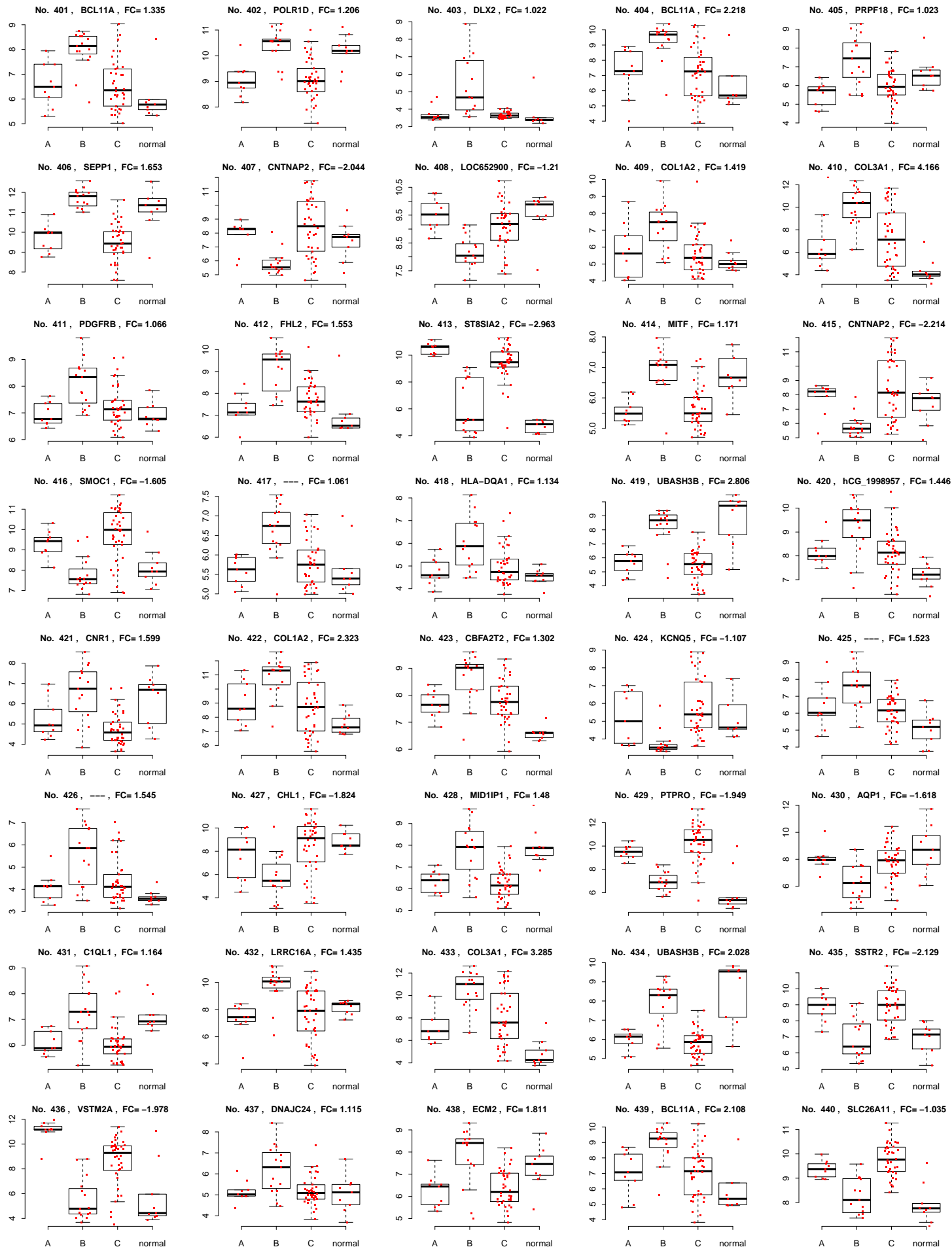

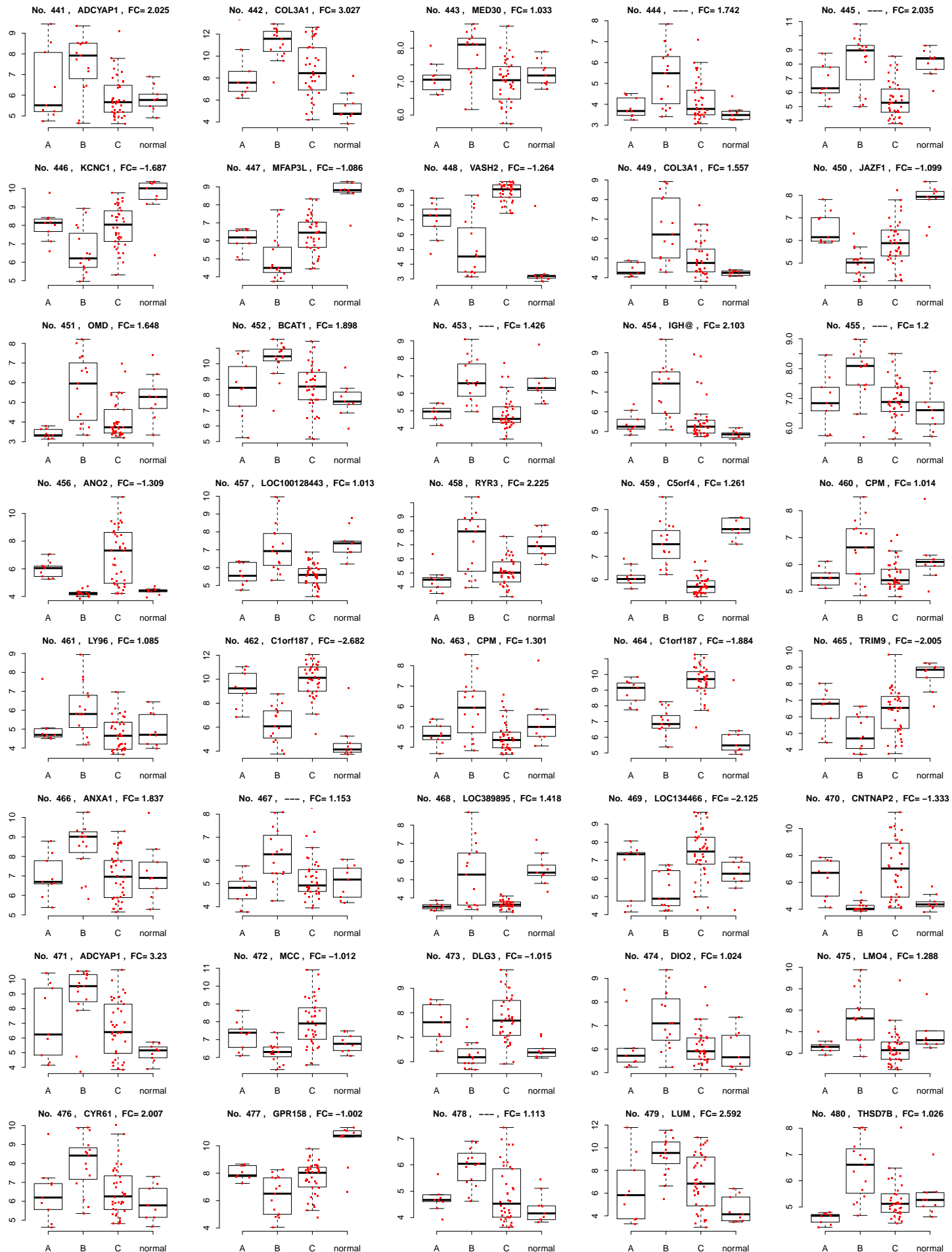

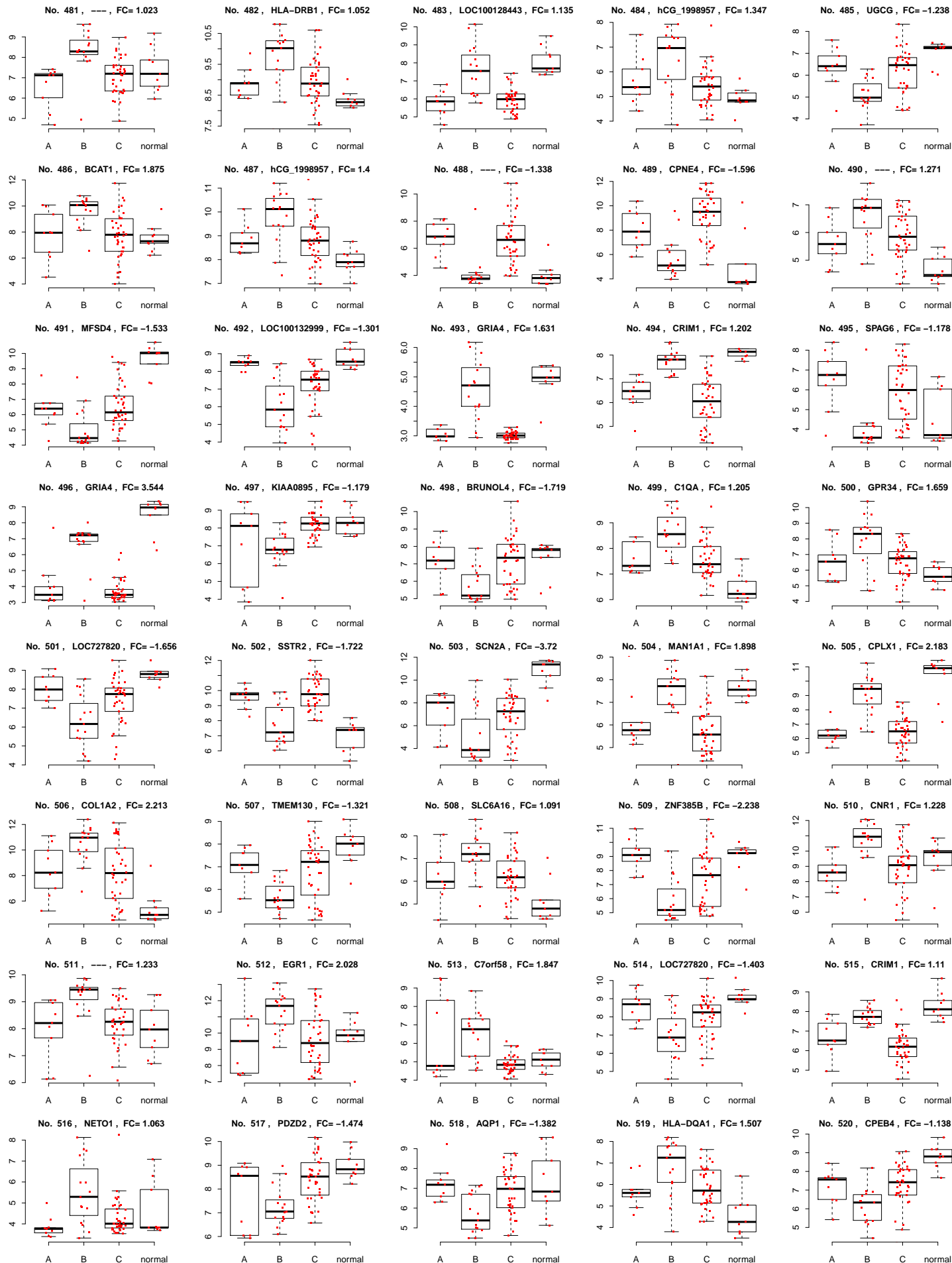

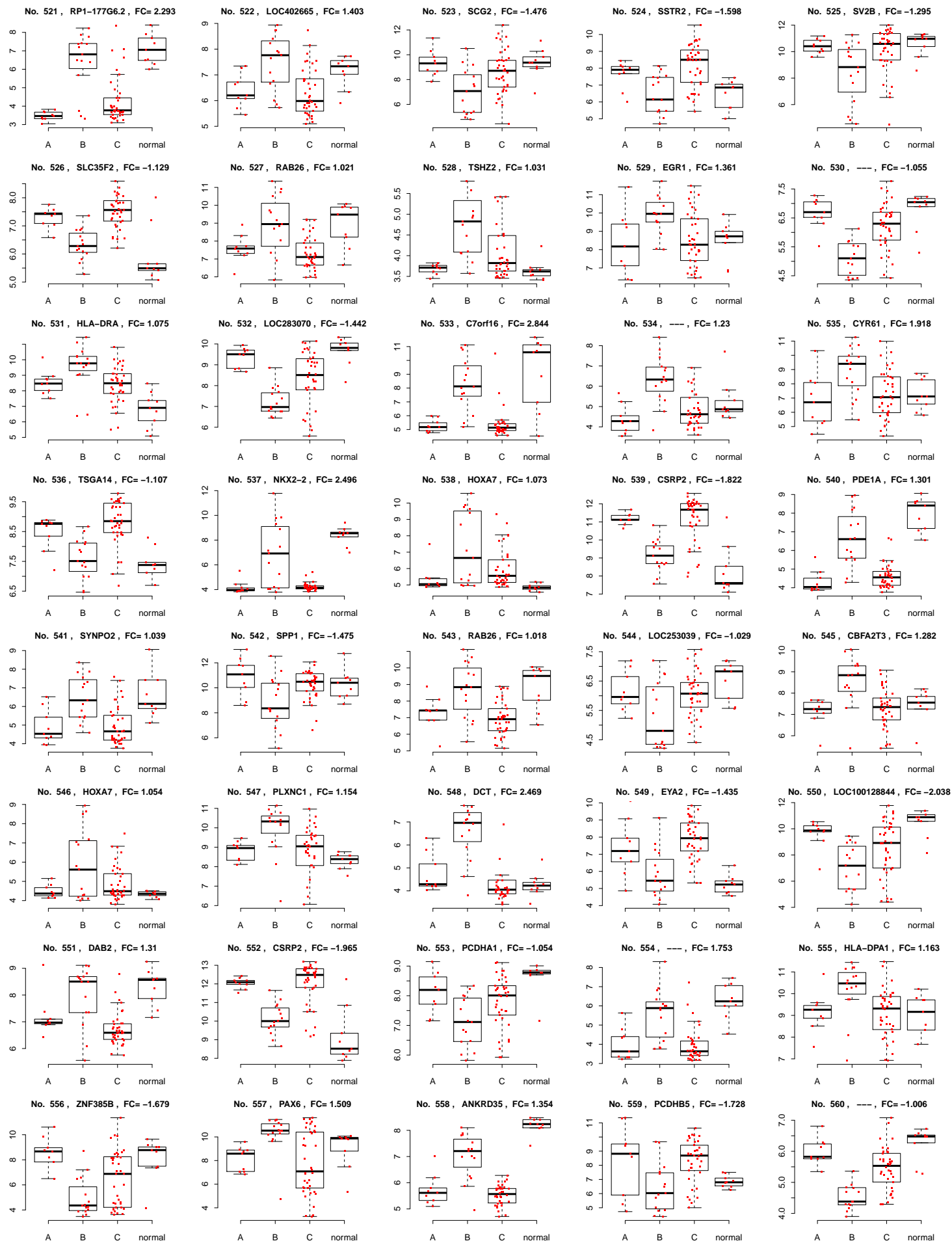

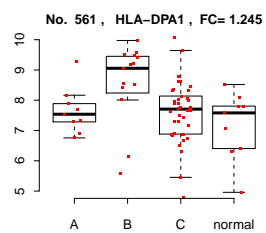

Supplement: Additional file 2 — The converged signatures for the subtypes of the three datasets. [file 1471-2105-14-S18-S1-S2.zip › plot-Kool62-SubtypeB.pdf]

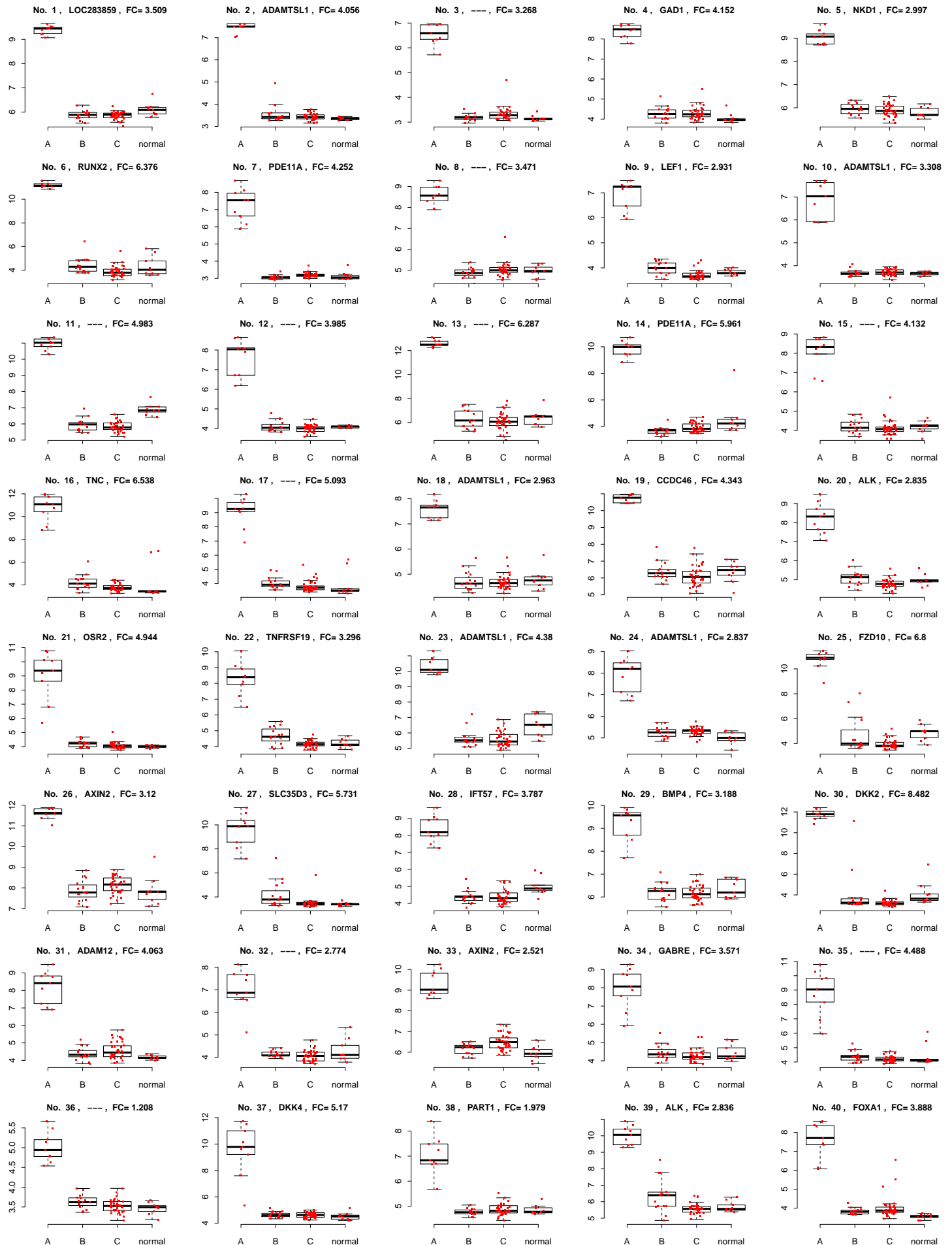

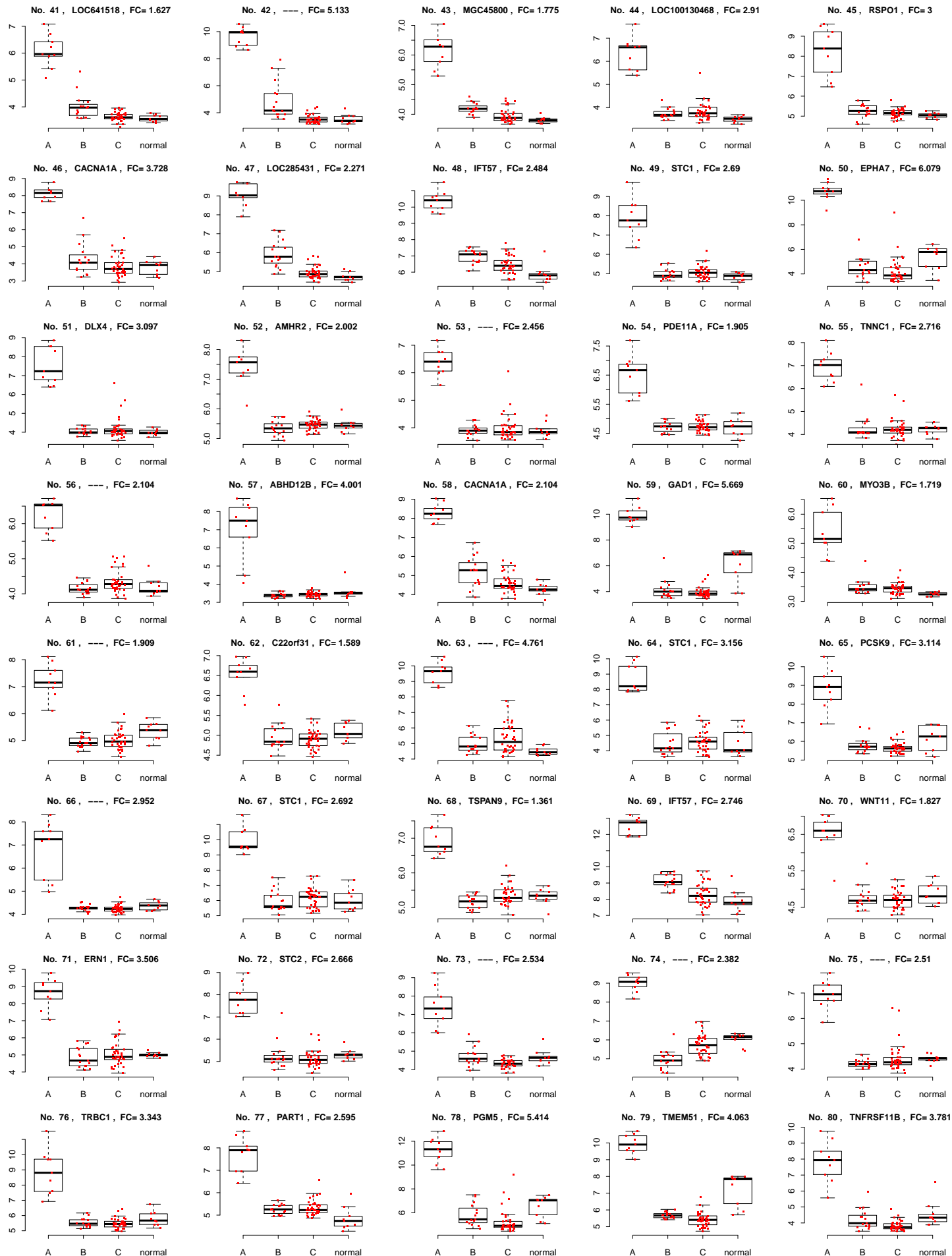

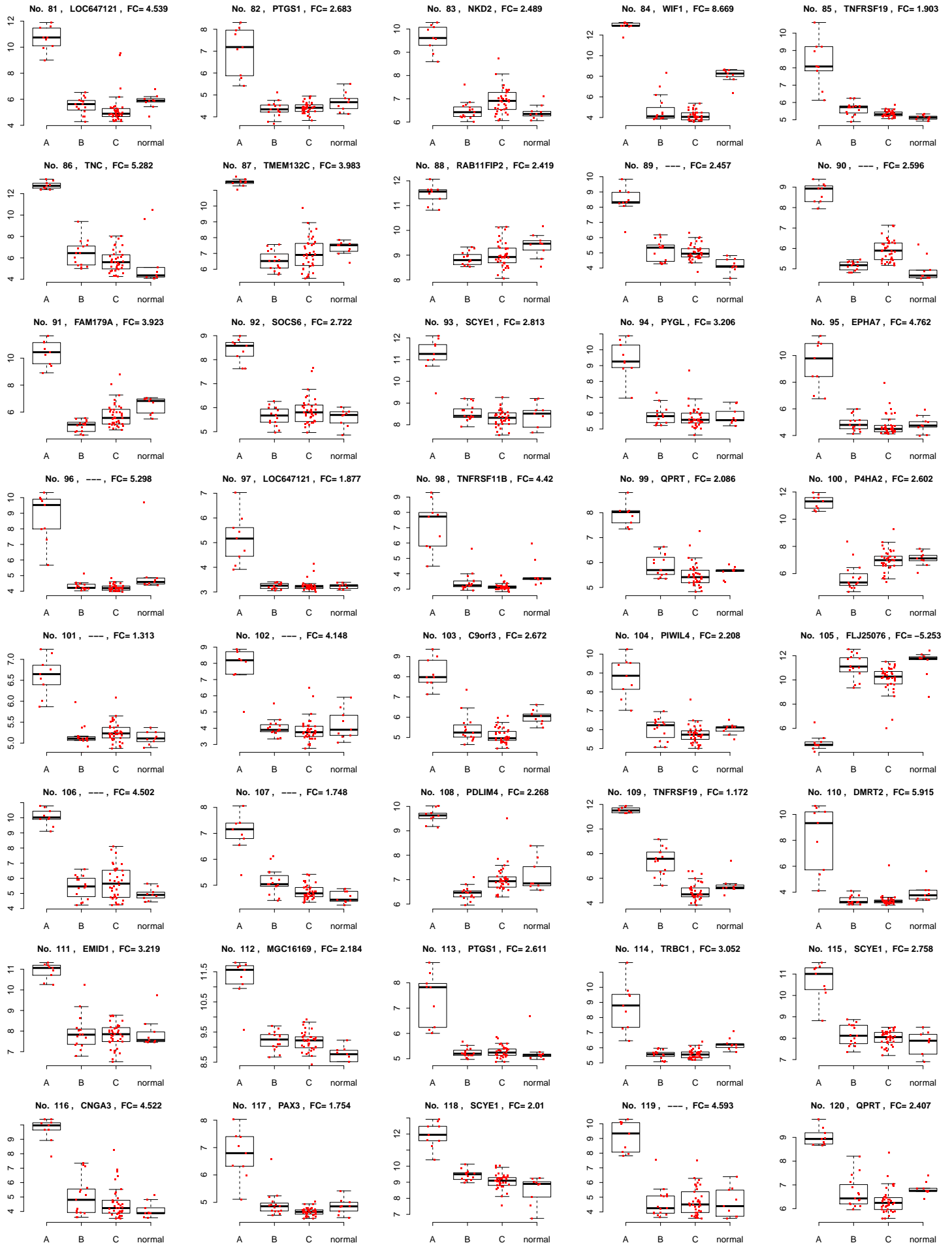

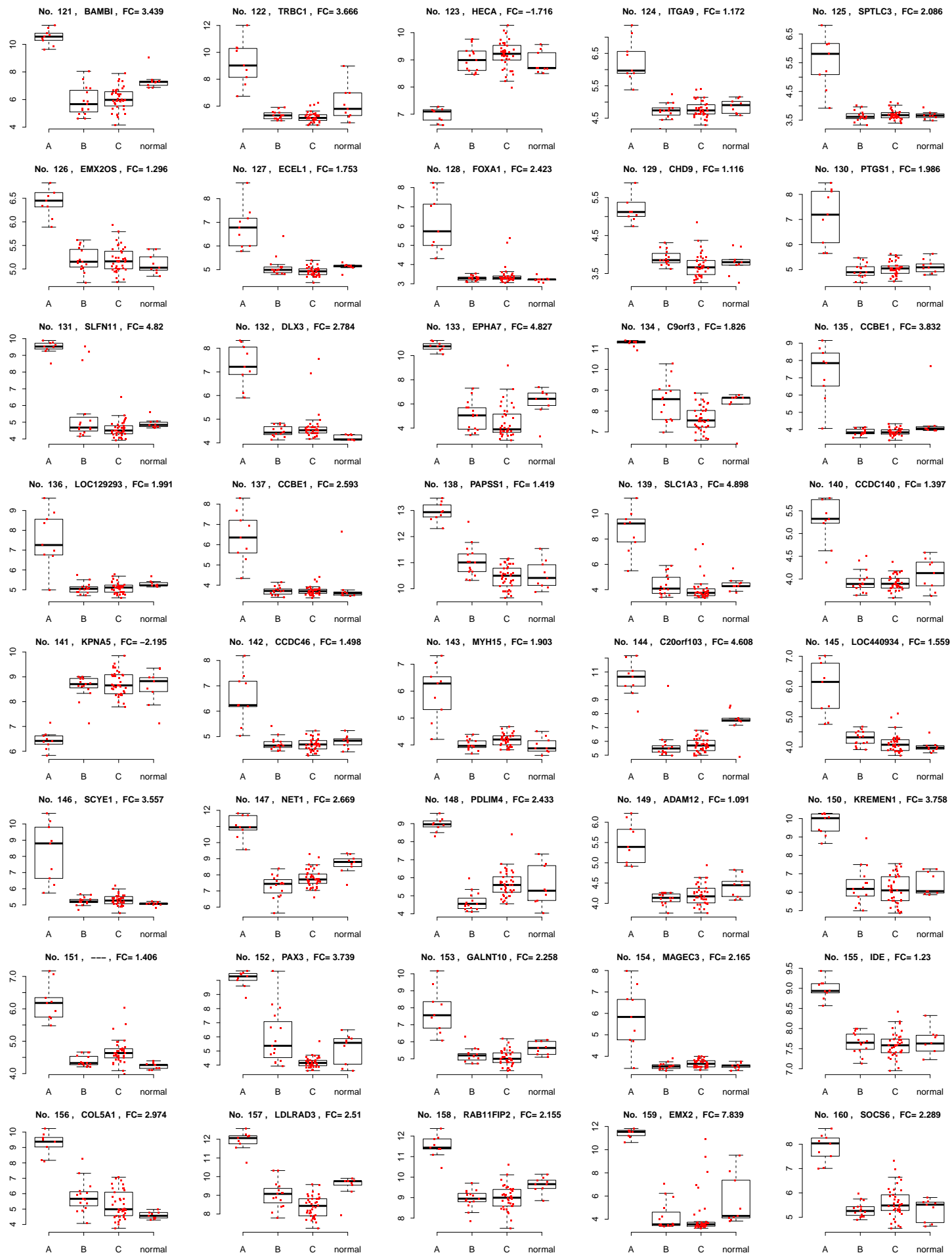

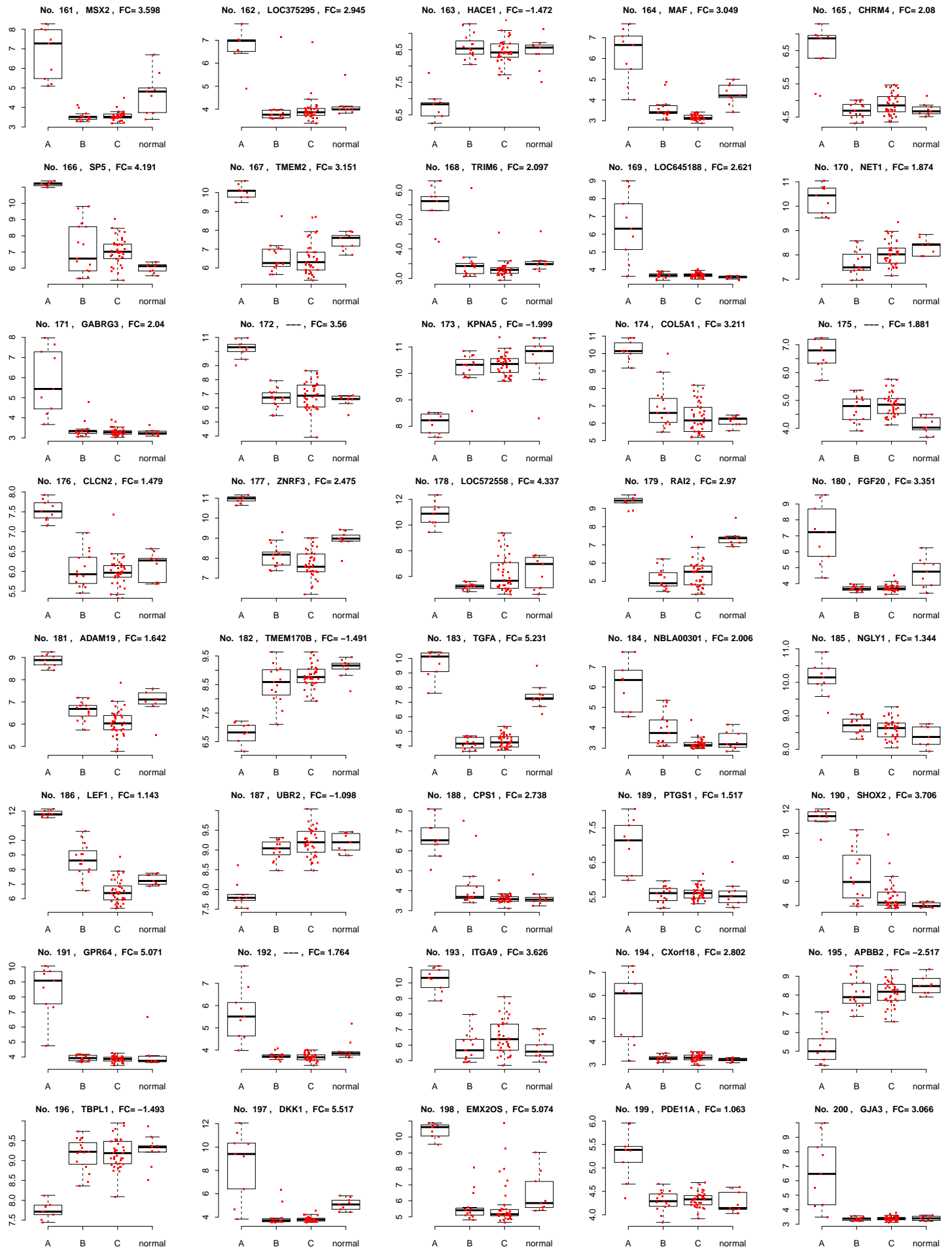

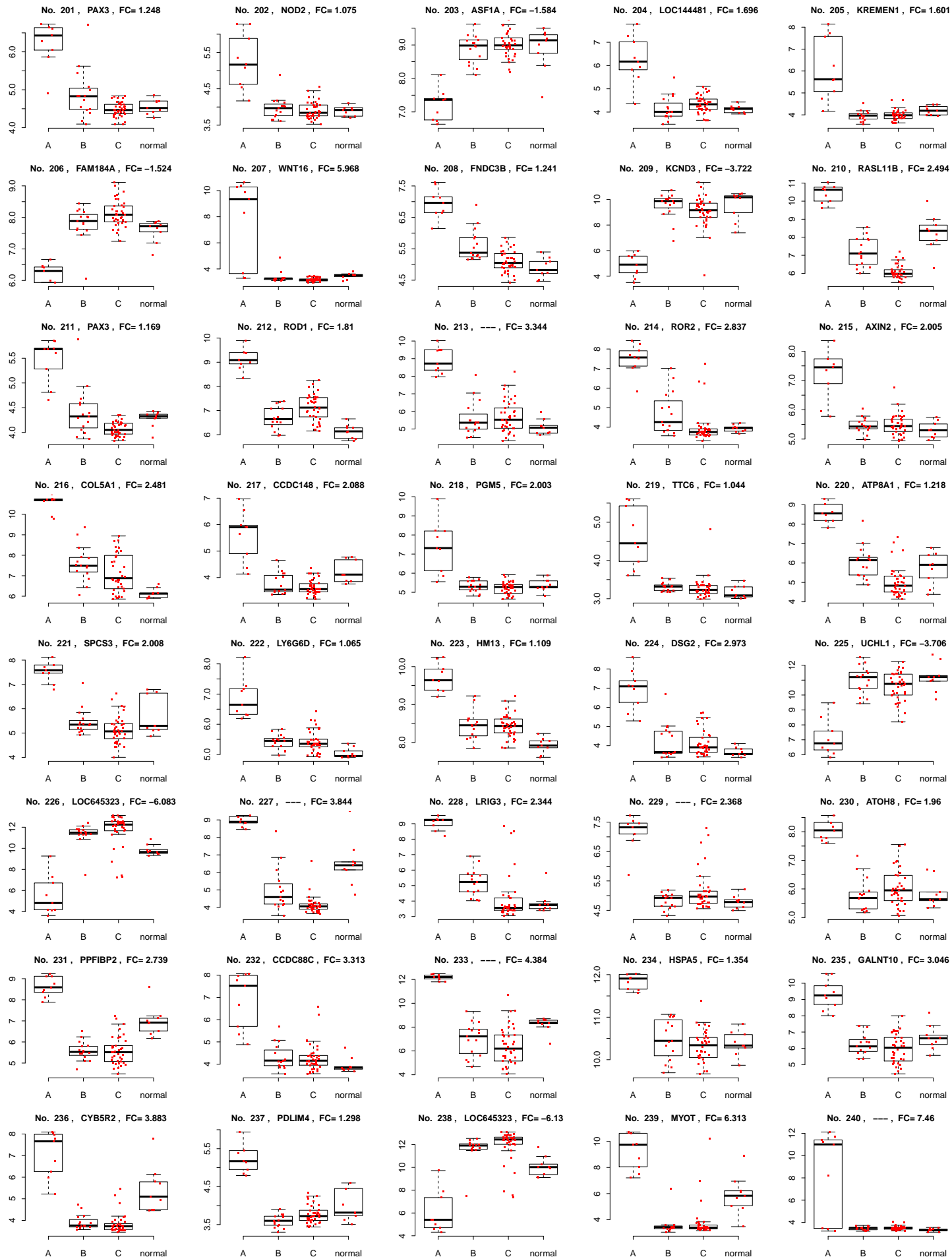

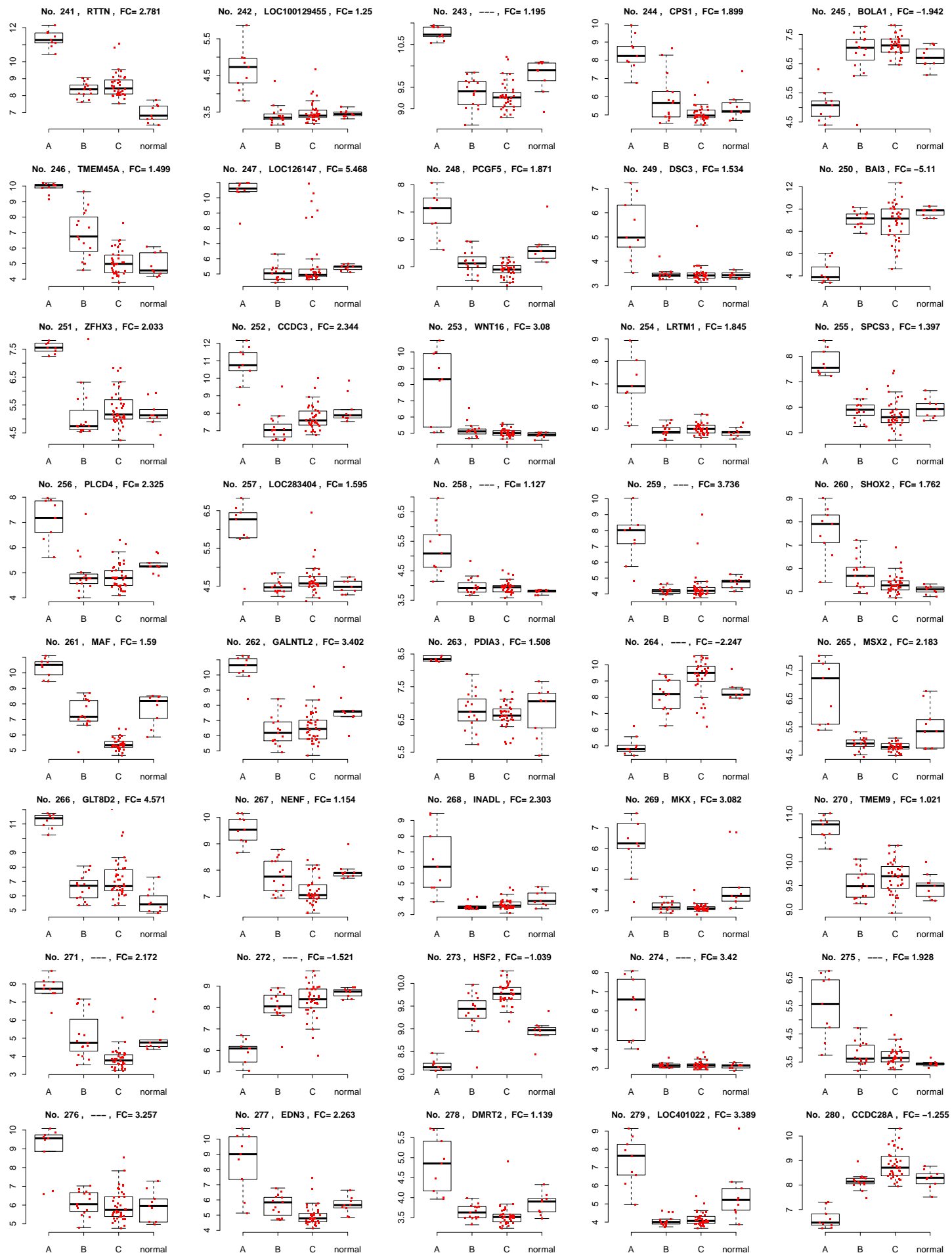

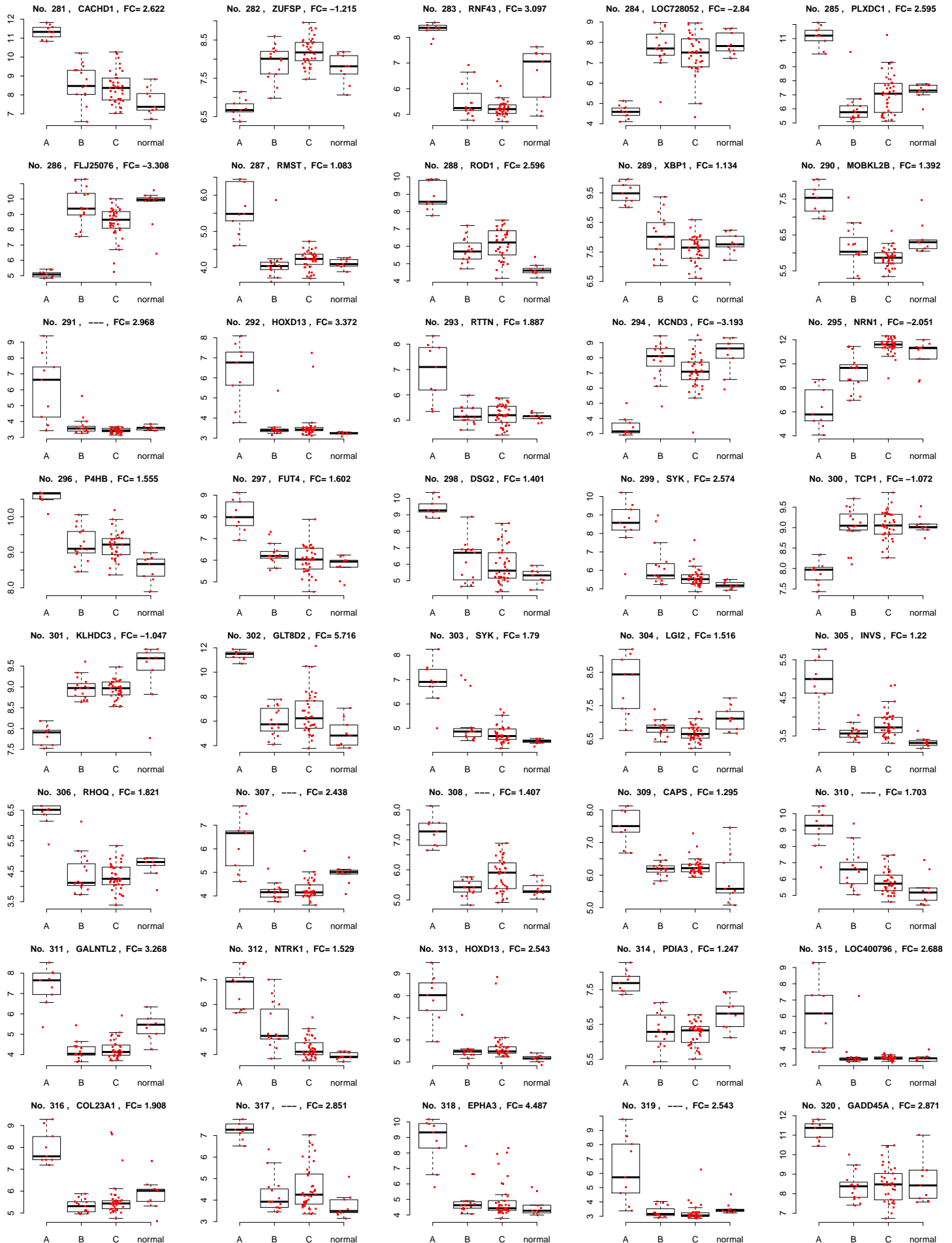

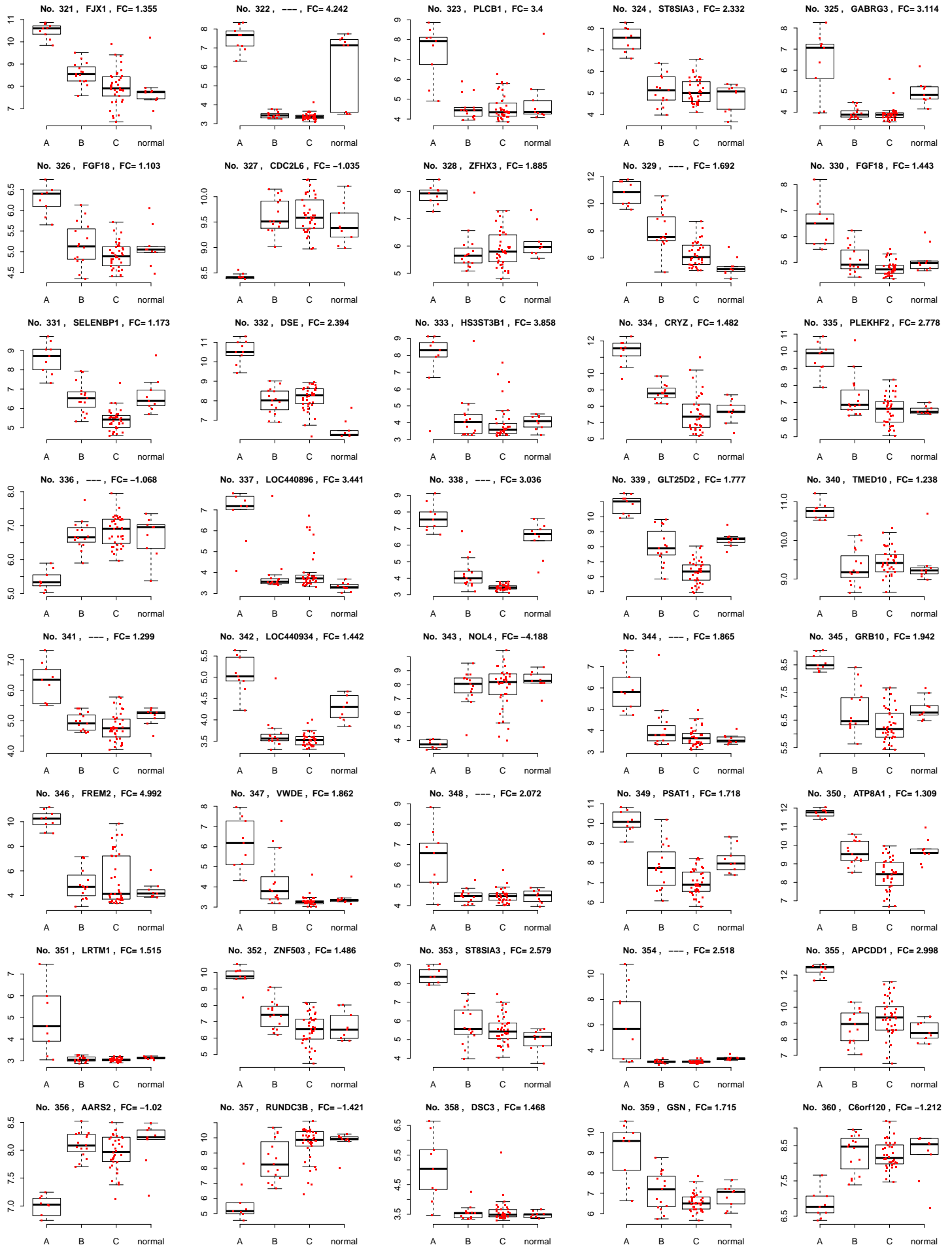

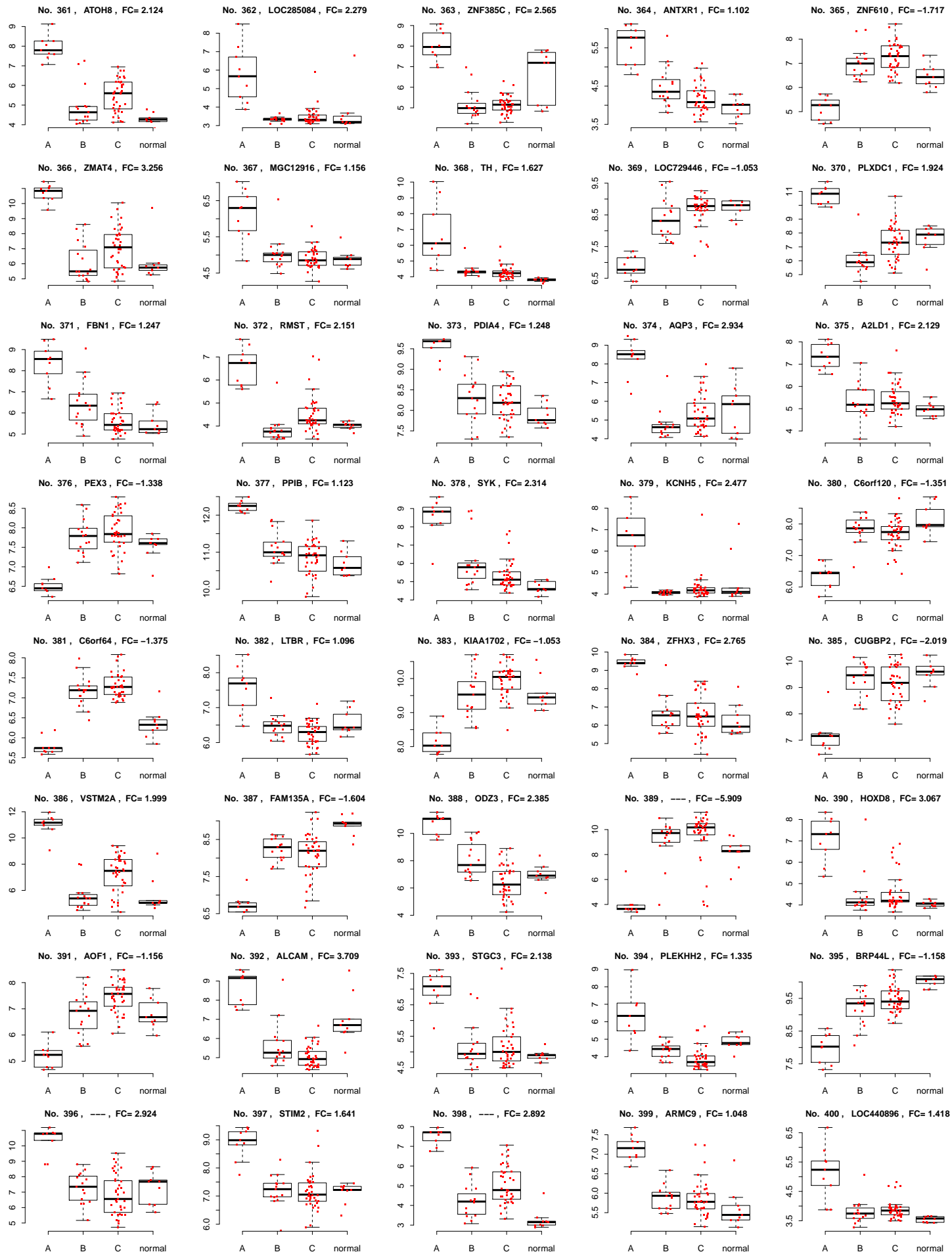

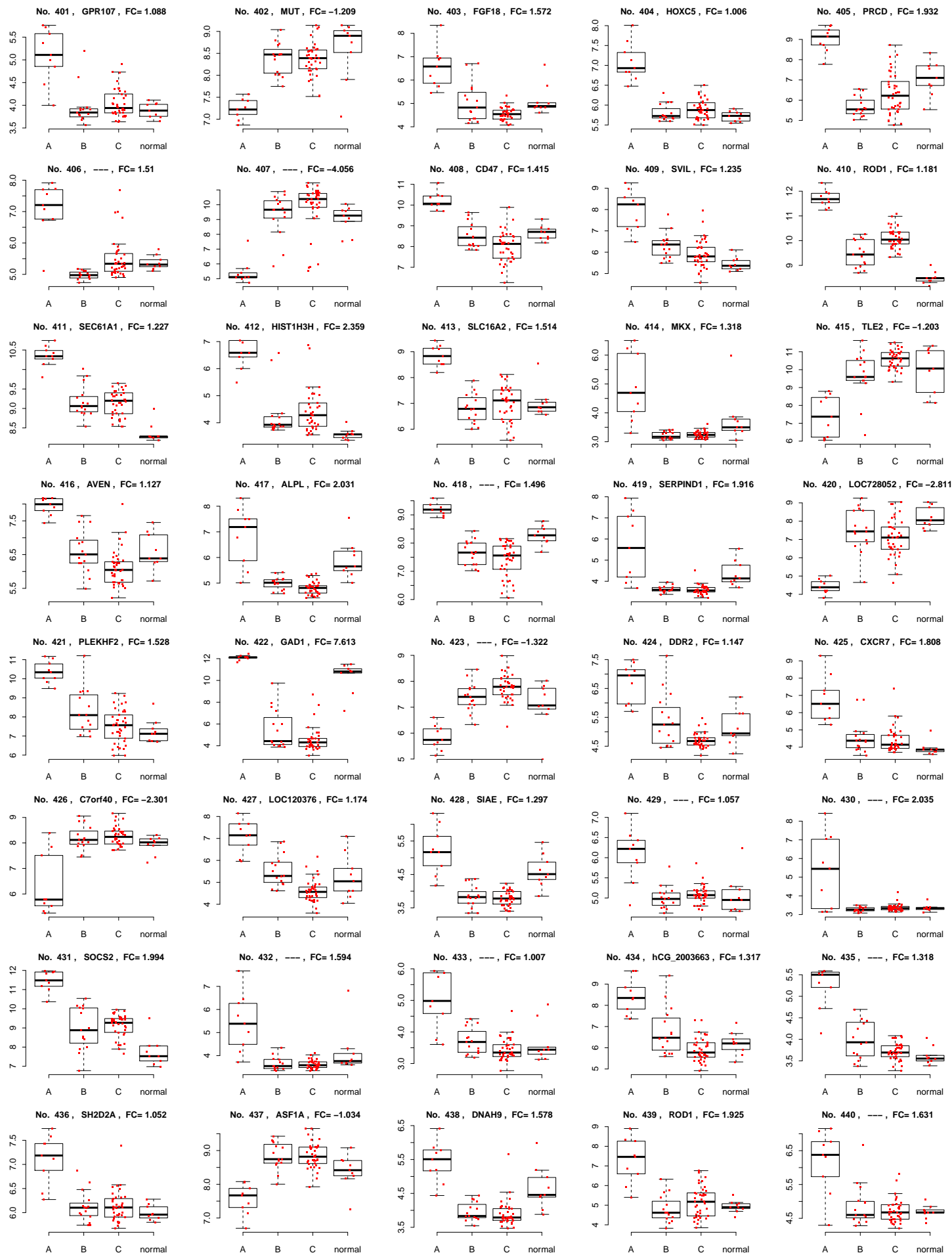

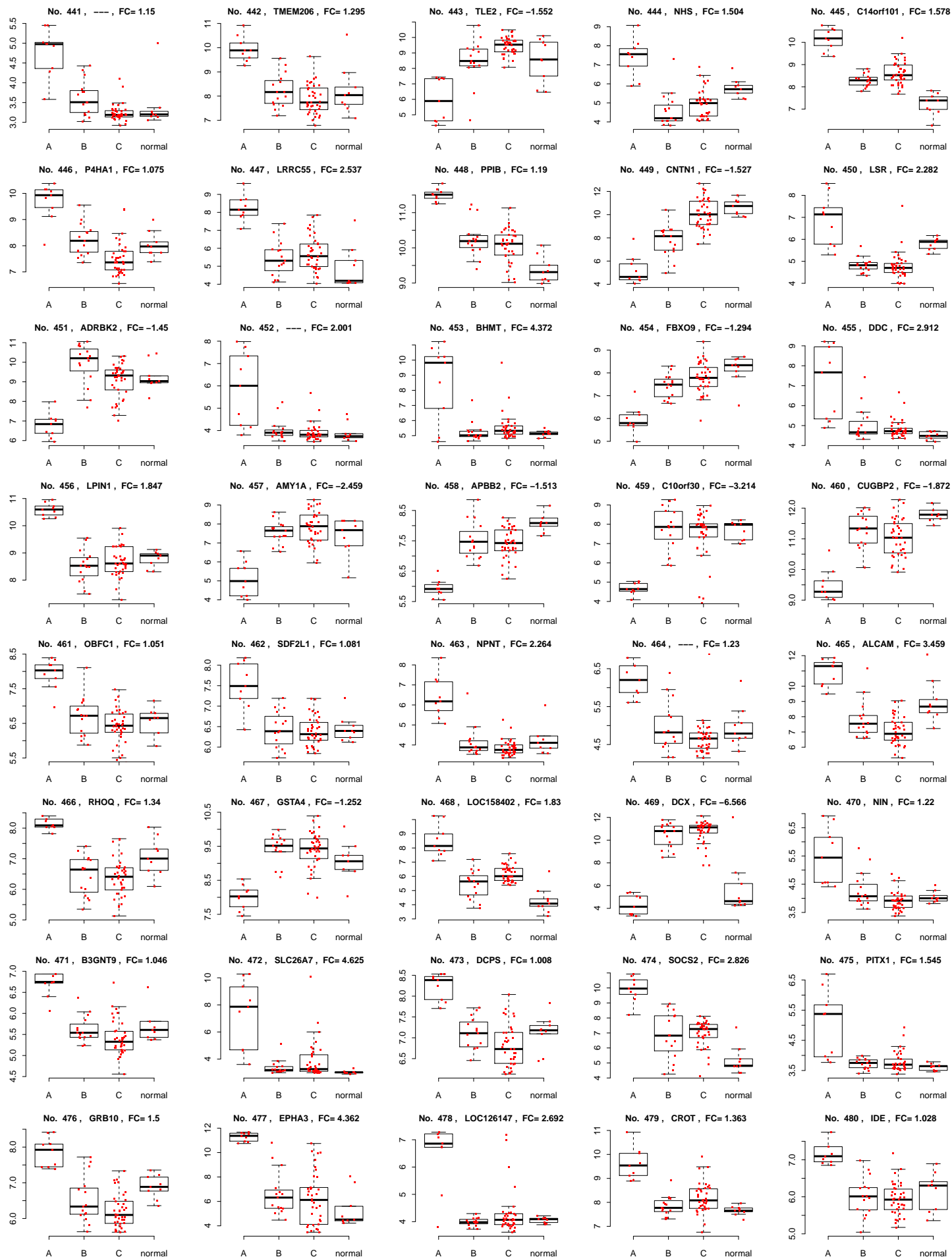

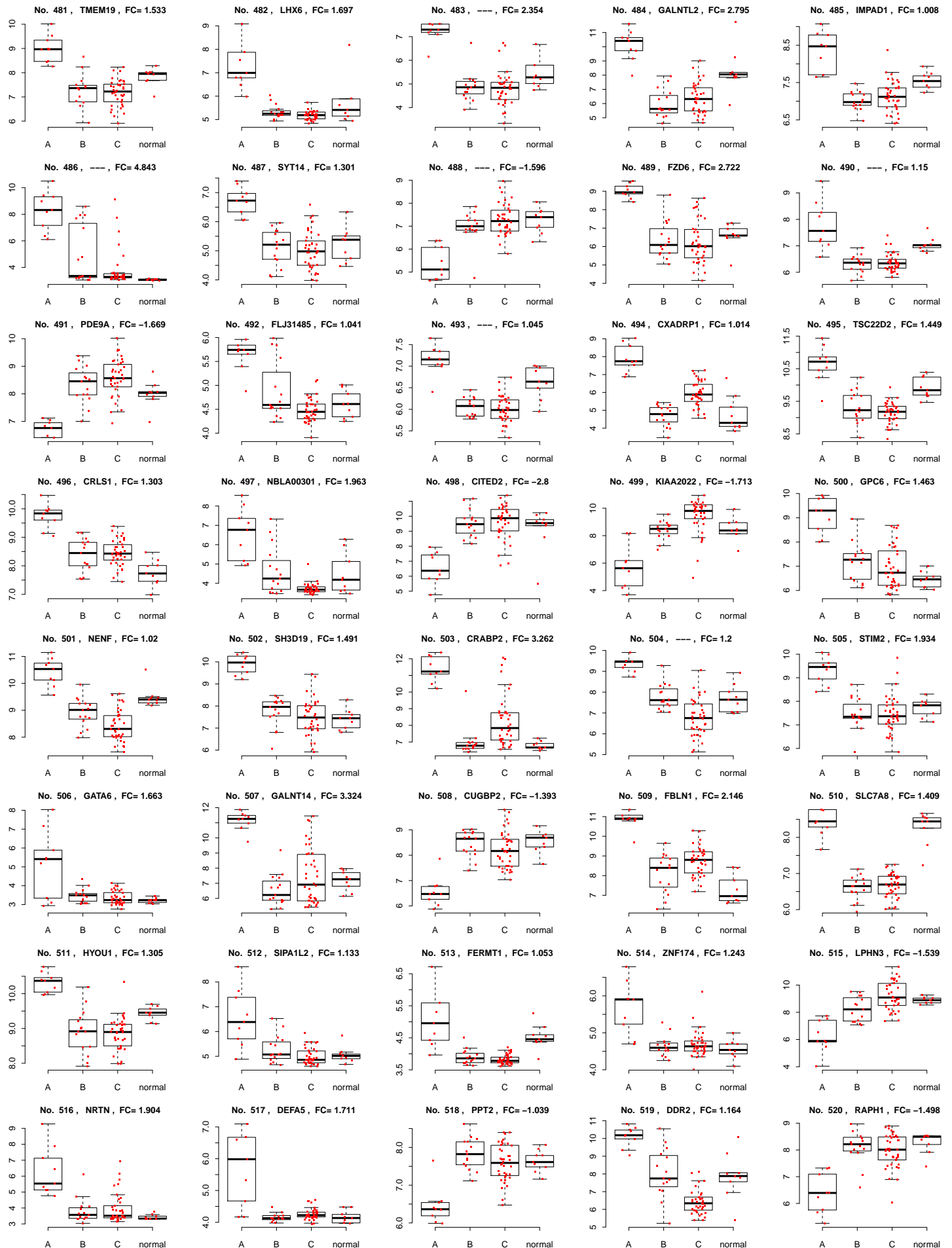

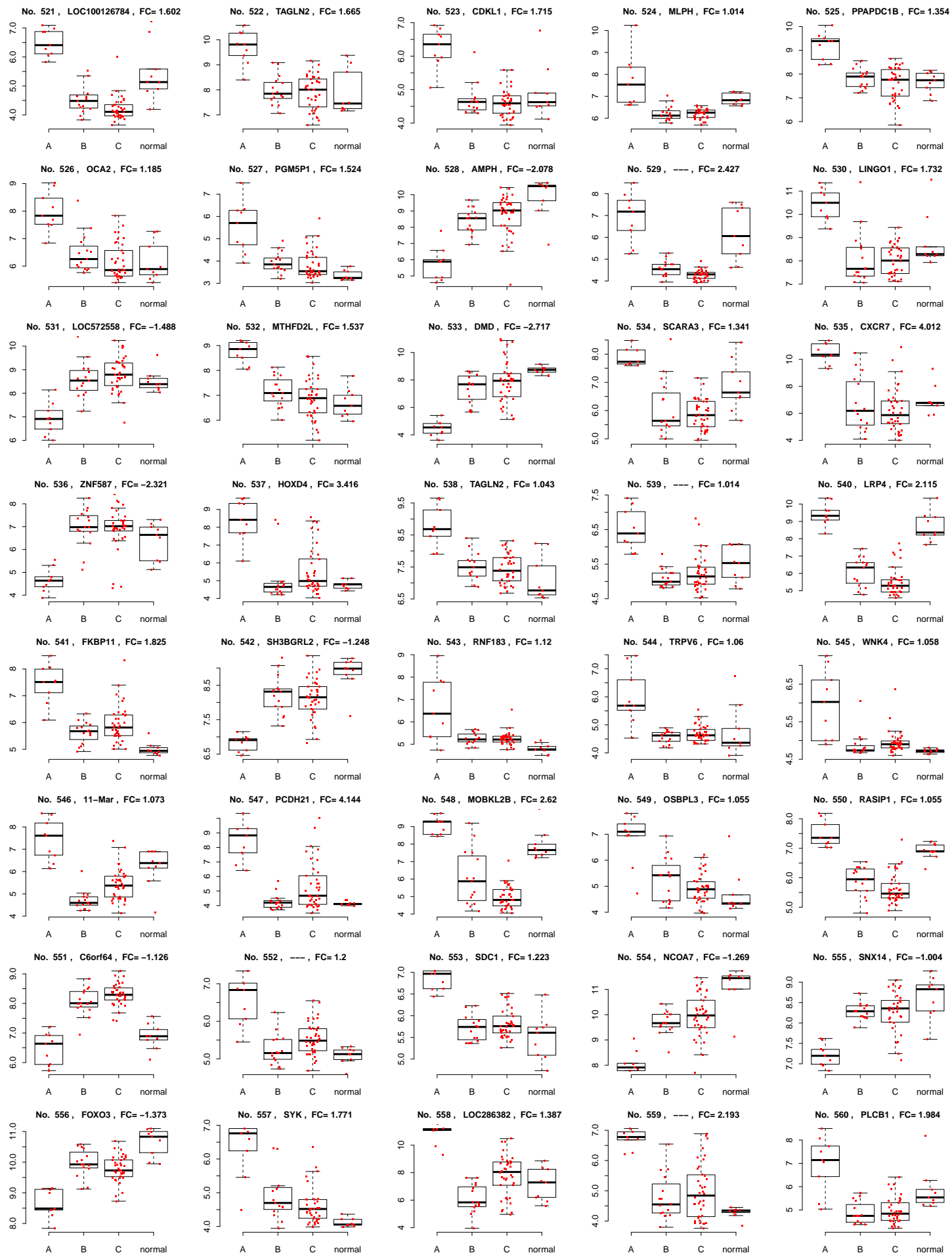

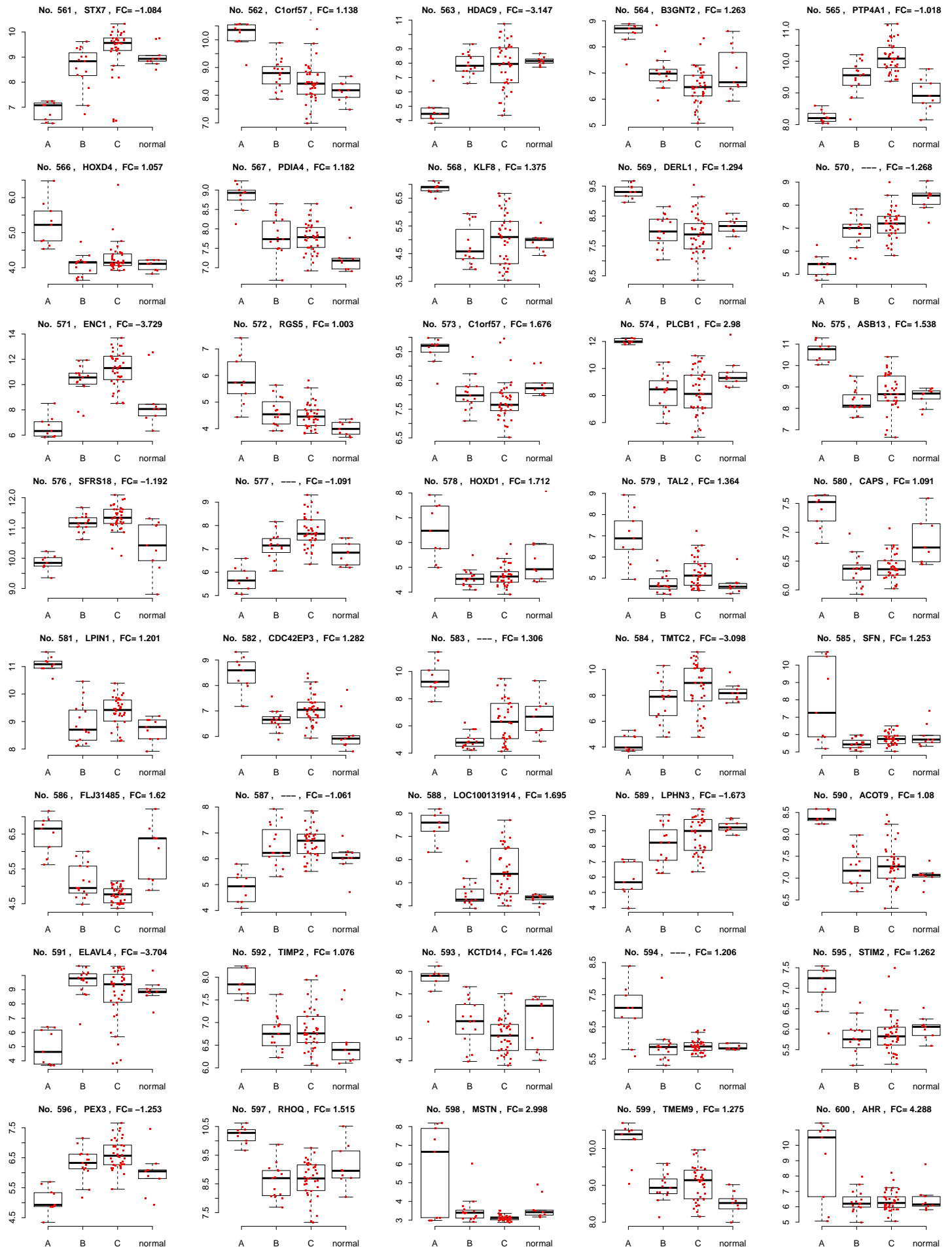

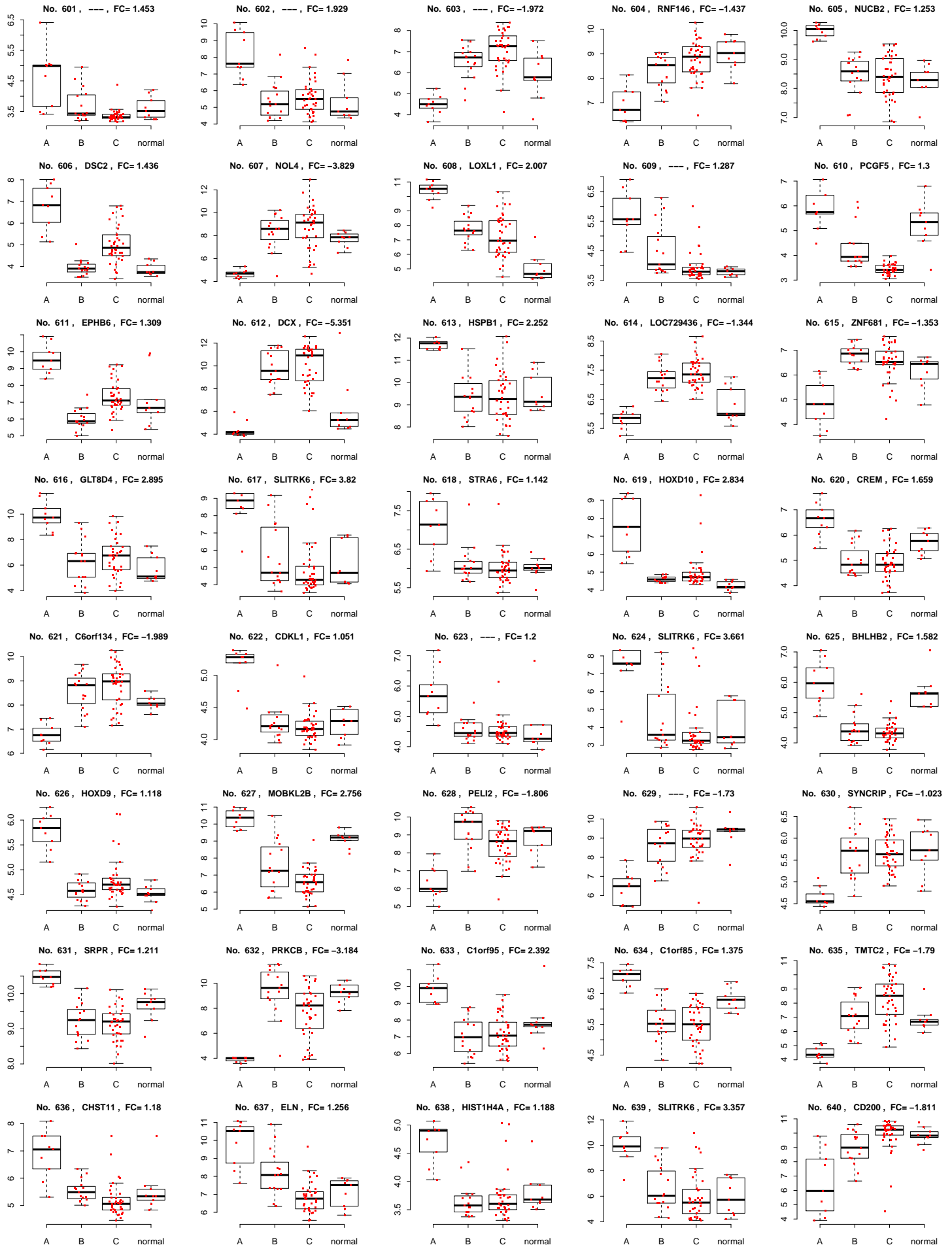

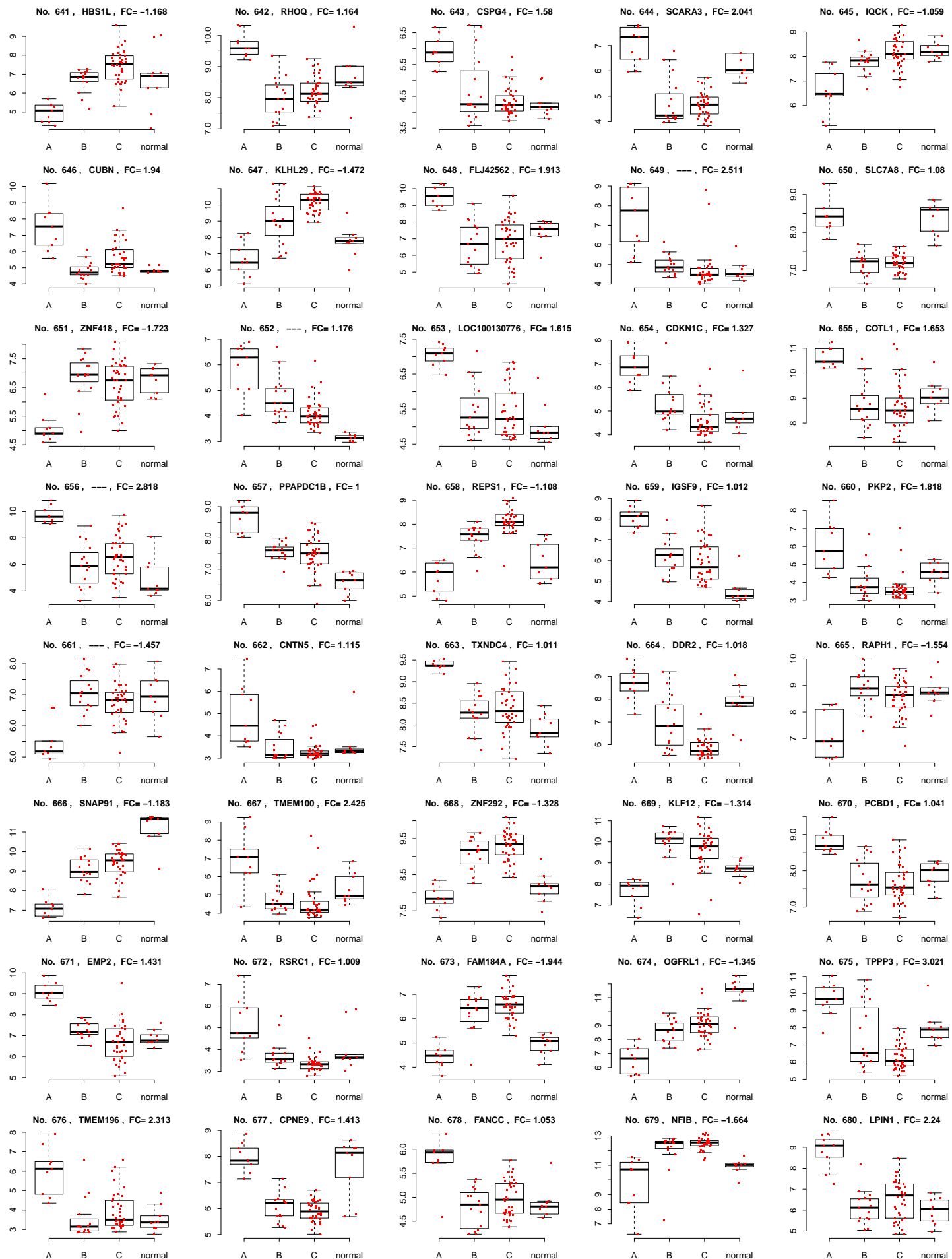

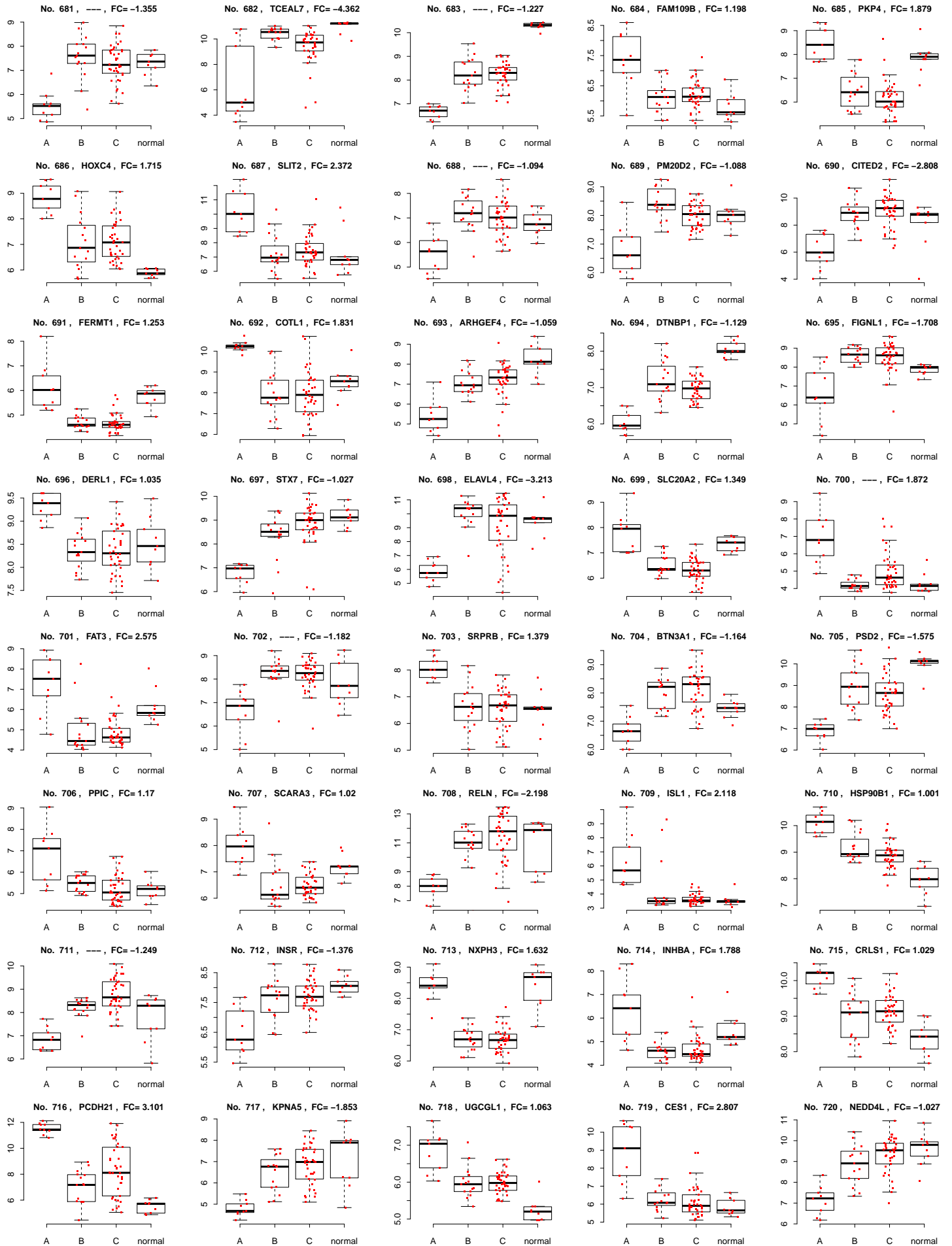

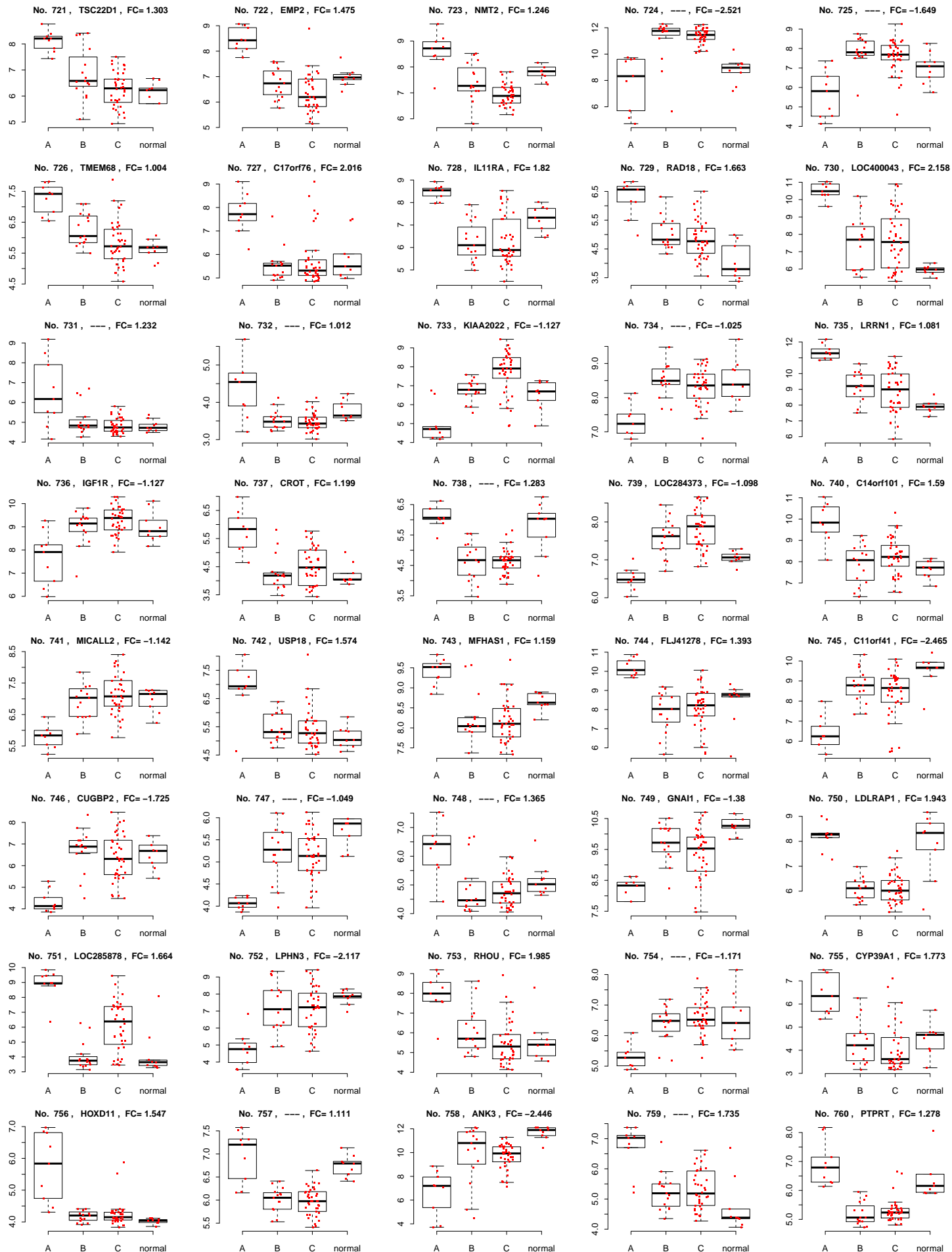

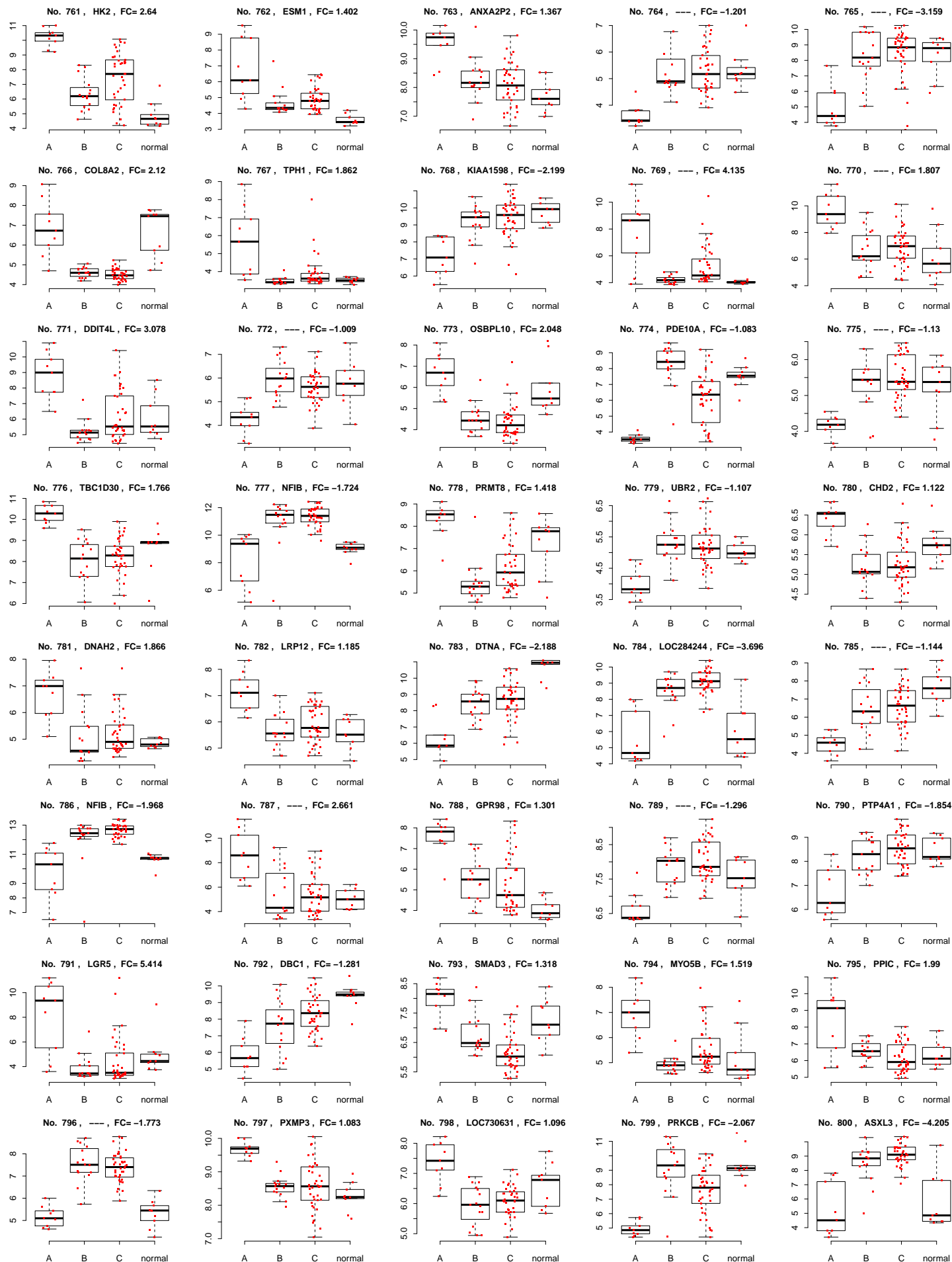

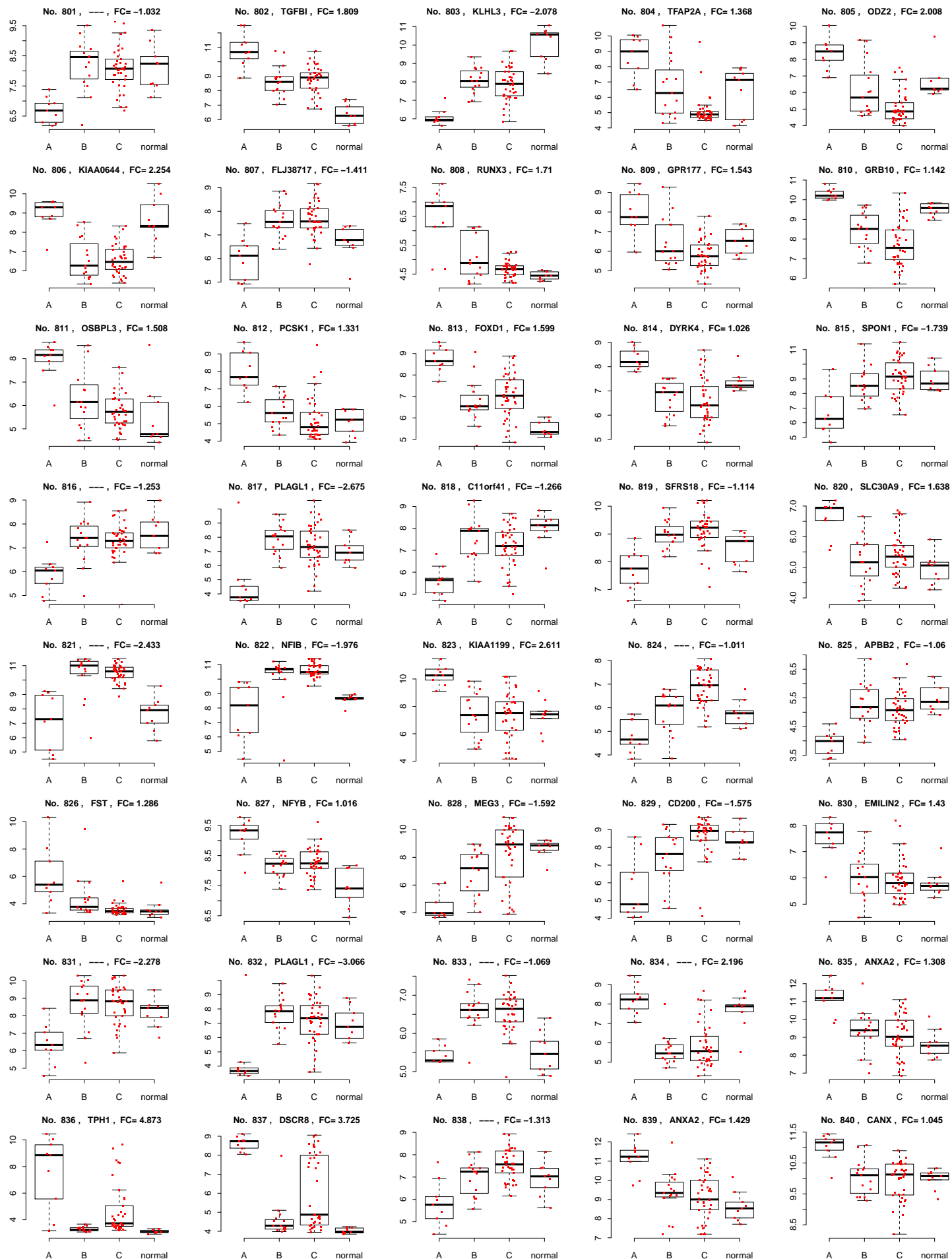

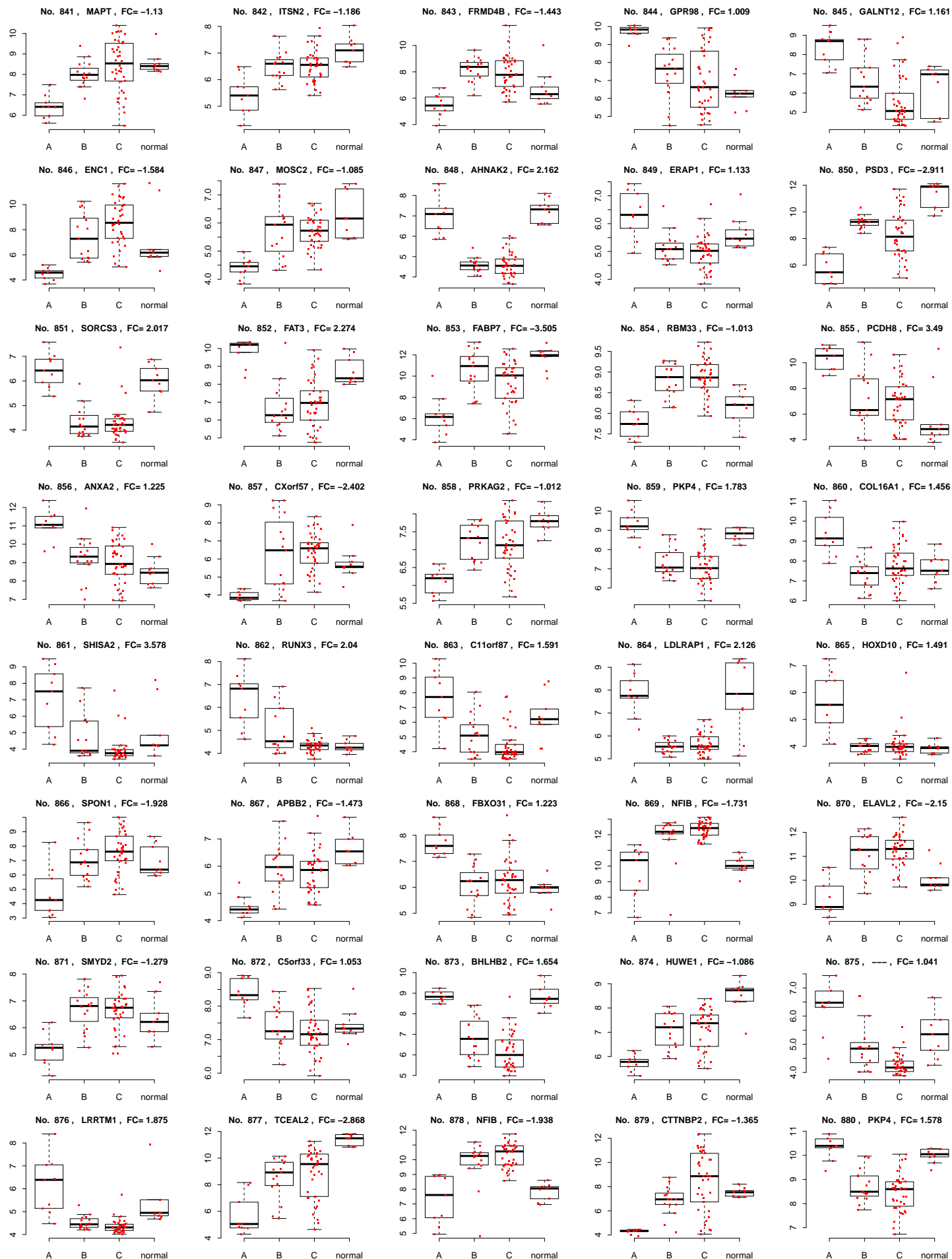

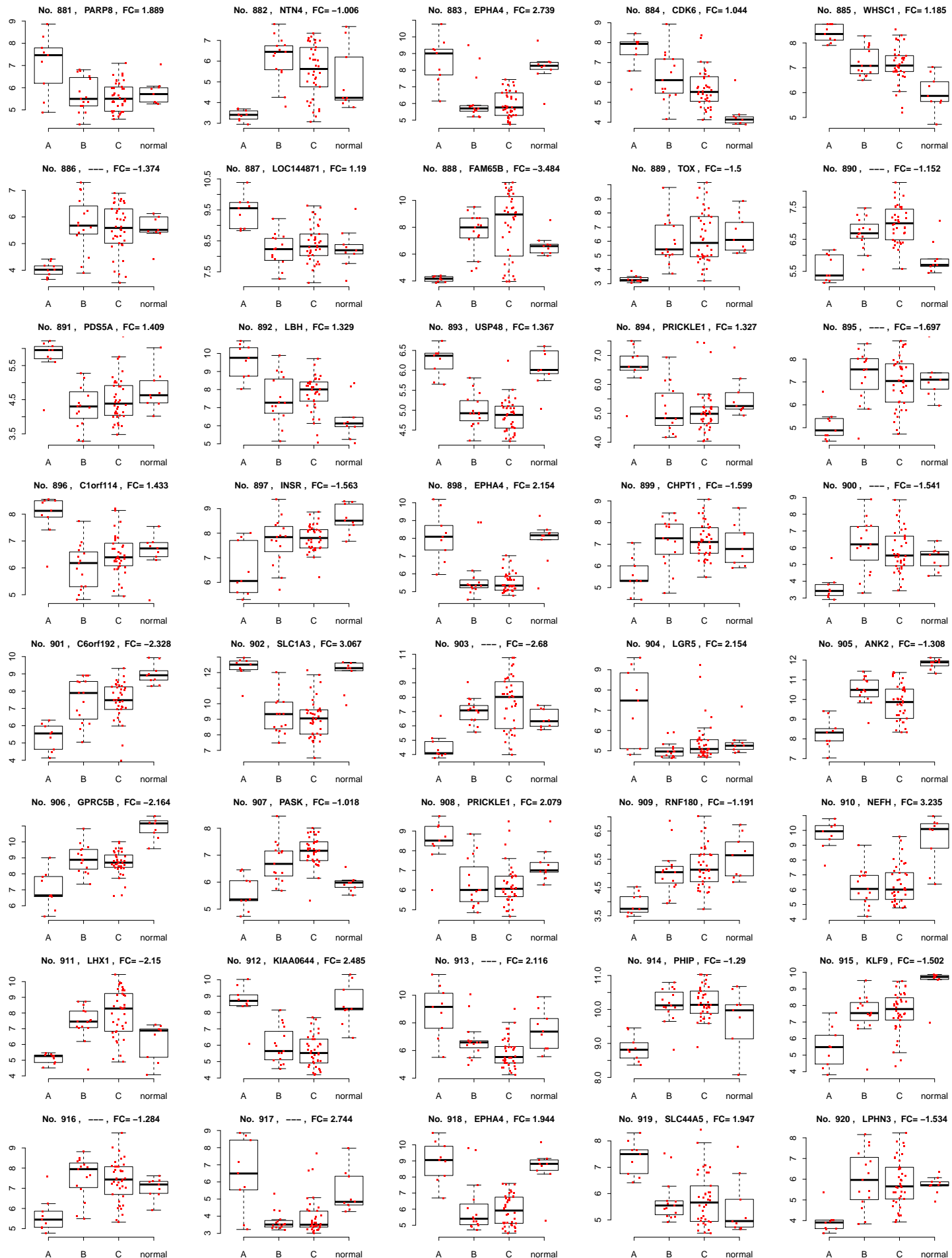

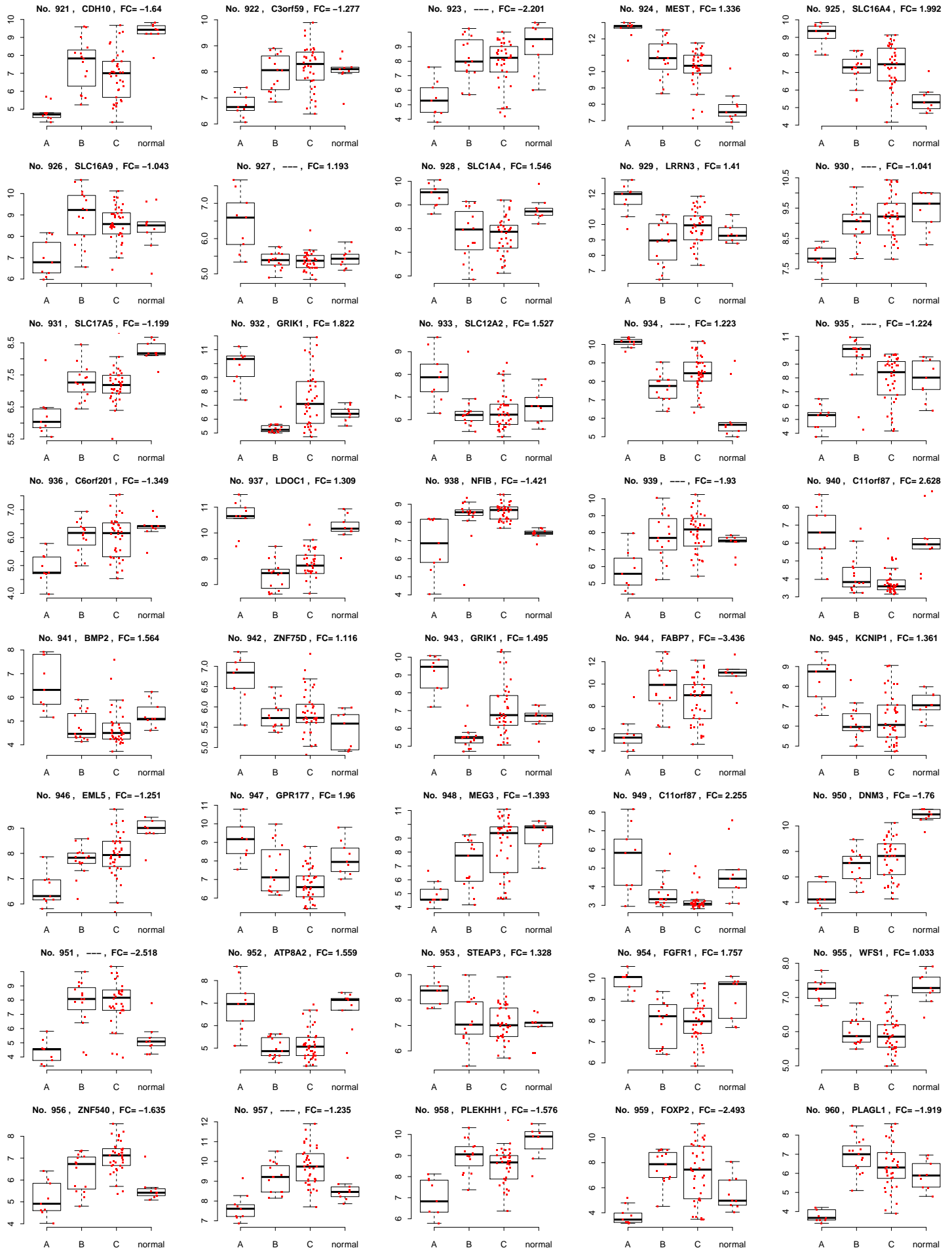

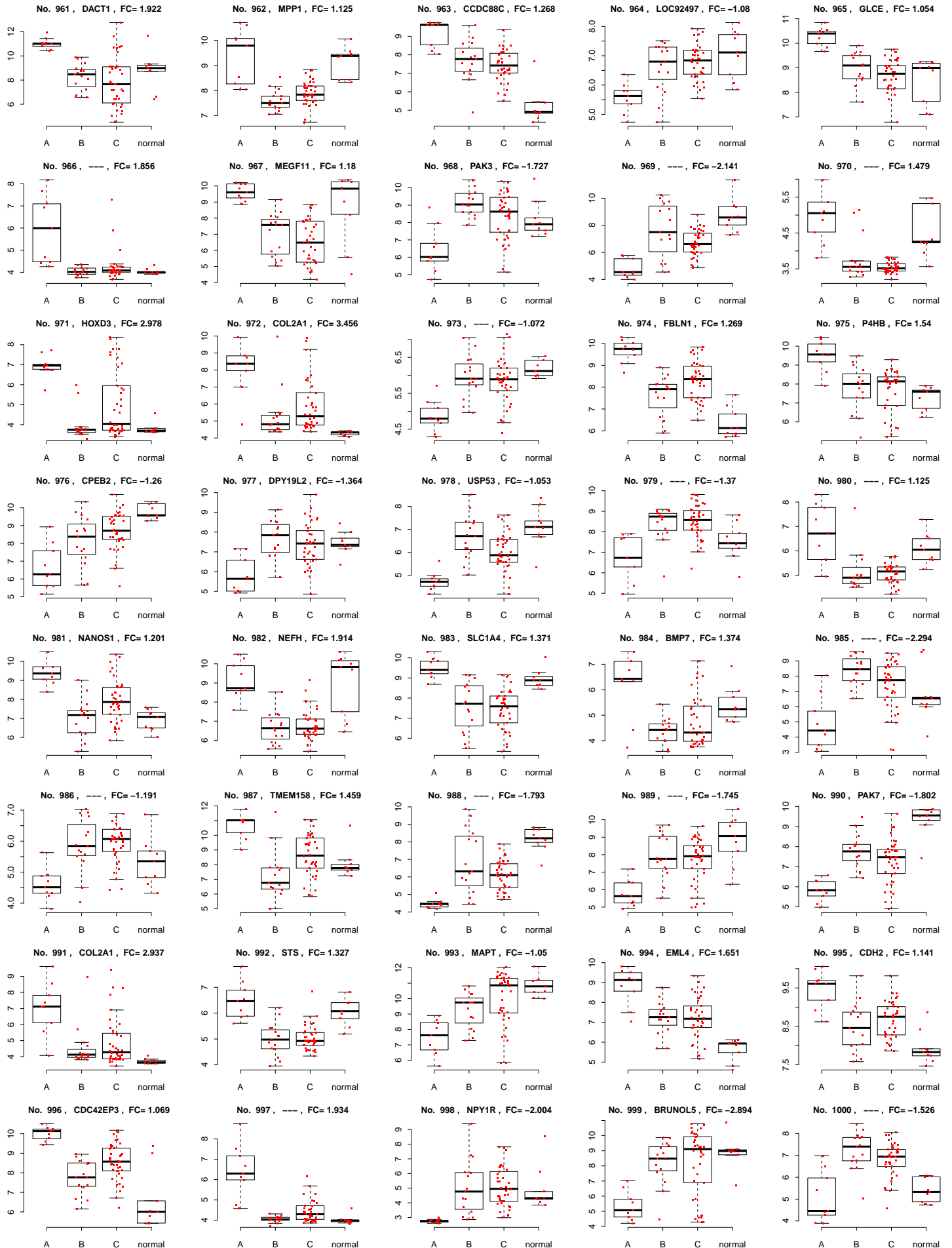

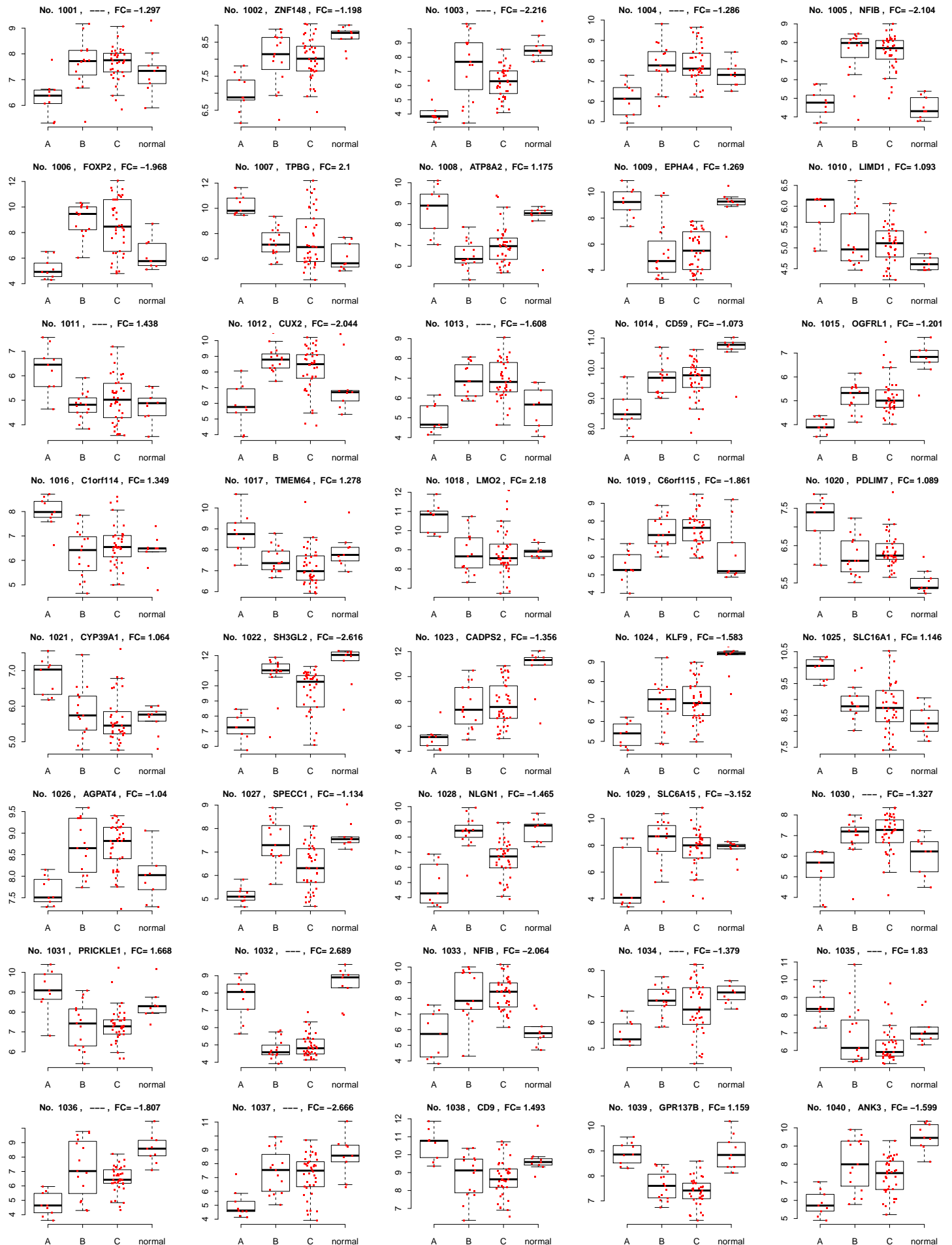

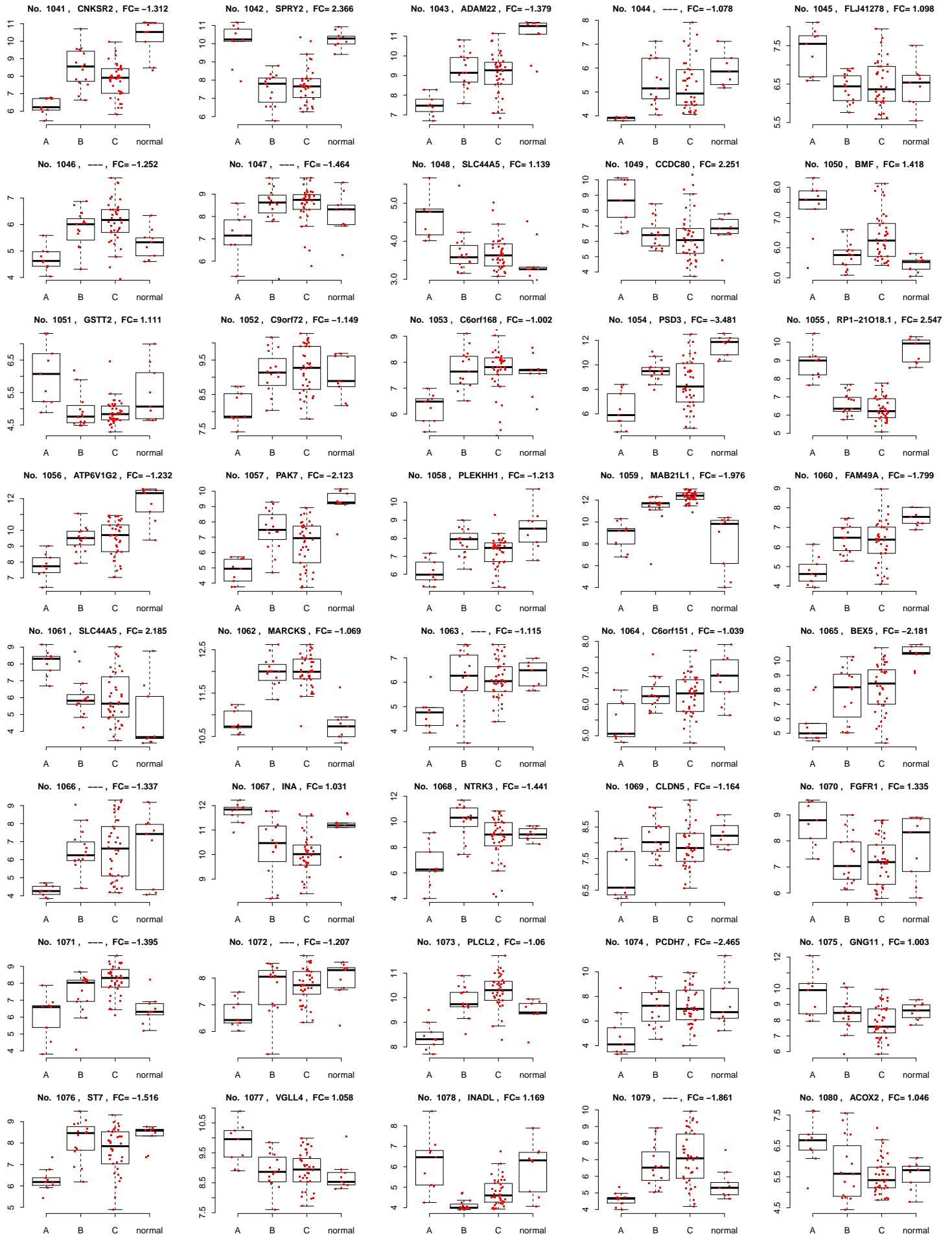

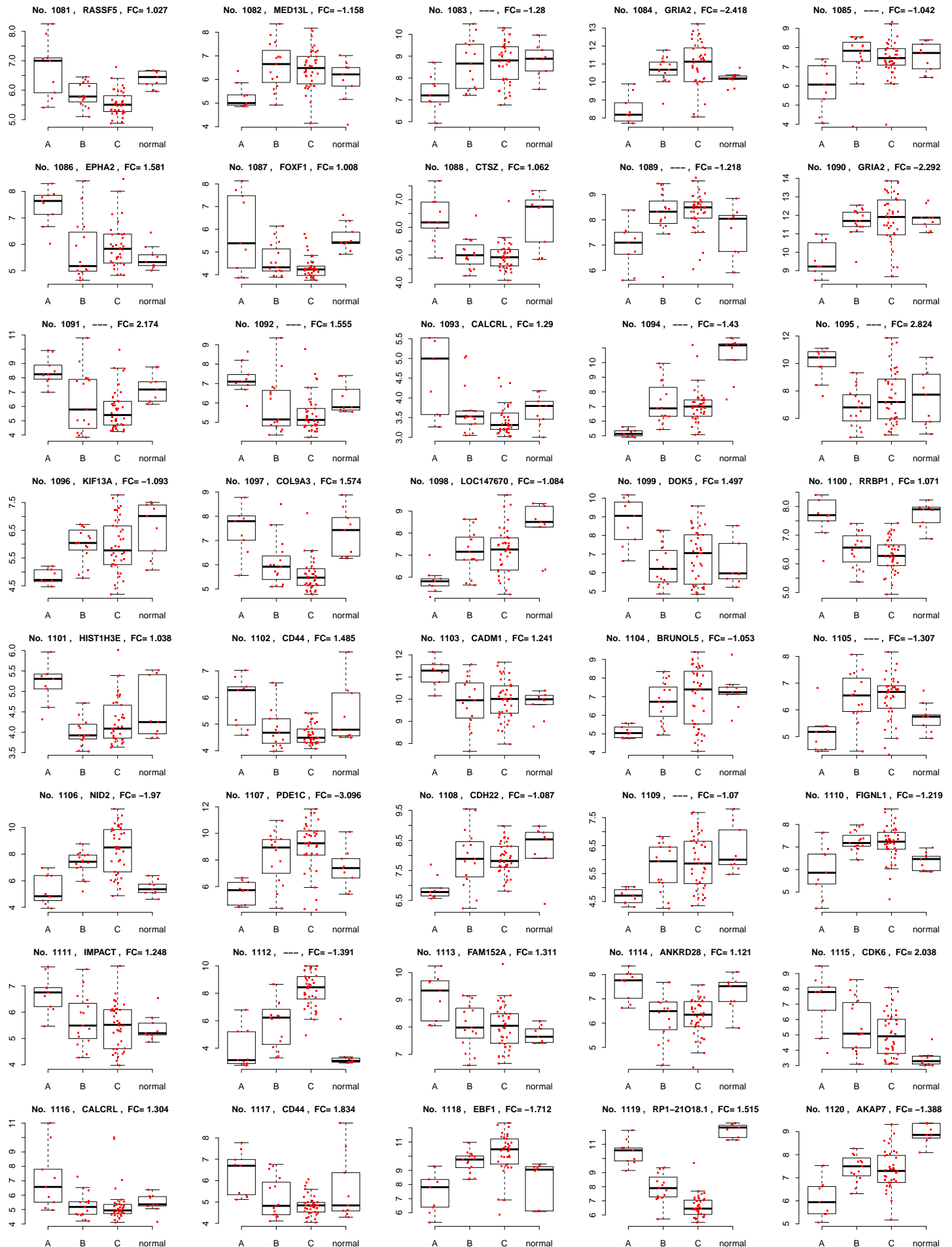

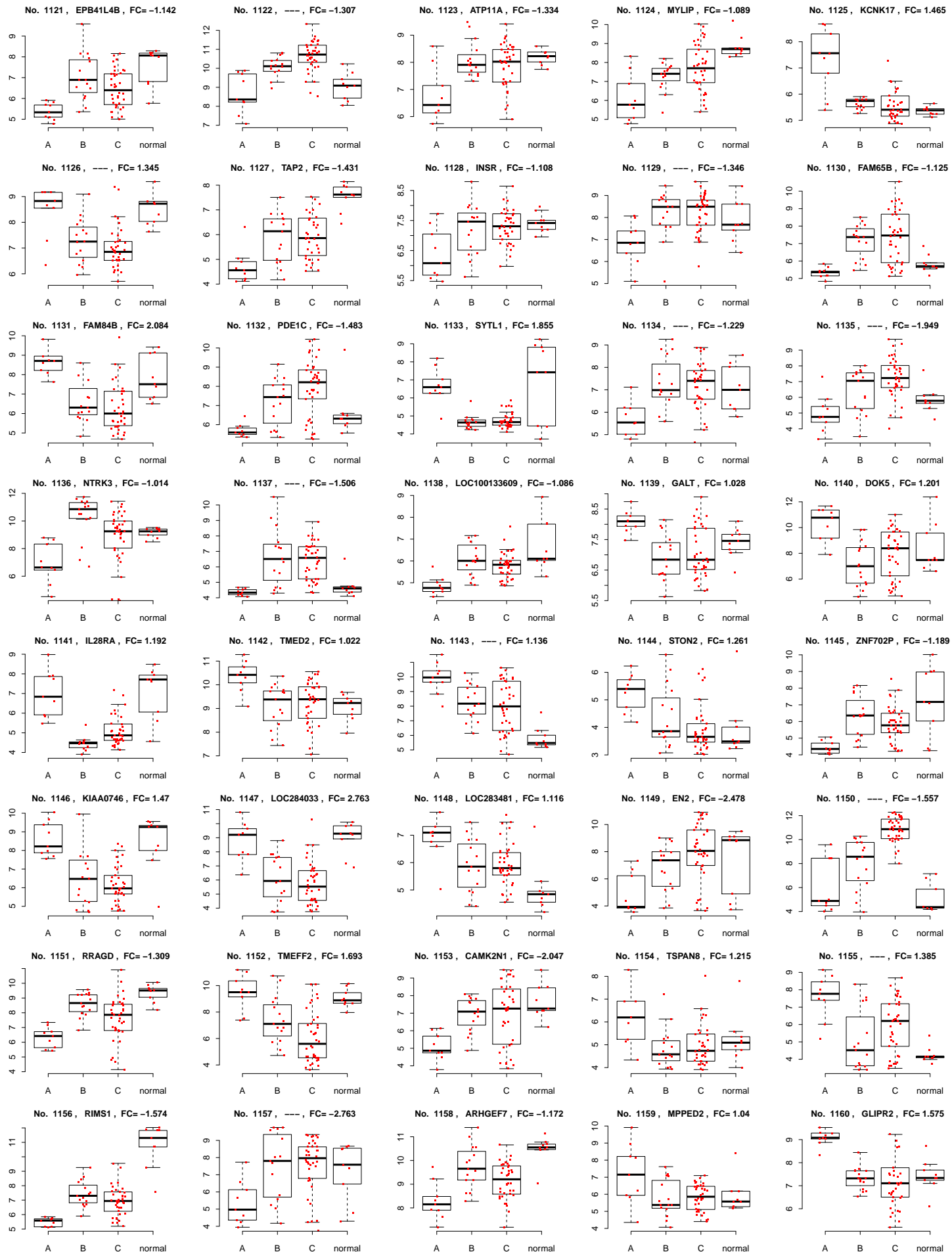

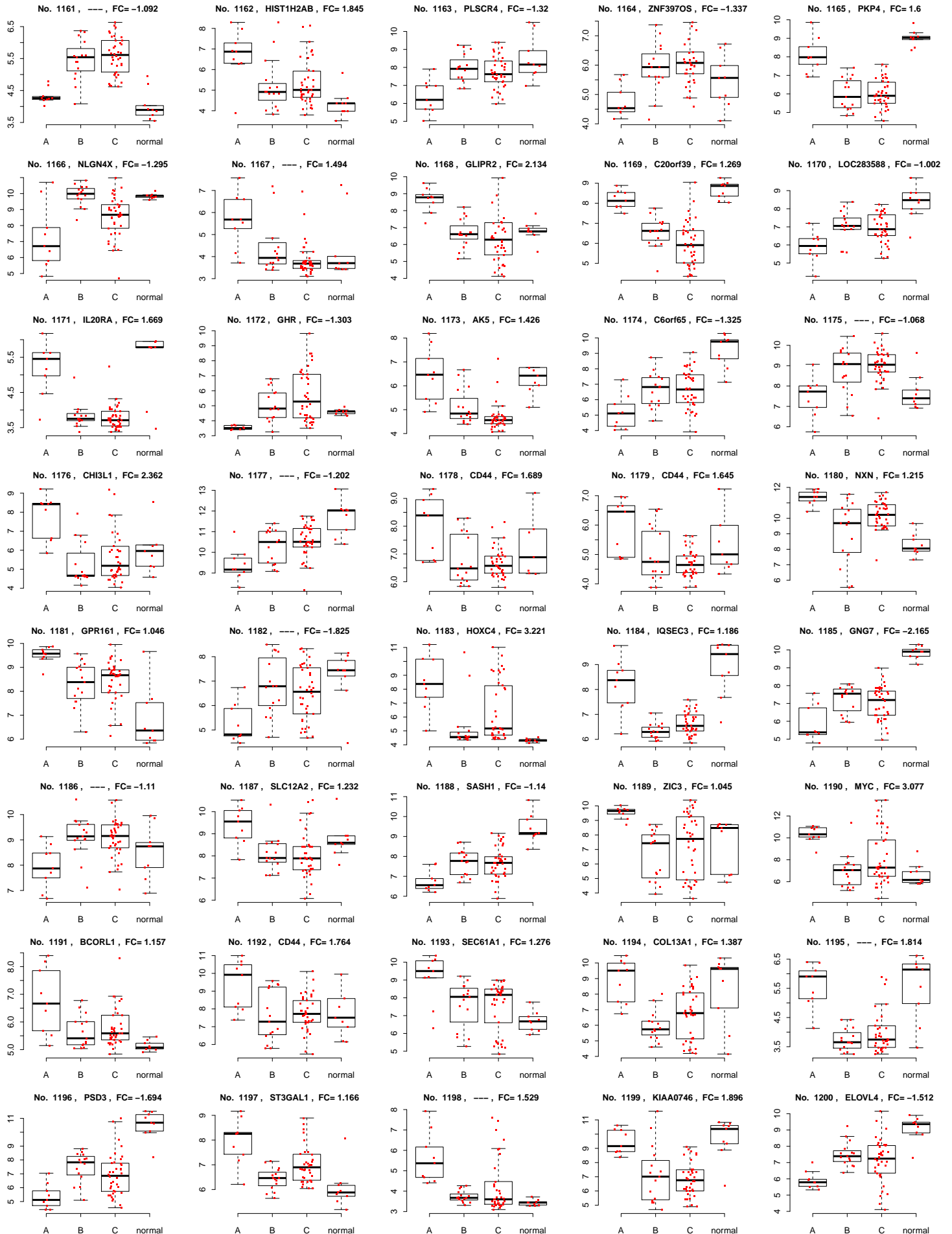

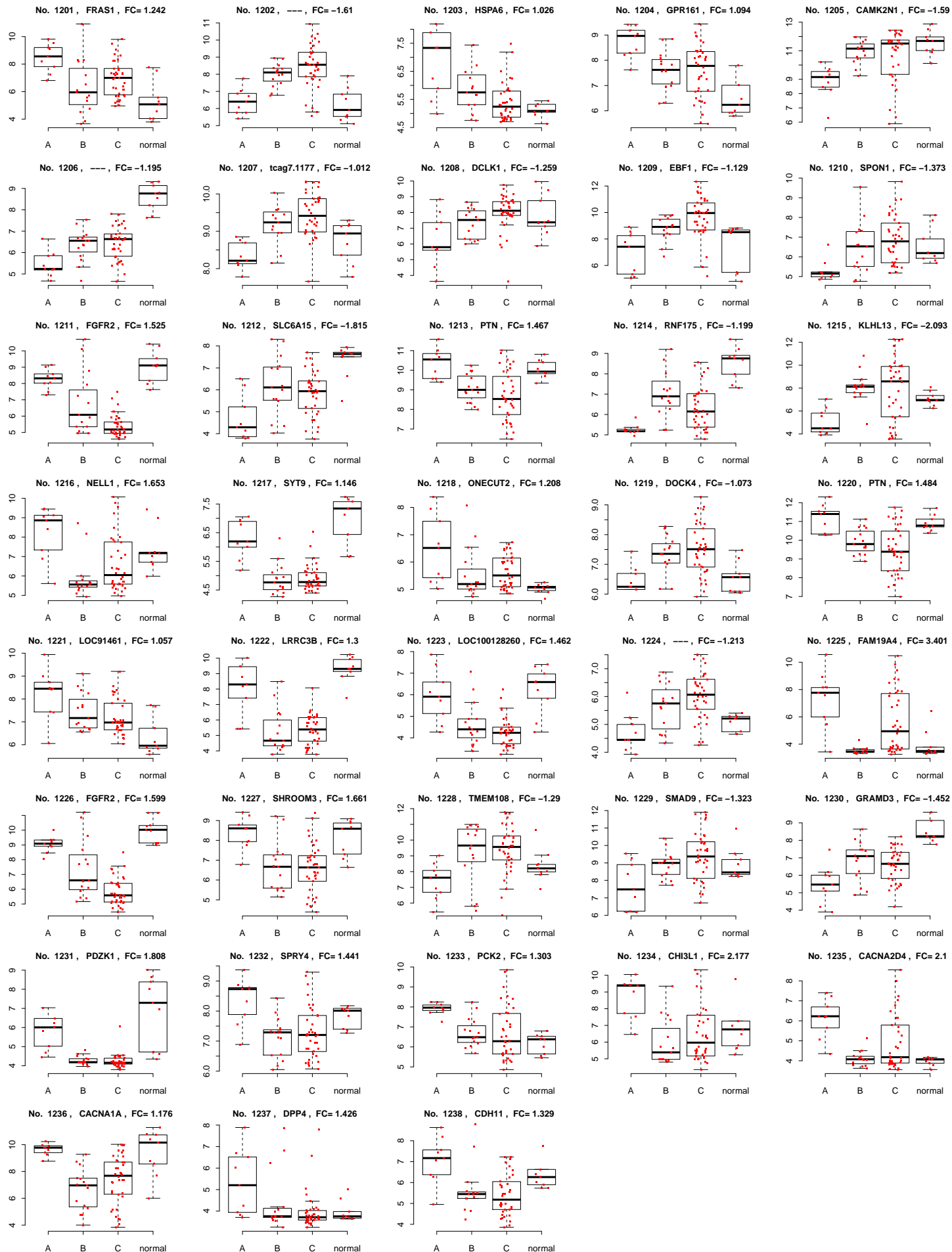

Supplement: Additional file 2 — The converged signatures for the subtypes of the three datasets. [file 1471-2105-14-S18-S1-S2.zip › plot-Kool62-SubtypeA.pdf]

# Northcott90

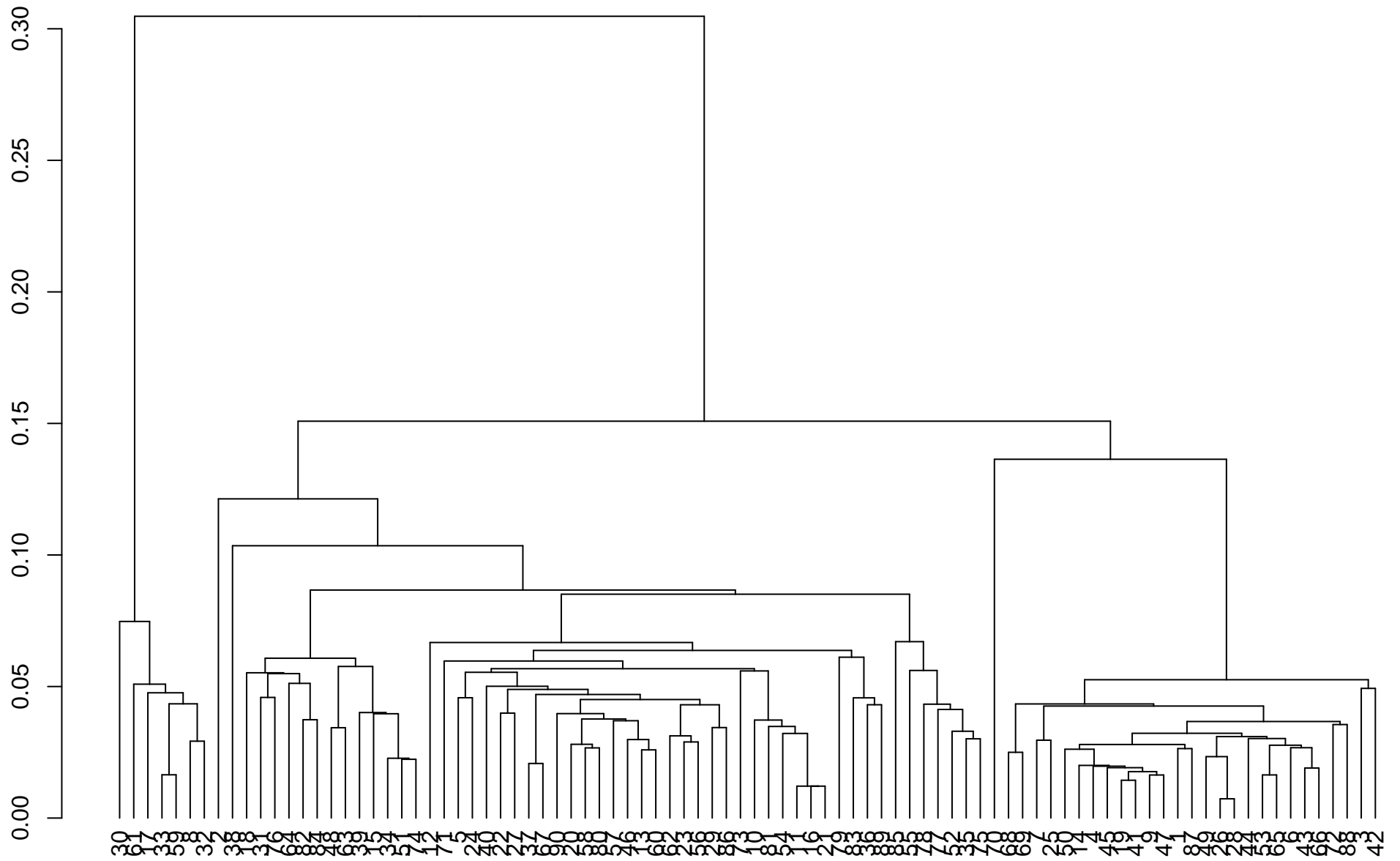

Supplement: Additional file 2 — The converged signatures for the subtypes of the three datasets. [file 1471-2105-14-S18-S1-S2.zip › convergeMap-Northcott90-dendrogram.pdf]

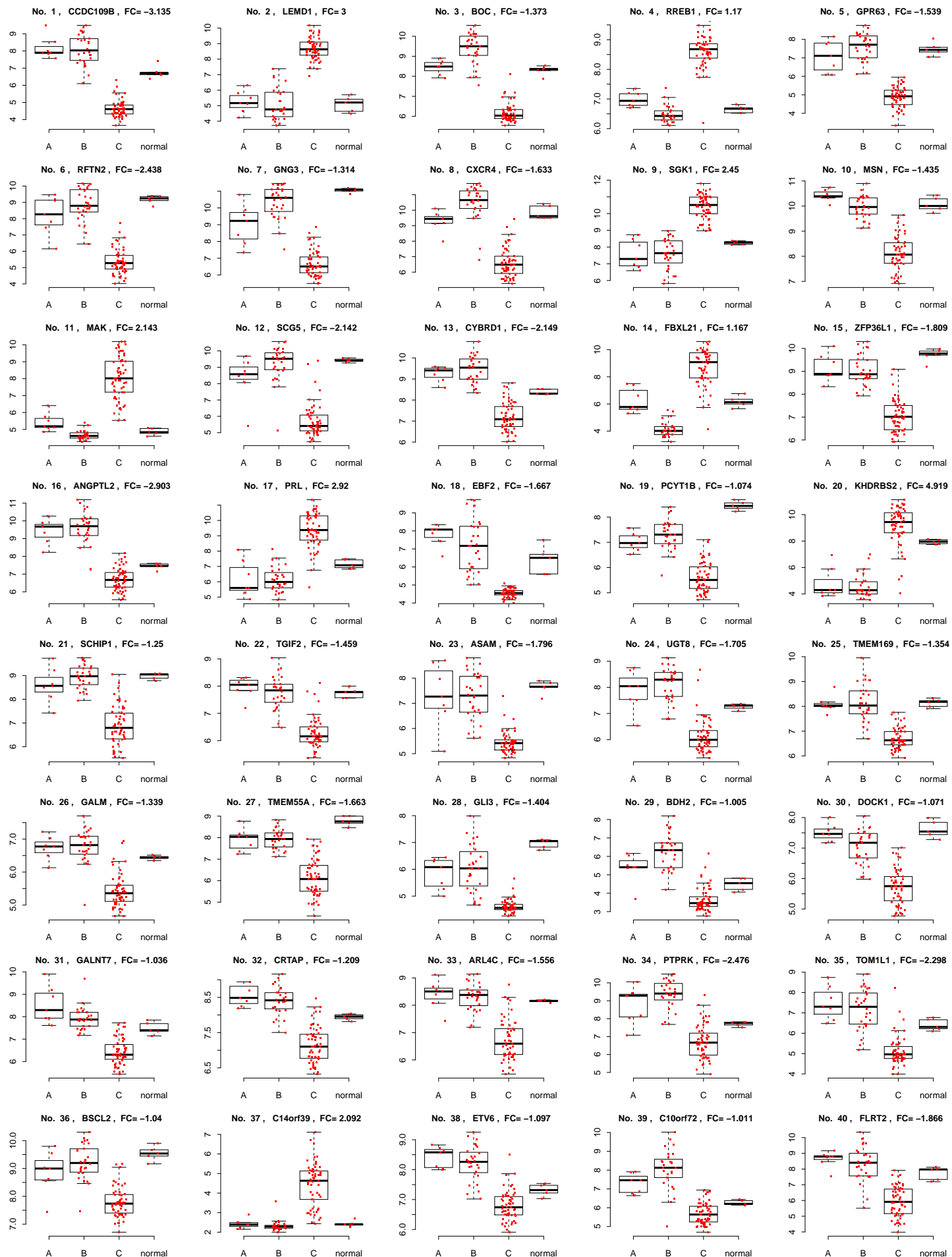

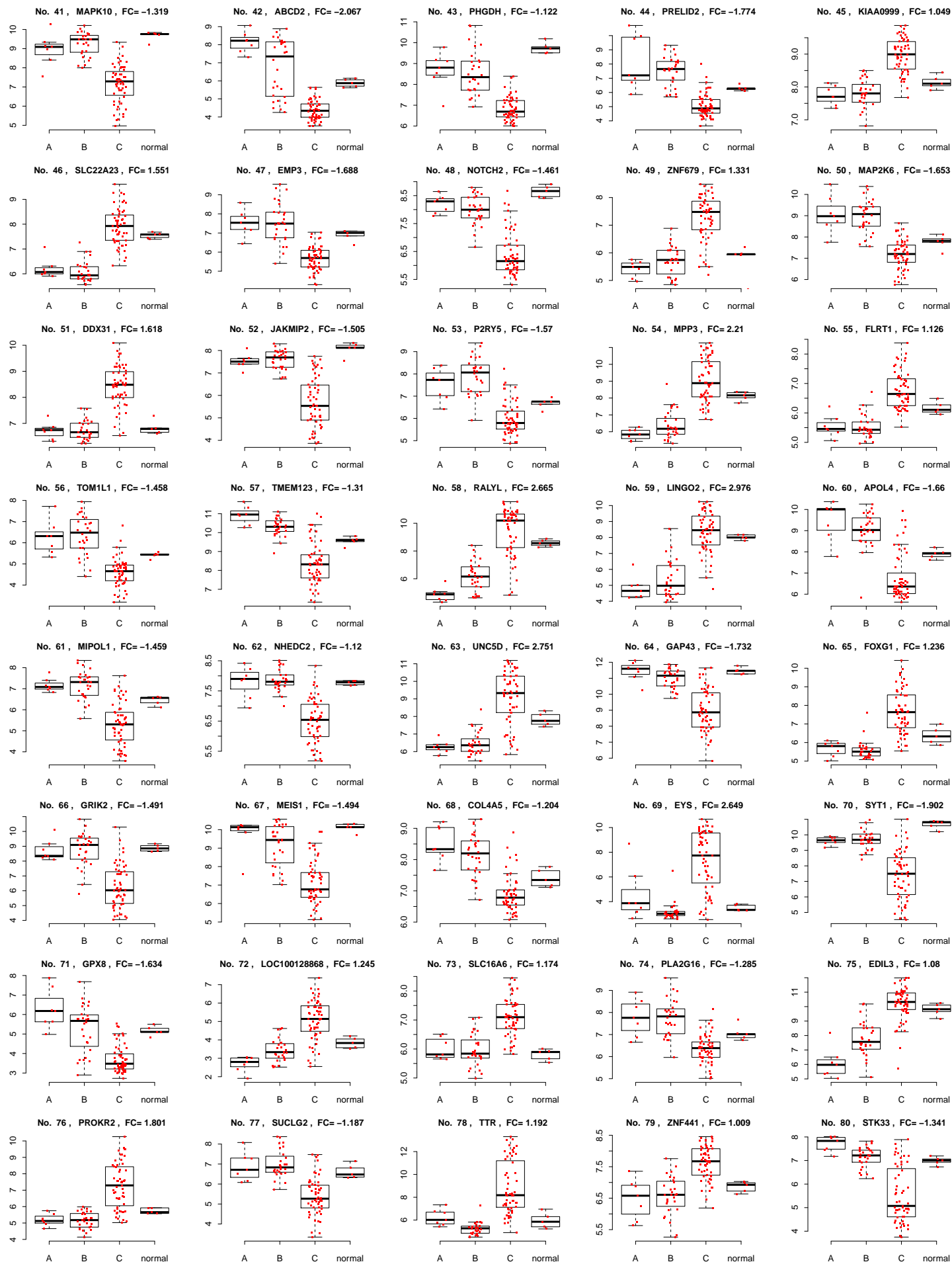

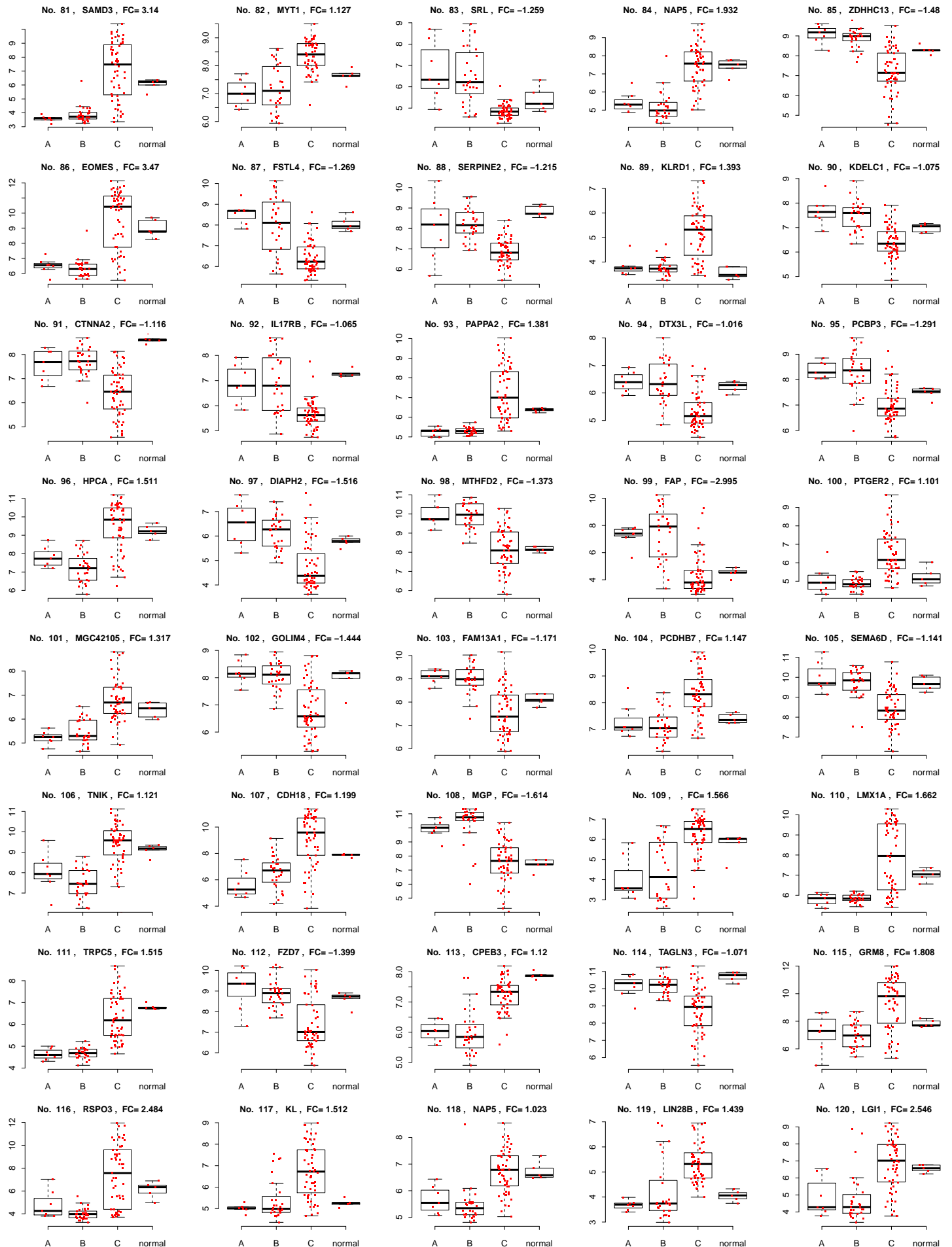

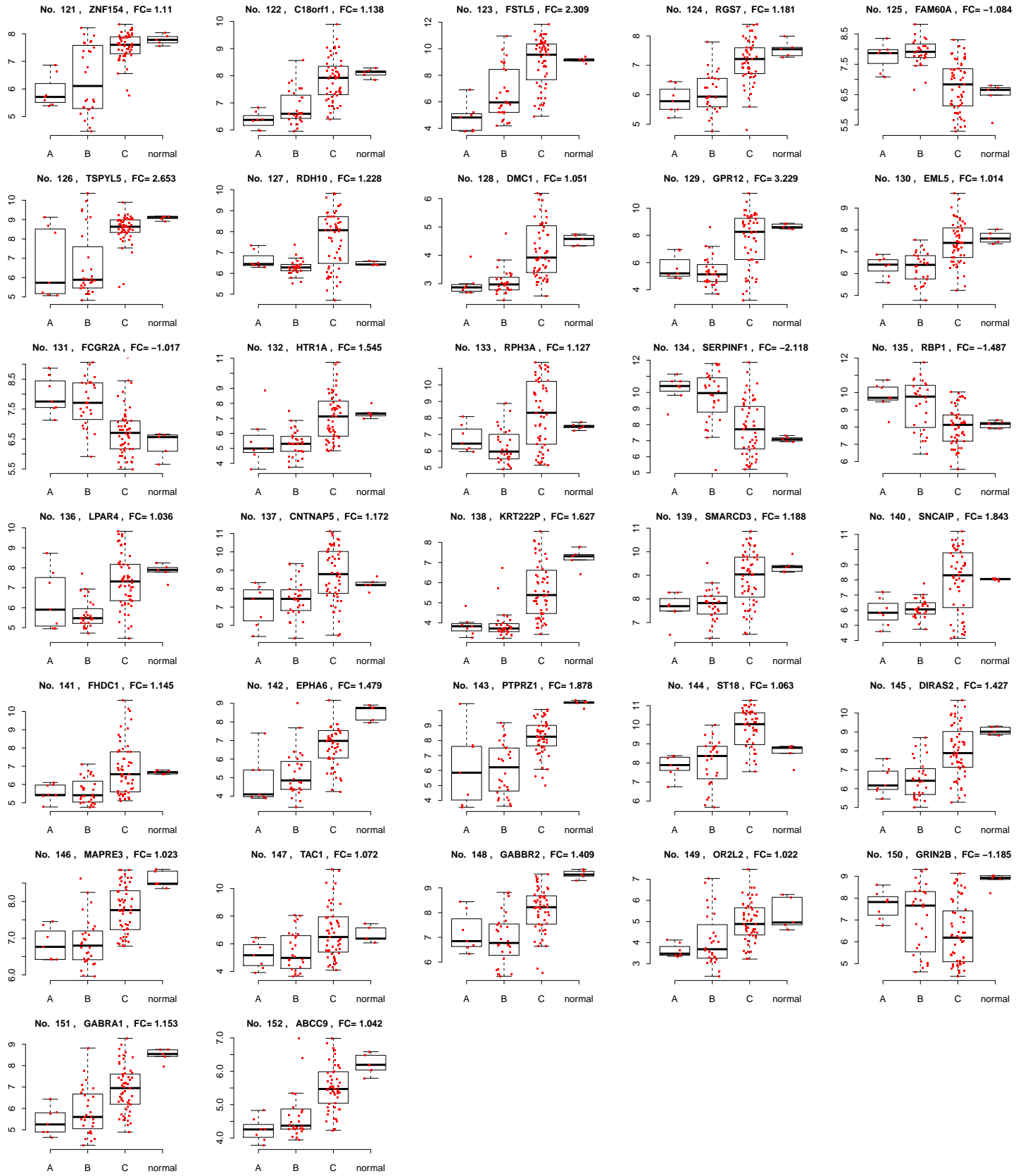

Supplement: Additional file 2 — The converged signatures for the subtypes of the three datasets. [file 1471-2105-14-S18-S1-S2.zip › plot-Northcott90-SubtypeC.pdf]
